# Treatment for preschool age children who stutter: Protocol of a randomised, non-inferiority parallel group pragmatic trial with Mini-KIDS, social cognitive behaviour treatment and the Lidcombe Program—TreatPaCS

**DOI:** 10.1371/journal.pone.0304212

**Published:** 2024-07-11

**Authors:** Anne-Lise Leclercq, Veerle Waelkens, Ella Roelant, Mathias Allegaert, Iris Verhaegen, Kim Claes, Estelle Dauvister, Steffi Snijders, Kurt Eggers, Astrid Moyse, Sabine Van Eerdenbrugh

**Affiliations:** 1 Research Unit for a life-Course perspective on Health and Education (RUCHE), University of Liège, Liège, Belgium; 2 Department of Speech and Language Pathology, Artevelde University of Applied Sciences, Ghent, Belgium; 3 Clinical Trials Center, Antwerp University Hospital (UZA), Antwerp, Belgium; 4 Center of Expertise Care and Wellbeing, Thomas More University of Applied Sciences, Antwerp, Belgium; 5 Department of Rehabilitation Sciences, Ghent University, Ghent, Belgium; 6 Department of Psychology and Speech-Language Pathology, Turku University, Turku, Finland; PLOS: Public Library of Science, UNITED KINGDOM

## Abstract

Stuttering is a speech disorder in which the flow of speech is disrupted by involuntary repetitions of sounds, syllables, words or phrases, stretched sounds or silent pauses in which the person is unable to produce sounds and sound transitions. Treatment success is the highest if stuttering is treated before the age of 6 years, before it develops into “persistent” stuttering. Stuttering treatment programs that focus directly on the speech of the child, like the Lidcombe Program, have shown to be effective in this age group. Mini-KIDS is also a treatment that focuses directly on the speech of the child. It is possible that capturing the increased brain plasticity at this age in combination with creating optimal conditions for recovery underlie these treatments’ success rate. A treatment focusing on the cognitions, emotions and behaviour of the child, the social cognitive behaviour treatment (SCBT), is also frequently delivered in Belgium. In this study we want to compare, and collect data on the effectiveness, of these three treatment programs: Mini-KIDS, SCBT and the Lidcombe Program (protocol registered under number NCT05185726). 249 children will be allocated to one of three treatment groups. Stuttering specialists will treat the child (and guide the parents) with Mini-KIDS, the SCBT or the Lidcombe Program. They will be trained to deliver the programs meticulously. At 18 months after randomisation, the speech fluency of the child and the attitude of the child and parent(s) towards speech will be measured. It is expected that the three programs will achieve the same (near) zero levels of stuttering in nearly all children and a positive attitude towards speech at 18 months after the start of treatment. The amount of treatment hours to reach the (near) zero levels of stuttering will be compared between the different programmes. For families as well as for the health system this could generate important information.

## Introduction

The World Health Organisation (Version: 01/2023) characterises developmental speech fluency disorder by

“frequent or pervasive disruption of the normal rhythmic flow and rate of speech characterised by repetitions and prolongations in sounds, syllables, words, and phrases, as well as blocking and word avoidance or substitutions. The speech dysfluency is persistent over time. The onset of speech dysfluency occurs during the developmental period and speech fluency is markedly below what would be expected for age. Speech dysfluency results in significant impairment in social communication, personal, family, social, educational, occupational or other important areas of functioning. The speech dysfluency is not better accounted for by a Disorder of Intellectual Development, a Disease of the Nervous System, a sensory impairment, or a structural abnormality, or other speech or voice disorder”

(ICD-11, 6A01.1).

Stuttering at preschool age is very different from stuttering in older children, adolescents and adults. A distinction between early childhood stuttering and persistent stuttering is therefore useful. Developmental stuttering typically appears in children between two and five years of age [1, 2], with about 95% before the age of 4 years [3]. The cumulative incidence of stuttering in preschool children is approximately 8% by the age of 3 years [3, 4]. About 74% of the children recover from (early childhood) stuttering within the first 4 years after stuttering onset [5]. Waiting for natural recovery to occur, however, is not current practice as evidence suggests that (1) stuttering treatment is most effective at preschool age, (2) persisting stuttering (after the age of 6) is not only more difficult to treat successfully, but also increases the risk of developing mental health problems, such as (social) anxiety disorders and (3) it is not possible to predict in which children stuttering will persist or not (e.g., [6]).

An additional reason not to wait for natural recovery to occur, is the finding that only a small proportion of the children who starts to stutter recovers without treatment within the first year after stuttering onset. More specifically, five (of 84 = 6%) recovered in 12 to 17 months after onset without treatment or with minimal stuttering management advice in a study of Yairi and Ambrose [5]. Also, preschool children become increasingly more aware of their stuttering when they get older (e.g., [7, 8]).

It is unclear what type of treatment stuttering specialists deliver most frequently to preschool age children who stutter (PCWS) in Belgium. However, from recent studies [9–11], it became clear that stuttering specialists in Belgium regularly deliver the Lidcombe Program, Mini-KIDS and Social-Cognitive Behaviour Treatment (SCBT) to PCWS. The Lidcombe Program and Mini-KIDS are both treatment programs for PCWS, directed at the speech of the child. A reduction of stuttering is the main aim of these two treatments. Just as in other treatment programs for PCWS, the parents are intensively involved and are psycho-educated about stuttering. These direct treatment programs give attention to social and cognitive aspects but do not focus primarily on them in treatment; the primary focus of direct treatment programs is on the motor aspects of the speech. In SCBT, the primary focus is on conditioning and cognitive training of emotions and cognitions, desensitisation (emotional training) and skill training of the PCWS and parents about stuttering and speaking.

### Rationale

Stuttering can have a significant impact on children’s social and emotional development [12]. PCWS can show signs of social discomfort [13, 14]. At school age, when the stuttering has become persistent, children who stutter are sometimes considered as less popular by peers, have an increased risk to be bullied, can experience more fear and can be more worried than peers who do not stutter [15, 16]. Teenagers and adolescents who stutter report having difficulties when communicating with peers and to belong to a group; some of them develop a low self-esteem [17]. Adults who stutter have a seven-fold increased risk to develop a social anxiety problem [18] and do not seem to have the same opportunities in their professional life compared to adults who do not stutter [19]. Also, treatment for early childhood stuttering (before the age of 6 years, so at preschool age) indicates to achieve the best possible results compared to stuttering treatment at older ages (e.g., [20]). So, it is clear that timely intervention, that is at preschool age, is necessary to reduce and avoid negative social implications and the development of social anxiety disorders, and to achieve the best possible treatment outcome for stuttering.

It is difficult, however, to predict which preschool age child will recover from early childhood stuttering without treatment (= natural recovery) and which child won’t. Therefore, a period of (active) monitoring is often the first step before initiating treatment [21] but needs to be limited in time to initiate treatment in a timely manner, i.e., before the age of 6 [22].

Evidence for the effectiveness of treating stuttering in preschool age children with direct, operant interventions is growing [20, 23–25]. Stuttering interventions directed at the speech of PCWS are introduced more frequently nowadays as option for the treatment of stuttering in preschool age children in Europe (e.g., [6, 26–28]).

Given the fact that, in Belgium, SLTs deliver predominantly one of these three treatment approaches for stuttering in PCWS, the first goal of the study is to assess the treatment outcome of those three treatments. Furthermore, their effect compared to each other is not known. The ‘Treatment for Preschool age Children who Stutter’ (TreatPaCS) trial is thus necessary to support current practice and determine which approach would lead to as much recovery of early childhood stuttering as possible before the age of 6 years (‘the window of opportunity’) and before stuttering becomes persistent.

We estimate, based on existing literature findings, that the three treatment programs will achieve similar outcome at 18 months post-treatment initiation [13, 29, 30]. That is, (near) zero levels of stuttering and linked to this result, a high score on Quality-of-Life scales (QOL), a positive attitude towards communication and no or low impact of stuttering on the preschool child and their parents.

As a secondary objective, we expect a significant difference in treatment time necessary to reach the treatment goals. We believe that the direct treatment programs (the Lidcombe Program and Mini-KIDS) need less treatment time (measured in hours) than SCBT with a reduction of about 1/3 to nearly ½ of the treatment time. This estimation is based on the available publications [13, 26, 29, 31–33]. This objective will have implications on families, costs and time.

## Materials and methods

### Trial design

This study is a three arm, 1:1:1 randomised, open-label pragmatic comparative trial comparing Mini-KIDS, SCBT and the Lidcombe Program. To show non-inferiority of Mini-KIDS compared to Lidcombe Program and SCBT compared to Lidcombe Program a parallel group design will be used. Stratified randomisation will be used according to treatment site (30 sites) and sex.

Recruitment began the 1^st^ of April 2022 and is ongoing until 249 preschool age children are randomised in the study.

The protocol was approved by the Ethics Committee of University Hospital Antwerp (UZA)/UAntwerpen on March 15^th^, 2022 (Project ID 3264). Informed consent is obtained from the participants in written format. The protocol approved at the start of the study (S1 Protocol), the Ethics’ approvals (S1–S3 Files) and the funder’s review process and agreement (S4 File) can be consulted in the supplementary information.

The study was reported following the SPIRIT Checklist (S1 Checklist).

### Participants

The TreatPaCS study takes place in both the Dutch-speaking and the French-speaking parts of Belgium. It is a multicentre trial composed of 30 sites (one site = one SLT). Each site is a private practice specialised in stuttering and each SLT is experienced in treating PCWS with at least two years of experience. SLTs were prepared to deliver the three treatment approaches and attended one workshop for each treatment. The participating SLT recruits the PCWS when the parent(s) contacts the SLT’s private practice.

Eligible participants are preschool age children who stutter aged between 2 and 6.5 years at screening. They have, if bilingual, a parent who speaks a language that the SLT understands and speaks to allow clear communication. They also have at least one parent agreeing to be intensively involved in treatment and knowing that they will implement the treatment at home and who is willing and able to video record their child seven times during the treatment. Participants have no hearing loss, as reported by the parent(s). The trial excludes children with a syndrome but not children with comorbidities (Autism Spectrum Disorder, ADHD, language disorders, …). Written informed consent is obtained from at least one parent of the participating child and oral consent is obtained from the child himself.

#### Sample size

The first aim of the trial is to prove non-inferiority of Mini-KIDS compared to Lidcombe Program and SCBT compared to Lidcombe Program. The primary outcome is %SS at 18 months post-treatment initiation. The non-inferiority margin was set at 1.0% SS following Bridgman et al. [32] and Donaghy et al. [33]. Several studies report a standard deviation on %SS at 18 months varying from 0.4 to 2.1 in different number of children [26, 31–33]. Pooling all these results (weighing them with the number of children per study), a value of 1.7 was estimated for the standard deviation at 18 months on %SS. The usual one-sided significance level of 2.5% was adjusted to 1.25% because two comparisons will be considered (Mini-KIDS versus Lidcombe Program and SCBT versus Lidcombe Program). Assuming that in reality, there is no difference between the three programs, and using a power of 90% and a one-sided significance level of 1.25%, 73 children are needed in each group to show non-inferiority. Taking into account a 11% dropout [26], 249 children in total (83 children per arm) have to be randomised.

### General procedure

**Fig 1:**
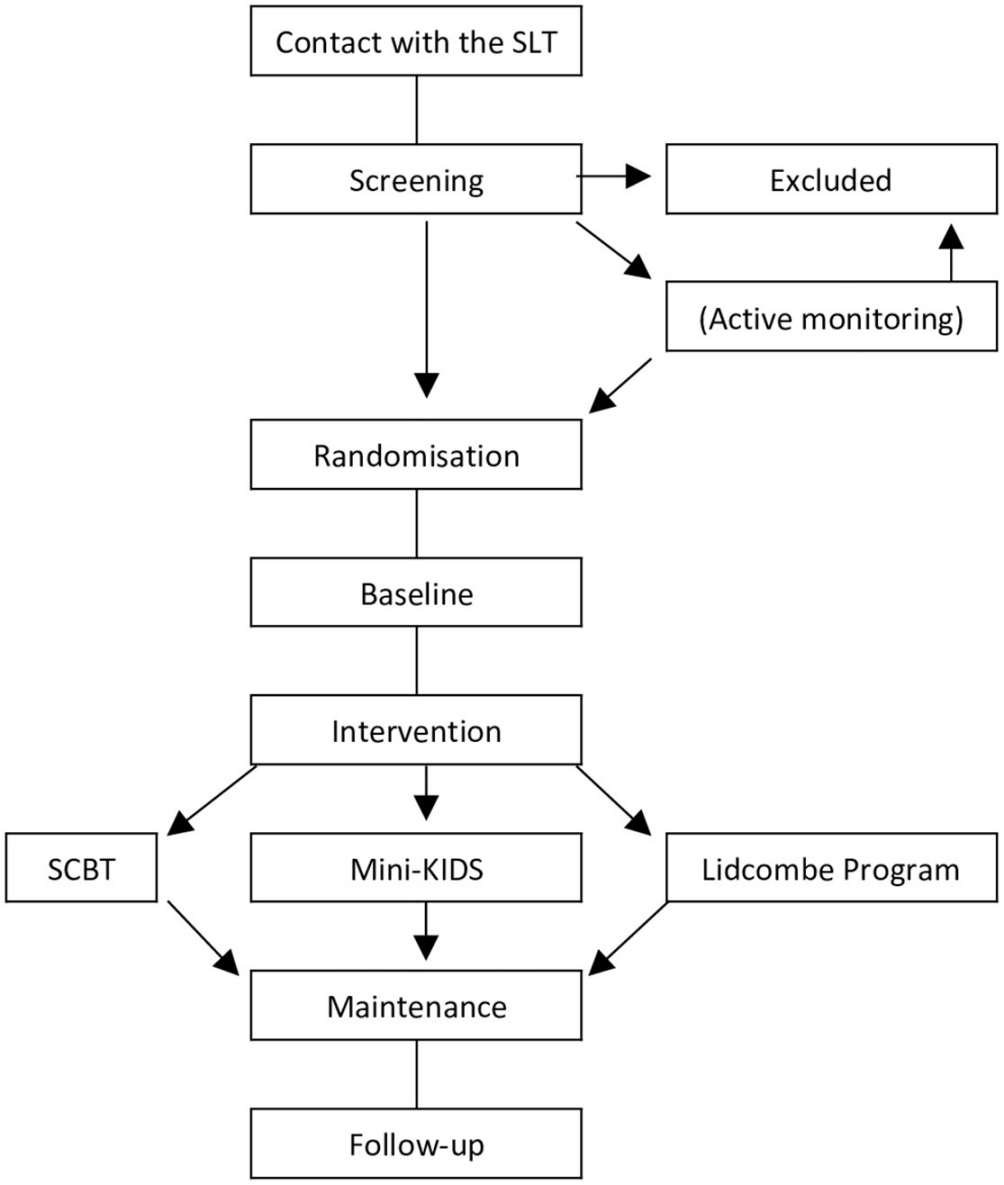
Flowchart of participants.

**Fig 2:**
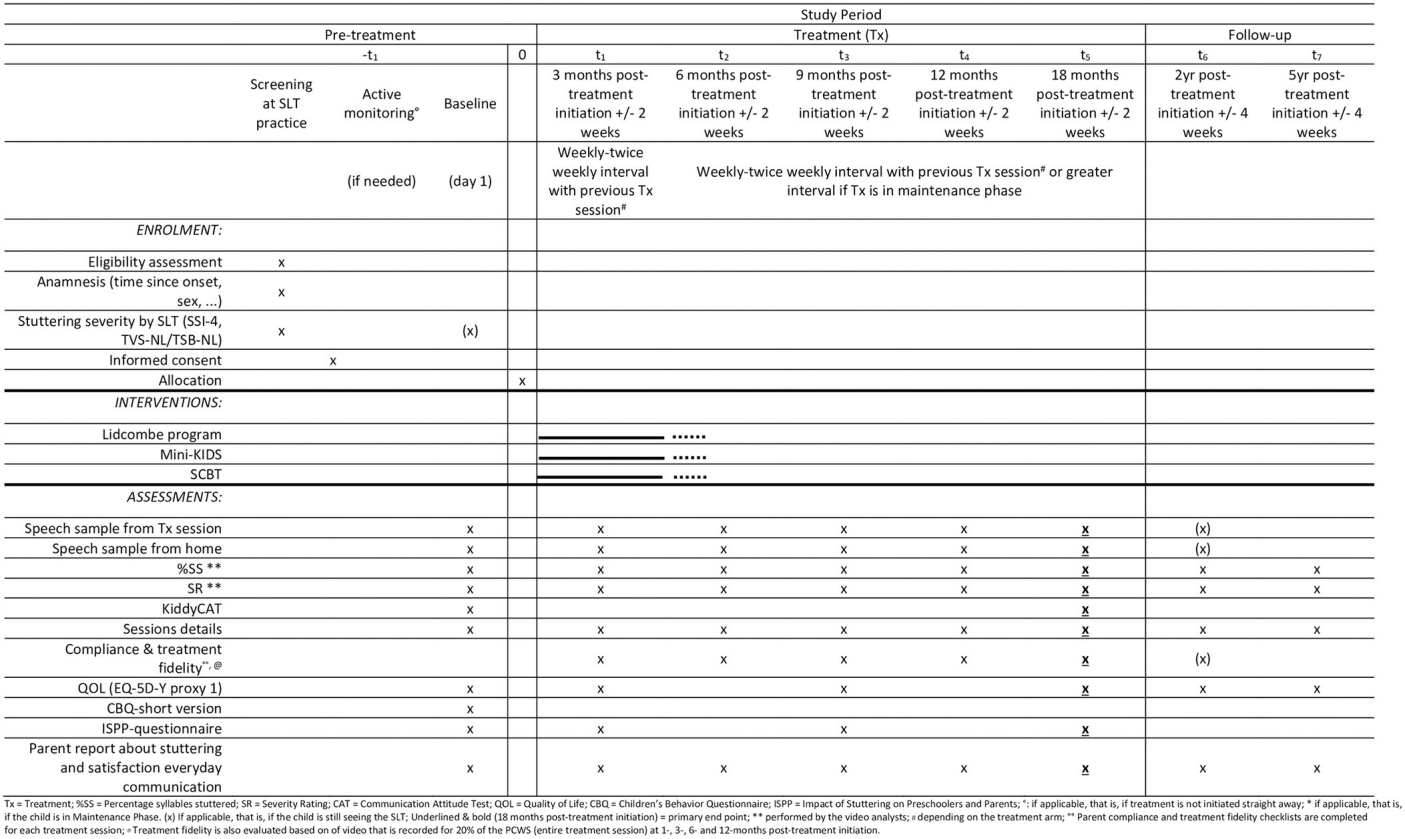
Schedule of enrolment, interventions, and assessments in TreatPaCS.

An overview of the schedule of enrolment, intervention and assessments can be seen in Fig 1 (S1 Fig). A flowchart of participants and of the study design is shown in Fig 2 (S2 Fig).

#### Screening

After being contacted by a parent, the SLT identifies the stuttering in the PCWS with the measuring instrument Stuttering Severity Instrument-4 (SSI-4, [34]) or the Test for Stuttering Severity-Non Readers, available in Dutch and French (TVS-NL, [35]; TSB-NL; [36]) during a screening session. The SLT also collects information about the PCWS, their stuttering and the parent(s) through an anamnesis form and screens the family for the inclusion and exclusion criteria. Parent(s) are educated about stuttering to decrease their level of concern if necessary. Based on the age of the PCWS, the severity of the stuttering, the family history of the stuttering and the evolution since onset, the SLT decides which action needs to be taken: (1) install an active monitoring period of maximum 3 months, (2) initiate stuttering treatment or (3) not initiate treatment.

#### Period of active monitoring (optional)

A period of active monitoring (up to 3 months) can be installed if the stuttering shows signs of natural recovery. The SLT records the start and the end date and information concerning the stuttering reported by the parent(s) as required by the SLT. During this period, the SLT may provide advice to the parent(s) but s/he does not model treatment techniques or introduce techniques that require clinical follow-up. The SLT records the advice that has been given.

#### Randomisation and blinding

After successfully completing the screening or at the end of an active monitoring period and once the informed consent is signed by the parent(s), the SLT fills out a request for randomisation in the electronic case report form system (eCRF). The randomisation form contains the unique study ID, year of birth and sex. Thereafter the database generates an automatic email to a staff member from the clinical trial center (CTC–Antwerp University Hospital) about the request including the unique study ID from the database. The allocation is performed in chronological order (based on the timing of the automatic emails from the eCRF). A web-based randomisation system QMinim is used for allocation of the participants. Participants are assigned following a stratified minimisation procedure. The information is made available in the database and can be viewed by the SLT after completing the baseline. The PCWS can start treatment the next day.

The participants (PCWS and their family) as well as the SLTs are not blinded to the treatment approaches that they receive or deliver. The information is recorded in the PCWS’s speech-language therapy file and the electronic Case Report From (eCRF). The video analysts who assess the French and Dutch video recordings for treatment fidelity are also not blinded. In contrast, the video analysts who are scoring the percentage of stuttered syllables (%SS) and severity rating (SR) (see below) are blinded to the treatment received.

#### Baseline

The baseline session takes place directly after screening or after the period of active monitoring if a stuttering treatment has to be installed. Parents give their informed consent between the screening and the baseline session. They then record a video speech sample from a home situation and answer questionnaires about the quality of life (EQ-5D-Youth proxy 1), the child’s temperament (Children’s Behavior Questionnaire—CBQ-short version) and the impact of stuttering (Impact of Stuttering on Preschoolers and Parents–ISPP-questionnaire).

The baseline session starts with a conversation between the PCWS and the parent(s) and/or SLT which is video recorded. Both videos, from home and from SLT, are about 10 to 15 minutes of conversation in which the child produces at least 300 syllables and no treatment is provided. The %SS and SR are scored by a blinded video analyst at a later time. The PCWS is assessed for speech attitude (KiddyCAT, [37]). Parents are asked to report on their child’s stuttering severity based on a 10-point scale [38] and their satisfaction of the PCWS’ communication in everyday speaking situations based on a 9-point scale adapted from Karimi et al. [39].

Once all information is collected and encoded into the trial database, the allocated treatment arm is revealed to the SLT and family (see randomisation above). If randomised in the Mini-KIDS arm, additional instruments are administered during the first session with parent(s) and/or child to measure the Reaction of the PCWS on the pseudo-Stuttering of the Examiner (RSE-1 and RSE-2), the Reaction on Communicative Stressors (RCS), the parent-child interaction (with a checklist used in the Restart-DCM program), and the Reaction Of the parent on the Stuttering of the Child (ROSC).

If randomised in the Lidcombe Program, parent(s) are introduced the 10-point stuttering rating scale [38] to record daily severity ratings of the PCWS’ typical stuttering throughout the day. A stuttering severity chart is provided to record the daily scores at the end of each day.

### Interventions

Participants will receive one of the three treatment arms according to the randomisation: SCBT, Mini-KIDS or the Lidcombe Program. A flow chart is given in Fig 1.

#### Description of the SCBT

The SCBT includes parent training [40] and treatment sessions with the child. The parent training usually comprises 7 to 10 one-hour individual or 90-minute group sessions for parents to discuss and offer education topics about stuttering. The parent sessions aim at changing parents’ attitude towards stuttering by desensitising and cognitive restructuring. The SCBT contains five treatment phases: (1) conditioning speaking activities, (2) cognitive training focused on emotions, (3) cognitive training focused on cognitions, (4) desensitisation (emotional training) and (5) skill training [40]. This treatment is not directed at the speech of the children, but rather at the cognitive and emotional aspects that surround the stuttering. Treatment sessions of 30 minutes are scheduled usually on a twice weekly basis, depending on the parents’ availability. These treatment sessions are attended by at least one parent and the PCWS. During treatment sessions, the parent(s) is encouraged to join the interaction between the child and the SLT and is asked to implement the techniques at home two or three times per week for 20 to 30 minutes. These techniques need to be implemented at least two times per week for 3 months, followed by once per week for another 3 months.

The PCWS can conclude the treatment phase and move to the maintenance phase when (1) the stuttering does not develop further, (2) there is a reduction of stuttering severity during at least 3 to 4 months, (3) the PCWS developed a positive attitude towards his/her speech, (4) the PCWS is resilient towards stuttering-inducing factors and (5) the parent(s) provides a more stable and fluency-inducing behavioural model environment to the PCWS and deals independently and appropriately with the PCWS’s stuttering. Usually, the stuttering is reduced to (near) zero stuttering.

#### Description of the Mini-KIDS

Mini-KIDS is a direct treatment based on principles of stuttering modification through pseudo-stuttering, that is, deliberate stuttering, as one of the main components. The program for 4- to 6-year old children consists of four phases: (1) desensitization, (2) identification, (3) modification and (4) generalization (maintenance phase). The program for 2- to 4-year old children does not include phase two. SLTs and parent(s) serve as a speech model for the child by adding normal dysfluencies and pseudo-stuttering to their speech. Later on in treatment and if necessary, children learn to recognise and alter their stuttering moments. Along with the direct work on speech (daring to stutter and being able to stutter easily) in the four phases, also risk factors that trigger stuttering are addressed, either with parents or directly with parents and child. The aim is that parents are competent to address these risk factors in the future once treatment has ended. The Mini-KIDS approach is based on the therapy concept KIDS (‘Kindern Dürfen Stottern’, [41]) for school-aged children, proceeding from a stuttering modification approach for adults. This approach combines (1) supporting metacognitive and metalinguistic skills, (2) desensitisation and (3) direct modification of speech motor loss of control. Mini-KIDS as entire program is not yet supported by research-based evidence, only practice-based evidence. Each of these components, however, has been validated in independent contexts. Pseudo-stuttering has been identified as an efficient mean to use with PCWS [42, 43] to work on metacognitive and metalinguistic skills, which underlie the direct work on desensitization. Desensitisation has also been found to be effective in the treatment of PCWS [44–46]. Finally, research on brain function and motor speech development argue in favour of direct work on modification of the speech motor loss of control in young children (e.g., [47–50]).

The Mini-KIDS treatment guide [29] prescribes treatment sessions of 60 minutes for Phases 1 to 4. This, however, is not compatible with standard Belgian care as only treatment sessions of 30 minutes with parent and PCWS are reimbursed. Therefore, this study limited the one-hour sessions to the first six treatment sessions. These sessions are scheduled with one PCWS and his/her parent(s). The parent(s) receive(s) education about stuttering and is(are) coached to implement pseudo-stuttering techniques, also in in vivo situations.

#### Description of the Lidcombe Program

The Lidcombe Program [38] is an operant program that directly provides verbal feedback to the child’s stutter-free speech (mainly) and the child’s stuttering (occasionally). Parents implement the treatment at home, in the daily environment of the child. The Lidcombe Program comprises two phases: phase 1 in which (near) zero levels of stuttering are achieved and phase 2 in which the achieved (near) zero levels of stuttering are maintained for a long period of time. The Lidcombe Program usually takes between 11 to 23 (45 to 60-minute) treatment sessions to achieve the goals of phase 1, i.e. (near) zero levels of stuttering [38]. The Lidcombe Program is recommended as the treatment option with the strongest evidence in (systematic) reviews to date [20, 24, 25, 51–54] and the Dutch clinical guideline [6]. Treatment with the Lidcombe Program does not seem to impact on the quality of the attachment between children and the parents who implement the treatment [55]. It also does not impact negatively on the speech and language of the parents nor the children [56, 57]; on the contrary, children seem to increase their linguistic complexity over the course of the treatment [58]. A meta-analysis of two clinical trials [59, 60] and two clinical experiments [58, 61] conducted with the Lidcombe Program [30] involved 134 children. The children in the control groups received the Lidcombe Program after 9 months of no treatment. At pre-randomisation the stuttering severity of the group who received the Lidcombe Program and the control group were about the same. There was a small average improvement in the control group over time due to natural recovery, which was predictable [62]. However, at a mean post-randomisation period of 6.3 months, the group who received the Lidcombe Program did significantly better than the control group. The odds ratio at that point in time was 7.5 for achieving % Syllables Stuttered (%SS, a measurement for the frequency of stuttering) of below 1.0 for the Lidcombe Program group versus the control group.

All sessions in phase 1 take 45 to 60 minutes according to the Lidcombe Program treatment guide [38]. Sessions of 45 to 60 minutes with parent and PCWS, however, are not reimbursed through the Belgian health care system, only sessions of 30 minutes. Therefore, in this study, one-hour sessions were limited to the first six treatment sessions. These sessions are scheduled weekly with the PCWS and his/her parent(s). The parent receives education about stuttering and is coached to implement the treatment techniques. During phase 1, parents learn to provide verbal contingencies during daily, 10-minute practice sessions with their PCWS. For unambiguous stutter-free speech, parents can praise, acknowledge the stutter-free speech or formulate a request for self-evaluation to the PCWS. The practice sessions that the parent(s) implement at home aim to give the PCWS the opportunity to practise stutter-free speech. When the verbal contingencies for stutter-free are provided appropriately and effectively, the verbal contingencies for stuttering are also introduced. These are an acknowledgement of the stuttering moment or a request for self-correction. The parent daily records a severity score (SR) score of the stuttering.

Later on, the practice sessions at home become more natural. They resemble everyday conversations. Also, a limited number of verbal contingencies are given throughout the day. When the PCWS has typical scores of 0 and 1 on the severity rating scale with more 0s than 1s per week for 3 consecutive weeks, and the stuttering is rated as 0 or 1 during the treatment session, the PCWS can proceed to phase 2 (see maintenance phase).

#### Description of the maintenance phase

In this study, the maintenance phase is similar for each treatment. Treatment sessions to monitor if achieved goals are maintained are scheduled with an interval of 2, 2, 4, 4, 8, 8 and 16 weeks. If necessary, SLTs add extra sessions during maintenance. During the maintenance sessions, the SLT asks the parent(s) to report about the PCWS and observes the speech of the PCWS. Depending on the speech of the PCWS, a decision is taken: (1) if the PCWS does not stutter anymore, the parent(s) continues to observe closely but there is no need to re-introduce treatment techniques. (2) If the PCWS responds with stuttering to stuttering-inducing factors, the parent(s) reinstates specific treatment techniques depending on the situation or the PCWS receives training on certain concepts or skills. (3) If the PCWS stutters severely: the SLT may decide to re-introduce specific treatment concepts or skills and may ask the parent(s) to implement them at home.

#### Fidelity of implementation

In treatment for PCWS, the treatment is not only implemented during the treatment session by the SLT, but also at home by the parents. It is therefore necessary to check compliance of implementation at home and during the treatment session. Fidelity of implementation is checked at two levels: (1) the parent’s compliance and quality of implementation (through compliance checklists and observation) and (2) the SLT’s treatment delivery in the practice (for treatment fidelity; treatment fidelity is checked through treatment fidelity checklists and video recordings of the treatment sessions).

#### Data collection points

Screening and baseline sessions are the two first collection points. Once the treatment has been initiated, data is collected 3-, 6-, 9-, 12- and 18-months post-treatment initiation with a tolerance interval of 2 weeks before or after the exact point. Additional data is collected 2- and 5-years post-treatment initiation as a follow-up. An overview of measurements for each data collection point can be seen in Fig 2.

### Outcomes

#### Primary outcome

The primary endpoint is the %SS at 18 months post-treatment initiation. Percentage of stuttered syllables (%SS) is measured by the blinded video analysts on video recorded speech samples from home and from the treatment session and will be computed as the average of the two speech samples. The %SS will be compared between the speech samples of PCWS treated with Mini-KIDS, SCBT and the Lidcombe Program. The 18-months post-treatment initiation time point was chosen to enable all participants to complete the treatment phase and achieve the goals of each treatment approach.

#### Secondary outcomes

Secondary outcomes measures will be used to further investigate the relative efficacy of the three treatments. Percentage of stuttered syllables (%SS) is also measured at 3-, 6-, 9-, 12-months and 2 and 5 years post-treatment initiation. Severity Rating (severity rating, measured on video recordings by blinded video analysts) is measured at 3-, 6-, 9-, 12-, 18-months and 2 and 5 years post-treatment initiation. Whereas %SS measures the frequency of stuttering, SR measures frequency and severity. For example, a sentence spoken with one fixed posture with audible airflow (prolongation) combined with a non-verbal superfluous behaviour (e.g., grimace) will result in a higher SR than three monosyllabic repetitions. The proportion of children that are successful (defined as <1%SS and SR ≤ 1) is evaluated at 18-months and at 5 years post-treatment initiation.

The impact of stuttering on PCWS and parents (ISPP) is measured at 3-, 9- and 18-months post-treatment initiation. The quality of life (EQ-5D-Y proxy 1) is measured at 3-, 9- and 18-months and 2 and 5 years post-treatment initiation. Communication attitude of the child (KiddyCAT) is measured at 18-months post-treatment initiation. These three measures assess the covert aspects of stuttering, as opposed to the overt aspects (measured by %SS and SR). Parents are asked to report about their child’s stuttering severity and their satisfaction with their child’s everyday communication at 3-, 6-, 9-, 12- and 18-months, 2 and 5 years post-treatment initiation. The number of treatment time (in hours), number of weeks and number of treatment sessions are counted until the start of the maintenance phase of each treatment approach.

#### Exploratory endpoints

Profiles of children will be explored in order to determine whether a profile of children is more successful to one of the treatment approaches. Possible attributes that will be studied are attributes established at baseline such as sex, comorbidities, age at assessment, family history of stuttering, family history of recovery of stuttering, onset data (time since onset, progress since onset), answers to the ISPP-questionnaire, stuttering severity (%SS and SR), parent-related profile questions and temperament. A survey to capture the experience of the participating SLTs about delivering the three treatments will be sent to the SLTs before and after the study and will be included in a qualitative analysis.

### Statistical methods

Considering this is a non-inferiority trial, the primary endpoint is analysed in first instance in the per-protocol population. The per-protocol population are all eligible children who followed the treatment for at least 80%. To establish this, the SLT will carry out a fidelity checklist for each treatment session. To correct for the fact that the per-protocol population is a subset of the randomised population, a weighted linear regression model will be used with %SS at 18 months post-treatment initiation as outcome and program as predictor and weighting individuals with inverse probability weighting. The weights which are determined in the full dataset of adherers and non-adherers are estimated as an individual’s probability to adhere to a certain program given observed confounders like sex, age at assessment, comorbidities, family history of stuttering and recovery of stuttering, parent profile, baseline stuttering severity assessment (%SS and SR), and temperament (CBQ-short version). Post-hoc comparison of SCBT compared to Lidcombe Program and Mini-KIDS to Lidcombe Program using a two-sided 97.5% (correction for two comparisons) confidence interval for the difference in %SS will be compared to the non-inferiority margin.

Regarding secondary endpoints, different sensitivity analyses will be done to evaluate the differences between the programs at 18 months post-treatment initiation. A linear regression model with %SS as outcome and type of program as a predictor will be considered with inclusion of other factors like sex, age at assessment, comorbidities, family history of stuttering and recovery of stuttering, baseline stuttering severity assessment (%SS and SR), time since onset and temperament (CBQ-short version). Linear regression assumptions will be checked and if needed transformations or other models will be explored. This analysis will be done in the per-protocol (naïve per-protocol) as well as in the intention-to-treat population. For the intention-to-treat population children who drop out from the study get the last recorded %SS carried forward. In the secondary analysis, we will also consider the comparison between Mini-KIDS and SCBT.

As %SS is measured repeatedly (3-, 6-, 9-, 12-, 18- months and 2 and 5 years post-treatment initiation), we can consider a linear mixed model with child as random intercept to compare the evolution over time between the programs and to get an even more precise estimate of the difference at 18 months post-treatment initiation. SR (also averaged over home and treatment session sample), score on the KiddyCAT and EQ-5D-Y-proxy 1 at 18 months post-treatment initiation will be compared between the three programs using an analysis of variance and answers to the ISPP-questionnaire using a Chi-square test. In case of significance, two by two post-hoc comparisons will be considered and for SR confidence intervals will be compared to a non-inferiority margin of 1. These outcomes will also be studied in a multiple linear regression model (with confounders as mentioned before) with adjustment for their baseline values. The same analysis will be done for the EQ-5D-Y-proxy 1 scores at the long-term follow-up at 2 years and 5 years post-treatment initiation. For the continuous measures SR, EQ-5D-Y proxy 1, measured at different time points we can compare the evolution over time between the programs using a linear mixed model. This model also allows to look at differences at a specific time point in a post-hoc comparison. For the answers to the ISPP-questionnaire measured at different time points this can be done using a generalized linear mixed model. For the ordinal outcomes parent report about their PCWS’s stuttering severity and their satisfaction about the PCWS’s communication in everyday speaking situations depending on variability a linear mixed or generalized linear mixed model will be used. The number of weeks, the number of treatment sessions, the treatment time needed until the maintenance phase begins will be compared between the three programs using an ANOVA or Kruskal Wallis test as appropriate. This outcome will also be studied in a multiple linear regression model adjusting for possible confounders. The proportion of children that are successful (defined as <1%SS and SR ≤ 1) at 18 months and at 5 years post-treatment initiation will be compared by a Chi-square test (or Fisher’s exact test as appropriate) between the three programs. The time to reach success between the three programs will be compared with a Kaplan-Meier plot and analysed using a Cox regression model with correction for confounders. %SS and SR at 18 months post-treatment initiation will also be evaluated separately for the videos at home and for the videos recorded during the treatment sessions using linear regression models as described for the averages of the two samples.

For the above analysis, we will first consider the intention-to-treat population to preserve randomisation, if necessary also per-protocol population will be considered. Numbers of children who drop out and reasons for drop-out, as well as leave the allocated treatment arm (numbers, reason and switched treatment) will be described per treatment. Numbers per program will be compared using a Chi-square test (or Fisher’s exact test as appropriate). A time to drop-out analysis or a time to switch analysis will be performed in case >10% drop-out or a 10% switch was present in the trial. Finally, exploratory outcomes will be studied in a linear regression model with %SS at 18 months as outcome and in a logistic regression model with success defined as <1%SS and SR ≤ 1 as a binary outcome. Possible factors that will be studied in finding a profile of children more successful to one of the programs are sex, comorbidities, age at assessment, family history of stuttering, family history of recovery of stuttering, onset data (time since onset, progress since onset), answers to the ISPP-questionnaire, baseline stuttering severity assessment (%SS and SR), parent profile and temperament (CBQ-short version).

## Discussion

Evidence shows that stuttering treatment is most effective at preschool age and that persisting stuttering increases the risk of developing mental health problems [12–17]. For this reason, we are conducting a non-inferior RCT in Belgium with three treatments that are regularly delivered: the Lidcombe Program, Mini-KIDS and social-cognitive behaviour treatment (SCBT). Evidence regarding these treatments’ efficacy in PCWS is growing but needs to be improved. The present study will help strengthen the evidence.

The set-up of the current trial resembles the set-up of the RESTART-DCM RCT trial, but this current trial is grafted on the Belgian context. In Belgium, various treatments are currently used to treat stuttering in pre-school aged children. Like other healthcare professionals, SLTs are increasingly required to think about their clinical decision-making from an Evidence-Based Practice (EBP) perspective, taking into account the best recent scientific evidence when making choices about the care of individual patients. It is therefore essential to develop evidence of effectiveness for the treatments currently used in Belgium. Specifically, we expect that each treatment approach has the same efficacy 18-months post-treatment initiation regarding stuttering severity. However, it is possible that some are more effective than others, or that similar results can be achieved in different timelines. In addition to effectiveness in reducing stuttering, our data should provide information on the functional impact of treatment on the patient’s day-to-day functioning, his or her attitude toward speech and that of those around him or her. Evidence-Based Practice highlight the importance of making clinical decisions based on the clinician’s expertise, the context of care and the profile of each patient. Exploratory analyses of this trial could be of particular relevance by providing information about the profiles of patients or families who will benefit most from different types of treatment. In this way, this study will not only determine the effectiveness of the treatments, but also explore which patient characteristics need to be considered when choosing one of these treatments.

Given that the SLTs deliver the three treatment programmes, a potential risk is that the SLTs will add elements of one programme to other treatments. However, three measures have been developed to prevent this: firstly, treatment fidelity checklists have been created to remind speech therapists of the essential elements of each treatment that need to be covered at each session. Secondly, compliance checklists have been designed to enable speech therapists to check that parents are implementing the treatment at home. These two checklists must be completed by the speech therapists at the end of each session. Finally, regular video recordings of the treatment sessions enable one of the study coordinators to check the quality of the treatments administered.

Patient representatives were consulted during the development of the study and are regularly informed of the project’s progress. In particular, they suggested that we ask parents to regularly measure their satisfaction with their child’s speech.

One of the limitations of this study is that the implementation of treatments by the SLTs, in real practice settings, may differ from the ideal conditions for administering the treatment. In the real world of clinical practice, sessions are cancelled due to illness, holidays or unforeseen circumstances, leading to treatments which may be less effective than in the context of extremely controlled laboratory research. Despite this, one of the strengths of the TreatPaCS trial is that its results will be directly applicable to real-life clinical practice conditions. Indeed, previous research has shown that outcomes for the Lidcombe Program in general clinical practice may be comparable to those obtained in research clinical trials, but the process can take longer given that there are no restrictions in terms of comorbidity of early stuttering with speech and language disorders, for example [63, 64].

A second limitation is linked to the context of the study. The health system in Belgium suggested the research team to slightly modify the study protocol from how it was initially proposed by the developers of the programs. The manuals for the Mini-KIDS and Lidcombe Program recommend one-hour treatment sessions [29, 38], whereas only 30-minute speech-therapy sessions are currently covered by the Belgian healthcare reimbursement system for children under the age of 10. This leads to deviations regarding treatment approaches that have been proved efficient, for example the limitation to six one-hour treatment sessions in the Lidcombe Program and Mini-KIDS. Given that the TreatPaCS trial is conducted in the Belgian context, care should be taken when interpreting the results before applying them to other healthcare contexts.

The current trial does not include all existing treatment approaches for preschool age children who stutter. Future randomised controlled trials could extend the TreatPaCS trial by evaluating the efficacy of other treatments which are widely used in other countries and which are less frequently used in Belgium, such as the RESTART-DCM programme [65] or the Palin Parent-Child Interaction programme [66]. However, the results of this trial should make a major contribution to the treatment options for stuttering in young children by providing efficacy data for two additional treatment approaches. If the efficacy of these treatment approaches is shown, this will make it possible to offer PCWS new evidence-based treatment options according to their profile, values and preferences.

## Supporting information

S1 Protocol Version 2.2. This version is cleaned.
(PDF)

S1 File **a.** Ethics’ Study Approval in English/Dutch. **b.** Ethics’ Approval of Main Study Documents.
(ZIP)

S2 File **a.** Ethics’ Approval Study Extension (recruitment period) in Dutch. **b.** Ethics’ Approval Study Extension (recruitment period) in English.
(ZIP)

S3 File **a** Annual Progress Report Form. **b.** Ethics’ Notification of Approval Annual Progress Report Form in Dutch. **c.** Ethics’ Notification of Approval Annual Progress Report Form in English.
(ZIP)

S4 File **a.** KCE Review process. Review process applied to the protocol for funding. **b.** Agreement between KCE and Thomas More University of Applied Sciences.
(ZIP)

S1 Checklist SPIRIT checklist. (PDF)

S1 Fig Flow chart. (TIF)

S2 Fig Schedule of enrolment. (TIF)
