# Supplementary material for: Treatment for preschool age children who stutter: Protocol of a randomised, non-inferiority parallel group pragmatic trial with Mini-KIDS, social cognitive behaviour treatment and the Lidcombe Program—TreatPaCS
Source: PLoS One. 2024 Jul 11;19(7):e0304212. doi: 10.1371/journal.pone.0304212 (PMC11239023; doi:10.1371/journal.pone.0304212)

Thomas More University of Applied Sciences
University of Liège
Artevelde University of Applied Sciences
Clinical trial Centre University of Antwerp

Treatment for preschool age children who stutter: A randomised, non-inferiority parallel group pragmatic trial with Mini-KIDS, social cognitive behaviour treatment and the Lidcombe Program

TreatPaCS = Treatment for **P**reschool **a**ge **C**hildren who **S**tutter

Version 2.2_INV20-1257_TreatPaCS

Clinical trials.gov Number / Other registry Number:	NCT05185726
EDGE	002129
SPONSOR Number:	M-OHC-210048
KCE Trials Number:	INV20-1257

■ TABLE OF CONTENTS

■ **SIGNATURE PAGE** **5**
■ **STUDY PROTOCOL** **16**
1 **BACKGROUND** **16**
2 **RATIONALE** **19**
3 **ASSESSMENT AND MANAGEMENT OF RISK** **20**
4 **OBJECTIVES AND ENDPOINTS / OUTCOME MEASURES** **21**
4.1 PRIMARY OBJECTIVE 21
4.2 SECONDARY OBJECTIVES 21
4.3 ENDPOINTS 22
4.3.1 Primary endpoint 22
4.3.2 Secondary endpoints 23
4.3.3 Exploratory endpoints 23
4.3.4 Long-term follow-up (5 years post-randomisation) 24
5 **TRIAL DESIGN** **24**
6 **STUDY SETTING** **24**
7 **ELIGIBILITY CRITERIA** **25**
7.1 INCLUSION CRITERIA 25
7.2 EXCLUSION CRITERIA 25
8 **TRIAL PROCEDURES** **25**
8.1 RECRUITMENT 25
8.1.1 Patient identification 25
8.1.2 Screening 25
8.2 CONSENT 27
8.3 TRIAL RANDOMISATION 27
8.3.1 Method of implementing the allocation sequence 28
8.4 BLINDING 28
8.5 UNBLINDING 29
8.6 BASELINE DATA 29
8.7 TRIAL ASSESSMENTS 31
8.7.1 Trial assessment for the three arms during the baseline session 32
8.7.2 If randomised in the SCBT-arm 32
8.7.3 If randomised in the Mini-KIDS-arm 33
8.7.4 If randomised in the LP 33
8.8 TABLE OF TRIAL PROCEDURES 33
8.8.1 Screening: 33
8.8.2 Period of active monitoring (optional) 34
8.8.3 Baseline session 34
8.8.4 Each treatment session 34
8.8.5 3-months post-randomisation data collection point 35
8.8.6 6-months post-randomisation data collection point 36
8.8.7 9-months post-randomisation data collection point 37
8.8.8 12-months post-randomisation data collection point 38
8.8.9 18-months post-randomisation data collection point (primary data collection point) 39
8.8.10 2-years post-randomisation data collection point (Follow-up) 40
8.8.11 5-years post-randomisation data collection point (Follow-up) 40
8.9 FIDELITY OF IMPLEMENTATION 41

8.9.1 Parent treatment compliance ... 42
8.9.2 SLT treatment fidelity ... 42
8.9.3 Video analysis: Rating of %SS and SR ... 43
8.10 WITHDRAWAL CRITERIA ... 46
8.10.1 Discontinuation of trial intervention (without withdrawal of consent) ... 46
8.10.2 Discontinuation of study (with withdrawal of consent) ... 47
8.10.3 Loss to follow-up ... 47
8.11 END OF TRIAL ... 48
9 TRIAL INTERVENTION ... 48
9.1 TRIAL PROCEDURES IN THE SCBT ... 49
9.1.1 Conditioning of speaking activities ... 49
9.1.2 Cognitive training focused on emotions ... 50
9.1.3 Cognitive training focused on cognitions ... 51
9.1.4 Desensitisation ... 51
9.1.5 Skill training ... 52
9.1.6 Maintenance phase ... 52
9.2 TRIAL PROCEDURES IN MINI-KIDS ... 53
9.2.1 Mandate ... 53
9.2.2 Stage 1 = Desensitisation ... 53
9.2.3 Stage 2 = Identification ... 54
9.2.4 Stage 3 = Modification ... 55
9.2.5 Stage 4 = Generalisation ... 56
9.2.6 Maintenance phase ... 56
9.3 TRIAL PROCEDURES IN THE LP ... 56
9.3.1 Stage 1 ... 56
9.3.2 Stage 2 = Maintenance phase ... 57
9.4 ASSESSMENT OF COMPLIANCE ... 57
10 SAFETY RECORDING AND REPORTING ... 57
10.1 DEFINITIONS ... 58
10.2 RECORDING OF SAFETY FINDINGS IN FUNCTION OF THE AVAILABLE EVIDENCE ... 58
10.2.1 The case of types of intervention that are in routine clinical use ... 58
10.2.2 General considerations for the recording of safety findings ... 58
10.3 EXPEDITED REPORTING OF ADVERSE EVENTS ... 59
10.4 RESPONSIBILITIES ... 59
10.5 NOTIFICATION OF DEATHS ... 60
10.6 REPORTING URGENT SAFETY MEASURES ... 60
10.7 THE TYPE AND DURATION OF THE FOLLOW-UP OF SUBJECTS AFTER ADVERSE EVENTS ... 60
11 STATISTICS AND DATA ANALYSIS ... 60
11.1 SAMPLE SIZE CALCULATION ... 60
11.2 PLANNED RECRUITMENT RATE ... 61
11.3 STATISTICAL ANALYSIS PLAN ... 61
11.3.1 Summary of baseline data and flow of patients ... 61
11.3.2 Primary outcome analysis ... 62
11.3.3 Secondary outcome analysis ... 62
11.3.4 Procedure(s) to account for missing or spurious data ... 64
11.3.5 Other statistical considerations. ... 64
11.4 DATA COLLECTION FOR ECONOMIC EVALUATION ... 64
11.4.1 Burden ... 65
11.4.2 Potential effects on health ... 66

11.4.3 Potential effect on costs ... 66

12 DATA HANDLING ... 68

12.1 DATA COLLECTION TOOLS AND SOURCE DOCUMENT IDENTIFICATION ... 68

12.2 DATA HANDLING AND RECORD KEEPING ... 69

12.3 ACCESS TO DATA ... 69

12.4 ARCHIVING ... 70

13 MONITORING, AUDIT & INSPECTION ... 70

14 ETHICAL AND REGULATORY CONSIDERATIONS ... 71

14.1 ETHICS COMMITTEE (EC) REVIEW & REPORTS ... 71

14.2 PEER REVIEW ... 71

14.3 PUBLIC AND PATIENT INVOLVEMENT ... 72

14.3.1 Design of the research ... 72

14.3.2 Management of the research ... 72

14.3.3 Undertaking the research ... 73

14.3.4 Analysis of results ... 73

14.3.5 Dissemination of findings ... 73

14.4 REGULATORY COMPLIANCE ... 73

14.5 PROTOCOL COMPLIANCE ... 73

14.6 NOTIFICATION OF SERIOUS BREACHES TO GCP AND/OR THE PROTOCOL ... 74

14.7 DATA PROTECTION AND PATIENT CONFIDENTIALITY ... 74

14.8 FINANCIAL AND OTHER COMPETING INTERESTS FOR THE CHIEF INVESTIGATOR, SLTS AT EACH SITE AND COMMITTEE MEMBERS FOR THE OVERALL TRIAL MANAGEMENT ... 75

14.9 INDEMNITY ... 75

14.10 ACCESS TO THE STUDY DATA BY KCE AND SIMILAR INSTITUTES IN THE EU ... 76

14.11 ACCESS TO THE FINAL TRIAL DATASET BY OTHER PARTIES ... 77

14.11.1 Results Access Right ... 77

15 DISSEMINATION POLICY ... 77

15.1 DISSEMINATION POLICY ... 78

16 REFERENCES ... 79

■ APPENDICES ... 87

APPENDIX 1. RISK ASSESSMENT OF THE TRIAL INTERVENTION(S) ... 87

APPENDIX 2. PERSPECTIVES OF SLTS INVOLVED IN THE TREATPACS STUDY ... 88

APPENDIX 3. ANAMNESIS FORM ... 97

APPENDIX 4. KIDDYCAT ... 110

APPENDIX 5. QUESTIONS OF THE EQ-5D-Y-PROXY 1 ... 111

APPENDIX 6. CHILDREN'S BEHAVIOR QUESTIONNAIRE (CBQ) ... 112

APPENDIX 7. THE IMPACT OF STUTTERING ON PRESCHOOLERS AND PARENTS (ISPP) ... 113

APPENDIX 8. DATA COLLECTION POINTS: PARENT ... 114

APPENDIX 9. COMPLIANCE & TREATMENT FIDELITY CHECKLISTS ... 115

APPENDIX 10. AUTHORISATION OF PARTICIPATING SITES ... 137

APPENDIX 11. SAFETY REPORTING FLOW CHART ... 139

■ SIGNATURE PAGE

The undersigned confirm that the following protocol has been agreed and accepted and that the Chief Investigator (CI) agrees to conduct the trial in compliance with the approved protocol and will adhere to the principles outlined in the requirements for the conduct of clinical trials in the European Union (EU) as provided for in "Directive 2001/20/EC", and any subsequent amendments, Good Clinical Practice (GCP) guidelines, the Belgian law of May 7th 2004 regarding experiments on the human person, the Sponsor's Standard Operating Procedures, and other regulatory requirements as amended.

I agree to ensure that the confidential information contained in this document will not be used for any other purpose other than the evaluation or conduct of the clinical investigation without the prior written consent of Thomas More Mechelen-Antwerpen vzw.

I also confirm that I will make the findings of the study publicly available through publication or other dissemination tools without any unnecessary delay and that an honest accurate and transparent account of the study will be given; and that any discrepancies from the study as planned in this protocol will be explained.

For and on behalf of the Study Sponsor (Thomas More Mechelen-Antwerpen vzw):		
Signature:		Date: ....../....../......
Name (please print): Stijn Coenen		
Position: *Director of Thomas More University of Applied Sciences*		
**Chief Investigator:** Sabine Van Eerdenbrugh		
Signature:		Date: ....../....../......
Name: (please print): Sabine Van Eerdenbrugh		
Position: *Researcher and lecturer at Thomas More Mechelen-Antwerpen vzw*		
**Trial Statistician:** Ella Roelant		
Signature:		Date: ....../....../......
Name: (please print):		
Position:		
For acknowledgement on behalf of the funder (KCE):		
Signature:		Date: ....../....../......
Name (please print):		
Position:		

I

KEY TRIAL CONTACTS

Chief Investigator (CI)	Sabine Van Eerdenbrugh, Thomas More Mechelen-Antwerpen
Co-Chief Investigators (Co-CI)	Anne-Lise Leclercq, Université de Liège,<br>Veerle Waelkens, Artevelde Hogeschool,
Sponsor	Thomas More Mechelen-Antwerpen vzw
Funder(s)	Belgian Health Care Knowledge Centre (KCE)
Clinical Trials Unit	Clinical Trials Center UZ-Antwerpen (CTC UZ-Antwerpen)
Trial Statistician	Ella Roelant<br>Universitair Ziekenhuis Antwerpen (UZ-ANTWERPEN)
Data Manager	Kim Claes<br>Universitair Ziekenhuis Antwerpen (UZ-ANTWERPEN)<br>Elyne Scheurwegs<br>Universitair Ziekenhuis Antwerpen (UZ-ANTWERPEN)
General support Clinical Trials Center	Iris Verhaegen<br>Universitair Ziekenhuis Antwerpen (UZ-ANTWERPEN)<br>Elke Smits<br>Universitair Ziekenhuis Antwerpen (UZ-ANTWERPEN)
Project Manager (PM)	Sabine Van Eerdenbrugh
Blinded Video Analysts (%SS & SR)	Kurt Eggers, Coordinator Video Analyses<br>Thomas More Mechelen-Antwerpen<br>Astrid Moyse<br>Veerle Waelkens<br>Steffi Snijders<br>Thomas More Mechelen-Antwerpen<br>Estelle Dauvister<br>Université de Liège
Unblinded Video Analyst (Treatment fidelity)	Sabine Van Eerdenbrugh<br>Anne-Lise Leclercq
Research Support Sponsor	Kris Vancluysen<br>Thomas More Mechelen-Antwerpen<br>Tim Vanhoomissen<br>Thomas More Mechelen-Antwerpen

Field Monitors	Dafne Balemans<br>Universitair Ziekenhuis Antwerpen (UZ-ANTWERPEN)<br>Lynsey Verhoeven<br>Universitair Ziekenhuis Antwerpen (UZ-ANTWERPEN)<br>David Hoogstad<br>Universitair Ziekenhuis Antwerpen (UZ-ANTWERPEN)
Administrative Support & Randomisation	Sofie Grenier and Debbie Smet<br>Universitair Ziekenhuis Antwerpen (UZ-ANTWERPEN)
Committees	**Trial Management Group**<br>Sabine Van Eerdenbrugh, Chief Investigator<br>Anne-Lise Leclercq, Co-Chief Investigator FR<br>Veerle Waelkens, Co-Chief Investigator FL<br>Ella Roelant, Trial Statistician<br>Kurt Eggers, Video Analysis Coordinator<br>Kim Claes, Data Manager<br>Iris Verhaegen or Elke Smits, Clinical Trial Center UZ-ANTWERPEN<br><br>**Trial Steering Committee**<br>Sabine Van Eerdenbrugh, Chief Investigator<br>Anne-Lise Leclercq, Co-Chief Investigator FR<br>Veerle Waelkens, Co-Chief Investigator FL<br>Ella Roelant, Trial Statistician<br>Kurt Eggers, Video Analysis Coordinator<br>Marie-Christine Franken, Independent Expert<br>Iris Verhaegen or Elke Smits, Clinical Trial Center UZ-Antwerpen<br>Dafne Balemans, Lynsey Verhoeven or David Hoogstad, Field Monitors<br>Betrand De Wolf, Patient Representative<br>Sarah Provin, Patient Representative<br>Annelies Vanhaesebrouck, Patient Representative<br>Liesbeth Hoste, Patient Representative<br>Charlotte Roggeman, Patient Representative<br>Fien Maes, Patient Representative<br>Kris Vancluysen, Representative of the sponsor<br>Tim Vanhoomissen, Representative of the sponsor<br>Representative of the Funder<br><br>**SLT-RCT committee-FR**<br>Anne-Lise Leclercq, Co-Chief Investigator FR<br>Sabine Van Eerdenbrugh, Chief investigator

	+ Participating French-speaking SLT-Sites<br><br>**SLT-RCT committee-FL**<br>Veerle Waelkens, Co-Chief Investigator FL<br>Sabine Van Eerdenbrugh, Chief investigator<br>+ Participating Dutch-speaking SLT-Sites

TRIAL SUMMARY

Trial Title	Treatment for preschool age children who stutter: a randomised, non-inferiority parallel group pragmatic trial with Mini-KIDS, Social-Cognitive Behaviour Treatment (SCBT) and the Lidcombe Program (LP)
Short title	Treatment for Preschool age Children who Stutter
Internal reference	TreatPaCS
Trial Design	A three arm 1:1:1 randomised, open-label non-inferiority parallel group pragmatic trial
Trial Participants and setting	249 preschool age children who stutter between 2 and 6.5 years
Intervention(s)	Mini-KIDS is a direct treatment based on principles of stuttering modification, with pseudo-stuttering, that is, deliberate stuttering, as one of the main components. The program for 4-6-year old children consists of four stages: Stage 1 = desensitization, Stage 2 = identification, Stage 3 = modification and Stage 4 = generalization. The program for 2-4-year old children does not include stage 3 because of metacognitive development at that age. Speech-Language Therapist (SLT) and parent(s) are the speech model for the child. They add normal disfluencies and pseudo-stuttering to their speech. Later on in treatment and if necessary, children learn to recognise and alter their stuttering moments.<br>SCBT contains 5 treatment phases: (1) conditioning speaking activities, (2) cognitive training focused on emotions, (3) cognitive training focused on cognitions, (4) desensitisation (emotional training) and (5) skill training. This treatment is mainly directed at the child's cognitive and emotional aspects that surround the stuttering.
Control	The LP is an operant program that directly provides verbal feedback to the child's stutter-free speech (mainly) and the child's stuttering (occasionally). The program comprises two stages: Stage 1 in which (near) zero levels of stuttering are achieved and Stage 2 in which the achieved (near) zero levels of stuttering are maintained for a long period of time.
Primary Endpoint	Percentage Syllables Stuttered (%SS, measured in video recorded speech samples) at 18-months post-randomisation.
Secondary Endpoint(s)	%SS and Severity Ratings (SR) at 3-, 6-, 9-, 12- and 18-months post-randomisation, QOL (EQ-5D-Y proxy 1) and Impact of Stuttering on Preschoolers and Parents (ISPP, Langevin et al., 2010) at 3-, 9-, and 18-months post-randomisation + KiddyCAT scores (Communication Attitude Test, Vanryckeghem & Brutten, 2015) at 18-months post-randomisation.<br>%SS, SR and QOL (EQ-5D-Y proxy 1) at 2-and 5-years post-randomisation.

	Proportion of children with < 1%SS and ≤1SR at 18 months post-randomisation. Separate analyses of %SS and SR for videos recorded at home and during the treatment sessions at 18 months post-randomisation.<br>Treatment hours, weeks and sessions until end of treatment (start maintenance phase).<br>Parent report about stuttering severity (based on Onslow et al., 2021) and satisfaction with everyday communication (based on Karimi et al., 2018) at 3-, 9-, and 18-months, 2 and 5 years post-randomisation.
Planned Sample Size	N = 249
Treatment duration	Varies between 3 months and 2 years of treatment. Consists of weekly or twice weekly treatment sessions until treatment gains have been achieved and followed by a maintenance phase of about 1 year (with 7 treatment sessions).
Follow up duration	Follow-up on treatment results 2-year post-randomisation.<br>The 5-year post-randomisation data collection point falls beyond the duration of this trial and will be carried out independently as we feel it important to have an extended long-term follow up.
Duration of the trial (FPI-CSR)	4 years

FUNDING AND SUPPORT IN KIND

FUNDER

BELGIAN HEALTH CARE KNOWLEDGE CENTRE, Administrative Centre Botanique (Doorbuilding)

Boulevard du Jardin Botanique 55, B-1000 Brussels, Belgium.

ROLE OF STUDY SPONSOR AND FUNDER

Thomas More Mechelen-Antwerpen vzw shall act as sponsor of the Study, as defined in the Law of 2004, and shall assume all responsibilities and liabilities in connection therewith and procure the mandatory liability insurance coverage in accordance with the Law of 2004. Thomas More Mechelen-Antwerpen vzw shall ensure that it shall be mentioned in the Protocol, the Informed Consent Forms and in other relevant communication with the Study Subjects or the Regulatory Authorities as sponsor of the Study. Thomas More Mechelen-Antwerpen vzw acknowledges and agrees for the avoidance of doubt that KCE shall under no circumstances be considered as sponsor of the Study or assume any responsibilities or liabilities in connection therewith, and Thomas More Mechelen-Antwerpen vzw shall make no representations whatsoever in this respect.

KCE provides the funding for the study and keeps an overview on the conduct of the study based on regular reports from the sponsor. KCE will perform health economic analyses with some of the study data.

ROLES AND RESPONSIBILITIES OF TRIAL MANAGEMENT COMMITEES

Trial Steering Committee

The role of the Trial Steering Committee is to provide the overall supervision of the trial. The Trial Steering Committee includes members who are independent of the participating sites (SLTs), their employing organisations (if applicable), funder and sponsor. The Trial Steering Committee monitors trial progress, conducts and advises on scientific credibility. The Trial Steering Committee will consider and act, as appropriate, and ultimately carries the responsibility for deciding whether a trial needs to be stopped on grounds of safety or efficacy.

The Trial Steering Committee will meet on average three times per year the first year and twice a year after that. The Trial Steering Committee in TreatPaCS is composed of the CI, the two Co-Cis, the Trial Statistician, the trial PM, the Independent Expert, a representative of CTC UZ-Antwerpen, a representative of the participating SLTs, two patients or members of the public, a representative of the sponsor and a representative of the funder.

The day-to-day management of the study will be performed by the Trial Management Group which is distinct from the Trial Steering Committee.

Trial Management Group

The Trial Management Group meets regularly and are responsible for the daily management of the study. During their meetings, they discuss overall progression of the study, practical hurdles that are reported by participating SLTs, questions coming from the SLTs, database issues and all other minor practical issues that demand quick resolution. The Trial Management Group is composed of CI, Co-Cis, PM, Trial Statistician, Data Manager, CTC UZ-Antwerpen support and when relevant, other members with specific interests such as the Field Monitors or the Video Analysts. If important or relevant items for the SLTs were discussed, the respective SLT or – if relevant – all SLTs will receive this information through e-mail. Minutes from every meeting as well as communication with the SLT(s) will be available in the Trial Master File.

Dutch-speaking SLT-RCT committee

The SLT-RCT committee consists of the Dutch-speaking Co-CI, the Flemish SLTs who participate in the trial and CI. Meetings are organised every 4 months during the first 2.5 years of the treatment period. After these 2.5 years, ad hoc meetings will be organised, depending on the need of the SLTs. The focus of the meetings is on the clinical aspects such as problem-solving when progress is not further achieved or when the treatment needs individual tailoring while adhering to the protocol, ...

French-speaking SLT-RCT committee

The SLT-RCT committee consists of the French-speaking Co-CI, the SLTs from Brussels and Wallonia who participate in the trial and the CI. Meetings are organised every 4 months during the first 2.5 years of the treatment period. After these 2.5 years, ad hoc meetings will be organised, depending on the need of the SLTs. The focus of the meetings is on the clinical aspects such as problem-solving when progress is not further achieved or when the treatment needs individual tailoring while adhering to the protocol, ...

LIST OF ABBREVIATIONS

ABBREVIATION	
%SS	Percentage Stuttered Syllables
CI	Chief Investigator
Co-CI	Co-Chief Investigator
CRF	Case Report Form
EC	Ethics Committee
e-CRF	Electronic CRF
EU	European Union
EudraCT	European Clinical Trials Database
GCP	Good Clinical Practice
ICH	International Conference on Harmonisation of technical requirements for registration of pharmaceuticals for human use.
KCE	Belgian Healthcare Knowledge Centre
LP	Lidcombe Program
PCWS	Preschool Age Child who Stutters
RCT	Randomised Control Trial
SCBT	Social Cognitive Behaviour Treatment
SLT	Speech-Language Therapist
SR	Severity Rating
SSI-4	Stuttering Severity Instrument-4
TSB-NL	Test de Sévérité du Bégaiement -Non Lecteurs
TVS-NL	Test Voor Stotterernst-Niet Lezers

DEFINITION	
PCWS' speech-language therapy file	The file in which the SLT notes down all relevant information about the PCWS
REDCap	System used to build the e-CRF in which the data are collected that SLTs and parents load
SLT site file	The file containing the treatment guides (for the LP, Mini-KIDS and SCBT), compliance checklist, treatment fidelity checklist, information about REDCap, insurance, ...
Trial Master File	The file in which all relevant documents about TreatPaCS are collected (e.g., compliance manual, treatment fidelity manual, safety reporting scheme, video analysis manual, statistical plan, trial monitoring plan, insurance, meeting minutes, ... This file is stored at Thomas More Mechelen-Antwerpen

TRIAL FLOW CHART

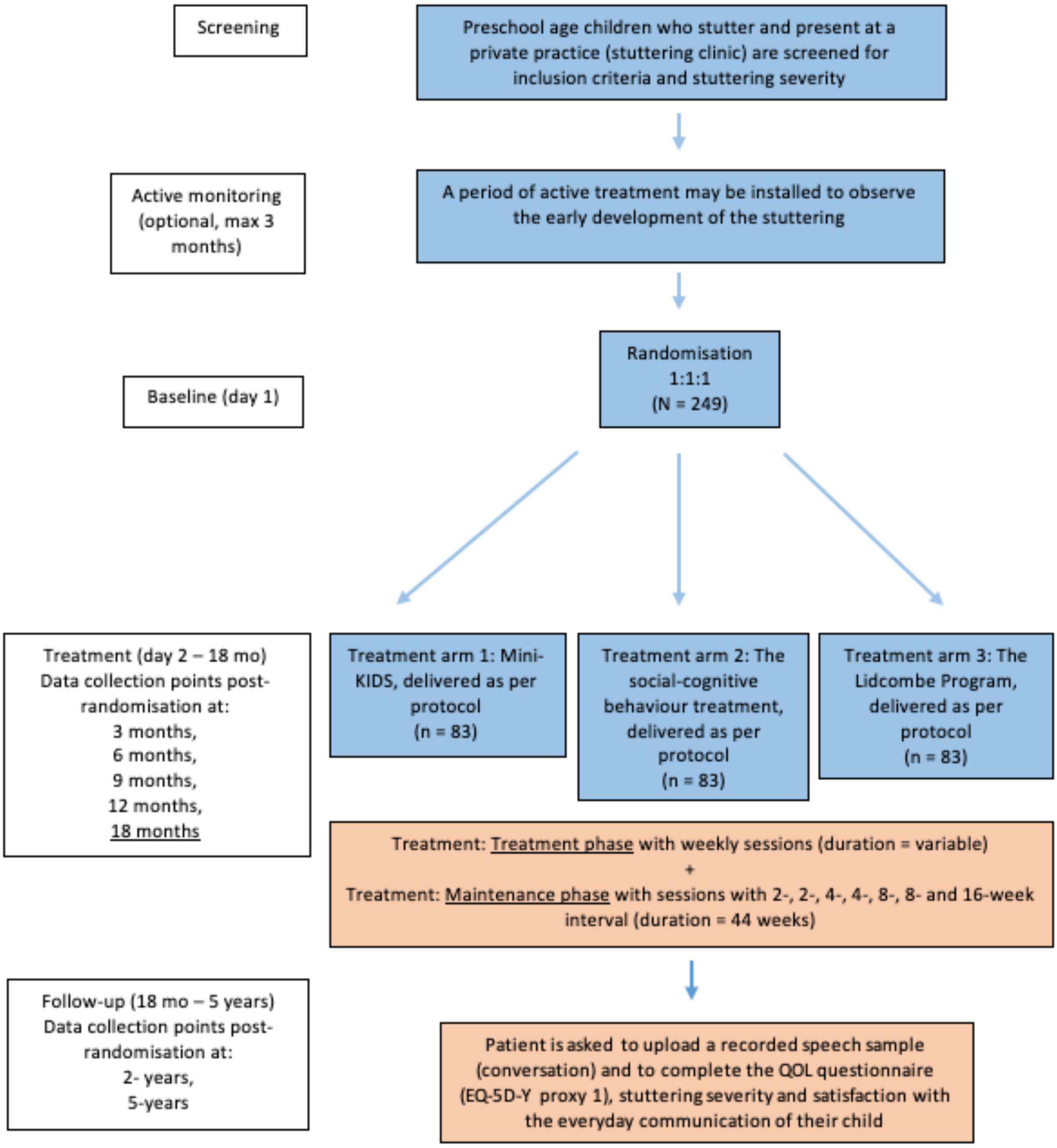

Figure 1. Flow chart of TreatPaCS

■ STUDY PROTOCOL

1 BACKGROUND

The World Health Organisation (2021) characterises developmental speech fluency disorder: "by frequent or pervasive disruption of the normal rhythmic flow and rate of speech characterised by repetitions and prolongations in sounds, syllables, words, and phrases, as well as blocking and word avoidance or substitutions. The speech dysfluency is persistent over time. The onset of speech dysfluency occurs during the developmental period and speech fluency is markedly below what would be expected for age. Speech dysfluency results in significant impairment in social communication, personal, family, social, educational, occupational or other important areas of functioning. The speech dysfluency is not better accounted for by a Disorder of Intellectual Development, a Disease of the Nervous System, a sensory impairment, or a structural abnormality, or other speech or voice disorder " (ICD-11, 6A01.1). Stuttering at preschool age is very different from stuttering in older children, adolescents and adults. A distinction between *early childhood stuttering* and *persistent stuttering* is therefore useful.

Developmental stuttering (as opposed to acquired stuttering) typically appears in children between two and five years of age (Guitar, 2018; Yairi et al., 1996), with about 95% before the age of 4 years (Yairi & Ambrose, 2013). The cumulative incidence of stuttering in preschool age children is reported between 8% (Yairi & Ambrose, 2013) and 11.2% by the age of 4 years (Reilly et al., 2013).

About 74% of the children recover from (early childhood) stuttering within the first 4 years after stuttering onset (Yairi & Ambrose, 1999). Waiting for spontaneous recovery to occur, however, is not current practice as evidence suggests that (1) stuttering treatment is most effective at preschool age, (2) persisting stuttering (after the age of 6) is not only more difficult to treat successfully, but also increases the risk of developing mental health problems, such as (social) anxiety disorders and (3) it is not possible to predict in which children stuttering will persist or not (e.g. Oonk et al., 2020).

An additional reason not to wait for spontaneous recovery to occur, is the finding that only a small proportion of the children who starts to stutter recovers within the first year after stuttering onset. More specifically, five (of 84 = 6%) recovered in 12 to 17 months after onset without treatment or with minimal stuttering management advice in a study of Yairi and Ambrose (1999). Also, preschool children become increasingly more aware about their stuttering when they get older (e. g. Langevin et al., 2010; Van Ryckeghem et al., 2005).

In Belgium, children who stutter are typically consulting a stuttering therapist in a private setting. In Belgium, 5066 speech-language therapists (SLTs) actively work in a private setting (PlanKad Logopedisten, 2016, p. 23). Stuttering therapists are SLTs that have specialised in stuttering as the basic speech-language therapy course does not provide sufficient background to treat this complex disorder. The number of Belgian SLTs who are specialised in stuttering, is unknown. The severity of the disorder and the number of

preschool age children that are treated for stuttering, is also unknown. The incidence of 8% to 11.2% however, gives an indication.

The Belgian health system provides 10 sessions for parents (without child) individually or in a group (Riziv, 2018). For the patient, 128 sessions of 30 minutes (minus the sessions with the parents) are reimbursed in a period of maximum 2 years. Children younger than 10 years are restricted to treatment sessions of 30 minutes. Between 6 months and 2 years after finalising treatment, maximum 38 treatment sessions to treat relapse are reimbursed in a period of one year if they were not yet used in the treatment period (pack of 128 treatment sessions).

Belgian stuttering therapists tend to use different approaches to treat stuttering (e. g. Eggers & Van Eerdenbrugh, 2019; Van Eerdenbrugh et al., 2020). Differences between Dutch-speaking and French-speaking therapists are obvious. While Dutch-speaking therapists focus on socio-emotional aspects of the stuttering, French-speaking therapists focus more often on the speech-related aspects of the stuttering. This, however, is a concise conclusion from the research study published by the authors above and does not take the age of the patient into account. That is, treatment of early childhood stuttering and treatment of persistent stuttering were not distinguished in that study.

It is unclear what type of treatment stuttering specialists deliver most frequently to preschool age children who stutter (PCWS) in Belgium. However, from recent studies (Eggers & Van Eerdenbrugh, 2019; Van Eerdenbrugh et al., 2020; Van Eerdenbrugh et al., currently in review), it became clear that stuttering specialists in Belgium regularly deliver the Lidcombe Program (LP), Mini-KIDS and social-cognitive behaviour treatment (SCBT) to PCWS. The LP and Mini-KIDS are both treatment programs for PCWS, directed at the speech of the child. A reduction of stuttering is the main aim of the direct treatment programs. Just as in other treatment programs for PCWS, the parents are intensively involved and are psycho-educated about stuttering. These direct treatment programs give attention to social and cognitive aspects but do not focus primarily on them in treatment; the primary focus of direct treatment programs is on the motor aspects of the speech. In SCBT, the primary focus is on conditioning and cognitive training of emotions and cognitions, desensitisation (emotional training) and skill training of the PCWS and parents about stuttering and speaking.

The LP (Onslow et al., 2021) is an operant program that directly provides verbal feedback to the child's stutter-free speech (mainly) and the child's stuttering (occasionally). Parents implement the treatment at home, in the daily environment of the child. The LP comprises two stages: Stage 1 in which (near) zero levels of stuttering are achieved and Stage 2 in which the achieved (near) zero levels of stuttering are maintained for a long period of time. The LP usually takes between 11 to 23 (45 to 60-minute) treatment sessions to achieve the goals of Stage 1, i. e. (near) zero levels of stuttering. The LP is recommended as the treatment option with the strongest evidence in (systematic) reviews to date (Baxter et al., 2015; Bothe et

al., 2006; Brignell et al., 2021; Nye & Hahs-Vaughn, 2011; Nye et al., 2013; Seth & Maruthy, 2018) and the Dutch clinical guideline (Oonk et al., 2020).

Treatment with the LP does not seem to impact on the quality of the attachment between children and the parents who implement the treatment (Woods, 2002). It also does not impact negatively on the speech and language of the parents nor the children (Bonelli et al., 2000; Onslow et al., 2002); on the contrary, children seem to increase their linguistic complexity over the course of the treatment (Lattermann et al., 2005).

A meta-analysis of two clinical trials (Jones et al., 2005; Lewis et al., 2008) and two clinical experiments (Lattermann et al., 2005;Harris et al., 2002) conducted with the LP (Onslow et al., 2012) involved 134 children. The children in the control groups received the LP after 9 months of no treatment; it would be unethical to deny them of any treatment. At pre-randomisation the stuttering severity of the group who received the LP and the control group were about the same. There was a small average improvement in the control group over time due to spontaneous recovery, which was predictable (Onslow, 2021). However, at a mean post-randomisation period of 6.3 months, the group who received the LP did significantly better than the control group. The LP odds ratio at that point in time was 7.5 for achieving %SS of below 1.0. So, the children who received the LP had 7.5 times greater odds of having “no stuttering” or “almost no stuttering” than children who did not receive the LP.

SCBT contains five treatment phases: (1) conditioning speaking activities, (2) cognitive training focused on emotions, (3) cognitive training focused on cognitions, (4) desensitisation (emotional training) and (5) skill training (Boey, 2010). This treatment is not directed at the speech of the children, but rather at the cognitive and emotional aspects that surround the stuttering. Boey (2008) published a study on the long-term effect of SCBT in different age groups as part of his dissertation.

Mini-KIDS is a direct treatment based on principles of stuttering modification through pseudo-stuttering, that is, deliberate stuttering, as one of the main components. The program for 4-6-year old children consists of four stages: Stage 1 = desensitization, Stage 2 = identification, Stage 3 = modification and Stage 4 = generalization. The program for 2-4-year old children does not include stage 2. SLTs and parent(s) are the speech model for the child. They add normal dysfluencies to their speech. Later on in treatment and if necessary, children learn to recognise and alter their stuttering moments.

This approach is based on the therapy concept KIDS (‘Kindern Dürfen Stottern’, Sandrieser & Schneider, 2015) for school-aged children and is based on a stuttering modification approach for adults. The main components of Mini-KIDS are supported by evidence. Evidence to support the necessary metacognitive and metalinguistic skills to work directly on desensitization, identification, and modification of stuttering like disfluencies in this young age group by means of pseudo-stuttering is found in several publications, e. g. Arias and Diaz (2010) and Hakim and Bernstein Ratner (2004). Evidence to argue for desensitisation in

treatment for PCWS can be found in Johnson et al. (2010), Karrass et al. (2006) and Prins et al. (2011). For direct work with the modification of speech motor loss of control in young children, evidence is found in research on brain function and motor speech development (e.g., Bohland et al. 2010; Galantucci et al. 2006; Guenther, 2006; Olander et al. 2010). Mini-KIDS as entire program, however, is not yet supported by research-based evidence, only practice-based evidence.

Parents are intensively involved in the three treatment programs.

2 RATIONALE

Stuttering can have a significant impact on children's social and emotional development (McAllister, 2016). PCWS can show signs of social discomfort (Boey et al., 2009; Langevin et al., 2009). At school age when the stuttering has become persistent, children who stutter can be considered as less popular by peers, have an increased risk to be bullied, can experience more fear and can be more worried than peers who do not stutter (Blood & Blood, 2007; Davis et al., 2002). Teenagers and adolescents who stutter report having difficulty communicating to peers and to belong to a group; some of them develop a low self-esteem (Blood & Blood, 2004). Adults who stutter have a seven-fold increased risk to develop a social anxiety problem (Iverach et al., 2009) and do not seem to have the same opportunities in their professional life compared to adults who do not stutter (Gerlach et al., 2018). Also, treatment for early childhood stuttering (before the age of 6 years, so at preschool age) indicates to achieve the best possible results compared to stuttering treatment at older ages (e. g. Bothe et al., 2006). So, it is clear that timely intervention, that is at preschool age, is necessary to reduce and avoid negative social implications and the development of social anxiety disorders, and to achieve the best possible treatment outcome for stuttering.

It is difficult, however, to predict which preschool age child will recover from early childhood stuttering without treatment (= spontaneous recovery) and which child won't. Therefore, a period of (active) monitoring is often the first step before initiating treatment but needs to be limited in time to initiate treatment in a timely manner, i.e., before the age of 6 (Packman et al., 2003).

Evidence for the effectiveness of treating stuttering in preschool age children with direct, operant interventions is growing (Blomgren, 2013; Bothe et al., 2006; Nye et al., 2013). Stuttering interventions directed at the speech of PCWS are introduced more frequently nowadays as option for the treatment of stuttering in preschool age children in Europe (e. g., de Sonneville-Koedoot et al., 2015; Kingston et al., 2003; Lattermann et al., 2008; Oonk et al., 2020).

Given the fact that in Belgium, SLTs deliver three treatment approaches for stuttering in PCWS, we believe it is necessary to assess the treatment outcome of those three treatments as it is vital to try to achieve the

best possible outcome for PCWS in the daily clinical practice before the age of 6 years, so within the preschool age years (also called ‘the window of opportunity’).

We estimate, based on findings from the literature, that the three treatment programs will achieve similar outcome at 18 months post-randomisation. That is, (near) zero levels of stuttering and linked to this result, a high score on Quality-Of-Life scores (QOL), a positive attitude towards communication and no or low impact of stuttering on the preschool child and his parents. Currently, these three stuttering treatment approaches for preschool age children are delivered daily to Belgian preschool age children who stutter. Their effect compared to each other, however, is not known. We believe, as it is our duty to aim for as much recovery of early childhood stuttering as possible before stuttering becomes persistent, that the current trial is necessary to support current practice in Belgium. Also, it is essential to provide evidence for the treatments to help SLTs choose the most appropriate treatment approach for a family.

As a secondary objective, we expect a significant difference in treatment time necessary to reach the treatment goals. We believe that the direct treatment programs (the LP and Mini-KIDS) need less treatment time (measured in hours) than SCBT with a difference of about 1/3 to nearly 1/2 of the treatment time. This is an estimation based on available publications (Arnott et al., 2014; Bridgman et al., 2016; De Sonneville-Koedoot et al., 2015; Donaghy et al., 2020). We believe that this is an important objective with implications on both the families and the Belgian reimbursement system. To shorten the treatment time implies a reduction of parents’ burden of providing time and travelling to the stuttering practice and the costs for parents and the National Institute for Health and Disability Insurance.

3 ASSESSMENT AND MANAGEMENT OF RISK

TreatPaCS is categorised as a low intervention clinical trial and provides justification in Appendix 1.

The three treatment approaches are standard of care treatment approaches in use in Belgium in this population. One treatment approach, the LP, is evidence-based and supported by published scientific evidence on the safety and efficacy of the treatment. The two others, SCBT and Mini-KIDS, are supported by evidence for individual techniques but the treatment approaches as a whole are not evidence-based. They are, however, practice-based as they are currently being delivered frequently to PCWS in standard care. The additional diagnostic or monitoring procedures do not pose more than minimal additional risk or burden to the safety of the subjects compared to normal clinical practice.

A risk management plan is developed for this study.

4 OBJECTIVES AND ENDPOINTS / OUTCOME MEASURES

4.1 Primary objective

The primary objective is to compare the % Syllables Stuttered (%SS, a measurement for the frequency of stuttering measured on video recordings by blinded video analysts) between the speech samples of PCWS treated with Mini-KIDS, SCBT and the LP at 18 months post-randomisation.

4.2 Secondary objectives

The secondary objectives are

- To compare the %SS, measured on video recordings by blinded video analysts, between PWCS treated with Mini-KIDS, SCBT and the LP at 3, 6, 9 and 12 months post-randomisation
- To compare the Severity Ratings (SR, a subjective measure for frequency and severity, measured on video recordings by blinded video analysts) between PCWS treated with Mini-KIDS, SCBT and the LP at 3, 6, 9, 12 and 18 months post-randomisation
- To compare the Quality of Life (QOL, measured with the EQ-5D-Y proxy 1) and the impact of stuttering on PCWS and parents (measured with the ISPP-questionnaire) between PCWS treated with Mini-KIDS, SCBT and the LP at 3, 9 and 18 months post-randomisation
- To compare parent report about stuttering severity and satisfaction with everyday communication between PCWS treated with Mini-KIDS, SCBT and the LP at 3, 6, 9, 12 and 18 months, 2 and 5 years post-randomisation
- To compare the communication attitude (measured with the KiddyCAT) between PCWS treated with Mini-KIDS, SCBT and the LP at 18 months post-randomisation
- To compare the %SS and SR (measured on video recordings by blinded video analysts) and QOL (EQ-5D-Y proxy 1) between PCWS treated with Mini-KIDS, SCBT and the LP at 2 and 5 years post-randomisation
- To compare the treatment time (in hours), number of weeks and number of treatment sessions until the start the maintenance phase of each treatment
- To look at the proportion of children that are successful (defined as <1 %SS and SR ≤ 1) at 18 months and at 5 years post-randomisation.
- To evaluate %SS and SR at 18 months post-randomisation for the videos at home.
- To evaluate %SS and SR at 18 months post-randomisation for the videos recorded during the treatment sessions.

4.3 Endpoints

4.3.1 Primary endpoint

The primary endpoint is %SS at 18 months post-randomisation. %SS is measured by the blinded video analysts on video recorded speech samples from home and from the treatment session and will be computed as the average of the two speech samples.

For stuttering, no core set of measurements exist. %SS was chosen as it is the most frequently used objective measure in clinical trials that compares stuttering treatment. It is used in universally known stuttering severity instruments (e.g., SSI-4). A similar measure on word level is used in locally administered stuttering severity instruments (e.g., TVS-NL, TSB-NL).

The taxonomy of Teesson et al. (2003, p. 1008) can be used to categorise the stuttered syllables:

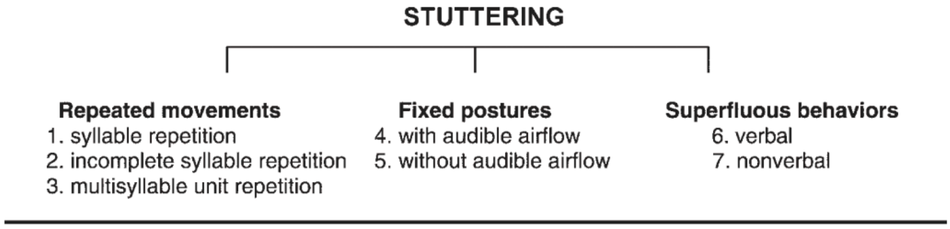

Figure 2. Taxonomy of Teeson et al. (2003) to classify stuttered syllables

Fixed postures with audible airflow are often referred to as prolongations; fixed postures without audible airflow are often referred to as blocks.

The 18-months post-randomisation time point was chosen to enable all participants to complete the treatment phase and achieve the goals of each treatment approach. The median number of treatment sessions, calculated in 30 minutes, are expected to differ. For the LP, it is expected to be 47, for Mini-KIDS 61 and for SCBT 94 (for SCBT treatment sessions are scheduled two times a week). These numbers of treatment sessions include the screening session, the parent coaching session(s) or parent training, the treatment phase and the maintenance phase, which is scheduled after the treatment goals were achieved to monitor the stuttering on the long term as stuttering is known to be a relapse-prone disorder.

A similar RCT conducted in the Netherlands in which the LP was compared to Restart-DCM, used the same primary endpoint (De Sonneville-Koedoot et al., 2015). Restart-DCM is an indirect treatment program for PCWS that is delivered frequently in the Netherlands. More RCTs with the LP used 18 months post-randomisation as data collection point: Donaghy et al. (2020) who compared the LP with and without verbal

contingencies, Bridgman et al. (2016) who compared the LP delivered in the clinical practice with the LP delivered via webcam, and Arnott et al. (2014) who compared the LP with the LP in group format.

4.3.2 Secondary endpoints

%SS (measured on video recordings by blinded video analysts) is also measured at 3, 6, 9, 12 months and 2 and 5 years post-randomisation.

SR (measured on video recordings by blinded video analysts) is measured at 3, 6, 9, 12, 18 months and 2 and 5 years post-randomisation. Whereas %SS measures the frequency of stuttering, SR measures frequency and severity. For example, a sentence spoken with one fixed posture with audible airflow (prolongation) combined with a non-verbal superfluous behaviour (e.g., grimace) will result in a higher SR than three monosyllabic repetitions.

%SS and SR (measured on video recordings by blinded video analysts) is measured at 18 months post-randomisation for the videos at home and for the videos recorded during the treatment sessions.
The proportion of children that are successful (defined as <1 %SS and SR ≤ 1) is evaluated at 18 months and at 5 years post-randomisation.

%SS and SR are determined by blinded video analysts on the videos of the speech samples. Procedures are explained in section 8.8.

The impact of stuttering on PCWS and parents (ISPP) is measured at 3, 9 and 18 months post-randomisation. The quality of life (EQ-5D-Y proxy 1) is measured at 3, 9 and 18 months and 2 and 5 years post-randomisation. Communication attitude of the child (KiddyCAT) is measured at 18 months post-randomisation. These three measures assess the covert aspects of stuttering, as opposed to the overt aspects (measured by %SS and SR).

Parents are asked to report about their child's stuttering severity and their satisfaction with their child's everyday communication at 3, 6, 9, 12 and 18 months, 2 and 5 years post-randomisation.

The number of treatment time (in hours), number of weeks and number of treatment sessions are counted until the start the maintenance phase of each treatment approach.

4.3.3 Exploratory endpoints

We will explore if we can find a profile of children more successful to one of the treatment approaches. Possible attributes that will be studied are attributes established at baseline such as gender, co-morbidities, age at assessment, family history of stuttering, family history of recovery of stuttering, onset data (gradual/sudden, time since onset, progress since onset), answers to the ISPP-questionnaire, stuttering

severity (%SS and SR), parent-related profile questions and temperament will be included in a multiple regression model (linear and logistic will be considered).

A survey to capture the perspectives of the participating SLTs about delivering the three treatments will be sent to the SLTs before and after the study (Appendix 2). This qualitative analysis will be carried out by the research team.

4.3.4 Long-term follow-up (5 years post-randomisation)

The trial ends at 2 years post-randomisation. Additional data collection, however, is scheduled at 5 years post-randomisation. This data collection will be organised by the CI and co-CIs to collect long-term data of the PCWS and their parents and is not funded by KCE. The parents of the PCWS will be asked to upload a video of their child's speech and will be asked to report about the severity of the stuttering and the satisfaction of their child's speech in everyday speaking situations, and about the child's the QOL (EQ-5D-Y proxy 1).

5 TRIAL DESIGN

This study is a three arm, 1:1:1 randomised, open-label pragmatic comparative trial comparing Mini-KIDS, SCBT and the LP.
To show non-inferiority of Mini-KIDS compared to LP and SCBT compared to LP a parallel group design will be used. Stratified randomisation will be used according to site (30 sites) and gender.

6 STUDY SETTING

TreatPaCS is a trial with 30 sites (SLTs). Eligibility criteria for the sites and participating SLTs are:

- The site is a private practice specialised in stuttering (does not need to be exclusively in stuttering)
- The participating SLT is experienced in treating PCWS with at least 2 years of experience
- The participating SLT is prepared to deliver the three treatment approaches
- The participating SLT attends (again) the three workshops (Mini-KIDS, SCBT and LP) and the International Conference on Harmonisation of technical requirements for registration of pharmaceuticals for human use- Good Clinical Practice (ICH-GCP) workshop
- The participating SLT starts stuttering treatment with at least 1 PCWS per three months or more (based on a retrospective survey conducted during the feasibility phase of the study)

7 ELIGIBILITY CRITERIA

7.1 Inclusion criteria

TreatPaCS includes preschool age children

- who stutter (identified with the Stuttering Severity Instrument-4 (SSI-4) or the Test For Stuttering Severity-Non Readers, available in Dutch and French (TVS-NL/TSB-NL)
- who are aged between 2 - 6.5 years
- who have no hearing loss, reported by the parent(s)
- who have, if bilingual, a parent who speaks a language that the SLT understands and speaks to allow clear communication
- who have at least one parent agreeing to be intensively involved in treatment and knowing that s/he will implement the treatment at home
- who have at least one parent who is willing and able to video record his/her child regularly

A PCWS can participate in the trial if the Informed Consent Form is signed by one of the parents.

7.2 Exclusion criteria

TreatPaCS will not include preschool age children with a syndrome such as Down Syndrome.
Note: PCWS with comorbidities (Autism Spectrum Disorder, ADHD, language disorders, ...) are not excluded from the trial as the goal is to resemble standard of care.

8 TRIAL PROCEDURES

8.1 Recruitment

8.1.1 Patient identification

The participating SLT will recruit the PCWS when the parent contacts the SLT's private practice. The participating SLT will notify colleague SLTs in the neighbourhood about TreatPaCS and will propose them to refer PCWS to her/his private practice.

8.1.2 Screening

In the first intake session (=screening), the participating SLT

- identifies the stuttering in the PCWS (with the measuring instrument SSI-4 or TVS-NL/TSB-NL).

- collects information from the parent about the PCWS, his/her stuttering and about the parent with the Anamnesis form (Appendix 3). The SLT records this in the PCWS' speech-language therapy file (on paper or electronically).
- educates the parents about stuttering to decrease the level of concern.
- decides, based on the age of the PCWS, the severity of the stuttering, the family history of the stuttering and the evolution of stuttering since onset, which action needs to be taken:

  (1) install a period of active monitoring of maximum 3 months to evaluate the progress of the stuttering (if signs of natural recovery are present),

  (2) initiate stuttering treatment or

  (3) not initiate stuttering treatment.

  If a period of active monitoring is installed, the SLT decides when sessions are scheduled (often every 4 or 6 weeks, depending on the need of the PCWS and the parents). The SLT decides how the parents record information about the stuttering (qualitative information by keeping a diary or quantitative data by recording daily typical stuttering severity scores on a 10-point scale with 0 = no stuttering, 1 = extremely mild stuttering and 9 = extremely severe stuttering). If the situation requires it, the SLT may provide advice, for example taking turns in conversations or not interrupting the PCWS when s/he is talking. The SLT does not model treatment techniques or introduces no techniques that require clinical follow-up. The SLT-file will provide an overview of advice that could be given during the active monitoring. The SLT decides based on the context of the PCWS and family if and when to initiate stuttering treatment.

  If stuttering treatment needs to be installed, the next session is the baseline session.

  If no stuttering treatment is initiated, the PCWS is not invited to participate in TreatPaCS.

- screens the PCWS and family for the inclusion and exclusion criteria.
- provides the parents with an information letter and consent form for TreatPaCS and asks consent of at least one of the parents (see 8.2 Consent). The signed consent form needs to be returned to the SLT at least 24 hours before the baseline session so that the PCWS is randomised before the start of the baseline session. The questionnaires need to be completed by the parent before the baseline session.

8.2 Consent

The participating SLT provides the parent(s) of the PCWS (who was successfully screened for eligibility) with an information letter and consent form. At least one of the parents signs the informed consent form. If the PCWS's parents are divorced, the SLT may consider it necessary to obtain both parents' signatures.

The sponsor's procedure regarding signing the informed consent form for minors in research studies requires that one of the parents signs the informed consent form. To increase understanding, a summary of the consent form with the most important points will be provided.

The participating SLT

- discusses with the parent the nature, objectives and possible risks of the TreatPaCS trial
- informs parents that the treatment approaches are delivered as standard of care treatment. Hence, they will have no extra costs. They will be asked to video record their child and answer some questionnaires at 7 points within 2 years and once after 5 years post-randomisation. For the first 7 times, parents will receive a voucher to compensate for the extra work because it is asked within the four-year period in which the study is funded.
- provides the opportunity to the parent(s) to ask questions
- assesses the parent's capacity to give his/her consent. The parent understands the purpose and nature of the trial, what research involves, the benefits of the trial, the risks and the burdens. The parent also understands what the alternative is to taking part. The parent is given sufficient time to make the decision and knows that it is a free choice.

The parent is asked to inform the participating SLT if s/he wants to participate in the TreatPaCS trial before the baseline session and to video record a speech sample of 10 to 15 minutes from a home situation before the next session. Clear instruction for how to record the video will be provided.

Parents know that the treatment will be standard of care.

An information form for the PCWS is also provided. The SLT informs the PCWS about the study by reading aloud what will happen at the child's language level and signs the form. This happens during the baseline session.

8.3 Trial randomisation

Stratified randomisation will be used according to site (30 sites) and gender. We will use a minimisation procedure to randomize. Minimisation assures similar distribution of selected participant factors between study groups. The first participant is truly randomly allocated; for each subsequent participant the imbalance score is computed based on all previous allocations as well as the hypothetical allocation of the current participant to each

treatment. The preferred treatment is then selected by choosing the treatment allocation associated with the smallest imbalance score.

The distance measure used to calculate the imbalance score is marginal balance. Marginal balance computes the cumulative difference between every possible pair of level counts (i.e. the number of participants in that particular factor level).

The allocation of the new participant is then made at random with a heavy weighting in favour of the intervention that would minimise imbalance. The remaining probability is equally divided between the non-preferred treatments. Full details will be provided in a separate document with restricted access.

If siblings or twins are eligible for this trial, only one child participate in the study otherwise there will be a dependency in the data. It is not feasible for a parent to implement two different treatment approaches. To avoid confusion, the second PCWS will receive the same treatment approach but will not be included in the study.

8.3.1 Method of implementing the allocation sequence

A web-based randomisation system QMinim will be used.

If the screening is successfully completed and the informed consent is signed by the parent(s), the SLT at the site fills out a Request to randomisation in REDCap. This randomisation form contains the unique enrolment ID from REDCap, year of birth and gender. Thereafter REDCap generates an automatic email to a staff member from CTC UZ-Antwerpen about the request including only the unique enrolment ID from REDCap. The staff member will fill in the values for the site and gender in QMinim in order to randomize the PCWS. The allocated treatment program and allocation ID (from QMinim) are filled in by the randomizer in REDCap. The allocation is performed in chronological order (based on the timing of the automatic emails from REDCap). The requests for randomisation are answered within 24 hours on weekdays (9 am-5 pm). The PCWS can start treatment the next day.

There is no need to have access to randomisation codes in case of an emergency as the study is open label. Hence, treatment will be known by the PCWS as well as SLT and recorded in the PCWS's speech-language therapy file and the electronic Case Report From (eCRF) (REDCap).

8.4 Blinding

The participating SLTs, PCWS and their parents are not blinded to the treatment approaches that they deliver/receive. The three treatment approaches (Mini-KIDS, SCBT and the LP) use distinct techniques. Blinding is thus impossible.

The video analysts who are scoring %SS and SR are blinded to the treatment approaches that the PCWS receives. In the unlikely event that a video analyst is unblinded for the treatment that is delivered by the SLT in a video, for example if the video analyst recognises the name of a PCWS in the video because it was mentioned by the SLT during a SLT-RCT committee meeting, the video analyst will stop rating the video and will transfer the video to another video analyst who is blinded to the treatment.

Two video analysts are not blinded and will assess the French and Dutch video recordings for treatment fidelity.

8.5 Unblinding

In the unlikely event that a video analyst is unblinded for the treatment that is delivered by the SLT in a video, the video analyst will stop rating the video and will transfer the video to another video analyst who is blinded to the treatment.

8.6 Baseline data

Before the PCWS and parent(s) attend the baseline session, the following data are collected:

- A dated informed consent form that is signed by one of the parents and returned to the participating SLT. A signed informed consent form is the condition for randomisation. The participating SLT completes a Request to randomisation in REDCap in order to start the randomisation. This needs to be done at the latest 24h before the baseline session on a weekday.
- A video recorded speech sample from a home situation of 10 to 15 minutes. The parent(s) brings this with him/her to the baseline session. These instructions for the video recording need to be followed:

  Instructions for the video recording of a speech sample (home and treatment session): The video is a recording of a conversation between the PWCS and an adult. The PCWS produces at least 300 syllables, which comes down to about 10 to 15 minutes of conversation in which the PCWS does most of the talking. The activity to elicit conversation with the PCWS can be a spontaneous conversation, describing a picture, telling a story, explaining a game or a similar language-promoting activity. **No treatment** is provided during this video recording (not at home, not during the treatment session).

  The parents/SLT may use a camera, a smartphone or a tablet device to video record the speech sample. It is important that the camera is placed close to the child so that the child's face is clearly visible and the child's voice is clearly heard. The child is positioned in an angle towards the camera so that the face movements are clearly visible in the video (see figure 2). It is important that this recording is done in a quiet environment, without external interruption, with sufficient light, and in the context of a game or conversation with the adult. The full name (first and last name) should never be mentioned in the video recordings.

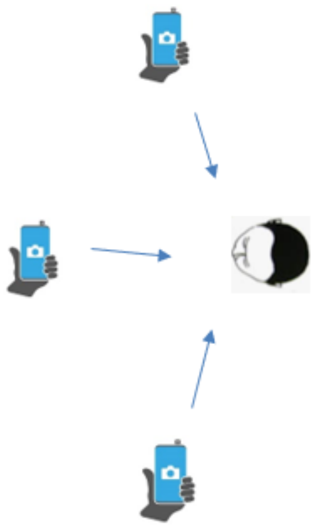

Figure 2: Position of PCWS and camera for video recording

At the baseline session, the following data are collected:

- A speech sample video recorded during the treatment session of the PCWS with the parent(s) and/or the SLT (ideally of the start of the session). The instructions for the video recording need to be followed.
- The SLT loads the video recorded speech sample that the parents bring to the baseline session.
- The stuttering severity score measured with the SSI-4 or TVS-NL/TSB-NL. The SLT puts the information in the eCRF.
- Scores on the KiddyCat (PCWS' attitude towards his/her speech, 12 questions that the SLT poses to the PCWS, Appendix 4). Consent to use the questionnaire in the eCRF for research purposes was obtained from the editor (Sig vzw). The SLT notes down the answers and puts the information in the eCRF straight after the baseline session.
- The information form for the PCWS. The SLT informs the PCWS at the child's language level and signs the form.
- Scores on the QOL (EQ-5D-Y proxy 1, six questions that the parent(s) rate how they think the PCWS would rate his/her own state if s/he were asked directly and could communicate it, Appendix 5). Consent to use the questionnaire in the eCRF for non-commercial purposes was obtained from the editor (Euroqol). This questionnaire is provided digitally and the parent(s) answers the questions on a digital device (computer, smart phone).
- Scores on the CBQ-short version (temperament, 94 questions that parent(s) rate about their PCWS, Appendix 6). Consent to use the questionnaire in the eCRF for research purposes was obtained

from the editor (Bowdoin College). This questionnaire is provided digitally and the parent(s) answers the questions on a digital device (computer, smartphone).

- The ISPP-questionnaire (Impact of Stuttering on Preschoolers and Parents, 20 questions that parent(s) answer about their child, Appendix 7). Consent to use the questionnaire in the eCRF for research purposes was obtained from the authors (Langevin, Packman & Onslow). This questionnaire is provided digitally and the parent(s) answers the questions on a digital device (computer, smart phone).
- Parent report on the PCWS's stuttering severity (Onslow et al., 2021) and satisfaction of communication in everyday speaking situations (based on but adapted from Karimi et al., 2018) (Appendix 8).

Completing these parent questionnaires was timed and is estimated at 1 minute for the QOL (EQ-5D-Y proxy 1), 13 minutes for the CBQ-short version, 10 minutes for the ISPP and 1 minute each for the stuttering severity and the satisfaction of the child's everyday communication.

The participating SLT also adds the information from the screening to the e-CRF:

- Answers to the Anamnesis form (Appendix 3). The SLT took notes (on paper or electronically) during the screening/ baseline session and puts the data in the eCRF straight after the baseline session.
- If the SLT installed a period of active monitoring before the treatment, the SLT puts the data of start and end date of the period of active monitoring and the information required during the monitoring period in the eCRF (stuttering severity rating on a numbered scale, qualitative information on the stuttering, other).

All instruments will be provided to the SLTs in a Dutch, a French and an English version, just as they are in this protocol. No Dutch and French versions are available for the ISPP yet. Therefore, the ISPP will be translated from English to French and Dutch, according to the WHO translation-back-translation procedure (WHO, n.d.).

8.7 Trial assessments

The trial assessments and trial procedures that follow are part of standard care, except for the data collected at the baseline session and at the 3-, 6-, 9-, 12-, 18-months and 2- and 5-year post-randomisation sessions. The trial assessments described here refer to the procedures undertaken in each treatment session at the data collection points.

8.7.1 Trial assessment for the three arms during the baseline session

The baseline session starts with a conversation between the PCWS and the parent(s) and/or SLT. The SLT video records this conversation. The child produces at least 300 syllables, which comes down to about 10 to 15 minutes of conversation in which the PCWS does most of the talking. The activity to elicit conversation with the PCWS can be a spontaneous conversation, describing a picture, telling a story, explaining a game or a similar verbal communication-promoting activity. No treatment is provided during this conversation. The instructions for the video recording need to be followed. This speech sample is loaded onto a GDPR-proof platform for analysis of the %SS and SR at a later time. The SSI-4 or TVS-NL/TSB-NL score is put in the eCRF after the baseline session.

The SLT administers the KiddyCAT to the PCWS during the baseline session, also if a child is younger than 3 years. S/he can take notes (on paper or electronically) and puts the answers into the eCRF straight after the baseline session.

The SLT went through the questions on the Anamnesis form during Screening and puts the information into the system at the baseline session.

The parent(s) completes the CBQ, the ISPP-questionnaire and the EQ-5D-Y proxy 1 before the baseline session, also if the child is younger than 3 (CBQ-short version) or 4 (EQ-5D-Y proxy 1) years of age. By answering the questionnaires digitally, the information is directly available in the database. If the questionnaires are completed by the parents, if the SLT administered the KiddyCAT and if the video recordings are uploaded to the GDPR-proof platform, the allocated treatment arm is revealed to the SLT and family. The SLT could administer more test instruments of the treatment arm requires so (see further).

Completing the questionnaires was estimated at 13 minutes for the CBQ-short version, 1 minute for the EQ-5D-Y proxy 1 and 10 minutes for the ISPP-questionnaire.

8.7.2 If randomised in the SCBT-arm

If a situation indicates so, additional instruments to the ones administered during the baseline session could be used to identify comorbid disorders. The CBQ-short version is used instead of the SDE (Scale for Description of Emotions, Boey, 2008) as it is a normed scale. SLTs can also administer the SDE if they require this information for the PCSW's treatment.

The baseline session in SCBT takes 1 hour 30 minutes, except if additional instruments are administered for comorbidities.

Results from the additional instruments are not recorded in the eCRF but are meant to individualise the treatment.

8.7.3 If randomised in the Mini-KIDS-arm

If a situation indicates so, additional instruments to the ones administered during the baseline session should be used to identify comorbid disorders. Other instruments (questionnaires) that are administered are the RSE-1 and RSE-2 (Reaction of the preschooler on the pseudo-Stuttering of the Examiner), the RCS (Reaction on Communicative Stressors), Parent-child interaction (observation checklist used in the Restart-DCM program) and ROSC (Reaction Of the parent on the Stuttering of the Child).

The baseline session in Mini-KIDS takes maximum 2 hours. If additional instruments are administered for comorbidities, the assessment takes longer. In that case it should be split up in two sessions.

Results from the additional instruments are not recorded in the eCRF but are meant to individualise the treatment.

8.7.4 If randomised in the LP

If a situation indicates so, additional instruments to the ones administered during the baseline session could be used to identify comorbid disorders. The 10-point stuttering rating scale (Onslow et al., 2021) is introduced to the parents to record daily severity ratings of the PCWS' typical stuttering throughout the day. The scale scores include 0 = for no stuttering, 1 = for extremely mild stuttering and 9 = for extremely severe stuttering. A stuttering severity chart is provided to record the daily scores (at the end of each day).

The baseline session for the PCWS who receives the LP takes 1 hour 30 minutes, except if additional instruments are administered for comorbidities but are meant to individualise the treatment.

Results from the additional instruments are not recorded in the eCRF.

8.8 Table of trial procedures

Figure 3 provides an overview of the trial procedures.

8.8.1 Screening:

During the screening, the SLT checks the eligibility of the PCWS. The SLT determines the presence of stuttering. The SLT also records the medical history of the PCWS with the focus on the stuttering history (stuttering onset, time since onset, family history of stuttering, ...) and uses the Anamnesis form (Appendix 3) to do this. The SLT informs the parent(s) of the PCWS about the nature and purpose of TreatPaCS and provides him/her with a consent form.

8.8.2 Period of active monitoring (optional)

A period of active monitoring can be installed if the stuttering shows signs of spontaneous recovery (e.g., decrease of stuttering severity since onset). A list of potential signs will be provided in the TreatPaCS manual (SLT site file). The SLT records the start and end date and the type of information that parents record during the period of active monitoring.

8.8.3 Baseline session

If the PCWS is eligible for the trial and at least one parent has signed the informed consent form, the PCWS is randomised in one of the three treatment groups (Mini-KIDS, SCBT or LP) when the PCWS and his/her parent(s) attend the SLT practice for the baseline session. The PCWS is assessed for speech attitude (KiddyCAT) and stuttering severity (SSI-4, TVS-NL or TSB-NL), and the SLT collects a video-recorded speech sample and loads this onto a GDPR-proof platform. No treatment is provided during this conversation. The instructions for the video recording need to be followed.

SLTs ask parents to report on their child's stuttering severity based on a 10-point scale with three descriptors only and to report on the satisfaction of the PCWS's communication in everyday speaking situations based on a 9-point scale with two descriptors only.

The SLT asks for the video recording that the parent(s) collected from his/her PCWS at home. This speech sample should contain at least 300 syllables which correlates to 10 to 15 minutes of speech if the PCWS speaks most of the time. No treatment is provided during this conversation. The instructions for the video recording need to be followed. The SLT loads the video from the parent onto a GDPR-proof platform and labels the video according to the requirements.

The parent is also asked to complete the CBQ, the EQ-5D-Y proxy 1 and the ISPP-questionnaire on an electronic device (computer or smart phone) before the baseline session. Access to these questionnaires is provided in an automatically sent email to the parent. The SLT checks in REDCap if the questionnaires were completed by the parent.

%SS and SR are scored by the blinded video analysts at a later time.

Treatment is installed after the baseline session at a weekly interval (in SCBT twice weekly) until the PCWS moves to the Maintenance Phase (part of treatment). The trial procedures of the treatment approaches are explained further in the protocol.

8.8.4 Each treatment session

The SLT completes a parent compliance checklist and a treatment fidelity checklist after each treatment session in the three treatment arms. The treatment compliance and fidelity checklists aim at increasing the

fidelity of treatment delivery, but also helps the SLT in delivering the treatment approach as per protocol. The description of this procedure is described below. The checklists are added in Appendix 9.

At one month post-randomisation, for 20% of the PCWS the SLT will be asked to video record entire treatment sessions, and to load those video recordings onto a GDPR-proof platform. The SLT will receive an automatic alert by email message if video recording is required. The instructions for this video recording need to be followed (these are different instructions than for recording the speech sample). To avoid bias, for example, SLTs who would behave differently during the video recording compared to other treatment sessions, they are asked to video record each session during that week if they see ≥ 3 PCWS in the study at that time or all treatment sessions during 3 weeks if they see < 3 PCWS at that time. The 20% correspond with two PCWS per site and will be chosen randomly with an equal distribution of the three delivered treatment approaches.

8.8.5 3-months post-randomisation data collection point

One week before the 3-month post-randomisation data collection point, the SLT receives an automatic alert that the 3-month data collection point approaches so the SLT can prepare the session.

At the session + or – 2 weeks the 3-months post-randomisation data collection point, the SLT video records a speech sample from the PCWS with the parent and/or the SLT at the beginning of the treatment session. The SLT loads this onto a GDPR-proof platform.

Like for each treatment session, the SLT checks for treatment compliance and treatment fidelity, based on the treatment arm. The description of this procedure is described below. The checklists are added in Appendix 9. The SLT reports any risks.

The parent is asked to video record a conversation with the PCWS at home. The SLT loads this a GDPR-proof platform and labels the video recording according to the requirements. No treatment is provided during this conversation. The instructions for the video recording need to be followed.

The parent is also asked to complete the EQ-5D-Y proxy 1 and the ISPP-questionnaire on an electronic device (computer or smart phone) within a fortnight. The parent is also asked to report on their child's stuttering severity based on a 10-point scale with three descriptors only and to report on the satisfaction of the PCWS's communication in everyday speaking situations based on a 9-point scale with two descriptors only. This will be automatically scheduled using REDCap and access to the questionnaires is emailed to the parent. The SLT checks in REDCap if the questionnaires were completed by the parent.

The SLT also ascertains that the information about the treatment sessions that were scheduled between the baseline and the 3-month data collection point is uploaded in REDCap: the date and duration of each session and the information of the compliance check and treatment fidelity checklist of each session.

For 20% of the PCWS the SLT will be asked to video record entire treatment sessions, and to load those video recordings onto a GDPR-proof platform. The SLT will receive an alert by email if video recording is required. To avoid bias, for example, SLTs who would behave differently during the video recording compared to other treatment sessions, they are asked to video record each session during that week if they see ≥ 3 PCWS in the study at that time or all treatment sessions during 3 weeks if they see < 3 PCWS at that time. The instructions for video recording the treatment session need to be followed (these are different instructions than for video recording the speech sample).

Instructions to record the video for treatment fidelity: The SLT may use a camera, a smartphone or a tablet device to video record the treatment session. It is important that the camera gives an overview of the clinic room. SLT, child and parent are in the video footage and their actions are clearly visible. The SLT's voice is clearly heard. The PCWS's full name should never be mentioned in the video recordings.

Blinded video analysts will score %SS and SR in the two video recorded speech samples of the PCWS at a later time. An unblinded video analyst will also check the treatment fidelity of the SLT. The procedure to assess SLT treatment fidelity is explained below.

8.8.6 6-months post-randomisation data collection point

One week before the 6-month post-randomisation data collection point, the SLT receives an automatic alert that the 6-month data collection point approaches so the SLT can prepare the session.

At the session + or – 2 weeks the 6-months post-randomisation data collection point, the SLT video records a speech sample from the PCWS with the parent and/or the SLT at the beginning of the treatment session. The SLT loads this onto a GDPR-proof platform.

Like for each treatment session, the SLT checks for treatment compliance and treatment fidelity, based on the treatment arm. The description of this procedure is described below. The checklists are added in Appendix 9. The SLT reports any risks.

The parent is asked to video record a conversation with the PCWS at home. The SLT loads this onto a GDPR-proof platform and labels the video recording according to the requirements. No treatment is provided during this conversation. The instructions for the video recording need to be followed.

The parent is also asked to report on their child's stuttering severity based on a 10-point scale with three descriptors only and to report on the satisfaction of the PCWS's communication in everyday speaking situations based on a 9-point scale with two descriptors only.

The SLT also ascertains that the information about the treatment sessions that were scheduled between the 3-month and 6-month data collection points is uploaded in REDCap: the date and duration of each session and the information of the compliance check and treatment fidelity checklist of each session.

For 20% of the PCWS the SLT will be asked to record entire treatment sessions, and to load those video recordings onto a GDPR-proof platform. The SLT will receive an automatic alert by email if video recording is required. To avoid bias, for example, SLTs who would behave differently during the video recording compared to other treatment sessions, they are asked to video record each session during that week if they see ≥ 3 PCWS in the study at that time or all treatment sessions during 3 weeks if they see < 3 PCWS at that time. The instructions for the video recording of the treatment session need to be followed (these are different instructions than for video recording the speech sample).

Video analysts will score %SS and SR in the two video recorded speech samples of the PCWS. A video analyst will also check the treatment fidelity of the SLT.

If the PCWS would have moved to the maintenance phase (still part of the treatment), the SLT reports any relapses and prepares the parent(s) for relapse management. Further in the protocol it is explained how they do this (depending on the treatment arm).

8.8.7 9-months post-randomisation data collection point

One week before the 9-month post-randomisation data collection point, the SLT receives an automatic alert that the 9-month data collection point approaches so the SLT can prepare the session.

At the session + or – 2 weeks the 9-months post-randomisation data collection point, the SLT video records a speech sample from the PCWS with the parent and/or the SLT at the beginning of the treatment session. The SLT loads this onto a GDPR-proof platform.

Like in each treatment session, the SLT checks for treatment compliance and treatment fidelity, based on the treatment arm. The description of this procedure is described below. The checklists are added in Appendix 9. The SLT reports any risks.

The parent is asked to video record a conversation with the PCWS at home. The SLT loads this onto a GDPR-proof platform and labels the video recording according to the requirements. No treatment is provided during this conversation. The instructions for the video recording need to be followed.

The parent is also asked to complete the EQ-5D-Y proxy 1 and the ISPP-questionnaire on an electronic device (computer or smart phone) within a fortnight. The parent is also asked to report on their child's stuttering severity based on a 10-point scale with three descriptors only and to report on the satisfaction of the PCWS's communication in everyday speaking situations based on a 9-point scale with two descriptors only. This will be automatically scheduled using REDCap and access to the questionnaires is emailed to the parent. The SLT checks in REDCap if the questionnaires were completed by the parent.

The SLT also ascertains that the information about the treatment sessions that were scheduled between the 6-month and 9-month data collection points is uploaded in REDCap: the date and duration of each session and the information of the compliance check and treatment fidelity checklist of each session.

Video analysts will score %SS and SR in the two video recorded speech samples of the PCWS. A video analyst will also check the treatment fidelity of the SLT.

If the PCWS would have moved to the maintenance phase (still part of the treatment), the SLT reports any relapses and prepares the parent(s) for relapse management. Further in the protocol it is explained how they do this (depending on the treatment arm).

8.8.8 12-months post-randomisation data collection point

One week before the 12-month post-randomisation data collection point, the SLT receives an automatic alert that the 12-month data collection point approaches so the SLT can prepare the session.

At the session + or – 2 weeks the 12-months post-randomisation data collection point, the SLT video records a speech sample from the PCWS with the parent and/or the SLT at the beginning of the treatment session. The SLT loads this onto a GDPR-proof platform.

Like in each treatment session, the SLT checks for treatment compliance and treatment fidelity, based on the treatment arm. The description of this procedure is described below. The checklists are added in Appendix 9. The SLT reports any risks.

The parent is asked to video record a conversation with the PCWS at home. The SLT loads this onto a GDPR-proof platform and labels the video recording according to the requirements. No treatment is provided during this conversation. The instructions for the video recording need to be followed.

The parent is also asked to report on their child's stuttering severity based on a 10-point scale with three descriptors only and to report on the satisfaction of the PCWS's communication in everyday speaking situations based on a 9-point scale with two descriptors only.

The SLT also ascertains that the information about the treatment sessions that were scheduled between the 9-month and 12-month data collection points is uploaded in REDCap: the date and duration of each session and the information of the compliance check and treatment fidelity checklist of each session.

For 20% of the PCWS the SLT will be asked to record entire treatment sessions, and to load those video recordings onto a GDPR-proof platform. The SLT will receive an automatic alert by email if video recording is required. To avoid bias, for example, SLTs who would behave differently during the video recording compared to other treatment sessions, they are asked to video record each session during that week if they see ≥ 3 PCWS in the study at that time or all treatment sessions during 3 weeks if they see < 3 PCWS

at that time. The instructions for the video recording the treatment session need to be followed (these are different instructions than for video recording the speech sample).

Video analysts will score %SS and SR in the two video recorded speech samples of the PCWS. A video analyst will also check the treatment fidelity of the SLT.

If the PCWS would have moved to the maintenance phase (still part of the treatment), the SLT reports any relapses and prepares the parent(s) for relapse management. Further in the protocol it is explained how they do this (depending on the treatment arm).

8.8.9 18-months post-randomisation data collection point (primary data collection point)

Two weeks before the 18-month post-randomisation data collection point, the SLT receives an automatic alert that the 18-month data collection point approaches so the SLT can prepare the session.

At the session + or – 2 weeks the 18-months post-randomisation data collection point, the primary data collection point in this trial, the SLT video records a speech sample from the PCWS with the parent and/or the SLT at the beginning of the treatment session. The SLT loads this onto a GDPR-proof platform. The SLT also assesses the PCWS for his/her speech attitude (KiddyCAT).

Like in each treatment session, the SLT checks for treatment compliance and treatment fidelity, based on the treatment arm. The description of this procedure is described below. The checklists are added in Appendix 9. The SLT reports any risks.

The parent is asked to video record a conversation with the PCWS at home. The SLT loads this onto a GDPR-proof platform and labels the video recording according to the requirements. No treatment is provided during this conversation. The instructions for the video recording need to be followed.

Video analysts will score %SS and SR in the two video recorded speech samples of the PCWS.

If the PCWS would have moved to the maintenance phase (still part of the treatment), the SLT reports any relapses and prepares the parent(s) for relapse management. Further in the protocol it is explained how they do this (depending on the treatment arm).

The parent is also asked to complete the EQ-5D-Y proxy 1 and the ISPP-questionnaire on an electronic device (computer or smart phone) within a fortnight. The parent is also asked to report on their child's stuttering severity based on a 10-point scale with three descriptors only and to report on the satisfaction of the PCWS's communication in everyday speaking situations based on a 9-point scale with two descriptors only. This will be automatically scheduled using REDCap and access to the questionnaires is emailed to the parent. The SLT checks in REDCap if the questionnaires were completed by the parent.

The SLT also ascertains that the information about the treatment sessions that were scheduled between the 12-month and 18-month data collection points is uploaded in REDCap: the date and duration of each session and the information of the compliance check and treatment fidelity checklist of each session.

%SS and SR will be scored by the blinded video analysts at a later time.

8.8.10 2-years post-randomisation data collection point (Follow-up)

Four weeks before the 2-year post-randomisation data collection point, the SLT receives an automatic alert that the 2-year data collection point approaches so the SLT can prepare the session.

At the session + or – 4 weeks the 2-years post-randomisation data collection point, the SLT video records a speech sample from the PCWS with the parent and/or the SLT at the beginning of the treatment session and loads this onto a GDPR-proof platform. The SLT also asks the parent to video record a conversation with the PCWS at home. The SLT loads this onto a GDPR-proof platform and labels the video according to the requirements. No treatment is provided during this conversation. The instructions for the video recording need to be followed.

Video analysts will score %SS and SR in the two video recorded speech samples of the PCWS.

If the PCWS is still in the maintenance phase (part of the treatment), the SLT reports any relapses and prepares the parent(s) for relapse management. Further in the protocol it is explained how they do this (depending on the treatment arm).

The parent is asked to complete the EQ-5D-Y proxy 1 on an electronic device (computer or smart phone) within 4 weeks before or after the data collection point. The parent is also asked to report on their child's stuttering severity based on a 10-point scale with three descriptors only and to report on the satisfaction of the PCWS's communication in everyday speaking situations based on a 9-point scale with two descriptors only. This will be automatically scheduled using REDCap and access to the questionnaire is emailed to the parent. The SLT checks in REDCap if the questionnaires were completed by the parent.

If the PCWS is still receiving treatment (and is not in Maintenance Phase), the SLT also ascertains that the information about the treatment sessions that were scheduled between the 18-month and the 2-year data collection points is provided: the date and duration of each session and the information of the compliance check and treatment fidelity checklist of each session.

8.8.11 5-years post-randomisation data collection point (Follow-up)

At the session + or – 4 weeks the 5-years post-randomisation data collection point, the CI and/or Co-CIs ask the parent to video record a conversation with the PCWS at home. The parent loads this onto a GDPR-proof

platform and labels the video recording according to the requirements. No treatment is provided during this conversation. The instructions for the video recording need to be followed.

Video analysts will score %SS and SR in the video recorded speech samples of the PCWS.

The parent is asked to complete the EQ-5D-Y proxy 1 on an electronic device (computer or smart phone) within 4 weeks before or after the data collection point. The parent is also asked to report on their child's stuttering severity based on a 10-point scale with three descriptors only and to report on the satisfaction of the PCWS's communication in everyday speaking situations based on a 9-point scale with two descriptors only. Access to the questionnaires is emailed to the parent.

8.9 Fidelity of implementation

Bergþórsdóttir et al. (2021) reported that clinical trials often fail to report fidelity of implementation. In treatment for preschool children who stutter, the treatment is not only implemented during the treatment session by the SLT, but also at home by the parents. It is therefore necessary to check compliance of implementation at home and during the treatment session.

In TreatPaCS, we therefore check (1) the parent's compliance and (2) the SLT's treatment delivery in the practice (for treatment fidelity). Four main components will be assessed:

- Dosage of the treatment. This includes how often the treatment was delivered, number of sessions attended, session length, length of the treatment period, length of practice time at home, number of practice times at home, ... This information is collected by the SLT when she is completing the compliance checklist.
- Adherence of the treatment. This refers to the accuracy with which the treatment was delivered. This information is known from the treatment fidelity checklists and the video recordings of the treatment sessions.
- Quality of the treatment. This refers to how well the intervention is delivered. It includes the skill with which the intervention was delivered by the parent and/or SLT, the parent's ability to engage with the child and awareness of treatment protocols. This information is collected by the SLT when she is completing the compliance checklist and the treatment fidelity checklist.
- Responsiveness to the treatment and engagement in the treatment. This is based on parent and/or child satisfaction reported by the SLT in the compliance checklists and/or direct observation of the child's behaviour as the child is the one the treatment is focused on.

Fidelity of implementation is often (and also in this trial) measured by the use of checklists, automatic recording devices, and independent observer judgment.

As mentioned previously, 20% of the PCWS the SLT will be asked to record entire treatment sessions, and to load those video recordings onto a GDPR-proof platform. The 20% correspond with two PCWS per site and will be chosen randomly with an equal distribution of the three delivered treatment approaches. The SLT will receive an automatic alert by email message if video recording is required. To avoid bias, for example, SLTs who would behave differently during the video recording compared to other treatment sessions, they are asked to video record each session during that week if they see ≥ 3 PCWS in the study at that time or all treatment sessions during 3 weeks if they see < 3 PCWS at that time. The instructions for the video recording the treatment session need to be followed (these are different instructions than for video recording the speech sample).

8.9.1 Parent treatment compliance

SLTs use a compliance checklist during each treatment session to assess treatment compliance at home for each type of treatment (see Appendix 9). The checklist mainly includes questions about treatment implementation (dosage and adherence). During the treatment session, the SLT asks the parent to demonstrate or observes how s/he implements the treatment at home. The observation of this parent demonstration provides an answer to the aspects of quality of treatment implementation and responsiveness to the treatment. The presence of at least 80% of treatment aspects defines acceptable parent treatment compliance. The SLT uses an electronic checklist or a paper-based checklist that s/he keeps in the PCWS' speech-language therapy file. The SLT uploads the information in REDCap at each data collection point.

8.9.2 SLT treatment fidelity

To assess SLTs' treatment fidelity to dosage and adherence, automatic recorded data are used. In addition, SLTs make a video recording of their treatment session with the PCWS and parent 1, 3, 6 and 12 months post-randomisation. Instructions for the video recording are provided.

The videos are assessed by two unblinded video analysts who have expertise in the three types of treatments. The French-speaking video analyst watches the videos of the treatment delivered in French; the Dutch-speaking video analyst watches the videos of the treatment delivered in Dutch. A treatment fidelity checklist consisting of treatment aspects related to quality of treatment implementation and responsiveness to the treatment is used (see Appendix 9). In case inconsistencies are observed, the second video analyst, independent of the TreatPaCS trial, watches the video recording as well. The presence of at least 80% of treatment aspects defines acceptable SLT treatment fidelity.

The unblinded video analysts won't be scoring the other video recordings (speech samples) for %SS and SR.

8.9.3 Video analysis: Rating of %SS and SR

The video analyst coordinator organises the video analysis process. The video analysts look at the videos that are uploaded onto a GDPR-proof platform to calculate %SS and assign a SR to the two video recordings (one from home and one from the treatment session) at each data collection point. An average of the two scores will be taken as outcome measure. Success of the intervention is frequently defined in terms of achieving <1% SS. To achieve such a low average, one must have very low scores in both speech samples (home and during the treatment sessions), so averaging the % SS is routine practice in the standard clinical care.

The video analysts will be able to access the video recordings without knowing which treatment approach the PCWS is receiving/has received. A list of the videos of the French-speaking PCWS (for the French-speaking video analysts) and a list of the videos of the Dutch-speaking PCWS (for the Dutch-speaking video analysts) will be generated without information about the treatment arm and the time on which the video is recorded. In each list, 5% of the video recordings will re-appear as those are intended to be rated by two video analysts to establish the interrater agreement (see further for detail about the interrater agreement analysis). This 10% of videos is clearly indicated on the list. The video analysis will commence 6 months after the start of recruitment. This 6-month waiting time is included to avoid bias for the baseline video recordings.

If a PCWS speaks another language than Dutch, French or English, the research team will use their network to search for a native speaker of the language spoken by the PCWS and parent. This person will be invited to help the video analyst with scoring the video.

Standardisation of the video analysts will be guaranteed by a manual and by a training in which three video recorded speech samples (other than TreatPaCS-videos) will be scored together with all video analysts for %SS and SR.

Quality of the ratings will be assured by an evaluation of five video samples of French-speaking PCWS performed by the French-speaking video analysists and five video samples of Dutch-speaking PCWS performed by the Dutch-speaking video analysists. In a meeting they will discuss the stuttering moments and come to a consensus.

For %SS, clear instructions will be provided, including:

- Only the stuttering behaviour is counted; not the normal disfluencies (a more detailed stuttering type is not to be considered in this judgement). The stuttering behaviour may appear with or without tension.
- When more than one stuttering behaviour appear on one syllable (for example, a block and a repetition such as: ... [tension, no sound] ... I-I-I-I want ... (= block, sound repetition) it is counted as one as it occurs on one syllable.

- For counting the total number of syllables, clear instructions are given for what to include in the counting and what not. For example, isolated /yes/ or /no/ are not to be counted as a syllable.

For SR, tools are provided, including:

- A visual scoring chart such as the one used in the LP (Onslow et al., 2021) will be provided with 0 = no stuttering, 1 = extremely mild stuttering and 9 = extremely severe stuttering.
- Five supporting questions will be provided to help the video analysts assign the SR score:

STUTTERING SEVERITY SCORES					
	**Score 0**	**Score 1-2**	**Score 3, 4 and 5**	**Score 6, 7 and 8**	**Score 9**
**Severity of stuttering**	No stuttering	Extremely mild/mild	Moderate	Severe	Extremely severe
**What types of stutters appear and how often?**	No stutters	Very occasional/ occasional repetitions and/or very occasional and brief prolongations and/or blocks	More frequent repetitions and/or prolongations and/or blocks	Frequent repetitions and/or prolongations and/or blocks	Frequent and severe repetitions and/or prolongations and/or blocks
**How does the child get his/her message across?**	Gets message across easily	Gets messages across easily/ quite easily	May take some time and effort to get message across	Takes lots of time and sometimes cannot get message across at all	Usually cannot get message across at all
**Does the child's speech sound effortful?**	No	No	Sometimes	Most of the time	Almost always
**Would another person notice the stuttering?**	There is no stuttering	Probably not/ Probably	Certainly	Certainly; likely to feel uncomfortable about it	Certainly; will frequently feel uncomfortable about it

A manual with clear guidelines will be provided to the video analysts in which it is described how they should count the disfluencies. Also, a clear description of how to assign SR will be given. This manual will be added to the Trial Master File.

Videos of PCWS who speak a language other than Dutch, French or English will be viewed by a video analyst and a person who speaks the language of the child. The video analyst can ask the speaker of the language for advice in case ambiguous disfluencies are present in the speech sample.

Inter-rater agreement will be computed for 10% of the videos. Inter-rater agreement will be calculated with an Intraclass Correlation Coefficient (ICC).

	Pre-treatment			Treatment (Tx)					Follow-up	
	Screening at SLT practice	Active monitoring°	Baseline	Tx at 3 months post-randomisation +/- 2 weeks	Tx at 6 months post-randomisation +/- 2 weeks	Tx at 9 months post-randomisation +/- 2 weeks	Tx at 12 months post-randomisation +/- 2 weeks	Tx at 18 months post-randomisation +/- 2 weeks	2yr post-randomisation +/- 4 weeks	5yr post-randomisation +/- 4 weeks
	(Day -92 - Day 0)	(if needed)	(Day 1)	(weekly-twice weekly interval with previous Tx session#)	(interval: weekly-twice weekly interval with previous Tx session# or greater interval if Tx is in Maintenance Phase)					
Informed consent form signed by one parent	x									
Information form for the child signed by the SLT			x							
Eligibility assessment	x									
Anamnesis (time since onset, gender, ...)	x									
Active monitoring data			(x)							
Randomisation			x							
Stuttering severity by SLT (SSI-4, TVS-NL/TSB-NL)	x		(x)							
Treatment depending on arm			x	x	x	x	x	**x**	(x)	
Speech sample from Tx session			x	x	x	x	x	**x**	(x)	
Speech sample from home			x	x	x	x	x	**x**	x	x
%SS **			x	x	x	x	x	**x**	x	x
SR **			x	x	x	x	x	**x**	x	x
KiddyCAT			x					**x**		
Treatment date & duration				x	x	x	x	**x**	(x)	
Compliance & treatment fidelity°°, @				x	x	x	x	**x**	(x)	
QOL (EQ-5D-Y proxy 1)			x	x		x		**x**	x	x
CBQ-short version			x							
ISPP-questionnaire			x	x		x		**x**		
Parent report about stuttering and satisfaction everyday communication			x	x	x	x	x	**x**	x	x
Drop-out/leave the allocated treatment arm				x	x	x	x	**x**	(x)	
Relapse reporting and relapse management					x*	x*	x*	**x**	(x)	
Risk assessment				x	x	x	x	**x**	x	

Tx = Treatment; %SS = Percentage syllables stuttered; SR = Severity Rating; CAT = Communication Attitude Test; QOL = Quality of Life; CBQ = Children's Behaviour Questionnaire; ISPP = Impact of Stuttering on Preschoolers and Parents; °: if applicable, that is, if treatment is not initiated straight away; * if applicable, that is, if the child is in Maintenance Phase. (x) If applicable, that is, if the child is still seeing the SLT; Underlined & bold (18 months post-randomisation) = primary end point; ** performed by the video analysts; # depending on the treatment arm;°° Parent compliance and treatment fidelity checklists are completed for each treatment session; @ Treatment fidelity is also evaluated based on of video that is recorded for 20% of the PCWS (entire treatment session) at 1-, 3-, 6- and 12-months post-randomisation.

Figure 3. Overview of measurements in TreatPaCS

8.10 Withdrawal criteria

8.10.1 Discontinuation of trial intervention (without withdrawal of consent)

During the entire treatment phase, the reason and date for drop-out of treatment or the reason, date for leaving the allocated treatment arm are recorded in the e-CRF as well as whether it was the PCWS's, the parent's or the SLT's decision.
Reasons for stopping trial intervention (protocol treatment) may include, but are not limited to:

 The PCWS and/or PCWS' parent do(es) not wish to continue with trial intervention

 Safety reasons (Checklist Safety Reporting)

 Compliance issues of the parent(s). If planned sessions are not attended by the PCWS and parent(s) without a valid reason, it is a compliance issue.

(1) The SLT may work around holidays or real-life situations in which a PCWS and the parent(s) cannot attend the session for one or more times (eg., illness of a parent, transport issues for some time, ...). These postponed sessions are limited in number and the SLT prepares the PCWS and parent for this gap in treatment.

The PCWS and the parent(s) can miss treatment sessions at most for three consecutive weeks without a valid reason before they are asked to withdraw from the trial intervention.

(2) The parent(s) are asked to implement the treatment at home. In the Mini-KIDS treatment arm, they agree on their involvement in the mandate. In the LP and SCBT treatment arm, they agree to implement the treatment daily at home for about 10 minutes (LP) or two to three times per week for 20 to 30 minutes (SCBT) for making speaking activities pleasant again. If the SLT records compliance issues that violates those listed in the compliance checklists for each treatment arm, these will be discussed by the Co-CI and CI. It may a reason for discontinuation.

 Treatment fidelity issues of the SLT

The video analyst may notice that a SLT adds treatment techniques or omits treatment techniques for the treatment approach that s/he is delivering compared to the treatment fidelity checklists for each treatment arm in this trial. If the SLT does not follow the protocol for more than 80% for a treatment session that was recorded on video, the SLT will be asked to follow the protocol more rigorously. If the SLT does not follow the protocol for more than 80% for each session indicated on the treatment fidelity checklists for more than six sessions, the SLT is considered to drift from the protocolised treatment approach and may be withdrawn from the trial intervention. Because data collection points often have more than a 6-week interval, SLTs are asked to report problems with treatment fidelity.

☐ Other reason

The trial will be analysed on an intention-to-treat basis and per-protocol analysis (Mo et al., 2021). All PCWS who stop randomised trial intervention will remain in the trial for follow-up unless the parent explicitly withdraws consent for data collection.

A PCWS's parent who refuses some or all trial procedures will still be followed up with the data collection procedures that the PCWS's parent(s) is agreeing unless the PCWS's parent(s) explicitly withdraws consent.

If the parent(s) discontinues with the trial intervention and did not withdraw consent, the PCWS will leave the allocated treatment arm or will receive other stuttering treatment than the manualised TreatPaCS treatment approaches from the SLT. The SLT will provide a video recorded speech sample from during the treatment session and from home, and the scores on the KiddyCAT, EQ-5D-Y proxy 1 and ISPP-questionnaire at the set data collection points during the trial after randomisation.

When a PCWS discontinues the trial intervention, a letter to inform the trial procedures for discontinuation will be provided to the PCWS and his/her parent(s). The parent(s) may decide to withdraw consent if s/he is not willing to provide the follow-up data.

The details of discontinuation (reason) should be clearly documented in the PCWS's speech-language therapy file and in the eCRF.

8.10.2 Discontinuation of study (with withdrawal of consent)

The parent(s) may withdraw consent at any time during the study. For the purposes of this trial, withdrawal is defined as:
The PCWS and his/her parent withdraw consent from TreatPaCS and are not willing to be followed up for the purposes of the trial at any further data collection points (i.e., only data collected prior to the withdrawal of consent can be used in the trial analysis, no data can be collected anymore from this time point).
The details of withdrawal (reason) should be clearly documented in the PCWS's speech-language therapy files and in the eCRF.

8.10.3 Loss to follow-up

If a PCWS is lost to follow-up, SLTs make every effort to contact the PCWS to obtain information on the PCWS's status. Similarly, if a PCWS's care is transferred to another SLT, SLTs make every effort so that follow-up information is obtained.

The SLT will make at least three attempt to contact the PCSW or the parent(s) before the PCWS is lost to follow-up. All attempts are documented into the PCWS' speech-language therapy files.

8.11 End of trial

A PCWS is considered to have completed TreatPaCS if any of the following apply:

- Completion of the treatment and data collected at the 2-year data collection point
- Lost to follow-up, after three attempts to reach the PCWS
- Withdrawal of informed consent form.

The end of the entire trial will occur when one of the following situations occurs:

- When all PCWS have completed their treatment and data is collected at the 2-year data collection point, are lost to follow-up or withdrew consent
- If the trial is terminated by Thomas More Mechelen-Antwerpen for safety reasons.

The Ethics Committee (EC) of UZ-Antwerpen will be notified within the 90 days of TreatPaCS' completion.

9 TRIAL INTERVENTION

The initial six treatment sessions with parent(s) and PCWS in Mini-KIDS and in the LP are 60-minute sessions. The Lidcombe and Mini-KIDS programs were designed to be delivered in weekly 60-minute treatment sessions (Onslow et al., 2021; Waelkens, 2018). However, the current reimbursement rules of INAMI-RIZIV do not allow for reimbursement of 60-minute sessions with children under 10 years of age. Therefore, we have included in the budget the reimbursement for an additional 30 minutes of care for the first six sessions of Mini-KIDS and the LP. This is in line with good practice recommendations for these two programs. All other treatment sessions of 30 minutes and parent sessions of 60 minutes are reimbursed through the existing health care system. The SCBT program is delivered in two 30-minute sessions per week and parent sessions of 60 minutes.

All trial procedures are standard care, except for the questionnaires that are administered at the baseline session, the 3-, 9-, and 18-month, the 2-years and 5-years post randomisation data collection points and

the speech samples that are recorded and uploaded at the 3-, 6-, 9-, 12-, 18-months and 2- and 5-year post-randomisation data colelction points.

9.1 Trial procedures in the SCBT

A parent training is organised for parents with PCWS who receive SCBT (Boey, 2010). The parent training groups can also include parents of PCWS who receive SCBT and do not participate on the TreatPaCS trial. Parents of the PCWS who receive Mini-KIDS or the LP **cannot** be included in this training.

The parent training usually comprises of 8 to 10 one-hour group evening sessions for parents to discuss and offer education topics about what stuttering is, what stuttering looks like, how stuttering looks like at onset, how overt stuttering can develop, explanation of the covert features of stuttering, what stuttering can elicit, what the influence of conversational circumstances and listener reactions are. The parent sessions aim at changing parents' attitude towards stuttering by desensitising and cognitive restructuring. The idea is that a changed attitude (adequate response to the stuttering and giving the correct example) is only possible if the attitude is aligned to neutral positive cognitions and emotions. They learn to analyse a speaking situation, to deduce and implement appropriate interventions. They learn this under guidance of the SLT with the aim to do this without their help. The interventions are directed at situations, the behaviour of the PCWS, the behaviour of people in the environment of the PCWS (home, school, other).

Treatment sessions of 30 minutes are scheduled usually on a twice weekly basis, depending on the parents' availability. These treatment sessions are at the SLT's private practice and are attended by at least one parent and the PCWS.

The program is delivered in five phases and each phase is only delivered if necessary and takes as long as is necessary for the PCWS (variable): (1) conditioning speaking activities, (2) cognitive training focused on emotions, (3) cognitive training focused on cognitions, (4) desensitisation and (5) skill training.

9.1.1 Conditioning of speaking activities

Conditioning of speaking activities aims at making speaking pleasant again. An activity that avoids stuttering-evoking aspects such as time pressure, cognitive load or linguistic complexity is chosen. The conditioning during this activity can include

- Removing time pressure
- Simplifying language content
- Decreasing the articulatory complexity requirements

- Decreasing the communication pressure
- Avoiding distraction (turn off mobile phones)
- Keeping the listener reactions neutral and pleasant
- Adding a neutral and positive emotional atmosphere
- Using clear rules for disruptive behaviour

The parent(s) observes the first few sessions of the treatment and then joins the interaction. Verbal and non-verbal praise are given to the parent for his/her adequate responses and speaking model. The parent(s) is asked to implement this technique at home two or three times per week for 20 to 30 minutes. These techniques need to be implemented at least two times per week for 3 months, followed by once per week for another 3 months.

These techniques can be expanded by introducing other activities after four or five sessions, e.g., playing another game, using different toys, talking about pictures in a book. These techniques can also be expanded by 'embedding': A listener shadows what the PCWS says and adds an element of information to the phrase. If the PCWS stops showing the target responses, the listener should not use embedding any longer.

9.1.2 Cognitive training focused on emotions

The PCWS needs to be able to label and recognise emotions as they are an important part of stuttering. The emotions need to be introduced as neutral concepts to the PCWS through play. At first, the focus is on the game, then it moves to the content.

The goal is that the PCWS shows behaviour that helps him/her escape stuttering-inducing factors or that helps him/her cope with them. Most often these emotions are excitement and moments with some time pressure.

After the initial basic emotions, the PCWS moves to ventilating (labelling) more complex emotions such as excitement, busy situations, stress, tension, impatience, frustration and irritation. Opposite emotions in visuals (pictures, drawings) are often used to explain them. The PCWS also learns what ignites those emotions for him/her.

Sometimes it is necessary to teach a PCWS to counter his/her thoughts (cognitive restructuring). Pedagogical themes such as going to bed are used to practise this technique. Books and role play are useful to work on restructuring the thoughts. The goal is that a PCWS can regulate his/her own emotional responses on stress-inducing events without intervention of another person. Praise is provided whenever the PCWS show any behaviour in that direction. A token economy system can be introduced. That is, the PCWS receives a reward each time s/he can counter a thought/emotion.

The SLT can work on more themes, such as being happy-excited, scared-excited, jealous, perfectionist and patient-impatient.

9.1.3 Cognitive training focused on cognitions

The cognitive training aims at restructuring cognitions by

- Teaching the PCWS to formulate positive judgements about him/herself
- Helping the PCWS to think about norms and standards
- Teaching the PCWS to problem-solve: formulate problem-solving behaviour, understand why a behaviour is deviating, generate solutions, select a solution and execute and evaluate the consequences. It may be necessary to 'drill' this skill. That is, to intensively offer problems that require a similar solution and provide praise whenever the PCWS shows coping skills. Role play facilitates the generalisation of what the PCWS has learnt.
- Teaching concepts and self-knowledge about speaking (about vocabulary and language, about normal disfluencies, about stuttering, about listeners)

Picture or story books are excellent resources to train these skills (e. g., Max Velthuijs (2005) Kikker op avontuur. Leopold Amsterdam).

9.1.4 Desensitisation

This phase is also often referred to as "Emotional training". Some PCWS need additional support besides the cognitive training to increase the frustration threshold and to decrease their anxiety for speaking. The training is focused on desensitisation for the PCWS's own speech and listener reactions.

This phase start with desensitisation of normal disfluencies. The SLT introduces the pseudo-stuttering, but parent(s) and siblings implement it as well. PCWS, older than 3 years of age, implement this too. Then follows the desensitisation of stuttering moments, mainly sound- and syllable repetitions. For some PCWS, it is necessary to also desensitise prolongations and blocks. The SLT needs to be vigilant to add comments to help the PCWS to counter negative associations to stuttering.

Finally, also positive associations towards the PCWS's own speaking competences are aimed for to trigger positive feelings.

9.1.5 Skill training

The goal of this phase is to train skills to use speech and language diversely to ask, to request, to explain, to make stories, to tell tales, to fantasize, ...

Conversation skills may also be introduced and practised such as taking turns (and waiting turns), speaking up, learning to interrupt, asking additional questions as well as listener skills such as giving feedback, giving attention or saying goodbye. The applied method is motivating the PCWS, modelling, concretising, associating neutral and positive emotions and applying skill training techniques.

Social conversation skills refer to verbal and non-verbal behaviour in interaction with other children and adults. Verbal behaviour includes language use and conversation skills. Non-verbal behaviour includes posture, gestures, eye contact and facial expression.

Training articulation skills is useful in young children who stutter severely, who have speech production skills below expectation and respond to the stuttering with irritation or frustration. The SLT mainly stimulates the PCWS and avoids direct interventions such as placing a tongue spatula. These skills include mobility with tongue and lips, sound imitation, strength and tension, diadochokinetic and sound-specific exercises.

Finally, variation of speech and voice lead to an increase of feelings of pleasure and being a competent speaker. These also create conditions in which the entire language and speech motor system becomes more flexible to make (unconscious) adjustments which can therefore also change the behavioural responses to stuttering (e.g. secondary behaviour, muscle tone...).

The PCWS can conclude the treatment phase and move to the maintenance phase when (1) the stuttering does not develop, (2) there is a reduction of stuttering severity during at least 3 to 4 months, (3) the PCWS developed a positive attitude towards his/her speech, (4) the PCWS is resilient towards stuttering-inducing factors and (5) the parent(s) provides a more stable and fluency-inducing behavioural model environment to the PCWS and deals independently and appropriately with the PCWS's stuttering.

9.1.6 Maintenance phase

The maintenance phase starts when the PCWS and parent(s) achieved the goals of the five phases. Usually the stuttering is reduced to (near) zero stuttering. Treatment sessions to monitor if achieved goals are maintained are scheduled with an interval of 2, 2, 4, 4, 8, 8 and 16 weeks. If necessary, SLTs add extra sessions during maintenance, for example if the goals were not maintained.

During the maintenance sessions, the SLT asks the parent(s) to report about the PCWS and observes the speech of the PCWS. Depending on the speech of the PCWS, a decision is taken:

- The PCWS does not stutter anymore: the parent(s) continues to observe closely but there is no need to re-introduce treatment techniques.
- The PCWS responds with stuttering to stuttering-inducing factors: the parent(s) re-instates specific treatment techniques depending on the situation or the PCWS receives training on certain concepts or skills.
- The PCWS stutters severely: the SLT may decide to re-introduce specific treatment concepts or skills and may ask the parent(s) to implement them at home.

9.2 Trial procedures in Mini-KIDS

The Mini-KIDS treatment guide (Waelkens, 2018) prescribes treatment sessions for Stage 1 to 4 to take 60 minutes. This, however, is also not standard Belgian care as only treatment sessions of 30 minutes with parent and PCWS are reimbursed. Therefore, this study limited the one hour sessions to the first six treatment sessions. These sessions are scheduled with one PCWS and his/her parent(s) alone (not as group sessions). The parent receives education about stuttering and is coached to implement the pseudo-stuttering techniques.

Mini-KIDS for 4-6-year old PCWS consists of four stages: Stage 1 = desensitization, Stage 2 = identification, Stage 3 = modification and Stage 4 = generalization (Waelkens, 2018). The program for 2-4-year old PCWS does not include stage 2. SLT and parent(s) are the speech model for the PCWS. They add normal disfluencies and pseudo-stuttering to their speech at first to make sure the PCWS dares to stutter and the PCWS as well as the parent(s) are desensitized for it. Later on in treatment and if necessary, PCWS learn to recognise and alter their stuttering moments.

9.2.1 Mandate

A mandate is formulated with both parents. The mandate specifies the treatment expectations, achievable goals and the requirements for parental involvement. Also with the PCWS, a mandate is formulated to inform him/her about what is going to happen on an individualized level. This mandate sets a positive working relation between SLT, the parent(s) and the CWS.

9.2.2 Stage 1 = Desensitisation

SLT, parent(s) and PCWS gradually learn to dare to pseudo-stutter. For the PCWS, the goal is to dare to use all relevant types of pseudo-stuttering (relevant = what is present in the PCWS' speech). For the parent(s), apart from daring to pseudo-stutter, it is also important that they perform this technically correct (without

tension and concomitant behaviours). The parent(s) may need a separate parent session to learn this as some don't want to practise this in front of their child. A parent session can be organised for one parent or for a group of parents of PCWS who receive the Mini-KIDS (parents of PCWS who receive Mini-KIDS but do not participate in the TreatPaCS trial can also be included in those parent sessions; parents who received the LP or SCBT **cannot** attend these sessions).

One parent and the PCWS attend the treatment sessions. During this stage, it is important that parents alternate when they both want to learn to pseudo-stutter (which is preferred). The PCWS' and the parents' experiences and emotions of using the pseudo-stuttering are gradually openly named and discussed. Tangible tokens can be used to visualise the pseudo-stuttering, such as a jumping frog ("co-co-come here") or a snake ("sssssssson you can play").

Also the SLT and eventually the PCWS will use pseudo-stuttering.

The aim is to desensitise parent(s) and PCWS for stuttering, to make stuttering a topic for discussion (no taboo) and to make sure parents use the pseudo-stuttering. The PCWS daring to pseudo-stutter is a prerequisite for identification (Stage 2).

It (often) happens that because of the desensitisation, the stuttering has recovered, frequency drops are observed or only short and easy moments of stuttering remain. In this case there is no identification (Stage 2) and no modification phase (Stage 3). CWS and parents move on to phase 4 (generalization).

9.2.3 Stage 2 = Identification

This stage is only for children of ≥ 4 years as it requires sufficient cognitive and auditory skills. The PCWS and the parent(s) identify and discriminate the relevant types of stuttering and the relevant qualities during games with gradual increasing linguistic level. First in the pseudo-stuttering of the SLT, later also in the pseudo-stuttering of the parent and PCWS. In the end, the PCWS identifies real moments of stuttering in his own speech. It is taken care of that there is no new 'sensitisation' (i.e., an increased awareness linked to negative feelings such as concern).

Only relevant types and qualities of stuttering are aimed for: For example, if only repetitions and prolongations are present in the speech of the PCWS and no blocks, only repetitions and prolongations will be identified as type. Once the PCWS can identify and discriminate these types, the quality that hinders communication (duration or tension or both) is introduced as well. Again, tangible tokens facilitate this process (a hard snake and a soft snake for tensed versus easy loose prolongation; long and short snake for prolongation with long duration versus short duration).

The PCWS is ready to proceed to Stage 3 if s/he identifies and discriminates relevant types and qualities of real stuttering in his/her own speech. This does not have to be 100% correct and mainly on long and tensed moments of stuttering.

The parent(s) is ready to move to Stage 3 if s/he easily produces each type of stuttering behaviour in pseudo-stuttering with a positive attitude and discriminate these adequately in the speech of the PCWS.

For the PCWS < 4 years, only the parent(s) learns to identify and discriminate in individual sessions or in a parent group. In this age group, the parent(s) is ready to move to Stage 3 (modification) if s/he easily produces each type of stuttering behaviour in pseudo-stuttering with a positive attitude and discriminate them adequately in the speech of the PCWS.

If by now the stuttering has recovered or only short and easy moments of stuttering remain with declining frequency, there is no modification phase (Stage 3) and CWS and parents move on to phase 4 (generalization).

9.2.4 Stage 3 = Modification

For PCWS < 4 years:

The parent(s) models easy ongoing pseudo-stuttering in his/her speech. The modification of the stuttering moments occurs gradually and spontaneously. Stuttering moments become less tense or shorter. In some PCWS, this is only possible if the SLT and parent(s) show them how to loosen tension via pseudo-stuttering, but that is not necessary for all PCWS.

For PCWS ≥ 4 years:

The knowledge and skills of the identification phase (Stage 2) are used here to try to alter a moment of stuttering that hinders the PCWS. The PCWS is in command of this, the parent(s) does not ask for modification. It is advised that here, the PCWS teaches this to his/her parent(s), so s/he feels in control. First this is trained with pseudo-stuttering. Again, tangible items can be used to visualise the features of the stuttering behaviour such as a soft toy. The SLT and the parent(s) experiment with their own speech (model soft and hard, long and short, easy and difficult stuttering moments).

Gradually, the PCWS will apply this to real moments of stuttering. The parent(s) gradually withdraws and leaves the PCWS in charge.

Goals of Stage 3 are achieved if gradually more easy, short moments of stuttering, frequency drops in stuttering moments or recovery of stuttering is observed in the PCWS' speech.

9.2.5 Stage 4 = Generalisation

Parent counseling is the most frequently used technique during the Generalisation phase. The SLT seeks for topics that the parent(s) is still unsure about or for topics that need more information or training. The parent(s) evolves to an independent speech model for his/her PCWS.

Recovery or low frequency (near zero) and no reactive behaviour over a long period is the goal of Stage 4. The parent(s) and PCWS report that they feel competent and successful to deal with fluctuations in the left-over stuttering, if any.

9.2.6 Maintenance phase

The maintenance phase starts when the PCWS and parent(s) achieved the goals of Stage 4. Usually the stuttering is reduced to (near) zero stuttering. Treatment sessions to monitor if achieved goals are maintained are scheduled with an interval of 2, 2, 4, 4, 8, 8 and 16 weeks. If necessary, SLTs add extra sessions during maintenance, for example if the goals were not maintained.

9.3 Trial procedures in the LP

All sessions in Stage 1 take 45 to 60 minutes according to the LP treatment guide (Onslow et al., 2021). Sessions of 45 to 60 minutes with parent and PCWS, however, are not reimbursed through the Belgian health care system, only sessions of 30 minutes. Therefore, in this study, one hour sessions were limited to the first six treatment sessions. These sessions are scheduled with one PCWS and his/her parent(s) alone (not in groups). The parent receives education about stuttering and is coached to implement the treatment techniques.

9.3.1 Stage 1

Stage 1 aims at reducing the stuttering of the PCWS to (near) zero levels of stuttering (Onslow et al., 2021). During stage 1, parents learn to provide verbal contingencies during daily, 10-minute practice sessions with their PCWS. For unambiguous stutter-free speech, parents can praise, acknowledge the stutter-free speech or formulate a request for self-evaluation to the PCWS. The practice sessions that the parent(s) implement a home aim to give the PCWS the opportunity to practise stutter-free speech. Therefore, activities are specifically chosen and need to be fun for the PCWS. When the verbal contingencies for stutter-free are provided appropriately and effectively, the verbal contingencies for stuttering are also introduced. These are an acknowledgement of the stuttering moment or a request for self-correction. If a PCWS fails to

correct himself, no further verbal contingency on the speech is given (but a praise for the try can be provided).

During Stage 1, treatment sessions are scheduled weekly. The sessions begin with a conversation between the parent (or the SLT) and the PCWS so that the SLT can record a SR (this can also be made based on a recording of the PCWS conversing during everyday life). The SLT then discusses the daily use of the SR-scale with the parent. They focus on stuttering severity and treatment responsiveness during the past week. The parent(s) then demonstrates to the SLT how verbal contingencies were given to the PCWS during the previous week (this can also be made based on a recording). The SLT determines if the parent(s) delivers them in an appropriate way and discusses them with the parent(s). If applicable, they discuss changes to the procedure for the coming week and the SLT models the new (or changed) procedure during an interaction with the PCWS. Then, the parent(s) demonstrates his/her delivering of the new (modified) verbal contingencies and the SLT gives feedback to the parent(s). The SLT concludes the session with a summary of the procedures to be applied for the coming week and discusses them with the parent(s).

Later on, the practice sessions, that the parent(s) implement at home with the PCWS, become more natural and resemble everyday conversations. Also later on in the treatment, a limited number of verbal contingencies are given throughout the day. When the PCWS has typical scores of 0 and 1 on the SR-scale with more 0s than 1s per week for three consecutive weeks, and the stuttering is rated as 0 or 1 during the treatment session, the PCWS can proceed to stage 2.

9.3.2 Stage 2 = Maintenance phase

The maintenance phase starts when the stuttering is reduced to (near) zero stuttering and parents are able to implement the practice sessions when it is necessary (when stuttering relapses). Treatment sessions to monitor if achieved goals are maintained are scheduled with an interval of 2, 2, 4, 4, 8, 8 and 16 weeks. If necessary, SLTs add extra sessions during maintenance, for example if the goals were not maintained.

9.4 Assessment of compliance

The SLT will check compliance with a parent compliance checklist that will be generated for each treatment arm (SLT site file). An example can be found in appendix 9.

10 SAFETY RECORDING AND REPORTING

10.1 Definitions

Adverse events in this trial are unlikely to occur but may include a significant increase (e.g., ≥5%SS) of the stuttering compared to baseline measures or a long-term lack of reduction of moderate or severe stuttering (e.g., for 10 weeks).

Serious adverse events, such as the death of a PCWS, are extremely unlikely to be linked to this TreatPaCS as the treatment approaches have no life-threatening consequences.

10.2 Recording of safety findings in function of the available evidence

10.2.1 The case of types of intervention that are in routine clinical use

Increased stuttering indicates that the treatment is not effective. It may harm a PCWS to stutter for a significant amount of time with increased stuttering compared to the baseline stuttering severity (e.g., ≥5%SS versus the %SS measured by the blinded video analysts in the video recordings of the baseline session) or to have no reduction of the moderate or severe stuttering compared to the baseline session (e.g., over a period of 10 weeks). As a PCWS's awareness about stuttering increases with age, the opportunity to receive treatment that can decrease stuttering successfully decreases with age and a PCWS may develop feelings of anxiety associated with the stuttering if the stuttering persists. Therefore, it is necessary to act when an adverse event is reported.

Mini-KIDS and SCBT are the experimental interventions in this trial. Even though they are not evidence-based as a treatment in their whole, they are practise-based given they are routinely delivered in daily practice. The techniques and procedures that are used in both treatments are evidence-based on their own.Therefore, possible adverse events are known and are limited to the ones listed previously.

10.2.2 General considerations for the recording of safety findings

Adverse events need to be reported in REDCap by the SLT at any time during the trial (also in between the data collection points). In case an adverse event occurs, the Trial Management Group will decide what type of action needs to be taken. Action can include individualising the treatment approach while still complying to the necessary requirements of the protocol, leaving the allocated treatment arm, or leaving the study. The Co-CI will follow up with the SLT on the decided action.

10.3 Expedited reporting of Adverse Events

It is important that the treatment approaches affect the stuttering and the response to the stuttering of the PCWS (and family). A significant increase of the stuttering (e.g., ≥5%SS) compared to baseline measures or a lack of stuttering reduction of moderate or severe stuttering (e.g., for 10 weeks) are considered to negatively affect the PCWS and his/her family. When an adverse event is reported in REDCap, the Trial Management Group will take a decision about the action that is necessary to take.

Depending on when in the treatment process the adverse event is reported, and depending on the treatment arm, various actions may need to be taken. These may include adjusting to metacognitive skills, checking for desensitisation in the parents, increasing the number of practice sessions or the number of verbal contingencies throughout natural conversations, helping the child manage his emotions or training on increasing the PCWS's speaking skills.

These actions are followed up by the Co-Cis for the next 6 weeks. If these actions are not sufficient to decrease the stuttering further, the Trial Management Group will decide on further action. These may include further individualising of the program, leaving the allocated treatment arm or leaving the trial.

10.4 Responsibilities

The SLT checks for unexpected increases of stuttering or long-term plateauing of severe or moderate stuttering when PCWS attend for treatment and/or maintenance. They ensure these events are recorded in REDCap.

The Trial Statistician reviews periodically unblinded overall safety data to determine patterns and trends of events, or to identify safety issues, which are not apparent on an individual case basis.

The CI has a clinical overview of the safety of the PCWS participating in the trial, including an ongoing review of the risk/benefit.

The Trial Management Group collects data centrally and evaluates the adverse event according to the trial protocol. The group takes a decision for action to mitigate the adverse event.

The Co-Cis follow up with the SLTs on the actions that need to be taken and report back to the Trial Management Group meeting.

10.5 Notification of deaths

Deaths will be notified to the Ethics Committee. However, these are extremely unlikely to be linked to this trial.

10.6 Reporting urgent safety measures

If any urgent safety measures are taken, Thomas More Mechelen-Antwerpen will immediately and in any event no later than 3 days from the date the measures are taken give written notice to KCE and the relevant EC of the measures taken and the circumstances giving rise to those measures.

10.7 The type and duration of the follow-up of subjects after adverse events

The decision can be (1) individualise the treatment to the PCWS's needs (2) leave the allocated treatment arm or (3) discontinuation of TreatPaCS. The first action can include a variety of measurements. The duration of the follow-up depends on the action that was taken and will be specified by the Trial Management Group but takes at least 6 weeks.

11 STATISTICS AND DATA ANALYSIS

Thomas More Mechelen-Antwerpen assigned the statistical analysis to CTC UZ-ANTWERPEN.

The details of the statistical analysis will be described in a separate statistical analysis plan which will be finalised before data base lock.

11.1 Sample size calculation

In this trial the aim is to prove non-inferiority of Mini-KIDS compared to LP and SCBT compared to LP. The primary outcome is %SS at 18 months post-randomisation.
The non-inferiority margin was set at 1.0% SS following Bridgeman et al. (2016) and Donaghy et al. (2020). Several studies report a standard deviation on %SS at 18 months varying from 0.4 to 2.1 in different number of children (Arnott et al. 2014; Bridgeman et al. 2016; Donaghy et al. 2020; De Sonneville-Koedoot et al. 2015). Pooling all these results (weighing them with the number of children per study) we estimated a value of 1.7 for the standard deviation at 18 months on %SS. We adjust the usual one-sided significance level of 2.5% to 1.25% because we are interested in the two comparisons (Mini-KIDS versus LP and SCBT versus LP).

Assuming that in reality there is no difference between the three programs and using a power of 90% and a one-sided significance level of 1.25% we need 73 children in each group to show non-inferiority. Taking into account a 11% dropout (de Sonneville-Koedoot et al. 2015) we need to randomise 249 children in total (83 children per arm).

11.2 Planned recruitment rate

The number of recruiting sites is 30, divided over the French-speaking and Dutch-speaking part of Belgium. Sample size per site differs and depends on number of the SLTs' active clinical hours in the treatment of stuttering and the diversity of the caseload (SLT treats only stuttering or also other disorders). We know most of the participating sites and know that nearly all (≥95%) of PCWS will be eligible for this trial.

We estimate, based on the survey that we sent to the sites that showed an interest to participate, that they will have maximum one PCWS per month eligible. We expect that about 2/3 will participate. Assuming that on average 1.5 PCWS per 3 months per site give consent (more specifically at least one of their parents), are eligible and willing to participate and assuming a recruitment period of 18 months, we will be able to include 249 participating PCWS.

11.3 Statistical analysis plan

11.3.1 Summary of baseline data and flow of patients

- Compare demographics like age, gender, comorbidities (Autism Spectrum Disorder, ADHD, language disorders…) and parent profile between the three programs. Means and standard deviation per group will be reported for age. For gender and comorbidities numbers and percentages are reported.
- Compare the stuttering state at baseline between the three programs using stuttering severity score (%SS) and the stuttering onset questionnaire. For the continuous variables means and standard deviations are reported (in case of skewness median and interquartile range are reported). For the categorical variable numbers and percentages are reported.
- Comparing the different questionnaires at baseline between the three programs (KiddyCat, EQ-5D-Y proxy 1, CBQ-short version, parent report about PCWS's stuttering severity and about their satisfaction of the PCWS's communication in everyday speaking situations). Means and standard deviations are reported (or median and interquartile range as appropriate). For the answers to the ISPP-questionnaire, frequencies and percentages are reported per program.

- A consort flow diagram will be produced to get an overview of the number of patients available at each stage: eligibility, randomisation, allocation, discontinuation and follow-up.

11.3.2 Primary outcome analysis

The %SS at 18 months post-randomisation will be determined in two video samples (at home and in the treatment session) and the average will be used.

Considering this is a non-inferiority trial, the primary endpoint is analysed in first instance in the per-protocol population. The per-protocol population are all eligible children who followed the treatment for at least 80%. To establish this, the SLT will carry out a compliance and fidelity checklist for each treatment session (Appendix 9). To correct for the fact that the per-protocol population is a subset of the randomised population we use a weighted linear regression model with %SS at 18 months post-randomisation as outcome and program as predictor and weighting individuals with inverse probability weighting. The weights which are determined in the full dataset of adherers and non-adherers are estimated as an individual's probability to adhere to a certain program given observed confounders like gender, age at assessment, comorbidities, family history of stuttering and recovery of stuttering, parent profile, baseline stuttering severity assessment (%SS and SR), and temperament (CBQ-short version). Post-hoc comparison of SCBT compared to LP and Mini-KIDS to LP using a two-sided 97.5% (correction for two comparisons) confidence interval for the difference in %SS will be compared to the non-inferiority margin.

11.3.3 Secondary outcome analysis

- Different sensitivity analysis will be done to evaluate the differences between the programs at 18 months post-randomisation. A linear regression model with %SS as outcome and type of program as a predictor will be considered with inclusion of other factors like gender, age at assessment, comorbidities, family history of stuttering and recovery of stuttering, baseline stuttering severity assessment (%SS and SR), and temperament (CBQ-short version). Linear regression assumptions will be checked and if needed transformations or other models will be explored. This analysis will be done in the per-protocol (naïve per-protocol) as well as in the intention-to-treat population. For the intention-to-treat population children who drop out from the study get the last recorded %SS carried forward.
- In the secondary analysis we will also consider the comparison between Mini-KIDS and SCBT.
- As %SS is measured repeatedly (3, 6, 9, 12, 18, 24, 60 months post-randomisation) we can consider a linear mixed model with child as random intercept to compare the evolution over time between the programs and to get an even more precise estimate of the difference at 18 months post-randomisation. The linear mixed model also allows the inclusion of confounders and also for this

model assumptions will be checked and if needed revised. This analysis will be done in the per-protocol as well as in the intention-to-treat population.

- SR (also averaged over home and treatment session sample), score on the KiddyCAT and EQ-5D-Y-proxy 1 at 18 months post-randomisation will be compared between the three programs using an analysis of variance and answers to the ISPP-questionnaire using a Chi-square test. These outcomes will also be studied in a multiple linear regression model (with confounders as mentioned before) with adjustment for their baseline values. The same analysis will be done for the EQ-5D-Y-proxy 1 scores at the long-term follow-up at 2 years and 5 years post-randomisation.
- For the continuous measures SR, EQ-5D-Y proxy 1, measured at different time points we can compare the evolution over time between the programs using a linear mixed model. This model also allows to look at differences at a specific time point in a post-hoc comparison. For the answers to the ISPP-questionnaire measured at different time points this can be done using a generalized linear mixed model. For the ordinal outcomes parent report about their PCWS's stuttering severity and their satisfaction about the PCWS's communication in everyday speaking situations depending on variability a linear mixed or generalized linear mixed model will be used.
- The number of weeks, the number of treatment sessions, the treatment time needed until the maintenance phase begins will be compared between the three programs using an ANOVA or Kruskal Wallis test as appropriate. This outcome will also be studied in a multiple linear regression model adjusting for possible confounders.
- We will look at the proportion of children that are successful (defined as <1 %SS and SR ≤ 1) at 18 months and at 5 years post-randomisation. They will be compared by a Chi-square test (or Fisher's exact test as appropriate) between the three programs. The time to reach success between the three programs will be compared with a Kaplan-Meier plot and analyzed using a Cox regression model with correction for confounders.
- %SS and SR at 18 months post-randomisation will be evaluated separately for the videos at home and for the videos recorded during the treatment sessions using linear regression models as described for the averages of the two samples.
- For the above analysis we will first consider the intention-to-treat population to preserve randomization, if necessary also per-protocol population will be considered.
- Safety analysis will be done in all patients that are randomised.
- Numbers of children who drop out and reasons for drop out, as well as leave the allocated treatment arm (numbers, reason and switched treatment) will be described per treatment. Numbers per program will be compared using a Chi-square test (or Fisher's exact test as appropriate). A time to drop-out analysis or a time to switch analysis will be performed in case > 10% drop-out or a 10% switch was present in the trial.

11.3.4 Procedure(s) to account for missing or spurious data

Most of the data is routinely collected as standard of care and the SLT will be in contact with the PCWS regularly to ensure collection of all relevant data.

The SLT follows up on the missing data and records any reasons for missing data in PCWS' speech-language therapy files and the eCRF (REDCap).

For the sensitivity analysis of the primary outcome, we considered last observation carried forward, as an alternative we will also explore multiple imputation techniques and compare results.

The proposed linear mixed model allows that subjects have missing values at certain time points as the model uses all available data points per subject. The missing value assumption of the model is Missing At Random which means that missing values can only be dependent on the observed responses which seems a reasonable assumption in this case.

11.3.5 Other statistical considerations.

We will explore if we can find a profile of children more successful to one of the programs, possible factors that will be studied are gender, co-morbidities, age at assessment, family history of stuttering, family history of recovery of stuttering, onset data (gradual/sudden, time since onset, progress since onset), answers to the ISPP-questionnaire, baseline stuttering severity assessment (%SS and SR), parent profile and temperament (CBQ-short version). This will be studied in a linear regression model with %SS at 18 months as outcome and in a logistic regression model with success defined as <1 %SS and SR ≤ 1 as a binary outcome.
We will explore the agreement between the video samples recorded at home and those recorded during the sessions for %SS and SR.

11.4 Data collection for economic evaluation

For the disorder of stuttering, 128 sessions of 30 minutes are being reimbursed by RIZIV-INAMI within a period of 2 years. For preschool age children, only sessions of 30 minutes are reimbursed. Of these 128 sessions, 10 can be provided to parents without the child, as sessions of 60 minutes. If treatment is finalised before using 128 sessions and the child relapses within 6 months to 2 years after treatment, which is common for this disorder, 38 sessions of 30 minutes can be provided within a period of 1 year. That is, until the 128 sessions are used.
These sessions are used for treatment and parent coaching. Of these sessions, the cost for RIZIV/INAMI and for the patient is calculated on the condition that the stuttering specialist is conventioned

(“geconventioneerd”, “met overeenkomst”) and that the patient does not have a preference scheme (“zonder voorkeursregeling”).

Important note: Independently of the results of this trial, we do not question the number of reimbursed treatment sessions in the existing reimbursement scheme. Older children and adults who stutter need more treatment over a long time. Also, stuttering is known to be a relapse-prone disorder.

	Total Time (in Assessment + Treatment of 30 minutes)*	Total cost (€)	Total R/I (€)	Total P (€)
LP Cost	47	1264.7	1000.2	264.5
Mini-KIDS	61	1642.6	1299.1	343.5
SCBT	94	2103.9	1660.9	443.0

* Assessment time is not included in the 128 sessions

The calculations differ with previous calculations as we adapted the calculations to the set-up of the trial and added the data from the file audit (Mini-KIDS) and the most recent meta-analysis (Park et al., 2021). The calculations are based on assessment, six 60-minute treatment sessions and then 30-minute sessions.

Treating stuttering at preschool age is more successful, but also, once stuttering evolves to persisting stuttering, it can have a significant impact on the social activities of the person who stutters. It is known that adults who stutter have a 7-fold risk to developing anxiety disorders (Iverach et al., 2009). Hence, a person who (persistently) stutters costs the health system and the patient much more, given that psychological treatment may be necessary besides speech therapy.

11.4.1 Burden

The number of patients affected by the intervention under study per year in Belgium is calculated based on incidence numbers in the literature. Between 8% to 11.2% of children aged up to 4 years start to stutter (Reilly et al., 2013; Yairi & Ambrose, 2013). About 75% from them recover with or without treatment in the first years after stuttering onset (Yairi & Ambrose, 1999). Reilly et al. (2013), Yairi and Ambrose (1999) and recently Carey et al. (2020), however, reported only 6%, to 9% recovery during the first year after stuttering onset, about half of them with the help from a stuttering specialist. Given the lack of concrete data, we need to estimate, based on numbers from the literature. We estimate that about one third of the preschool

age children who start to stutter, seek treatment. That is, 2.6% - 3.7% of children between 2.5 and 6.5 years of age.

The number of interventions performed per year in Belgium is estimated as to our knowledge, there is no access to data about the number of interventions for stuttering with preschool age children in Belgium. In Flanders (Statistiek Vlaanderen, 2020), 263 517 children attend preschool; In Wallonia and Brussels, 187 791 (La FWB en chiffres, 2018, p. 124). Based on our own calculations, we estimate that about 45 130 children start to stutter, and about 1354 children of these children seek treatment for stuttering (10% incidence, 3% seeking treatment).

11.4.2 Potential effects on health

Potential effects on health from the intervention on the QOL-measures (EQ-5D-5L proxy 1) are expected to be visible in increased QOL-scores due to increased fluency. In this QOL-instrument, the subscales of "Doing usual activities" and "Feeling worried, sad or unhappy" are the ones of interest.

The estimated outcome in the comparator arm (LP) is "no problems doing the usual activities" and "being not worried, sad or unhappy" in 95% of the children 2 years post-randomisation and in 85% of the children 5 years post-randomisation. The estimated outcome in the experimental treatment arms (Mini-KIDS and SCBT) is also "no problems doing the usual activities" and "not worried, sad or unhappy" in 95% of the children 2 years post-randomisation and in 85% of the children 5 years post-randomisation.

The estimated outcome in the comparator arm (LP) is (near) zero stuttering levels that are maintained in 80% of the children at 2 and 5 years post-randomisation. The estimated outcome in the experimental treatment arms (Mini-KIDS and SCBT) is also (near) zero stuttering levels that are maintained in 80% of the children at 2 and 5 years post-randomisation.

11.4.3 Potential effect on costs

The estimate of the potential effects on costs (EUR) associated with the experimental treatment arms and the comparator arm is expected to be explicit. The potential impact on costs of the experimental treatment arms per patient in comparison to the comparator arm is an expectation of 1410 hours for the LP, which is 23% less treatment hours than for Mini-KIDS (1830 hours) and 50% less than for SCBT (2820 hours). This reduction of treatment hours results in a significant reduction of costs (see table above).

If the trial generates evidence that treatment with the LP, SCBT and Mini-KIDS requires the expected treatment time as mentioned previously, direct stuttering treatment (the LP and Mini-KIDS) consumes significantly less treatment hours than SCBT. If all stuttering specialists would switch to direct treatment (the LP or Mini-KIDS), we estimate that RIZIV/INAMI could save 447 280,38€ (LP vs SCBT), 244 938,60€ (Mini-KIDS vs SCBT) or 202 341,76€ (LP vs Mini-KIDS) per year (= absolute maximum gain). This is based on

N = 677 PCWS seeking treatment during preschool years (based on ½ of 1354 preschool age children who seek treatment for stuttering). This number is halved because it is calculated per year. Comparison with other scenarios, e. g., with costs for stuttering treatment of children whose stuttering was not timely treated during preschool age compared to PCWS who received stuttering treatment at preschool age, is not possible as such data are lacking.

The main economic consideration is the financial gain that can be achieved with direct treatment programs compared to SCBT for PCWS, especially with the LP. Savings can also be made by comparing direct treatment timely started with PCWS compared to the treatment needs of children whose stuttering developed into persisting stuttering because they did not receive treatment timely (during the preschool years, aiming at increased fluency).

The main health saving is that PCWS who are treated with the LP or Mini-KIDS are spared of long-life treatment for stuttering and stuttering-related issues such as social anxiety if treatment is initiated timely and focuses on increased fluency at preschool age.

It is expected that most relapse of stuttering will be treated successfully in the treatment approaches as they all aim for independent managing of relapse of stuttering by the parents. The treatment approaches are also expected to achieve similar outcomes regarding QOL and attitude towards speech.

It is however important to realise that stuttering will persist in a small number of children. When a patient is treated for stuttering at an older age, treatment is much more complicated and takes more time than treatment at a younger age, i.e. before the age of 6 years. We do not want to imply that the existing reimbursement scheme should decrease the number of reimbursed sessions. We think, if the trial shows that direct treatment, and especially the LP, is efficient and a more economical way of delivering the treatment, an extension to the reimbursement of 60-minutes sessions for children <10 years if parents are attending/participate in treatment would be beneficial.

If less treatment sessions are required and if they are grouped to one session per week, the burden upon the family of traveling to the stuttering practice and the risk on a negative impact on the parents' jobs and life will significantly decrease.

To evaluate the economic value of the trial (differences between the three treatment arms), no extra data are required. With the data that are provided in the trial, it is possible to calculate burden (based on number of treatment sessions) and cost of the treatment (based on number and length of sessions). In addition, the time spent implementing the program at home during the week will also be considered. Between the three treatment arms, travel time to the SLT is not expected to be different.

12 DATA HANDLING

12.1 Data collection tools and source document identification

Thomas More Mechelen-Antwerpen allocates the Data Management to CTC UZ-Antwerpen. CTC UZ-Antwerpen will develop and manage the data capturing system. All participating sites will use the eCRF system REDCap to collect the individual patient data required by the TreatPaCS protocol. There are three primary sources for the collection of trial data. For data collected during routine stuttering treatment sessions (clinical findings, observations), the PCWS' speech-language therapy file will be used as a primary source (paper-based or electronical). The eCRF system REDCap will be the primary source for questionnaire data collected electronically via the PCWS' parent's email address (EQ-5D-Y proxy 1, CBQ-short version, ISPP-questionnaire and the score for the child's stuttering severity and parent's satisfaction about the child's everyday communication) and reporting adverse events. SLTs also use REDCap to load data about the compliance and treatment fidelity for each treatment session. For parents of PCWS who were unable to complete the questionnaires electronically, the paper questionnaires will be the primary source. The eCRF data will be used to perform the statistical analyses for the trial, as described previously.

The CTC UZ-Antwerpen's data management team will design the eCRFs in REDCap, and will provide role-specific system usage training (data entry, data extraction, user accessibility ,…) to the Trial Management Group. CTC UZ-Antwerpen will provide data entry training sessions for the SLTs of the participating sites. The training sessions will be repeated if necessary (e.g. new version, delayed data entry, …). Thomas More Mechelen-Antwerpen designates the CI and the Trial Statistician to validate the designed eCRFs to ensure that the data points required for statistical analysis for the trial are included, and no redundant or secondary data is being collected. Validation of the designed eCRFs is done by means of a paper print with wet signature by both CI and Trial Statistician. In case of changes in the design of the eCRFs, the validation is repeated. Full audit trail of data collected is available in REDCap. The CI, PM, Data Manager, Field Monitors and auditors will receive access to the audit log in REDCap. Data extraction can be performed at any point during the trial by the designated people. Data tagged as personal information will be excluded from these data exports.

The eCRF system will not be used as a primary source of data, except for trial-related questionnaires that were completed electronically per email by the parent of the PCWS and to report adverse events.

A GDPR-proof platform will be used to collect and store the video recordings that are uploaded during the trial. SLTs will only be able to upload the video recordings of the PCWS at home, of the PCWS during the treatment session and of the entire treatment session for treatment fidelity. The videos will be stored on the platform for 7 years (01/04/2029). They will then be archived on an external drive.

More details on data handling can be found in the Data Management Plan.

12.2 Data handling and record keeping

All trial data will be collected in REDCap, a web-based eCRF system hosted by CTC UZ-Antwerpen on a virtual server located in the central facility at the Antwerp University Hospital. The software and updates are provided by Vanderbilt University and controlled by CTC UZ-Antwerpen. Vanderbilt University has no access to the virtual server or its data. REDCap complies with all applicable laws and regulations, including ICH E6 GCP, 21 CFR Part 11, EU Annex 11, General Data Protection Regulation (GDPR), HIPAA (US), ISO 9001 and ISO 27001.

TreatPaCS' data will be entered in REDCap's eCRFs. PCWSs will be pseudonymised by their unique Subject ID consisting of the site's unique number followed by an incremental number upon subject record creation in REDCap. In this multicenter study, this module will automatically generate the subject record ID based in the site. Example: if site A has site number 11 and site B has site number 12, then records will have the following subject IDs: Site A: 11-1, 11-2, 11-3,…; Site B: 12-1, 12-2, 12-3,… Sites are automatically allocated to a number when enabling this module, and this number cannot be edited.

Data is backed up daily, with a long-term backup (25 years) every month. This data is placed on a dedicated storage server at the CTC UZ-Antwerpen. Since the trial data will be stored at that location, no eCRF data storage needs to be foreseen with the participating sites.

Users have individual accounts and strong passwords are required. Access to data is determined by Thomas More Mechelen-Antwerpen and CTC UZ-Antwerpen and can be granted per person per institute, preventing unauthorized access to data by other researchers or institutes. Audit logs provide a fine-grained overview of data access and modifications.

The CI (Thomas More Mechelen-Antwerpen) and Data Manager (CTC UZ-Antwerpen) will be responsible for data entry and quality in collaboration with the site's SLTs. The CI (Thomas More Mechelen-Antwerpen) and Statistician (CTC UZ-Antwerpen) will be responsible for data analysis after monitoring has been concluded. The Data Manager (CTC UZ-Antwerpen) will overlook the process of randomisation and constructing the code table; the SLTs will be responsible for their code table.

12.3 Access to Data

Direct access will be granted upon written request to authorised representatives from the Belgian Healthcare Knowledge Centre (KCE), Thomas More Mechelen-Antwerpen and CTC UZ-Antwerpen to permit trial-related monitoring, audits and inspections.

12.4 Archiving

The trial database will be kept on a virtual server at the location of CTC UZ-Antwerpen for 25 years after database lock. Essential documents will be archived safely and securely according to applicable law and actual regulations to ensure that they are readily available upon authorities' request.

PCWS' speech-language therapy files will be archived according to local regulations and in accordance with the maximum period of time permitted by the SLT sites. Where the archiving procedures do not meet the minimum timelines required by Thomas More Mechelen-Antwerpen, alternative arrangements will be made to ensure the availability of the source documents for the required period.

The SLT at the participating sites notifies Thomas More Mechelen-Antwerpen if the archival arrangements change (e.g., relocation or transfer of ownership).

The SLT site file will be archived according the Belgian legislation and will not be destroyed without the approval of Thomas More Mechelen-Antwerpen.

The contracts with the participating SLTs will contain all regulations relevant for the study centre.

13 MONITORING, AUDIT & INSPECTION

Thomas More Mechelen-Antwerpen assigns the trial monitoring of TreatPaCS to CTC UZ-Antwerpen. A representative from the CTC UZ-Antwerpen will review the protocol and data capture requirements (i.e., eCRFs) with the SLTs during the site's initiation visit. During the study, Field Monitors employed by the CTC UZ-Antwerpen will employ several methods of ensuring protocol and GCP-compliance and the quality/integrity of the sites' data. The Field Monitor will visit the site to check the completeness of PCWS's speech-language therapy files, the accuracy of data capture and data entry, the adherence to the protocol and to GCP, and the progress of enrolment. SLTs must be on-hand to assist the Field Monitor during these visits. Continuous remote monitoring of each site's data may be performed by the centralised CTC UZ-Antwerpen's trial monitors.

The SLT must maintain source documents for each PCWS in the study, consisting of case and session notes (PCWS' speech-language therapy files) containing demographic and medical information, and the results of any other tests or assessments. Baseline information collected by the SLT (SSI-4 or TVS-NL/TSB-NL, KiddyCAT, Anamnesis form) on eCRFs must be traceable to these source documents in the PCWS's speech-language therapy file. The SLTs must also keep the original informed consent form signed by at least one parent of the PCWS (a signed copy is given to the parent(s) of the PCWS) and the signed information form for the PCWS.

The SLTs must give the Field Monitor access to all relevant source documents to confirm their consistency with the data capture and/or data entry. CTC UZ-Antwerpen's monitoring standards require full verification for the presence of informed consent form, adherence to the inclusion/exclusion criteria, documentation of adverse events, and of data that will be used for all primary variables. Additional checks of the consistency of the source data with the

eCRFs are performed according to a study-specific monitoring plan. This monitoring plan will be approved by the CI. The monitoring visits, including the site initiation visit and close out visit, will be performed at each site. The frequency of monitoring visits depends on the progress of enrolment and is determined in the monitoring plan. If there is no on-site monitoring visit, then a remote monitoring visit will be performed according to the monitoring plan. Any significant deviation from the planned monitoring timeline will be explained and documented in the monitoring report. If necessary, an amendment of the monitoring plan will be drawn up and approved again by the CI.

No regular monitoring visit will be planned for study sites that do not register PCWS or that stop enrolment. In the case of long-term absence (more than 4 months) of research activities, the monitor will ensure that the research team is adequately trained when the research activity is restarted.

14 ETHICAL AND REGULATORY CONSIDERATIONS

14.1 Ethics Committee (EC) review & reports

Before the start of the trial, approval will be sought from an EC for the trial protocol, informed consent forms, and other relevant documents (e.g., information brochures). Substantial amendments that require review by the EC will not be implemented until the EC grants a favourable opinion for the study before they can be implemented in practice at sites.

All correspondence with the EC will be retained in the Trial Master File and SLT site file. An annual progress report will be submitted to the EC within 30 days of the anniversary date on which the favourable opinion was given, and annually until the trial is declared ended. It is the responsibility of the CI to produce these annual progress reports.

The CI will also notify the EC of the end of the study. Should the study be ended prematurely, the CI will also notify the EC with a report also detailing the reasons for the premature termination.

Within one year after the end of the study, the CI will submit the final trial report with results including any publications/abstracts to the EC.

14.2 Peer review

The protocol was peer-reviewed independently, proportionately by three experts.

The three experts have expertise in coordinating multicentre trials in stuttering treatment for PCWS and have knowledge of the relevant discipline to consider the clinical and stuttering delivery service-based aspects of the protocol. Two of them are from Australia and are independent of Thomas More Mechelen-

Antwerpen and CTC UZ-Antwerpen. One expert lives in the Netherlands and is involved in the trial as a member of the Trial Steering Committee. They were nominated by Thomas More Mechelen-Antwerpen.

14.3 Public and Patient Involvement

14.3.1 Design of the research

A focus group was held to discuss the design of the study with six SLTs. During this focus group, the initial, general set-up was described. They formulated (1) doubts:

"*What if the treatment program does not provide the desired results. Will parents "blame" the outcome on being in the study?" - "It is a challenge not being able to follow your personal preference as stuttering specialist." - "What if not enough children can be recruited..."*

(2) and positive reflections:

"It is positive that the study does not include a control group consisting of children who don't receive treatment."

"The study may help making the choice between treatment programs in the future more easily." - "Expertise in treating stuttering with the [different] programs may have an impact on the study. It is great that workshops and guidance for the treatment programs are provided."

Parents of PCWS and were receiving treatment with Mini-KIDS (N = 3) at the time they were interviewed, with one parent who stuttered himself, formulated that

"A timely, profound approach for PCWS is essential. Great that researchers want to investigate that." - "I think that is a great idea. It is only positive to timely intervene to avoid problems in the long run. If this can help to support parents and children who stutter, I can only be in favour of the idea." - "Being able to offer several treatment programs enables adequate support for young children who stutter. Choosing the adequate treatment program will help to shorten the treatment period."

To redefine the primary outcome based on the suggestions from the Trial Board, two parents of PCWS (one who received treatment with the LP and one with Mini-KIDS) were contacted and asked for their opinion.

The patient representatives will review the final draft version of the protocol and provide the TreatPaCS' team with their feedback. Modification may be done based on their feedback.

14.3.2 Management of the research

Six patient representatives are included in the Trial Steering Committee for this trial: two adults who stutter, two parents of a PCWS and two SLT specialised in (early) stuttering.

We will involve our patient representatives to discuss the Risk Management Plan. The patient representatives will participate in the Trial Steering Committee meetings.

14.3.3 Undertaking the research

The patient representative from the Stuttering Association in Wallonia suggested to add a measure to evaluate the impact of stuttering on the PCWS and his/her parents (ISPP-questionnaire). The patient representatives gave input on the conduct of the trial but are not directly included in the conduct of the trial.

14.3.4 Analysis of results

No patient representatives are included in the analysis of the trial.

14.3.5 Dissemination of findings

The patient representative from the Stuttering Association will help disseminate the results. In addition, other stuttering groups will be contacted to help disseminate the results such as Belgian Interest Group of People who Stutter (BeST vzw), the Belgian Stuttering Organisation (B.S.V.) and Trust Your Ssstruggle.

14.4 Regulatory Compliance

The trial will be conducted in compliance with the approved protocol, the Declaration of Helsinki (2013), the principles of ICH-GCP. The trial will not begin until a Clinical Trial Authorisation is obtained from the EC.

The trial will be conducted in compliance with the Belgian law of May 7th 2004 regarding experiments on the human person and any relevant amendments.

14.5 Protocol compliance

The trial will be carried out in full compliance with the final version of the protocol. Waivers will only be allowed after written approval of the CI.

All protocol deviations that occur during the protocol which are related to a PCWS will be documented in the eCRF (REDCap). The eCRF deviation log will capture the deviation description, deviation type, deviation date, date identified, relation to adverse event. If a protocol deviation is related to more than one PCWS, this deviation must be recorded in the eCRF of each PCWS. General protocol deviations will be recorded

on a paper protocol deviation log and will be present in the SLT site file and Trial Master File. A protocol deviation with impact on the process of informed consent form and risk reporting will initiate a retraining by a Field Monitor during an on-site monitoring visit.

Other protocol deviations will be documented on the paper or eCRF log form. If the same protocol deviation re-appears three times within the same trial site, a protocol retraining (with special attention to the deviation process) will be performed by the Field Monitor during an on-site monitoring visit.

Deviations from the protocol that are found to recur frequently are not acceptable, will require immediate action, and could potentially be classified as a “serious breach”.

14.6 Notification of Serious Breaches to GCP and/or the protocol

A “serious breach” is a breach which is likely to effect to a significant degree:

- the safety or physical or mental integrity of the PCWS in the trial or
- the scientific value of the trial

Thomas More Mechelen-Antwerpen will be notified immediately of any case where the above definition applies during the trial conduct phase. Thomas More Mechelen-Antwerpen will notify the licensing authority (KCE, EC UZ-Antwerpen -UA and/or the GDPR commission if case the serious breach contains a data leak) in writing of any serious breach of the conditions and principles of GCP in connection with the trial or the protocol relating to the trial, as amended from time to time, within 7 days of becoming aware of that breach.

14.7 Data protection and patient confidentiality

Confidentiality will be maintained and Thomas More Mechelen-Antwerpen will ensure that the trial is conducted in compliance with the requirements of the Belgian and European Privacy legislation (https://www.dataprotectionauthority.be/citzen). All investigators and trial site staff must comply with the requirements of the above legislation on the protection of privacy in relation to the processing of personal data, with regards to collection, storage, processing, and disclosure of personal information.

Personal information collected at the trial site will be maintained and kept secure by the participating SLT. Data entered into REDCap will be coded and pseudonymised.

The pseudonymised data and key coding table will be kept in separate locations. The key coding table will be stored in an encrypted and password protected digital files. Access to these data will be limited to only those who need it for the purposes of the performance of the trial, quality control, audit, and analysis.

CTC UZ-Antwerpen will maintain the data as a data processor of Thomas More Mechelen-Antwerpen. Data will be managed subject to the terms of agreement between CTC UZ-Antwerpen and Thomas More Mechelen-Antwerpen and will be stored for 25 years after completion of the trial. CTC UZ-Antwerpen will only destroy the data after obtaining approval for destroying from Thomas More Mechelen-Antwerpen.

Videos may contain the child's first name, but it is highly unlikely that the entire identity of the PCWS is uncovered. To ascertain this, video recording instructions will include that the PCWS's full name should not be mentioned. Also, full names (first and last names) will never be mentioned between SLT and the research team (e.g., during the SLT-RCT committee meetings).

14.8 Financial and other competing interests for the Chief Investigator, SLTs at each site and committee members for the overall trial management

The CI has no financial interest in any of the interventions or products associated with the interventions in this trial. She has no commercial ties, nor do any family members of hers, and she does not receive financial compensation for any activities that are associated with this study.

The participating SLTs referred to in the governance documentation have also reported no conflict of interest related to this study. Should the site personnel change, then further information on conflicts of interest will be collected and documented in the governance documentation. A potential participating SLT with a relevant conflict of interest will not be accepted for trial participation.

14.9 Indemnity

In accordance with the Belgian law relating to experiments in humans dated May 7th 2004, Thomas More Mechelen-Antwerpen assumes the responsibility of any damages incurred by a PCWS and linked directly or indirectly to the participation to TreatPaCS. To provide compensation therefore Thomas More Mechelen-Antwerpen has undersigned a sufficient insurance policy with Ethias, policy n° 45.427.596.

Any participating SLT (whether or not through the participating site to which she is affiliated) has and maintains in full force and effect during the term of the study (and following termination of the study to cover any claims arising from the study) adequate insurance coverage for: (i) professional malpractice liability; and (ii) general liability; each such insurance coverage in amounts appropriate to the conduct of its tasks and responsibilities in the study.

All partners (participating SLTs and researchers) have knowledge of the joint and legal liability conform article 82 (https://www.privacy-regulation.eu/nl/artikel-82-recht-op-schadevergoeding-en-aansprakelijkheid-EU-AVG.htm).

14.10 Access to the Study Data by KCE and similar institutes in the EU

Upon submission of the first draft of the final report (as defined in the Research Agreement) and for a further period of six (6) years, and subject to this section, Thomas More Mechelen-Antwerpen will provide KCE upon KCE's request with the study data (as defined in the Research Agreement) in the format to be agreed between Thomas More Mechelen-Antwerpen and KCE. KCE may only access the study data for non-commercial health care research purposes and for health care purposes. The latter purpose is to be understood as the use with the aim to improve clinical practice and the health care system and/or to design, evaluate, and/or implement policies or programmes in connection with or related to health care, health economics, pharmaco-economics and/or social security. To avoid doubt, no access shall be provided to KCE with respect to patient health records (PCWS' speech-language therapy files).

Thomas More Mechelen-Antwerpen will ensure that the study data that are disclosed to KCE or to which KCE has otherwise access to upon submission of the first draft of the final report will only include pseudonymised personal data (as defined in the Research Agreement). Upon submission of the first draft of the final report, KCE will be given a copy (in a format to be agreed between Thomas More Mechelen-Antwerpen and KCE of the study data. The Research Agreement will require KCE to use such copy in compliance with all applicable personal data protection legislation. Thomas More Mechelen-Antwerpen will at all times ensure that (i) the unique code concerning such pseudonymised personal data will only be in the possession of the members of the TreatPaCS team who are in direct contact with the relevant data subjects (Co-Cis, CI and Trial Monitoring Assistants), (ii) such pseudonymised personal data can only be traced or linked back by the aforementioned study team members and (iii) the aforementioned study team members (as defined in the Research Agreement) will treat these codes as strictly confidential. In relation to the pseudonymised personal data to which KCE is granted access in accordance with the Research Agreement, KCE to comply as a separate and independent controller with all applicable personal data protection legislation. Notwithstanding the foregoing, Thomas More Mechelen-Antwerpen and KCE may, on an exceptional basis, mutually agree to disclose any pseudonymised personal data to KCE prior to the submission of the first draft of the final report.

Except if the procedure for publication as set forth in the Research Agreement has been followed, Thomas More Mechelen-Antwerpen will not provide (a copy of) the study data to a third party without the prior written approval of KCE.

All partners (KCE, participating SLTs and researchers) have knowledge of the joint and legal liability conform article 82 (https://www.privacy-regulation.eu/nl/artikel-82-recht-op-schadevergoeding-en-aansprakelijkheid-EU-AVG.htm). Thomas More Mechelen-Antwerpen and will only provide access to study data if the procedures are in accordance with article 82.

14.11 Access to the final trial dataset by other parties

14.11.1 Results Access Right

Thomas More Mechelen-Antwerpen will grant, and procures (where applicable) that any of its collaborator grant, to KCE and any other Belgian federal or regional institution, body, office, public service and/or agency at the end of the clinical trial, a non-exclusive, worldwide, irrevocable, unlimited, royalty-free and transferable access right to the results (both as defined in the Research Agreement), with the right to sub-license, for any non-commercial research purposes, public health care services purposes, and/or for designing, evaluating, and/or implementing policies or programmes in connection with or related to health care, health economics, pharmaco-economics and/or social security.

Upon request of KCE after the end of TreatPaCS, Thomas More Mechelen-Antwerpen will grant, and procures (where applicable) that any collaborator will grant, non-exclusive and royalty-free access rights to the results (as defined in the Research Agreement) to use such results, to EU or EU member state's institutions, bodies, offices, public services and/or agencies, for any non-commercial research purposes, public health care services purposes and/or for designing, evaluating and/or implementing policies or programmes in connection with or related to health care, health economics, pharmaco-economics and/or social security.

The foregoing access rights will include the right to publish, upon consultation with and after approval of Thomas More Mechelen-Antwerpen, any results for any non-commercial purpose, including any entry in a register of research findings or an individual issue of or a review article in a monograph series prepared on KCE's behalf. The content and timing for such publication will be subject to consultation with Thomas More Mechelen-Antwerpen and will take into account the publication timetables in other peer-reviewed journals and the need to make research findings publicly available as soon as practicable.

After the completion date (as defined in the Research Agreement), Thomas More Mechelen-Antwerpen will use its best efforts to grant and procures (where applicable) that any collaborator will grant access rights to the results to third parties on a non-exclusive basis and at fair and reasonable terms. If Thomas More Mechelen-Antwerpen and the relevant third party are unable to agree on the terms and conditions for the access to the results, Thomas More Mechelen-Antwerpen may request KCE to facilitate the discussions.

All parties explicitly agree that any access to study data will be construed in accordance with the section about access to the study data of the Research Agreement.

15 DISSEMINATION POLICY

15.1 Dissemination policy

General obligation. (a) Unless otherwise agreed between Thomas More Mechelen-Antwerpen and KCE, Thomas More Mechelen-Antwerpen will as soon as possible disseminate the results owned by it and/or any collaborator, by disclosing them to the public by appropriate means, including in scientific publications. Thomas More Mechelen-Antwerpen will inform and discuss its dissemination strategy with KCE in advance.

The final report will be made available for review and comment by KCE in accordance with the Research Agreement, before the results are disseminated.

Thomas More Mechelen-Antwerpen will notify KCE prior to any dissemination (including publication) (whether in oral, written or other form) (as defined in the Research Agreement) of the results or study data or of matters arising from TreatPaCS. Thomas More Mechelen-Antwerpen will send one draft copy of the proposed dissemination to KCE at least ten (10) calendar days for an abstract and thirty (30) calendar days for a manuscript before the date intended for dissemination. For the avoidance of doubt, Thomas More Mechelen-Antwerpen will continue to do so six (6) years after the end of TreatPaCS. KCE will have the right to object to such dissemination, by giving written notice to Thomas More Mechelen-Antwerpen (*a confidentiality notice*), to prevent the dissemination of KCE's confidential information (as defined in the Research Agreement), or to delay the proposed dissemination for a maximum of four months after the date of receipt of the *confidentiality notice* if, in its reasonable opinion, such delay is necessary in order to seek PCWS or similar protection for any results which are the subject of the intended dissemination. In the event Thomas More Mechelen-Antwerpen or any collaborator intends not to protect the results, it needs to formally notify KCE thereof before the dissemination takes place and parties will act as defined in the Research Agreement.

If such objection has been raised, Thomas More Mechelen-Antwerpen will discuss with KCE how to overcome the justified grounds for the objection on a timely basis (for example by adapting the planned publication and/or by protecting results before publication).

Thomas More Mechelen-Antwerpen will ensure that the manuscript for publication includes references to the data access plan, as well as the contact details of the person responsible within contractor for the management of third party access to the study data.

Thomas More Mechelen-Antwerpen will use its best efforts to ensure that its collaborators (KCE, participating SLTs and researchers) will not independently publish or otherwise disclose any findings resulting from TreatPaCS before publication of the main publication. In the event the TreatPaCS publication is not published within eighteen (18) months from the date the final report is accepted by KCE in accordance the Research Agreement, Thomas More Mechelen-Antwerpen and/or the collaborators will be entitled to publish the site-specific publication, subject to the procedure and conditions set forth in the Research Agreement.

Thomas More Mechelen-Antwerpen will ensure that any dissemination will acknowledge KCE's financial

support and carry a disclaimer.

Open access to scientific publications. Thomas More Mechelen-Antwerpen will ensure open access (free of charge, online access for any user) to all peer-reviewed scientific publications relating to the results owned by it and/or the collaborators. In particular it will (1) As soon as possible and at the latest on publication, deposit a machine readable electronic copy of the published version or final peer-reviewed manuscript accepted for publication in a repository for scientific publications; moreover Thomas More Mechelen-Antwerpen will aim to deposit at the same time the research data needed to validate the results presented in the deposited scientific publications; and (2) ensure open access to the deposited publication, via the repository at the latest on publication (if an electronic version is available for free via the publisher) or, within six (6) months of publication in any other case.

16 REFERENCES

Al-Khaledi, M., Lincoln, M., McCabe, P. & Alshatti, T. (2018). The Lidcombe Program: A series of case studies with Kuwaiti preschool children who stutter. Speech, Language and Hearing, 21, 224-235. https://doi.org/10.1080/2050571X.2017.1370523

Arias, J. L., & Diaz, A. L. (2010). Assessing self-regulated learning in early childhood education: Dififculties, needs, and prospects. *Psicothema*, *22*(2), 278-283

Arnott, S., Onslow, M., O'Brian, S., Packman, A., Jones, M., & Block, S. (2014). Group Lidcombe program treatment for early stuttering: A randomized controlled trial. *Journal of Speech, Language, and Hearing Research*, *57*(5), 1606-1618. https://doi.org/10.1044/2014_jslhr-s-13-0090

Baxter, S., Johnson, M., Blank, L., Cantrell, A., Brumfitt, S., Enderby, P., & Goyder, E. (2015). The state of the art in non- pharmacological interventions for developmental stuttering. Part 1: A systematic review of effectiveness. *International Journal of Language & Communication Disorders, 50*, 676–718. https://doi:10.1111/1460-6984.12171

Bergþórsdóttir, I. Ö., Crowe, K., & Einarsdóttir, J. T. (2021): Implementation fidelity in parent-implemented interventions for stuttering. Clinical Linguistics & Phonetics, 1-24. https://doi.org/10.1080/02699206.2021.1965659

Blomgren, M. (2013). Review of the successful stuttering management program. In S. J. Jaksic (Ed.), *The science and practice of stuttering treatment* (pp 99-113). John Wiley & Sons, Ltd (online library). https://doi.org/10.1002/9781118702796.ch8

Blood, G. W., & Blood, I. M. (2004). Bullying in adolescents who stutter: Communicative competence and self-esteem. *Contemporary Issues in Communication Science and Disorders, 31*, 69–79

Blood, G. W., & Blood, I. M. (2007). Preliminary study of self- reported experience of physical aggression and bullying of boys who stutter: Relation to increased anxiety. *Perceptual and Motor Skills, 104,* 1060–1066. https://doi.org/10.2466/pms.104.4.1060-1066

Boey, R. A., Van de Heyning, P. H., Wuyts, F. L., Heylen, L., Stoop, R., & De Bodt, M. S. (2009). Awareness and reactions of young stuttering children aged 2–7 years old towards their speech disfluency. *Journal of Communication Disorders, 42*, 334– 346. https//doi.org/10.1016/j.jcomdis.2009.03.002.

Bohland, J., Bullock, D., & Guenther, F. (2010). Neural representations and mechanisms for the performance of simple speech sequences. *Journal of Cognitive Neuroscience*, *22*(7), 1504-1529. https://doi.org/10.1162/jocn.2009.21306

Bonelli, P., Dixon, M., Ratner, N. B., & Onslow, M. (2000). Child and parent speech and language following the Lidcombe Programme of early stuttering intervention. *Clinical linguistics & phonetics, 14*(6), 427-446. https://doi.org/10.1080/026992000415868

Bothe, A. K., Davidow, J. H., Bramlett, R. E., & Ingham, R. J. (2006). Stuttering treatment research 1970–2005: I. Systematic review incorporating trial quality assessment of behavioral, cognitive, and related approaches. *American Journal of Speech-Language Pathology, 15*, 321–341. doi:10.1044/1058-0360(2008/009)

Bridgman, K., Onslow, M., O'Brian, S., Jones, M., & Block, S. (2016). Lidcombe program webcam treatment for early stuttering: A randomized controlled trial. *Journal of Speech, Language, and Hearing Research*, *59*(5), 932-939. https://doi.org/10.1044/2016_jslhr-s-15-0011

Brignell, A., Krahe, M., Downes, M., Kefalianos, E., Reilly, S., & Morgan, A. (2021). Interventions for children and adolescents who stutter: A systematic review, meta-analysis, and evidence map. *Journal of Fluency Disorders, 70*, 105843. https://doi.org/10.1016/j.jfludis.2021.105843

Davis, S., Howell, P., & Cooke, F. (2002). Sociodynamic relationships between children who stutter and their non-stuttering classmates. *Journal of Child Psychology, 43*(7), 939-947. https://doi.org/10.1111/1469-7610.00093

de Sonneville-Koedoot, C., Stolk, E., Rietveld, T., & Franken, M. C. (2015). Direct versus indirect treatment for preschool children who stutter: The RESTART randomizes trial. *Plos One, 10*(7), e0133758. https://doi.org/10.1371/journal.pone.0133758

Donaghy, M., Harrison, E., O'Brian, S., Menzies, R., Onslow, M., Packman, A., et al. (2015). An investigation of the role of parental request for self-correction of stuttering in the Lidcombe Program. *International Journal of Speech-Language Pathology, 17,* 511-517. https://doi.org/10.3109/17549507.2015.1016110

Donaghy, M., O'Brian, S., Onslow, M., Lowe, R., Jones, M., & Menzies, R. G. (2020). Verbal Contingencies in the Lidcombe Program: A Noninferiority Trial. *Journal of Speech, Language, and Hearing Research*, *63*(10), 3419-3431. https://doi.org/10.1044/2020_jslhr-20-00155

Druker, K., Hennessey, N., Mazzucchelli, T., & Beilby, J. (2019). Elevated attention deficit hyperactivity disorder symptoms in children who stutter. *Journal of fluency disorders*, *59*, 80-90. https://doi.org/10.1016/j.jfludis.2018.11.002

Eggers, K., & Van Eerdenbrugh, S. (2019). Inventarisatie van de inhoud en vorm van stottertherapie in Vlaanderen en Wallonië. *Logopedie, 32*(6), 30-40

EQ-5D-Y proxy 1 (2020). https://euroqol.org/eq-5d-instruments/eq-5d-y-available-modes-of-administration/

Femrell, L., Åvall, M., & Lindström, E. (2012). Two-year follow-up of the Lidcombe Program in ten Swedish-speaking children. *Folia Phoniatrica et Logopaedica*, *64*(5), 248-253. https://doi.org/10.1159/000342149

Ferdinands, B. & Bridgman, K. (2019). An investigation into the relationship between parent satisfaction and child fluency in the Lidcombe Program: Clinic versus telehealth delivery. International Journal of Speech-Language Pathology, 21, 347-354. https://doi.org/10.1080/17549507.2018.1445779

Galantucci, B., Fowler, C.A., & Turvey, M.T. (2006). The motor theory of speech perception reviewed. *Psychonomic Bulletin and Review*, 13 (3), 361-377

Gerlach, H., Totty, E., Subramanian, A., & Zebrowski, P. (2018). Stuttering and labor market outcomes in the United States. *Journal of Speech, Language, and Hearing Reserarch, 61*(7), 1649-1663. https://doi.org/10.1044/2018_jslhr-s-17-0353

Guenther, F. (2006). Cortical interactions underlying the production of speech sounds. *Journal of Communication Disorders*, 39, 350-365. https://doi.org.10.1016/j.jcomdis.2006.06.013

Guitar, B (2018). *Stuttering*. Philadelphia: Lippincott Wiliams & Williams

Hakim, H.B., & Bernstein Ratner, N. (2004). Nonword repetition abilities of children who stutter: an exploratory study. *Journal of Fluency Disorders*, 29, 179-199. https://doi.org/10.1016/j.jfludi.2004.06.001

Harris, V., Onslow, M., Packman, A., Harrison, E., & Menzies, R. (2002). An experimental investigation of the impact of the Lidcombe Program on early stuttering. *Journal of fluency disorders, 27*(3), 203-214. https://doi.org/10.1016/s0094-730x(02)0127-4

Hewat, Sally, Unicomb, Rachael, Dean, Imogen & Cui, Guangli. (2018). Treatment of childhood stuttering using the lidcombe program in mainland china: Case studies. *Speech, Language and Hearing, 23(*4), 221-231. https://doi.org/10.1080/2050571X.2018.1511106

Imeson, J., Lowe, R., Onslow, M., Munro, N., Heard, R., O'Brian, S., & Arnott, S. (2018). The Lidcombe Program and child language development: Long-term assessment. *Clin Linguist Phon., 32*(9),860-875. doi: 10.1080/02699206.2018.1448897.

Iverach, L., O'Brian, S., Jones, M., Block, S., Lincoln, M., Harrison, E., … Onslow, M. (2009). Prevalence of anxiety disorders among adults seeking speech therapy for stuttering. *Journal of Anxiety Disorders, 23*(7), 928-934. https://doi.org/10.1016/j.janxdis.2009.06.003

Johnson, K. N., Conture, E. G., & Walden, T. A. (2012). Efficacy of attention regulation in preschool-age children who stutter: A preliminary investigation. *Journal of Communication Disorders*, 45 (4), 263-278. https://doi.org/10/1016/j.jcomdis.2012.04.001

Jones, M., Onslow, M., Packman, A., Williams, S., Ormond, T., Schwarz, I., & Gebski, V. (2005). Randomised controlled trial of the Lidcombe program of early stuttering intervention. *British Medical Journal, 331*(7518), 659–661. doi:10.1136/ bmj.38520.451840.E0

Jones, M., Onslow, M., Packman, A., O'Brian, S., Hearne, A., Williams, S., Ormond, T., & Schwarz, I. (2008). Extended follow-up of a randomized controlled trial of the Lidcombe Program of Early Stuttering Intervention. *International Journal of Language and Communication Disorders, 43*(6), 649–661. https://doi.org/10.1080/13682820801895599

Karimi, H., Onslwo, M., Jones, M., O'Brian, S., Packman, A., Menzies, R., Reilly, S., Summer, M., & Jelčić-Jakšiće, S. (20&8). The Satisfaction with Communication in Everyday Speaking Situations (SCESS) scale: An overarching outcome measure of treatment effect. Journal of Fluency Disorders, 58, 77-85; https://doi.org/10.1016/j.jfludis2018.10.002

Karrass, J., Walden, T. A., Conture, E. G., Graham, C. G., Arnold, H. S., Hartfield, K. N., & Schwenk, K. A. (2006). Relation of emotional reactivity and regulation to childhood stuttering. *Journal of Communication Disorders*, *39*(402), 276-293. https://doi.org/10.1016/J.Jcomdis.2005.12.004

Kingston, M., Huber, A., Onslow, M., Jones, M., & Packman, A. (2003). Predicting treatment time with the Lidcombe Program: Replication and meta-analysis. *International Journal of Language and Communication Disorders, 38*(2), 165–177. https://doi.org/10.1080/1368282031000062882.

Koushik, S., Hewat, S., Shenker, R. C., Jones, M., & Onslow, M. (2011). North-American Lidcombe Program file audit: Replication and meta-analysis. *International journal of speech-language pathology*, *13*(4), 301-307. https://doi.org/10.3109/17549507.2011.538434

Koushik, S., Hewat, S., Onslow, M., Shenker, R., Jones, M., O'Brian, S., ... & Wilson, L. (2019). Three Lidcombe program clinic visit options: a phase II trial. *Journal of communication disorders*, *82*, 105919. https://doi.org/10.1016/j.jcomdis.2019.105919

La FWB en chiffres (2018). http://www.directionrecherche.cfwb.be/index.php?eID=tx_nawsecuredl&u=0&g=0&hash=295b2

a1d544e64e79cf11544e197cc0633481b21&file=fileadmin/sites/sr/upload/sr_super_editor/sr_editor/documents/statistiques/CC2018_web.pdf

Langevin, M., Packman, A., & Onslow, M. (2010). Parent perceptions of the impact of stuttering on their preschoolers and themselves. *Journal of communication disorders*, *43*(5), 407-423. https://doi.org/10.1016/j.jcomdis.2010.05.003

Langevin, M., Packman, A., & Onslow, M. (2009). Peer responses to stuttering in the preschool. *American Journal of Speech-Language Pathology, 18*(3), 264-276. https://doi.org/10.1044/1058-0360(2009/07-0087)

Lattermann, C., Shenker, R. C., & Thordardottir, E. (2005). Progression of language complexity during treatment with the Lidcombe Program for early stuttering intervention. *American Journal of Speech-Language Pathology, 14*(3), 242–253. https://doi.org/10.1044/1058-0360(2005/024)

Lattermann, C., Euler, H. A., & Neumann, K. (2008). A randomized control trial to investigate the impact of the Lidcombe Program on early stuttering in German-speaking preschoolers. *Journal of Fluency Disorders, 33*(1), 52-65. https://doi.org/10.1016/j.jfludis.2007.12.002

Leclercq, A. - L., & Kister, J. (2015). Stuttering in young children: Preliminary data on the implementation of the Lidcombe program in a French-speaking clinical context. A.N.A.E. *Approche Neuropsychologique des Apprentissages chez l'Enfant, 27*, 188-192. Retrieved from http://ovidsp.ovid.com/ovidweb.cgi?T=JS&PAGE=reference&D=psyc12&NEWS=N&AN=2015-53775-008.

Lewis, C., Packman, A., Onslow, M., Simpson, J. M., & Jones, M. (2008). A phase II trial of telehealth delivery of the Lidcombe Program of Early Stuttering Intervention. *American Journal of Speech-Language Pathology, 17*(2), 139–149. https://doi.org/10.1044/1058-0360(2008/014)

Guitar, Barry, Kazenski, Danra, Howard, Alan, Cousins, S. Freddie, Fader, Elena & Haskell, Piper. (2015). Predicting treatment time and long-term outcome of the Lidcombe Program: A replication and reanalysis. *American Journal of Speech-Language Pathology, 24,* 533-544. https://doi.org/10.1044/2015_AJSLP-13-0156

McCulloch, J., Swift, M. C., & Wagnitz, B. (2017). Case file audit of Lidcombe program outcomes in a student-led stuttering clinic. *International journal of speech-language pathology*, *19*(2), 165-173. https://doi.org/10.3109/17549507.2016.1159336

Mo, Y., Lim, C., Watson, J. A., White, N. J., & Cooper, B. S. (2020). Non-adherence in non-inferiority trials: pitfalls and recommendations. British Medical Journal, m2215. https://doi.org/10.1136/bmj.m2215

Neumann, K., Euler, H. A., Bosshardt, H., Sandrieser, P., & Sommer, M. (2017). The pathogenesis, assessment and treatment of speech fluency disorders. https://www.aerzteblatt.de/int/archive/article?id=189154

Nye, C., & Hahs-Vaughn, D. (2011). Assessing methodological quality of randomized and quasi-experimental trials: A summary of stuttering treatment research. *International Journal of Speech-Language Pathology, 13*(1), 49-60. https://doi.org/10.3109/17549507.2010.492873

Nye, C., Vanryckeghem, M., Schwartz, J. B., Herder, C., Turner, H. M., & Howard, C. (2013). Behavioral stuttering interventions for children and adolescents: A systematic review and meta-analysis. *Journal of Speech, Language, and Hearing Research, 56*(3), 921–932. https://doi.org/10.1044/1092-4388(2012/12- 0036)

Olander, L., Smith, A., & Zelaznik, H. (2010). Evidence that motor timing deficit is a factor in the development of stuttering*. Journal of Speech, Language and Hearing Research*, 53, 876-886. https://soi.org/10.1044/1092-4388(2009/09-0007)

Onslow, M., Webber, M., Harrison, E., Arnott, S., Bridgman, K., Carey, B., Sheedy, S., O'Brian, S., MacMillan, V., Lloyd, W., & Hearne, A. (2021). *The Lidcombe Program treatment guide* (version 1.3). Retrieved from https://www.uts.edu.au/sites/default/files/2021-04/Lidcombe%20Program%20Treatment%20Guide%202021%20v1.3%202021-04-27.pdf

Onslow, M. (2021). *Stuttering and its treatment: Eleven lectures*. Retrieved from https://www.uts.edu.au/asrc/resources

Onslow, M., Stocker, S., Packman, A., & McLeod, S. (2002). Speech timing in children after the Lidcombe Program of early stuttering intervention. *Clinical linguistics & phonetics, 16*(1), 21-33. https://doi.org/10.1080/02699200110092577

Onslow, M., Packman, A., & Harrison, E. (2003), *The Lidcombe Program of early stuttering intervention: A clinician's guide*. Austin TX: Pro-Ed

Onslow, M., Jones, M., Menzies, R., O'Brian, S., & Packman, A. (2012). Stuttering. In P. Sturney & M. Hersen (Eds.), *Handbook of evidence-based practice in clinical psychology: Vol 1. Child and adolescent disorders* (pp. 185-207). Wiley

Oonk, L. C., Koolhaas, C., Blom, S., Mooi, M. J.., Vriens-Bol, A. M., Busser, A. G., Franken, M. C., Tonnis, M. S. El Youssfi, Z., & Ormondt, J. (2020). Richtlijn Stotteren bij kinderen, adolescenten en volwassenen. https://www.nvlf.nl/

Packman, A., Onslow, M., & Attanasio, J. (2003). The timing of early intervention with the Lidcombe Program. In M. Onslow, A. Packman, & E. Harrison (Eds.), *The Lidcombe Program of early stuttering intervention: A clinician's guide* (pp. 41–55). Austin TX: Pro-Ed

Park, V., Onslow, M., Lowe, R., Jones, M., O'Brian, S., Packman, A., Menzies, R., Block, S., Wilson, L., Harrison, E., & Hewat, S. (2021). Predictors of Lidcombe Program treatment dropout and outcome for early stuttering. *Int J Lang Commun Disord*, 56(1), 102-115. doi: 10.1111/1460-6984.12586.

Pertijs, M. A., Oonk, L. C., de Beer, J. J., Bunschoten, E. M., Bast, E. J., van Ormondt, J., … van Veenendaal, H. (2014). Evidence-based Richtlijn Stotteren bij kinderen, adolescenten en volwassenen. Woerden: Nederlandse Vereniging voor Logopedie en Foniatrie

PlanKad Logopedisten (2016). https://overlegorganen.gezondheid.belgie.be/sites/default/files/documents/plankad_logopedisten_2016_nl_update.pdf

Prins, P. J., Bosch, J. D., & Braet, C. (2011). *Methoden en technieken van gedragstherapie bij kinderen en jeugdigen*. 2nd edition. Houten: Bohn Stafleu van Loghum.

Reilly, S., Onslow, M., Packman, A., Cini, E., Conway, L., Ukoumunne, O.C., ... Wake, M. (2013). Natural history of stuttering to 4 years of age: A prospective community-based study. *Pediatrics, 132*(3), 460–467. https://doi.org/10.1542/peds.2012-3067.

Riziv (2018). https://www.inami.fgov.be/nl/publicaties/jv2018/Paginas/default.aspx

Sandrieser, P., Schneider, P. (2015) Stottern im Kindesalter. Deutschland, Thieme Stuttgart

Seth, D. & Maruthy, S. (2018). Speech Therapy for children who stutter: A systematic review. In S. K. Gupta, & S. Venkatesan, (Eds.), *Handbook of research on psychosocial perspectives of human communication disorders* (pp. 321-344) doi.org/10.4018/978-1-5225-0.ch17

Shafiei, B., Faramarzi, S., Abedi, A., Dehqan, A., & Scherer, RC (2019). Effects of the Lidcombe Program and Parent-Child Interaction Therapy on Stuttering Reduction in Preschool Children. *Folia Phoniatr Logop.,71*(1),29-41. doi: 10.1159/000493915.

Statistiek Vlaanderen (2020). https://www.statistiekvlaanderen.be/nl/aanwezigheid-kleuters-in-kleuteronderwijs

Stes, R., & Boey, R. (1997). Detectie Instrument Stotteren. Antwerpen: CIOOS vzw

Swift, M. C., Jones, M., O'Brian, S., Onslow, M., Packman, A. & Menzies, R. (2016). Parent verbal contingencies during the Lidcombe Program: Observations and statistical modeling of the treatment process. *Journal of Fluency Disorders, 47*, 13-26. https://doi.org/10.1016/j.jfludis.2015.12.002

Trajkovski, N., O'Brian, S., Onslow, M., Packman, A., Lowe, R., Menzies, R., Jones, M., & Reilly, S. (2019). A three-arm randomized controlled trial of Lidcombe Program and Westmead Program early stuttering interventions. *J Fluency Disord., 61,* 105708. doi: 10.1016/j.jfludis.2019.105708.

Van Eerdenbrugh, S., Stuyvaert, V., & Eggers, K. (2020). Inventaire du contenu et des types de traitement du begaiement en Fédération Wallonie-Bruxelles et en Flandre. *UPLF info, 2020*(3),*16-28*

Van Eerdenbrugh, S., Ijterlinde, I., Eggers, K., & Franken, M. C. (in review). Which aspects help decide stuttering specialists in making the choice for treatment for preschool age children who stutter: a cross-sectional study

Van Eerdenbrugh, S., Packman, A., O'Brian, S. & Onslow, M. (2018). Challenges and strategies for speech-language pathologists using the Lidcombe program for early stuttering. *American Journal of Speech-Language Pathology, 27,* 1259-1272. https://doi.org/10.1044/2018_AJSLP-ODC11-17-0185

Van Eerdenbrugh, S., Packman, A., Onslow, M., O'brian, S. & Menzies, R. (2018). Development of an internet version of the Lidcombe Program of early stuttering intervention: A trial of Part 1. *International Journal of Speech-Language Pathology, 20,* 216-225. https://doi.org/10.1080/17549507.2016.1257653

Vanryckeghem, M., & Brutten, G. (2015). KiddyCAT: Communication Attitude Test voor stotterende kleuters. Gijzegem: Sig

Waelkens, V. (2018). *Mini-Kids, stottertherapie bij jonge stotterende kinderen (2-6).* Leuven: Acco

Webber, M. & Onslow, M. (2003). Mainentance of treatment effects. In *The Lidcombe Program of early stuttering intervention: A clinican's guide*, M. Onslow, A. Packman, & E. Harrison (pp. 81-90). Pro-Ed

World Health Organisation (2021). https://icd.who.int/browse11/l-m/en#/http://id.who.int/icd/entity/654956298

World Health Organisation (n.d.). Translation guidelines. WHODAS 2.0. https://terrance.who.int/mediacentre/data/WHODAS/Guidelines/WHODAS%202.0%20Translation%20guidelines.pdf

Yairi, E., & Ambrose, N.G. (1999). Early childhood stuttering I: Persistency and recovery rates. *Journal of Speech Language, Hearing and Research, 42*, 1097–1112. doi:10.1044/ jslhr.4205.1097

Yairi, E., Ambrose, N. G., Paden, E. P., & Throneburg, R. N. (1996). Predictive factors of persistence and recovery: Pathways of childhood stuttering. *Journal of Communication Disorders, 29*(1), 51–77. doi:10.1016/0021-9924(95)00051-8.

Yairi, E., & Ambrose, N. (2013). Epidemiology of stuttering: 21st century advances. *Journal of Fluency Disorders, 38*(2), 66-87. https://doi.org/10.1016/j.jfludis.2012.11.002

Yandeau, Elaine, Carey, Brenda & Onslow, Mark. (2021). The lidcombe program: A client report 7 years post-treatment. Speech, Language and Hearing, No Pagination Specified. https://doi.org/10.1080/2050571X.2021.1923304

■ APPENDICES

APPENDIX 1. RISK ASSESSMENT OF THE TRIAL INTERVENTION(S)

Risks associated with trial interventions
☒ A ≡ Comparable to the risk of standard medical care
☐ B ≡ Somewhat higher than the risk of standard medical care
☐ C ≡ Markedly higher than the risk of standard medical care

Potential risks include

(a) Participation in the study requires parents to video record their child at 7 points in time and to answer questions about their child and the child's speech. This may be experienced as a burden by the family.
(b) Even though the highest precautions are taken to keep the confidentiality of the PCWS and family, there is a risk that confidentiality of the PCWS/family is lapsed due to data collection (mainly in the video recordings).

**The key risks related to the treatment approaches in TreatPaCS**		**Activities to minimise the risks**		
Intervention	***Body system/Hazard***	***Action***	***Frequency***	***Comments***
Mini-KIDS<br>Lidcombe Program<br>SCBT	Participation to the study is experienced as a burden to the family	Questionnaires were kept as short as possible, tested out beforehand to estimate the time required; data collection points were kept to a minimum; only one video recording from the home situation is asked	Not specified	/
Mini-KIDS<br>Lidcombe Program<br>SCBT	Confidentiality PCWS/family	In the video instruction it was specifically asked that the full name (first and last name) should never be mentioned in the video recordings. If the video analyst recognises the name of a PCWS in the video because it was mentioned by the SLT during a SLT-RCT committee meeting, the video analyst will stop rating the video and will transfer the video to another video analyst who is blinded to the treatment.	Not specified	

The Trial Management Group will decide which actions need to be taken (individualise the treatment approach more within the protocol instructions, leaving the allocated treatment arm or leave the study) and for how long.

APPENDIX 2. PERSPECTIVES OF SLTS INVOLVED IN THE TREATPACS STUDY

Last name, first name:

Experience with therapy for preschool-age children who stutter (in years) :

Experience with the Lidcombe Program (in years) :

Experience with Mini-KIDS (in years) :

Experience with the SCBT (in years) :

Do you currently have a preference for any of the three treatment approaches? If so, can you elaborate on what currently explains this preference?

Response : ____

Concerning the Lidcombe program,

What are your current **expectations of the effectiveness** of the Lidcombe Program (effectiveness may relate to more than one objective, you may wish to detail/nuance)? Have these expectations changed since the beginning of TreatPaCS? If yes, please specify.

What are your current impressions of the **ease of implementation** of the Lidcombe Program? Have these impressions changed since the beginning of TreatPaCS? If so, please explain.

What is your current judgement of the **objectives and aspects worked** on by the Lidcombe Program? Has this judgement changed since the beginning of TreatPaCS? If so, please specify.

What are your current impressions of the **adaptability** of the Lidcombe Program to each child? Have these impressions changed since the beginning of TreatPaCS? If so, please explain.

What are your current impressions of the **investment required** of the parent and the SLT in the Lidcombe Program? Have these impressions changed since the beginning of TreatPaCS? If so, please explain.

Is there any aspect of the Lidcombe Program that you **particularly appreciate** at present? If yes, please specify. Have there been any changes in this area since the start of TreatPaCS?

Is there any aspect of the Lidcombe Program that you **currently dislike**? If yes, please specify. Have there been any changes in this area since the start of TreatPaCS?

What do you think of the **documents accompanying** the Lidcombe Program (manual, checklists, others)? Do you find them sufficiently helpful and detailed? Please specify.

Concerning the Mini-KIDS,

What are your current **expectations of the effectiveness** of Mini-KIDS (effectiveness may relate to more than one objective, you may wish to detail/nuance)? Have these expectations changed since the beginning of TreatPaCS? If yes, please specify.

What are your current impressions of the **ease of implementation** of Mini-KIDS? Have these impressions changed since the beginning of TreatPaCS? If so, please explain.

What is your current judgement of the **objectives and aspects worked** on by Mini-KIDS? Has this judgement changed since the beginning of TreatPaCS? If so, please specify.

What are your current impressions of the **adaptability** of Mini-KIDS to each child? Have these impressions changed since the beginning of TreatPaCS? If so, please explain.

What are your current impressions of the **investment required** of the parent and the SLT in Mini-KIDS? Have these impressions changed since the beginning of TreatPaCS? If so, please explain.

Is there any aspect of Mini-KIDS that you **particularly appreciate** at present? If yes, please specify. Have there been any changes in this area since the start of TreatPaCS?

Is there any aspect of Mini-KIDS that you **currently dislike**? If yes, please specify. Have there been any changes in this area since the start of TreatPaCS?

What do you think of the **documents accompanying** Mini-KIDS (manual, checklists, others)? Do you find them sufficiently helpful and detailed? Please specify.

Concerning the SCBT,

What are your current **expectations of the effectiveness** of the SCBT (effectiveness may relate to more than one objective, you may wish to detail/nuance)? Have these expectations changed since the beginning of TreatPaCS? If yes, please specify.

What are your current impressions of the **ease of implementation** of the SCBT? Have these impressions changed since the beginning of TreatPaCS? If so, please explain.

What is your current judgement of the **objectives and aspects worked** on by the SCBT? Has this judgement changed since the beginning of TreatPaCS? If so, please specify.

What are your current impressions of the **adaptability** of the SCBT to each child? Have these impressions changed since the beginning of TreatPaCS? If so, please explain.

What are your current impressions of the **investment** required of the parent and the SLT in the SCBT? Have these impressions changed since the beginning of TreatPaCS? If so, please explain.

Is there any aspect of the SCBT that you **particularly appreciate** at present? If yes, please specify. Have there been any changes in this area since the start of TreatPaCS?

Is there any aspect of the SCBT that you **currently dislike**? If yes, please specify. Have there been any changes in this area since the start of TreatPaCS?

What do you think of the **documents accompanying** the SCBT (manual, checklists, others)? Do you find them sufficiently helpful and detailed? Please specify.

Did your participation in this study lead to any changes in your professional practice? If so, please specify.

Le point de vue des cliniciens impliqués dans l'étude TreatPaCS

Nom, prénom :

Nombre d'années d'expérience dans la prise en charge des enfants d'âge préscolaire qui bégaient :

Nombre d'années d'expérience dans la mise en place du programme Lidcombe :

Nombre d'années d'expérience dans la mise en place du programme Mini-KIDS :

Nombre d'années d'expérience dans la mise en place de la thérapie sociale-cognitive :

Avez-vous actuellement une préférence pour un des trois programmes ? Si oui, pouvez-vous détailler ce qui explique actuellement cette préférence ?

Par rapport au programme Lidcombe,

Quelles sont vos attentes actuelles quant à l'efficacité du Programme Lidcombe (l'efficacité peut porter sur plusieurs objectifs, vous pouvez détailler/nuancer) ? Ces attentes ont-elles changé depuis le début de TreatPaCS ? Si oui, précisez.

Quelles sont vos impressions actuelles par rapport à la facilité de mise en œuvre du Programme Lidcombe ? Ces impressions ont-elles changé depuis le début de TreatPaCS ? Si oui, précisez.

Quelle est votre appréciation actuelle des objectifs visés et des aspects travaillés par le Programme Lidcombe ? Cette appréciation a-t-elle changé depuis le début de TreatPaCS ? Si oui, précisez.

Quelles sont vos impressions actuelles par rapport au caractère adaptable du Programme Lidcombe à chaque patient ? Ces impressions ont-elles changé depuis le début de TreatPaCS ? SI oui, précisez.

Quelles sont vos impressions actuelles par rapport à l'investissement demandé dans le Programme Lidcombe pour le parent et la logopède? Ces impressions ont-elles changé depuis le début de TreatPaCS ? SI oui, précisez.

Y a-t-il un aspect du Programme Lidcombe que vous appréciez spécialement, actuellement ? Si oui, veuillez préciser. Y a-t-il eu des changements à ce niveau depuis le début de TreatPaCS ?

Y a-t-il un aspect de du Programme Lidcombe que vous aimez moins, actuellement ? SI oui, veuillez préciser. Y a-t-il eu des changements à ce niveau depuis le début de TreatPaCS ?

Que pensez-vous ses documents accompagnant le Programme Lidcombe (manuel, checklists, autres) ? Vous semblent-ils suffisamment aidant et détaillés ? Veuillez préciser.

Par rapport au programme Mini-KIDS

Quelles sont vos attentes actuelles par rapport à l'efficacité du programme Mini-KIDS (l'efficacité peut porter sur plusieurs objectifs, vous pouvez détailler/nuancer) ? Ces attentes ont-elles changé depuis le début de TreatPaCS ? Si oui, précisez.

Quelles sont vos impressions actuelles par rapport à la facilité de mise en œuvre du programme Mini-KIDS ? Ces impressions ont-elles changé depuis le début de TreatPaCS ? Si oui, précisez.

Quelle est votre appréciation actuelle des objectifs visés et des aspects travaillés par le programme Mini-KIDS ? Cette appréciation a-t-elle changé depuis le début de l TreatPaCS ? Si oui, précisez.

Quelles sont vos impressions actuelles par rapport au caractère adaptable du programme Mini-KIDS à chaque patient ? Ces impressions ont-elles changé depuis le début de l TreatPaCS ? Si oui, précisez.

Quelles sont vos impressions actuelles par rapport à l'investissement demandé dans le programme Mini-KIDS pour le parent et la logopède ? Ces impressions ont-elles changé depuis le début de TreatPaCS ? Si oui, précisez.

Y a-t-il un aspect du programme Mini-KIDS que vous appréciez spécialement, actuellement ? SI oui, veuillez préciser. Y a-t-il eu des changements à ce niveau depuis le début de TreatPaCS ?

Y a-t-il un aspect du programme Mini-KIDS que vous aimez moins, actuellement ? SI oui, veuillez préciser. Y a-t-il eu des changements à ce niveau depuis le début de TreatPaCS ?

Que pensez-vous des documents accompagnant le programme Mini-KIDS (manuel, checklists, autres) ? Vous semblent-ils suffisamment aidant et détaillés ? Veuillez préciser.

Par rapport à la thérapie sociale-cognitive

Quelles sont vos attentes actuelles par rapport à l'efficacité de la thérapie sociale-cognitive (l'efficacité peut porter sur plusieurs objectifs, vous pouvez détailler/nuancer) ? Ces attentes ont-elles changé depuis le début de TreatPaCS ? Si oui, précisez.

Quelles sont vos impressions actuelles par rapport à la facilité de mise en œuvre de la thérapie sociale-cognitive ? Ces impressions ont-elles changé depuis le début de TreatPaCS ? Si oui, précisez.

Quelle est votre appréciation actuelle des objectifs visés et des aspects travaillés par la thérapie sociale-cognitive? Cette appréciation a-t-elle changé depuis le début de TreatPaCS ? Si oui, précisez.

Quelles sont vos impressions actuelles par rapport au caractère adaptable de la thérapie sociale-cognitive à chaque patient ? Ces impressions ont-elles changé depuis le début de TreatPaCS ? Si oui, précisez.

Quelles sont vos impressions actuelles par rapport à l'investissement demandé dans la thérapie sociale-cognitive pour le parent et la logopède ? Ces impressions ont-elles changé depuis le début de TreatPaCS ? Si oui, précisez.

Y a-t-il un aspect de la thérapie sociale-cognitive que vous appréciez spécialement, actuellement ? SI oui, veuillez préciser. Y a-t-il eu des changements à ce niveau depuis le début de TreatPaCS ?

Y a-t-il un aspect de la thérapie sociale-cognitive que vous aimez moins, actuellement ? Si oui, veuillez préciser. Y a-t-il eu des changements à ce niveau depuis le début de TreatPaCS ?

Que pensez-vous des documents accompagnant la thérapie sociale-cognitive (manuel, checklists, autres) ? Vous semblent-ils suffisamment aidant et détaillés ? Veuillez préciser.

Votre participation à cette étude a-t-elle amené des changements dans votre pratique professionnelle ? Si oui, veuillez spécifier.

Perspectief van logopedisten betrokken in de TreatPaCS-studie

Familienaam, voornaam:

Ervaringen met het behandelen van kleuters die stotteren (in jaren ervaring) :

Ervaringen met het Lidcombe Programma (in jaren ervaring) :

Ervaringen met Mini-KIDS (in jaren ervaring) :

Ervaringen met de sociaal-cognitieve gedragstherapie (in jaren ervaring) :

Heb je momenteel een voorkeur voor één van de drie behandelingen? Indien ja, kan je uitleggen wat deze voorkeur momenteel inhoudt?

Over het Lidcombe Programma,

Wat zijn je verwachtingen op dit moment over de effectiviteit van het Lidcombe Programma (effectiviteit houdt mogelijks meer dan één doel in, specifieer/nuanceer gerust)? Zijn je verwachtingen veranderd sinds de start van TreatPaCS? Indien ja, wil je uitleggen waarom?

Hoe moeilijk of gemakkelijk denk je op dit moment dat het Lidcombe Programma kan worden geïmplementeerd? Denk je hier anders over sinds de start van TreatPaCS? Indien ja, wil je uitleggen waarom?

Wat denk je op dit moment over de doelen en aspecten waarop gewerkt wordt in het Lidcombe Programma? Denk je hier anders over sinds de start van TreatPaCS? Indien ja, wil je uitleggen waarom?

Wat denk je op dit moment over de mogelijkheid om het Lidcombe Programma aan te passen aan elke kleuter die je behandelt? Denk je hier anders over sinds de start van TreatPaCS? Indien ja, wil je uitleggen waarom?

Wat denk je op dit moment over de betrokkenheid die in het Lidcombe Programma gevraagd wordt aan de ouder en de logopedist? Denk je hier anders over sinds de start van TreatPaCS? Indien ja, wil je uitleggen waarom?

Is er een aspect in het Lidcombe Programma dat je op dit moment bijzonder waardeert? Indien ja, wil je uitleggen waarom? Denk je hier anders over sinds de start van TreatPaCS? Indien ja, wil je uitleggen waarom?

Is er een aspect in het Lidcombe Programma dat je op dit moment helemaal niet waardeert? Indien ja, wil je uitleggen waarom? Denk je hier anders over sinds de start van TreatPaCS? Indien ja, wil je uitleggen waarom?

Hoe vind je de de documenten die voor het Lidcombe Programma beschikbaar zijn (handleiding, checklijsten, andere)? Vind je ze Vind je ze voldoende behulpzaam en gedetailleerd? Kan je dit uitleggen?

Over Mini-KIDS,

Wat zijn je verwachtingen op dit moment over de effectiviteit van Mini-KIDS (effectiviteit houdt mogelijks meer dan één doel in, specifieer/nuanceer gerust)? Zijn je verwachtingen veranderd sinds de start van TreatPaCS? Indien ja, wil je uitleggen waarom?

Hoe moeilijk of gemakkelijk denk je op dit moment dat Mini-KIDS kan worden geïmplementeerd? Denk je hier anders over sinds de start van TreatPaCS? Indien ja, wil je uitleggen waarom?

Wat denk je op dit moment over de doelen en aspecten waarop gewerkt wordt in Mini-KIDS? Denk je hier anders over sinds de start van TreatPaCS? Indien ja, wil je uitleggen waarom?

Wat denk je op dit moment over de mogelijkheid om Mini-KIDS aan te passen aan elke kleuter die je behandelt? Denk je hier anders over sinds de start van TreatPaCS? Indien ja, wil je uitleggen waarom?

Wat denk je op dit moment over de betrokkenheid die in Mini-KIDS gevraagd wordt aan de ouder en de logopedist? Denk je hier anders over sinds de start van TreatPaCS? Indien ja, wil je uitleggen waarom?

Is er een aspect in Mini-KIDS dat je op dit moment bijzonder waardeert? Indien ja, wil je uitleggen waarom? Denk je hier anders over sinds de start van TreatPaCS? Indien ja, wil je uitleggen waarom?

Is er een aspect in Mini-KIDS dat je op dit moment helemaal niet waardeert? Indien ja, wil je uitleggen waarom? Denk je hier anders over sinds de start van TreatPaCS? Indien ja, wil je uitleggen waarom?

Hoe vind je de de documenten die voor Mini-KIDS beschikbaar zijn (handleiding, checklijsten, andere)? Vind je ze Vind je ze voldoende behulpzaam en gedetailleerd? Kan je dit uitleggen?

Concerning the SCBT,

Wat zijn je verwachtingen op dit moment over de effectiviteit van de sociale cognitieve gedragstherapie (effectiviteit houdt mogelijks meer dan één doel in, specifieer/nuanceer gerust)? Zijn je verwachtingen veranderd sinds de start van TreatPaCS? Indien ja, wil je uitleggen waarom?

Hoe moeilijk of gemakkelijk denk je op dit moment dat de sociale cognitieve gedragstherapie kan worden geïmplementeerd? Denk je hier anders over sinds de start van TreatPaCS? Indien ja, wil je uitleggen waarom?

Wat denk je op dit moment over de doelen en aspecten waarop gewerkt wordt in de sociale cognitieve gedragstherapie? Denk je hier anders over sinds de start van TreatPaCS? Indien ja, wil je uitleggen waarom?

Wat denk je op dit moment over de mogelijkheid om de sociale cognitieve gedragstherapie aan te passen aan elke kleuter die je behandelt? Denk je hier anders over sinds de start van TreatPaCS? Indien ja, wil je uitleggen waarom?

Wat denk je op dit moment over de betrokkenheid die in h de sociale cognitieve gedragstherapie gevraagd wordt aan de ouder en de logopedist? Denk je hier anders over sinds de start van TreatPaCS? Indien ja, wil je uitleggen waarom?

Is er een aspect van de sociale cognitieve gedragstherapie dat je op dit moment bijzonder waardeert? Indien ja, wil je uitleggen waarom? Denk je hier anders over sinds de start van TreatPaCS? Indien ja, wil je uitleggen waarom?

Is er een aspect van de sociale cognitieve gedragstherapie dat je op dit moment helemaal niet waardeert? Indien ja, wil je uitleggen waarom? Denk je hier anders over sinds de start van TreatPaCS? Indien ja, wil je uitleggen waarom?

Hoe vind je de de documenten die voor de sociale cognitieve gedragstherapie beschikbaar zijn (handleiding, checklijsten, andere)? Vind je ze Vind je ze voldoende behulpzaam en gedetailleerd? Kan je dit uitleggen?

Leidde je deelname aan TreatPaCS tot veranderingen in je professionele aanpak? Indien ja, wil je dit uitleggen?

APPENDIX 3. ANAMNESIS FORM

English/French/Dutch version

Underlined text is to be uploaded to the REDCap system.

(I/E) = Inclusion/Exclusion criterion (also uploaded to REDCap)

IDENTIFICATION CHILD/IDENTICATION ENFANT/IDENTIFICATIE KIND

Full name/Nom et prénom/Volledige naam:

Gender child/Sexe de l'enfant/Geslacht van het kind:

Dat of birth of child/Date de naissance de l'enfant/Geboortedatum van het kind:

(I/E) Age of child at this time (= the time of informed consent signature)/Age de l'enfant à ce moment (= moment de la signature du consentement éclairé)/Leeftijd van het kind op dit moment (= het moment waarop het informed consent getekend wordt): ... Y (Number of years/ Nombre d'années/ Aantal jaren) .... (Number of months/ Nombre de mois/ Aantal maanden)

Travel time to the SLT/ Temps de déplacement chez la logopède/ Reistijd naar logopedist:

- 0-5 minutes
- 6-10 minutes
- 11-15 minutes
- 16-20 minutes
- 21-25 minutes
- 26 – 30 minutes
- > 30 minutes (please specify)

FAMILY CIRCUMSTANCES/CONDITIONS FAMILIALES/FAMILIALE OMSTANDIGHEDEN

Do the parents live together? Les parents vivent-ils ensemble? Wonen de ouders samen?

Yes/no Oui/non Ja/nee

Parents are married/ Parents sont mariés/ Ouders zijn getrouwd

Parents are living together/ Parents sont co-habitants/ Ouders zijn samenwonend

Parents are divorced/ Parents sont divorcés/ Ouders zijn gescheiden

Other/ Autre/ Ander:

Extra information/Commentaires/Extra informatie:

Does the child have siblings? / L'enfant, a-t-il des frères-soeurs? / Heeft het kind broers-zussen?

Yes/no/ Oui/non/ Ja/nee,

How many?/Combien?/Hoeveel?

Age for each brother/Age pour chaque frère /Leeftijd voor elke broer:

1. ......
2. ....
3. ....
4.

Age for each sister/Age pour chaque soeur /Leeftijd voor elke zus:

1. ......
2. ....
3. ....
4.

DESCRIPTION OF STUTTERING BEHAVIOUR/DESCRIPTION DU BEGAIEMENT/BESCHRIJVING VAN HET STOTTEREN

Description (by the parent) of the stuttering behaviour at home and at school at this moment- short summary):....................

Description (par le parent) du bégaiement à la maison et à l'école maintenant -: court résumé: ....

Beschrijving (door de ouder) van het stottergedrag thuis en op school op dit moment - / korte samenvatting: ....

Identified (by SLT) overt stuttering behaviour/ Identifié (par la logopède) comportements de bégaiement observables /Overt stottergedrag (geïdenitificeerd door de logopedist)

More than one can apply/ Plus qu'une peut être présent/ Meer dan één kan aanwezig zijn:

- Repetitions (syllable, incomplete syllable repetition, multisyllable unit repetition)
  Répétitions (syllabe, syllabes incomplètes, répétitions de plusieurs syllabes)
  Herhalingen (lettergreep, onvolledige lettergreepherhaling, multisyllabische deelherhaling)
  Ask for examples/Demander des exemples/Vraag naar een voorbeeld:
- 'Fixed postures' (audible prolongations, prolongations without audible airflow, blocks)
  'Postures fixes' (prolongations audibles, prolongations silencieuses, blocages)
  'Fixed postures' (hoorbare verlengingen, niet hoorbare verlengingen, blokkades)
  Ask for examples/Demander des exemples/Vraag naar een voorbeeld:
- Reactive behaviour/Comportement associés/Reactief gedrag:
- Tension/Tension/Spanning (Where, how? /Où, comment? /Waar, hoe?):
- Other fighting behaviour/Autre comportement de forçage/Ander vechtgedrag:
- Avoiding behaviour/Comportement d'évitement/Vermijdingsgedrag:
- Other observable behaviour (Start behaviour, postponing behaviour, other):
  Autre comportement observable pour aider au démarrage des mots, pour retarder la prononciation d'un mot, etc.):
  Ander observeerbaar gedrag (startgedrag, uitstelgedrag, ander):

Identified (by SLT) covert stuttering behaviour/ Identifié (par la logopède) comportements non observables/ Covert stottergedrag (geïdenitificeerd door de logopedist):

- Non-verbal signs/Signes non-verbaux/Non verbale tekenen:
- Verbal signs (Child formulated a concern)/ Signes verbaux (L'enfant formulait une préoccupation./ Verbale tekenen (Kind formuleerde een probleem). Explain/Expliquez/Leg uit: ....

How would you (parent) rate the child's stuttering severity today? / Comment évalues-tu (parent) la gravité du bégaiement de l'enfant aujourd'hui ? / Hoe zou u (ouder) de stotterernst van je kind vandaag beoordelen?
Please put the slider on the line for your child's stuttering severity today with 0 = no stuttering, 1 = extremely mild stuttering and 9 = extremely severe stuttering. / Veuillez mettre le curseur sur la ligne à l'endroit correspondant à la sévérité du bégaiement de votre enfant aujourd'hui avec la sévérité de 0 = pas de bégaiement, 1 = extrêmement léger et 9 = extrêmement grave. / Zet de slider alstublieft op de lijn voor de stotterernst van uw kind vandaag met 0 = geen stotteren, 1 = extreem mild stotteren en 9 = extreem ernstig stotteren.

No stuttering	Extremely mild stuttering	Extremely severe stuttering

How satisfied are you (parent) with your child's communication in everyday speaking situations at the present time? / Dans quelle mesure es-tu (parent) satisfait de la communication de ton enfant dans les situations d'expression quotidienne à ce moment-ci? / Hoe tevreden bent u (ouder) met de communicatie van uw kind in alledaagse spreeksituaties op dit moment?
Please put the slider on the line on this 9-point scale with 1 = extremely satisfied and 9 = extremely dissatisfied / Veuillez mettre le curseur sur la ligne avec 1 = extrêmement satisfait et 9 = extrêmement mécontent/ Zet de slider alstublieft op de lijn met 1 = extreem tevreden en 9 = extreem ontevreden.

Extremely satisfied	Extremely dissatisfied

STUTTERING DETAILS/DETAILS DU BEGAIEMENT/GEGEVENS OVER HET STOTTEREN

- What was the onset of the stuttering (date or period)/Quand est-ce que le bégaiement apparaît (date ou période)/ Wanneer was de onset van het stotteren:.... (months/mois/maand) ...... (year/année/jaar)

- Time since onset/ Temps écoulé depuis l'apparition du bégaiement / Periode sinds de onset van het stotteren =

- Description (by the parent) of the stuttering / Description (par le parent) du bégaiement tel qu'il était au moment de son apparition/ Beschrijving (door de ouder)van het stotteren bij onset:
  More than one can apply/ Plus qu'une peut être présent/ Meer dan één kan aanwezig zijn:
  - Repetitions/Répétitions/Herhalingen
  - Audible prolongations/Prolongation audibles/Hoorbare verlengingen
  - Prolongations without audible airflow/Prolongations silencieuses, blocades/Niet hoorbare fixaties/blokkades
  - Reactive behaviour/Comportement associé/Reactief gedrag:
  - Tension (waar, hoe)/Tension (où, comment)/Spanning (waar, hoe):
  - Other fighting beahaviour/Autre comportement de forçage/Ander vechtgedrag:
  - Avoiding behaviour/Comportement d'évitement/Vermijdingsgedrag:
  - Other observable behaviour (Start behaviour, postponing behaviour, other):
    Autre comportement observable pour aider au démarrage des mots, pour retarder la prononciation d'un mot, etc.):
    Ander observeerbaar gedrag (startgedrag, uitstelgedrag, ander):
  - Non-verbal signs/Signes non-verbaux/Non verbale tekenen: Explain/Expliquez/Leg uit: ....
  - Verbal signs (Child formulated a concern)/ Signes verbaux (L'enfant formulait une préoccupation. / Verbale tekenen (Kind formuleerde een probleem). Explain/Expliquez/Leg uit: ....

- Evolution since onset/Evolution depuis le début/Evolutie sinds onset:
  - Gradual increase of stuttering frequency and severity/ Augmentation graduelle de la fréquence et de la gravité du bégaiement/ Geleidelijke toename stotterfrequentie en -ernst:
  - Cyclic but with increased frequency and increased tension, physical and emotional reactions/ Cyclique mais la fréquence et la gravité augmentent avec chaque épisode (tension, réponses physiques et émotionelles)/ Cyclisch maar met toenemende frequentie en spanning, en met fysieke en emotionele reacties
  - Cyclic but with same frequency and severity, it stays the same/ Cyclique mais avec la même gravité et la même fréquence dans chaque épisode/ Cyclisch maar met dezelfde frequentie en ernst, het blijft hetzelfde

  - When does the stuttering gets more severe?
    Quand le bégaiement s'aggrave-t-il?
    Wanneer is het stotteren ernstiger?

  - Has the urge to speak decreased since onset? Yes/No. *Ask examples.*

Le besoin de parler, a-t-elle diminué? Oui/Non. *Demander des exemples.*

Is de drang om te spreken verminderd sinds onset? Ja/Nee. *Vraag naar voorbeelden*.

- Is the urge to speak the same since onset? Yes/No. *Ask for examples.*

  L'envie de parler, est-elle restée la même qu'avant? Oui/Non. *Demande des exemples.*

  Is de drang om te spreken hetzelfde sinds onset? Ja/Nee. *Vraag naar voorbeelden*.

- Are there reactions towards the stuttering from the child, parent(s) or others? Yes/No. Ask for examples.

  Y a-t-il des réactions par rapport au bégaiement de la part de l'enfant, des parent(s) ou autres? Oui/Non. Demander des exemples.

  Zijn er reacties op het stotteren door het kind, ouder(s) of anderen? Ja/Nee. Vraag naar voorbeelden.

  - How does the child respond?

    Comment réagit l'enfant?

    Hoe reageert uw kind?
  - How do you respond?

    Comment réagissez-vous?

    Hoe reageert u?
  - How do you try to help?

    Comment essayez-vous d'aider votre enfant?

    Hoe helpt u?
  - How do the siblings repond (how do they try to help)?

    Comment réagissent les frères/les soeurs (comment aident-ils)?

    Hoe reageren de broers/zussen (hoe helpen ze)?
  - How do the grandparents respond (how do they try to help)?

    Comment les grands-parents réagissent-ils (comment aident-ils)?

    Hoe reageren de grootouders (hoe helpen ze)?
  - How do peers respond (how do they try to help)?

    Comment réagissent les petits amis (comment aident-ils)?

    Hoe reageren leeftijdsgenootjes (hoe helpen ze)?
  - Others?

    Autres?

    Anderen?

- Has your child received stuttering treatment before? Yes/No. If yes, please explain. Votre enfant a-t-il déjà reçu un traitement contre le bégaiement ? Si oui, pouvez-vous expliquer? Werd uw kind reeds behandeld voor stotteren? Indien wel, kan u dit uitleggen?

- Family history of stuttering/Hérédité du bégaiement/Familiaal voorkomen van stotteren

- Present-Not present/Présente -Pas présente/Komt voor-Komt niet voor.
- Relationship with that person/Relation avec cette personne/Relatie met deze persoon:
- Recovery of stuttering? Yes-No/ Récupération? Oui-Non/Herstel? Ja-Nee.
- Did this relative receive treatment? Yes-No. Duration? Type of treatment?

  Cette personne a-t-elle suivi une prise en charge? Oui-Non. Durée? Type de prise en charge?

  Werd deze persoon behandeld? Ja-Nee. Duur? Soort behandeling?

LANGUAGE AND SPEECH DEVELOPMENT/DEVELOPEMENT DU LANGUAGE ET PAROLE/SPRAAK- EN TAALONTWIKKELING

- How does the speech- and language development evolve?

  Comment le language et la parole se développent-ils?

  Hoe verloopt de spraak- en taalontwikkeling? L
  - Slower than his/her peers/ Plus lent que ses camarades / Langzamer dan zijn/haar leeftijdsgenoten
  - At the same pace as his/her peers/ Au même rythme que ses camarades / In hetzelfde tempo als zijn/haar leeftijdsgenoten
  - Faster than his/her peers/ Plus rapide que ses camarades/ - - Sneller dan zijn/haar leeftijdsgenoten
- When did s/he start using words?

  Quand a-t-il/elle dit ses premiers mots ?

  Wanneer uitte h/zij de eerste woordjes ?
- When has s/he started to combine words?

  Quand a-t-il/elle fait ses premières combinaisons de mots ?

  Wanneer maakte h/zij de eerste zinnetjes ?
- Was there a rapid growth of language?

  Y a-t-il eu une phase d'explosion rapide du langage?

  Was er een plotse toename in de taalontwikkeling?

- Is your child well intelligible or difficult to understand? (*ask examples*)

  Votre enfant est facile ou difficile à comprendre? (*demander des exemples*)

  Is uw kind gemakkelijk of moeilijk te verstaan? (*vraag voorbeelden*)
  - Well intelligible / facile à comprendre / gemakkelijk te verstaan
  - Difficult to understand / difficile à comprendre / moeilijk te verstaan

- Do you think your child's vocabulary (knowledge of words) and syntax (sentence buidling) is well developed? Yes/no ((*ask examples*)

  Trouvez-vous que le vocabulaire (connaissance des mots) et la syntaxe (construction des phrases) sont bien développés? Oui/non (*demander des exemples*):

  Vindt u de woordenschat (kennis van woorden) en syntax (zinsbouw) van uw kind goed ontwikkeld? Ja/nee (*vraag voorbeelden*):

- Did your child have speech-language therapy? Yes/ No. Why?
  Votre enfant a-t-il déjà été suivi en logopédie ? Oui/ Non. Pour quelles raisons ?
  Volgde uw kind reeds logopedie? Ja/Nee. Waarom?

- Is your child bilingual? Yes/No. If yes, what languages does s/he speak?
  Votre enfant est-il bilingue ? Oui/Non. Si oui, quelles sont les langues qu'il/elle parle ?
  Is uw kind tweetalig? Ja/Nee. Zo ja, welke talen spreekt hij/zij?

- Are you bilingual? Yes/No. If yes, what languages do you speak? (I) Do you understand one of these languages well enough to have a conversation? English / Dutch / French?
  Êtes-vous bilingue ? Oui/Non. Si oui, quelles langues parlez-vous ? Comprenez-vous l'une de ces langues suffisamment bien pour tenir une conversation ? Anglais / néerlandais / français ?
  Bent u tweetalig? Ja/Nee. Zo ja, welke talen spreekt u? Verstaat u een van deze talen goed genoeg om een gesprek te voeren? Engels / Nederlands / Frans?

GENERAL DEVELOPMENT/DEVELOPEMENT GENERALE/ALGEMENE ONTWIKKELING

- How does the gross motor development evolve?/ Développement de la motricité globale/Grove motorische ontwikkeling:
  - Slower than his/her peers/ Plus lent que ses camarades / Langzamer dan zijn/haar leeftijdsgenoten
  - At the same pace as his/her peers/ Au même rythme que ses camarades / In hetzelfde tempo als zijn/haar leeftijdsgenoten
  - Faster than his/her peers/ Plus rapide que ses camarades/ - - Sneller dan zijn/haar leeftijdsgenoten

- How does the fine motor development evolve?/ Développement de la motricité fine/Fijne motorische ontwikkeling:
  - Slower than his/her peers/ Plus lent que ses camarades / Langzamer dan zijn/haar leeftijdsgenoten
  - At the same pace as his/her peers/ Au même rythme que ses camarades / In hetzelfde tempo als zijn/haar leeftijdsgenoten
  - Faster than his/her peers/ Plus rapide que ses camarades/ - - Sneller dan zijn/haar leeftijdsgenoten

- Does your child attend day care or preschool ? in what year is s/he ?
  Votre enfant fréquente-t-il l'école ou la crèche ? En quelle année est-il ?
  Gaat uw kind naar de kribbe of kleuterschool ? In welk jaar in de kleuterschool zit h/zij ?
  - To day care/ à la crèche/ naar de kribbe
  - To preschool/ à l 'école / naar school
  - Other : ....

- Do you have concerns about development from school/Des problèmes ont-ils été signalés à l'école/ Werden er bezorgdheden gerapporteerd op school? Yes/no – Oui/Non – Ja/ Nee

- **(I/E) Does your child have a hearing loss? Yes/No**
  Votre enfant a-t-il une perte d'audition ? Oui/Non
  Heeft uw kind een gehoorverlies? Ja/Nee

- **(I/E) Does your child have Down Syndrome? Yes/no**
  Votre enfant est-il atteint du syndrome de Down ? Oui/Non
  Heeft uw kind het syndroom van Down? Ja/Nee

- Are there other comorbidities (genetic, neurological or medical such as vision, illnesses)? Yes/no Explain.
  Existent-ils d'autres comorbidités (génétique, neurologique ou médicaux des problèmes de vue ou d'autres maladies)? Oui/Non. Expliquez.
  Zijn er andere comorbiditeiten (genetische, neurologische of medische zoals zicht, ziektes)? Ja:Nee. Leg uit

- Does s/he take any medication? Yes/no
  Prend-il des médicaments ? Oui/Non
  Neemt h/zij medicatie? Ja/Nee

QUESTIONS ABOUT PARENT PROFILE/ QUESTIONS CONCERNANT DU PARENT/ VRAGEN OVER PROFIEL OUDER (PARENT TRAINED TO IMPLEMENT THE TREATMENT)

- What is your email address?
- What is your phone number?
- What is your highest level of education? / Quel est votre plus haut niveau d'éducation ? / Wat is uw hoogst behaalde diploma?
  ☐ Secondary education not completed / Enseignement secondaire non terminé / Secundair onderwijs niet afgewerkt
  ☐ Secondary education completed (or equivalent) / Enseignement secondaire terminé (ou équivalent) / Secundair onderwijs afgewerkt (of equivalent)
  ☐ Higher Education (bachelor degree, master degree, doctoral degree) / Enseignement supérieur (licence, master, doctorat) / Hoger onderwijs (bachelor diploma, master diploma, doctoraal diploma)

- What is your profession? / Quelle est votre profession? / Wat is uw beroep? ....

- Do you have any speech or language related difficulties (e.g., ADHD, Autism Spectrum Disorder, learning disorder, ...)? No – Yes, ...

Avez-vous des difficultés liées à la parole, au langage ou aux apprentissages (par exemple, TDAH, troubles du spectre autistique, difficultés d'apprentissage, ...)?
Ondervindt u moeilijkheden met spraak- of taal (e.g., ADHD, Autism Spectrum Stoornis, leerstoornis, ...)?

- <u>Do you have any medical problems, such as acute or chronic illness, disability, depression or other psychological disorder? Yes/no Please, specify</u>
  Avez-vous des problèmes médicaux, tels qu'une maladie acute ou chronique, un handicap, une dépression ou un autre trouble psychologique ? Oui/Non. Veuillez préciser
  Heeft u medische problemen, zoals een acute of chronische ziekte, handicap, depressie of andere psychische aandoening? Ja/Nee. Gelieve te specificeren
- <u>Does a close family member have any medical difficulties, such as acute or chronic illness, disability, speech or language related difficulties, depression or other psychological disorder? Yes/No. Please, specify</u>
  Un membre de la famille proche a-t-il des difficultés médicales, telles qu'une maladie acute ou chronique, un handicap, des difficultés associées à la parole ou le language, une dépression ou un autre trouble psychologique ? Oui/Non. Veuillez préciser
  Heeft een naast familielid medische problemen, zoals een acute of chronische ziekte, handicap,spraak- of taalgeralteerde moeilijkheden, depressie of andere psychische stoornis? Ja/Nee. Gelieve te specificeren

- How much do you agree or disagree with the following statements?
  Dans quelle mesure êtes-vous d'accord ou non avec les affirmations suivantes ?
  In hoeverre bent u het eens of oneens met de volgende beweringen?

Your life is very busy due to work, child activities, social outing and/or other. You have hardly any moment to be relaxed at home with your child/children.<br>Votre vie est très chargée en raison du travail, des activités des enfants, des sorties sociales et/ou autres. Vous n'avez pratiquement aucun moment pour être transuille à la maison avec votre/vos enfant(s).<br>Uw leven is erg druk door werk, kinderactiviteiten, sociale uitjes en/of andere zaken. U heeft nauwelijks tijd omrustig thuis bij uw kind(eren) te zijn.				
Totally disagree	Disagree	Not disagree, not agree	Agree	Totally agree
Your relationship with your child is very good at any moment.<br>Votre relation avec votre enfant est très bonne à tout moment.<br>Uw relatie met uw kind is op elk moment heel goed.				
Totally disagree	Disagree	Not disagree, not agree	Agree	Totally agree
It is very easy for you to steer a conversation with your child. That means, if your child talks a lot (in a flow), you still manage to take turns and participate in the conversation.<br>Il est très facile pour vous de diriger une conversation avec votre enfant. Cela signifie que si votre enfant parle beaucoup (dans un flux), vous parvenez tout de même à prendre votre tour et à participer à la conversation.<br>Het is heel gemakkelijk voor u om een gesprek met uw kind te sturen. Dat wil zeggen, als uw kind veel praat (in een stroom), lukt het u nog steeds om de beurt te nemen en deel te nemen aan het gesprek.				
Totally disagree	Disagree	Not disagree, not agree	Agree	Totally agree
You are a very anxious person.<br>Vous êtes très anxieux.<br>U bent erg angstig				
Totally disagree	Disagree	Not disagree, not agree	Agree	Totally agree
You enjoy playing with your child very much. You do this often at home.<br>Vous aimez beaucoup jouer avec votre enfant. Vous le faites souvent à la maison.<br>U speelt graag met uw kind. U doet dit vaak thuis.				
Totally disagree	Disagree	Not disagree, not agree	Agree	Totally agree
It is very easy for you to set limits to certain behaviour of your child.<br>Il est très facile pour vous de fixer des limites à certains comportements de votre enfant.<br>Het is heel gemakkelijk voor u om grenzen te stellen aan bepaald gedrag van uw kind.				
Totally disagree	Disagree	Not disagree, not agree	Agree	Totally agree
You are a fast talker.<br>Vous parlez vite.<br>U bent een snelle prater.				
Totally disagree	Disagree	Not disagree, not agree	Agree	Totally agree
You are a rigorous person, that means, you always try to do a job thoroughly.<br>Vous êtes une personne rigoureuse, c'est-à-dire que vous essayez toujours de faire un travail à fond.<br>U bent een rigoureus persoon, dat wil zeggen, u probeert altijd een klus grondig uit te voeren.				
Totally disagree	Disagree	Not disagree, not agree	Agree	Totally agree
You are a very organised person.<br>Vous êtes une personne très organisée.<br>U bent een zeer georganiseerd persoon.				

Totally disagree	Disagree	Not disagree, not agree	Agree	Totally agree
You are very worried about your child's stuttering.<br>Vous êtes très inquiet au sujet du bégaiement de votre enfant.<br>U bent erg ongerust over het stotteren van uw kind.				
Totally disagree	Disagree	Not disagree, not agree	Agree	Totally agree
You are used to give your child many compliments for different types of behaviour.				
Totally disagree	Disagree	Not disagree, not agree	Agree	Totally agree

QUESTION FOR HELP/QUESTION DE L'AIDE/HULPVRAAG

- What is the question for help of the parent(s)/Quel sorte d'aide est-ce que les/le parent(s) demande pour leur enfant?/ Welke soort hulp vraagt/vragen de ouder(s):

- What is the question for help of the child?/ Quel sorte d'aide est-ce que l' enfant demande?/ Welke soort hulp vraagt het kind?

- **(I/E) Are you willing to be intensively involved in treatment (implement treatment at home)?**
  Êtes-vous prêt à participer de manière intensive au traitement (mise en œuvre du traitement à domicile)?
  Bent u bereid om intensief bij de behandeling betrokken te zijn (behandeling thuis uitvoeren)?

- **(I/E) Are you willing and able to video record your child regularly for 10-15 minutes in the home environment?**
  Êtes-vous prêt et capable d'enregistrer régulièrement votre enfant par vidéo pendant 10 à 15 minutes dans son environnement familial ?
  Bent u bereid en in staat om uw kind regelmatig gedurende 10-15 minuten in de huiselijke omgeving op video op te nemen?

(I/E) IDENTIFICATION OF STUTTERING

You have used the/ Vous avez utilisé le/ Je grbuikte de SSI-4 / TVS-NL / TSB-NL / other

SSI-4: Total score: .... Percentile: .... Severity equivalent: ....
TVS-NL <6 years: Total score: .... Percentile: .... Severity equivalent: ....
TVS-NL ≥6 years: Total score: .... Percentile: .... Severity equivalent: ....
TBS-NL <6 years: Total score: .... Percentile: .... Severity equivalent: ....
TBS-NL ≥6 years: Total score: .... Percentile: .... Severity equivalent: ....
Other: Total score: .... Percentile: .... Severity equivalent: ....

ACTIVE MONITORING

Did you monitor the child's stuttering actively? / Avez-vous surveillé activement le bégaiement de l'enfant ? / Hebt je het stotteren actief gemonitored voor een periode?

Yes/No – Oui/Non – Ja/Nee

If no, no need to complete the next questions/ Si non, il n'est pas nécessaire de répondre aux questions suivantes/ Indien dienen de volgende vragen niet beantwoord te worden

If yes/ Si oui/ Indien ja:

Did you ask the parent to record information about the stuttering? Yes/no/ Avez-vous demandé au parent de consigner des informations sur le bégaiement ? Oui/Non/ Vroeg je de ouders om informatie van het stotteren bij te houden?

If yes, how? Si oui, comment? Indien ja, hoe?

More than one can apply/ Plus qu'une peut être présent/ Meer dan één kan aanwezig zijn

- ☐ Description of the stuttering, aurally / Description du bégaiement, en parlant / Beschrijven van het stotteren, mondeling
- ☐ Description of the stuttering, written down/ Description du bégaiement, en écrite/ Beschrijven van het stotteren, schriftelijk
- ☐ Severity ratings (10-point scale)/ sévérité du bégaiement (échelle 10-point)/ Stotterernstscores (10-puntenschaal)
- ☐ Other/Autre/Andere

Did you provide general advice about communication? Yes/no / Avez-vous donné des conseils généraux sur la communication? Oui/non / Gaf u ander advies over communicatie? Ja/Nee

If yes, explain/ Si oui, expliquez/ Indien ja, leg uit

When did you have contact with the child and/or parent? Quand avez-vous eu des contacts avec l'enfant et/ou le parent? Wanneer had je contact met het kind en/of de ouder?

1. ..../..../............. (DD/MM/YYYY) Contact via telephone/téléphone/telefoon / email / in a session/séance/sessie / other, explain/Autre, expliquez/Andere, leg uit
2. ..../..../............. (DD/MM/YYYY) Contact via telephone/téléphone/telefoon / email / in a session/séance/sessie / other, explain/Autre, expliquez/Andere, leg uit
3. ..../..../............. (DD/MM/YYYY) Contact via telephone/téléphone/telefoon / email / in a session/séance/sessie / other, explain/Autre, expliquez/Andere, leg uit
4. ..../..../............. (DD/MM/YYYY) Contact via telephone/téléphone/telefoon / email / in a session/séance/sessie / other, explain/Autre, expliquez/Andere, leg uit

5. ..../..../............. (DD/MM/YYYY) Contact via telephone/téléphone/telefoon / email / in a session/séance/sessie / other, explain/Autre, expliquez/Andere, leg uit
6. ..../..../............. (DD/MM/YYYY) Contact via telephone/téléphone/telefoon / email / in a session/séance/sessie / other, explain/Autre, expliquez/Andere, leg uit
7. ..../..../............. (DD/MM/YYYY) Contact via telephone/téléphone/telefoon / email / in a session/séance/sessie / other, explain/Autre, expliquez/Andere, leg uit
8. ..../..../............. (DD/MM/YYYY) Contact via telephone/téléphone/telefoon / email / in a session/séance/sessie / other, explain/Autre, expliquez/Andere, leg uit
9. ..../..../............. (DD/MM/YYYY) Contact via telephone/téléphone/telefoon / email / in a session/séance/sessie / other, explain/Autre, expliquez/Andere, leg uit
10. ..../..../............. (DD/MM/YYYY) Contact via telephone/téléphone/telefoon / email / in a session/séance/sessie / other, explain/Autre, expliquez/Andere, leg uit

APPENDIX 4. KIDDYCAT

APPENDIX 5. QUESTIONS OF THE EQ-5D-Y-PROXY 1

APPENDIX 6. CHILDREN'S BEHAVIOR QUESTIONNAIRE (CBQ)

APPENDIX 7. THE IMPACT OF STUTTERING ON PRESCHOOLERS AND PARENTS (ISPP)

APPENDIX 8. DATA COLLECTION POINTS: PARENT

How would you (parent) rate the child's stuttering severity today? / Comment évaluez-vous (en tant que parent) la sévérité du bégaiement de votre enfant aujourd'hui ? / Hoe zou u (ouder) de stotterernst van uw kind vandaag beoordelen?

Please put the slider on the line for your child's stuttering severity today with 0 = no stuttering, 1 = extremely mild stuttering and 9 = extremely severe stuttering. / Veuillez mettre le curseur sur la ligne à l'endroit correspondant à la sévérité du bégaiement de votre enfant aujourd'hui avec la sévérité de 0 = pas de bégaiement, 1 = extrêmement léger et 9 = extrêmement grave. / Zet de slider alstublieft op de lijn voor de stotterernst van uw kind vandaag met 0 = geen stotteren, 1 = extreem mild stotteren en 9 = extreem ernstig stotteren.

No stuttering	Extremely mild stuttering	Extremely severe stuttering

Pas de bégaiement – bégaiement extrêmement léger - bégaiement extrêmement grave /
Geen stotteren – extreem mild stotteren – extreem ernstig stotteren

How satisfied are you (parent) with your child's communication in everyday speaking situations at the present time? / Dans quelle mesure êtes-vous (en tant que parent) satisfait de la communication de votre enfant dans la vie quotidienne ces moments-ci?/ / Hoe tevreden bent u (ouder) met de communicatie van uw kind in alledaagse spreeksituaties op dit moment?
Please put the slider on the line with 1 = extremely satisfied and 9 = extremely dissastisfied. / Veuillez mettre le curseur sur la ligne avec 1 = extrêmement satisfait et 9 = extrêmement mécontant. /Zet de slider alstublieft op de lijn met 1= Extreem tevreden en 9 = Extreem ontevreden.

Extremely satisfied	Extremely dissatisfied

Extrêmement satisfait – Extrêmement mécontent /
Extreem tevreden – extreem ontevreden

APPENDIX 9. COMPLIANCE & TREATMENT FIDELITY CHECKLISTS

Compliance Checklist *(to check whether parents are implementing the treatment)*

Lidcombe Program

Date treatment session: ..../......../............

Duration of the treatment session: 30 / 60 / ..... minutes

Implementation of the treatment at home was

OR ☐ an agreed practice time at home and lasted ...... minutes. It was performed ... times since the previous treatment session

AND/OR ☐ applying a technique or advice throughout the day

☐ At least one parent attends the treatment session with the child

☐ The parent brings the severity ratings for the typical stuttering severity observed each day (outside of the practice conversations) since the previous treatment session

☐ The parent manages the treatment activity while remaining focused on the child's speech

☐ The parent identifies stuttering moments and only gives verbal contingencies for unambiguous stuttering or unambiguous stutter-free speech

☐ The parent gives verbal contingencies:

- ☐ correctly (in an immediate, accurate, genuine and positive way that does not disrupt the parent-child communication)
- ☐ mostly for stutter-free speech
- ☐ in a varied way regarding the wording, timing, and context
- ☐ frequently enough
- ☐ without negative reaction from the child

☐ The child is fluent most of the time during the practice sessions at home

☐ The parent implemented verbal contingencies at home during practice sessions during 10 to 15 minutes or during natural conversations.

☐ The verbal contingencies were only given by the parent who is trained to do so

Treatment Fidelity Checklist *(to check whether SLTs are implementing the treatment per protocol)*

Lidcombe Program

- ☐ The SLT and/or parent converse(s) with the child
- ☐ The SLT asks the parent to assign a SR score to this conversation and coaches the parent if necessary (difference of >1 SR)
- ☐ The SLT and the parent discuss about stuttering severity and treatment responsiveness during the previous week, based on the stuttering severity ratings brought in by the parents
- ☐ The SLT and the parent discussed on how the treatment was implemented during the previous week
- ☐ The parent demonstrates to the SLT how verbal contingencies were conducted during the previous week and the SLT provides him/her with feedback
- ☐ The SLT observes that the child is feeling comfortable during the treatment sessions and with the treatment that is implemented
- ☐ The parent and SLT discuss changes to procedures for the coming week (verbal contingencies, frequency, context, structure). If time permits (possibly not during the 30-minute treatment sessions), the SLT models them, the parent applies them during a game or a conversation with the child and the SLT gives feedback before deciding whether this new procedure can be applied at home
- ☐ The parent and SLT summarize the plan for the coming week

Compliance Checklist *(vérifier si les parents appliquent le traitement)*

Programme Lidcombe

Date séance de traitement: ..../......../............

Durée de séance de traitement : 30 / 60 / ... minutes

Mettre en œuvre le traitement à la maison était

OU BIEN ☐ un temps de pratique convenu à la maison et a duré ...... minutes. Elle a été réalisée... fois depuis la dernière séance de traitement

ET/OU BIEN ☐ appliquer une technique ou un conseil tout au long de la journée

☐ Au moins un parent est présent à la séance de traitement avec l'enfant

☐ Le parent apporte l'échelle d'évaluation de la sévérité du bégaiement observée chaque jour (en dehors des conversations d'entraînement) depuis la séance de traitement précédente.

☐ Le parent gère l'activité de traitement tout en restant concentré sur le discours de l'enfant

☐ Le parent identifie les moments de bégaiement et ne donne des commentaires verbaux que pour un bégaiement non ambigu ou une parole non bégayée (non ambiguë).

☐ Le parent donne des commentaires verbaux :

- ☐ correctement (d'une manière immédiate, précise, authentique et positive qui ne perturbe pas la communication parent-enfant).
- ☐ principalement pour une parole sans bégaiement
- ☐ d'une manière variée en ce qui concerne la formulation, le moment et le contexte
- ☐ assez souvent
- ☐ sans réaction négative de la part de l'enfant

☐ L'enfant parle de manière fluide la plupart du temps pendant les séances de pratique à la maison.

☐ Le parent a mis en place des commentaires verbaux à la maison chaque jour pendant 10 à 15 minutes au cours de séances d'entraînement ou de conversations naturelles.

☐ Les commentaires verbaux n'ont été donnés que par le parent qui est formé pour le faire.

Treatment Fidelity Checklist *(vérifier si les logopèdes mettent en œuvre le traitement conformément au protocole)*

Programme Lidcombe

☐ Le logopède et/ou le parent converse(nt) avec l'enfant.

☐ Le logopède demande au parent d'attribuer un score ES à cette conversation et l'accompagne si nécessaire (différence de > 1 valeur ES)

☐ Le logopède et le parent discutent de la gravité du bégaiement et de la réponse au traitement au cours de la semaine précédente, sur la base des évaluations de la gravité du bégaiement apportées par les parents.

☐ Le parent montre au logopède comment les commentaires verbaux ont été mis en œuvre au cours de la semaine précédente, et le logopède donne un feedback.

☐ Le logopède observe que l'enfant se sent à l'aise pendant les séances de traitement et avec le traitement mis en œuvre.

☐ Le parent et le logopède discutent des modifications à apporter aux procédures pour la semaine suivante. Si le temps le permet (éventuellement pas pendant les séances de traitement de 30 minutes) et si une nouvelle contingence verbale est introduite, le logopède la modélise, le parent l'applique au cours d'un jeu ou d'une conversation avec l'enfant et le logopède donne son avis avant de décider si cette nouvelle contingence verbale peut être appliquée à la maison.

☐ Le parent et le logopède résument le plan de la semaine

Compliance Checklist *(om na te gaan of ouders de behandelng volgens het behandelprotocol uitvoeren)*

Lidcombe Programma

Datum van de therapiesessie: ..../......../............

Duur van de therapiesessie: 30 / 60 / ..... minuten

Het toepassen van de therapie thuis was

OFWEL ☐ een afgesproken oefenmoment thuis en duurde ...... minuten. Het werd... keer sinds de vorige therapiesessie uitgevoerd

OFWEL ☐ het toepassen van een techniek of advies doorheen de dag

☐ Ten minste 1 ouder volgt de sessie samen met het kind

☐ De ouder brengt de ernstscores voor de typische stotterernst van elke dag (buiten de oefengesprekken) mee van de afgelopen week (sinds de vorige sessie)

☐ De ouder voert het oefengesprek uit én blijft gefocust op de spraak van het kind

☐ De ouder identificeert stottermomenten en geeft enkel verbale contingenties voor onbetwistbaar stotteren en onbetwistbare stottervrije spraak

☐ De ouder geeft verbale contingenties:

- ☐ correct (onmiddellijk en op een accurate, gemeende en positieve manier zonder dat het de kind-ouder communicatie stoort)
- ☐ voornamelijk voor de stottervrije spraak
- ☐ op een gevarieerde manier (woordkeuze, timing en context)
- ☐ voldoende frequent
- ☐ zonder negatieve reactie van het kind

☐ Het kind is meestal stottervrij tijdens de oefengesprekken thuis

☐ De ouder geeft verbale contingenties thuis tijdens oefengesprekken van 10-15 minuten of tijdens natuurlijke gesprekken.

☐ De verbale contingenties werden enkel gegeven door de ouder die getraind is om ze te geven.

Treatment Fidelity Checklist *(om na te gaan of SLT's de behandeling volgens protocol uitvoeren)*

Lidcombe Programma

☐ De logopedist en/of ouder praten met het kind

☐ De logopedist vraagt de ouder om een stotterernstscore (SE) aan dit gesprek toe te wijzen en coacht de ouder indien nodig (indien verschil > SE is)

☐ De logopedist en de ouder praten over de stotterernst en respons op de behandeling tijdens de afgelopen week, en baseren zich hiervoor op de SEs die de ouder meebracht

☐ De logopedist en de ouder praten over hoe het was om de behandeling uit te voeren de afgelopen week

☐ De ouder toont de logopedist hoe de verbale contingenties werden gegeven thuis de afgelopen week en geeft feedback aan de ouder

☐ The logopedist observert of het kind zich op zijn gemak voelt tijdens oefengesprekken en met de behandeling die wordt uitgevoerd

☐ De ouder en de logopedist praten over de wijzigingen voor de behandeling voor de komende week (verbale contingenties, frequentie, context, structuur). Als de tijd het toelaat (mogelijks niet indien het een 30-minuten sessie is), modelleert de logopedist deze wijzigingen en voert de ouder deze uit tijdens een spel of gesprek met het kind. De logopedist geeft feedback vooralleer te beslissen of deze nieuwe procedure uitgevoerd kan worden thuis

☐ De ouder en logopedist vatten het plan voor de komende week samen

Compliance Checklist *(to check whether parents are implementing the treatment per protocol)*

Mini-KIDS

Date treatment session: ..../......../............

Duration of the treatment session: 30 / 60 / ..... minutes

Implementation of the treatment at home was

OR ☐ an agreed practice time at home and lasted ...... minutes. It was performed ... times since the previous treatment session

AND/OR ☐ applying a technique or advice throughout the day

☐ At least one parent attends the treatment session with the child

☐ Parent is involved / participates:

- ☐ Parent reacts and interacts with both SLT and child
- ☐ Parent informs SLT about previous week and home activities/games

☐ Parent talks openly about stuttering and speech in a tolerant style (curious, self-confident, relaxed), using appropriate vocabulary.

☐ Parent uses techniques to reduce communicational stress/pressure/time pressure

☐ Parent completes and hands in observation forms and checklists as agreed upon

☐ Parent uses pseudo stuttering in an easy and self-confident manner according to the treatment phase

☐ Parent handles initiatives, signs of intolerance or resistance of the child in an accepting and agreed upon manner at home (shows good problem-solving skills)

Treatment Fidelity Checklist *(to check whether SLTs are implementing the treatment per protocol)*

Mini-KIDS

☐ The SLT uses pseudo stuttering in a qualitative correct, easy, tolerant, and self-assured manner according to the treatment phase

☐ The SLT talks openly about stuttering, names it using appropriate vocabulary with an open and tolerant attitude

☐ The SLT makes sure the parent is actively involved / participates during the treatment session throughout the treatment process. Active involvement can include active observation, joining in an activity, demonstration ...

☐ The SLT observes that the child is feeling comfortable during the treatment sessions and with the treatment that is implemented

☐ The SLT gives feedback to the parent (and to the child whenever relevant) in a tolerant and encouraging style

☐ The SLT works hierarchical according to each treatment phase (linguistic, emotional, in vitro versus in vivo)

Compliance Checklist *(vérifier si les parents appliquent le traitement conformément au protocole)*

Mini-KIDS

Date séance de traitement: ..../......../............

Durée de séance de traitement : 30 / 60 / ... minutes

Mettre en œuvre le traitement à la maison était

OU BIEN ☐ un temps de pratique convenu à la maison et a duré ...... minutes. Elle a été réalisée... fois depuis la dernière séance de traitement

ET/OU BIEN ☐ appliquer une technique ou un conseil tout au long de la journée

☐ Au moins un parent est présent à la séance de traitement avec l'enfant

☐ Le parent est impliqué / participe:

- ☐ Le parent réagit et interagit avec le logopède et l'enfant
- ☐ Le parent informe le logopède sur le déroulement de la semaine précédente et des activités/jeux à la maison

☐ Le parent parle ouvertement du bégaiement et de la parole dans un style tolérant (curieux, sûr de lui, détendu), en utilisant un vocabulaire approprié.

☐ Le parent utilise des techniques pour réduire la pression sur la communication (la pression temporelle, les questions, la complexité)

☐ Le parent remplit et remet les formulaires d'observation et les listes de contrôle comme convenu

☐ Le parent utilise le pseudo-bégaiement de manière aisée et sûre d'elle, conformément à la phase de traitement

☐ Le parent gère les initiatives, les signes d'intolérance ou de résistance de l'enfant d'une manière acceptante et comme cela a été convenu avec le logopède durant la séance et à la maison (montre de bonnes aptitudes à résoudre les problèmes)

Treatment Fidelity Checklist *pour vérifier si les logopèdes appliquent le traitement en respectant le protocole)*

Mini-KIDS

☐ Le logopède utilise le pseudo-bégaiement de manière qualitativement correcte, facile, tolérante et sûre d'elle en fonction de la phase de traitement

☐ Le logopède parle ouvertement du bégaiement, le nomme, utilise un vocabulaire approprié en adoptant une attitude ouverte et tolérante.

☐ Le logopède s'assure que le parent est activement impliqué / participe à la session de traitement tout au long du processus de traitement. La participation active peut inclure l'observation active, la participation à une activité, une démonstration...

☐ Le logopède observe que l'enfant se sent à l'aise pendant les séances de traitement et avec le traitement mis en œuvre.

☐ Le logopède donne un retour d'information au parent (et à l'enfant si nécessaire) dans un style tolérant et encourageant.

☐ Le logopède travaille de manière hiérarchique en fonction de chaque phase du traitement (linguistique, émotionnelle, in vitro versus in vivo)

Compliance Checklist *(om n ate gaan of de ouders de therapie volgens het behandelprotocol uitvoeren)*

Mini-KIDS

Datum therapiesessie: ..../......../............

Duur therapiesessie: 30 / 60 / ... minutes

Het toepassen van de therapie thuis was

OFWEL ☐ een afgesproken oefenmoment thuis en duurde ...... minuten. Het werd... keer sinds de vorige therapiesessie uitgevoerd

OFWEL ☐ het toepassen van een techniek of advies doorheen de dag

☐ Ten minste 1 ouder volgt de sessie samen met het kind

☐ De ouder is betrokken/ participeert:

- ☐ De ouder reageert en interageert zowel met het kind als met de logopedist
- ☐ De ouder informeert de logopedist over de voorbije week en over de activiteiten of spelletjes die thuis zijn gedaan

☐ De ouder praat openlijk over het stotteren en over spreken, op een tolerante manier (nieuwsgierig, zelfzeker en rustig) en gebruikt hierbij een aangepaste woordenschat.

☐ De ouder gebruikt communicatietechnieken om de tijdsdruk en complexiteit zo laag mogelijk te houden

☐ De ouder voert afgesproken observatieopdrachten uit en dient de betreffende checklists in.

☐ de ouder gebruikt pseudostotteren op een gemakkelijke en zelfzekere manier volgens de betreffende therapiefase

☐ De ouder hanteert (in de therapie én thuis) tekenen van weerstand of intolerantie bij het kind op een accepterende en afgesproken manier (de ouder toont goede probleem-oplossende vaardigheden)

Treatment Fidelity Checklist *(om na te gaan of de logopedist de behandeling per therapie arm volgens het protocol correct uitvoert)*

Mini-KIDS

☐ De logopedist gebruikt pseudostotteren op een kwalitatief correcte manier, met gemak, tolerant en zelfverzekerd volgens de betreffende therapiefase

☐ De logopedist bespreekt het stotteren transparant, benoemt het op een aangepast niveau (woordenschat) met het kind en toont daarbij een open en tolerante attitude

☐ De logopedist betrekt de ouder actief in de therapiesessie, de ouder kan deelnemen in de sessies volgens de betreffende fase. Actieve deelnemen kan ook inhouden dat de ouder observeert, noteert, instapt in een activiteit die al begonnen is, iets demonstreert…

☐ De logopedist bewaakt (observeert) dat het kind zich comfortabel voelt tijdens de therapiesessies en bij de behandeltechnieken die gebruikt worden

☐ De logopedist geeft feedback aan de ouder (en aan het kind waar nodig) op een tolerante en aanmoedigende, motiverende manier

☐ De logopedist werkt zeer geleidelijk volgens de hiërarchie die bij de betreffende therapiefase hoort (linguïstisch, emotioneel, in vitro versus in vivo)

Compliance Checklist *(to check whether parents are implementing the treatment)*

Social-Cognitive Behavioural Therapy (SCBT)

Date treatment session: ..../......../............ (add if there was a parent training session and if it was an individual session or in group)

Duration of the treatment session: 30 / 60 / ..... minutes

Implementation of the treatment at home was

OR ☐ an agreed practice time at home and lasted ...... minutes. It was performed ... times since the previous treatment session

AND/OR ☐ applying a technique or advice throughout the day

☐ At least one parent attends the treatment session with the child

☐ Treatment sessions are scheduled once/two times per week *(indicate which one applies)*

☐ The parent implements techniques during the treatment session to work on making the speaking activities enjoyable (eg., removing time pressure, simplifying language, ...)

☐ The parent implemented techniques to work on making the speaking activities enjoyable (eg., removing time pressure, simplifying language, ...) at home at least 2 times/week for 20-30 minutes for the first 3 months and at least once per week during the next 3 months

☐ The parent observes the interaction during the treatment session a few times and then joins the interaction

☐ The parent demonstrates during the treatment session how s/he implements the treatment at home (eg, praises the child verbally or non-verbally for the PCWS's correct behaviour and speaking model)

☐ If there was a training moment (parent session, evening group session) between the previous treatment session and this treatment session with the child, the parent has attended that training moment (parent session, evening group sessions)

Treatment Fidelity Checklist *(to check whether SLTs are implementing the treatment per protocol)*

Social-Cognitive Behavioural Therapy (SCBT)

☐ The SLT makes sure the parent is actively involved / participates during the treatment session throughout the treatment process. Active involvement can include active observation, joining in an activity, demonstration ...

☐ The SLT observes that the child is feeling comfortable during the treatment sessions and with the treatment that is implemented

☐ The SLT works on one or more of these aspects *(indicate which ones apply)*:

- ☐ Conditioning speaking activities
- ☐ Coginitive training focused on emotions
- ☐ Cognitive training focused on cognitions
- ☐ Desensitisation (emotional training)
- ☐ Skill training (conversation skills, social conversation skills, articulation skills, variation of speech and voice)

☐ The SLT involves the child's teacher in treatment

☐ The SLT involves (an)other significant important person(s) in the child's environment (other than the immediate family) in treatment

French version

Compliance Checklist *(vérifier si les parents appliquent le traitement)*

Social-Cognitive Behavioural Therapy (SCBT)

Date séance de traitement: ..../......../............ (ajoutez s'il y a eu une session de formation des parents et si c'était une session individuelle ou en groupe)
Durée de séance de traitement : 30 / 60 / ... minutes
Mettre en œuvre le traitement à la maison était
OU BIEN ☐ un temps de pratique convenu à la maison et a duré ...... minutes. Elle a été réalisée... fois depuis la dernière séance de traitement
ET/OU BIEN ☐ appliquer une technique ou un conseil tout au long de la journée

☐ Au moins un parent est présent à la séance de traitement avec l'enfant

☐ Les séances de traitement sont prévues une ou deux fois par semaine (*indiquez ce qui s'applique*).

☐ Le parent met en œuvre des techniques pendant la session de traitement pour travailler sur rendre les activités de parole agréables (par exemple, supprimer la pression du temps, simplifier le langage, ...)

☐ Le parent a mis en œuvre des techniques pour travailler sur rendre les activités de parole agréables (par exemple, supprimer la pression du temps, simplifier le langage, ...) à la maison au moins 2 fois par semaine pendant 20-30 minutes pendant les 3 premiers mois et au moins une fois par semaine pendant les 3 mois suivants.

☐ Le parent observe l'interaction pendant la séance de traitement à quelques reprises, puis se joint à l'interaction.

☐ Pendant la séance de traitement, le parent montre comment il met en œuvre le traitement à la maison (par exemple, il félicite l'enfant, verbalement ou non, pour son comportement correct et son modèle d'expression).

☐ S'il y a eu un moment de formation (session parentale, session de groupe du soir) entre la session de traitement précédente et cette session de traitement avec l'enfant, le parent a assisté à ce moment de formation (session parentale, sessions de groupe du soir).

Treatment Fidelity Checklist *(vérifier si les logopèdes mettent en œuvre le traitement conformément au protocole)*

Social-Cognitive Behavioural Therapy (SCBT)

☐ Le logopède s'assure que le parent est activement impliqué / participe à la session de traitement tout au long du processus de traitement. La participation active peut inclure l'observation active, la participation à une activité, la démonstration ...

☐ Le logopède observe que l'enfant se sent à l'aise pendant les séances de traitement et avec le traitement mis en œuvre.

☐ Le logopède travaille sur un ou plusieurs de ces aspects (indiquez ceux qui s'appliquent):

- ☐ Rendre les activités de parole agréable
- ☐ Formation coginitive axée sur les émotions
- ☐ Entraînement cognitif axé sur les cognitions
- ☐ Entraînement émotionnel
- ☐ Entraînement des compétences (compétences de conversation, compétences de conversation sociale, compétences d'articulation, variation de la parole et de la voix)

☐ Le logopède implique l'enseignant de l'enfant dans le traitement

☐ Le logopède fait participer au traitement une ou plusieurs autres personnes importantes de l'environnement de l'enfant (autres que la famille immédiate).

Dutch version

Compliance Checklist *(om te ontroleren of ouders de behandeling toepassen)*

Social-Cognitive Behavioural Therapy (SCBT)

Datum therapiesessie: ..../......../............ (voeg de datum toe indien een ouderstrainingsessie plaatsvond en duid aan of deze individueel of in groep plaats vond)

Duur therapiesessie: 30 / 60 / ... minutes

Het toepassen van de therapie thuis was

OFWEL ☐ een afgesproken oefenmoment thuis en duurde ...... minuten. Het werd... keer sinds de vorige therapiesessie uitgevoerd

OFWEL ☐ het toepassen van een techniek of advies doorheen de dag

☐ Ten minste één ouder is aanwezig bij de behandelingssessie met het kind

☐ Behandelingssessies worden een- of tweemaal per week gepland (*aangeven wat van toepassing is*)

☐ De ouder past tijdens de behandelingssessie technieken toe om spreken aangenaam te maken (bv. de tijdsdruk wegnemen, de taal vereenvoudigen, ...)

☐ De ouder past thuis minstens twee keer per week gedurende 20-30 minuten en de volgende 3 maanden minstens een keer per week technieken toe om spreken aangenaam te maken (bv. de tijdsdruk wegnemen, de taal vereenvoudigen, ...).

☐ De ouder observeert de interactie tijdens de behandelingssessie een paar keer en neemt dan deel aan de interactie.

☐ Tijdens de behandelingssessie laat de ouder zien hoe hij of zij de behandeling thuis toepast (bv. prijst het kind, verbaal of non-verbaal, voor correct gedrag en spraakpatronen).

☐ Indien er een trainingmoment was (sessie met de ouder, in de avond en mogelijk in groep) tijdens de vorige therapiesessie met het kind en deze therapiesessie, heeft de ouder dit trainingsmoment bijgewoond.

Treatment Fidelity Checklist *(controleren of de logopedisten de behandeling volgens het protocol uitvoeren)*

Social-Cognitive Behavioural Therapy (SCBT)

☐ De logopedist zorgt ervoor dat de ouder actief betrokken is / deelneemt aan de behandelsessie gedurende het hele behandelingsproces. Actieve participatie kan bestaan uit actieve observatie, deelname aan een activiteit, demonstratie ...

☐ De logopedist merkt op dat het kind zich op zijn gemak voelt tijdens de behandelsessies en met de behandeling die wordt uitgevoerd.

☐ De logopedist werkt aan een of meer van deze aspecten (geef aan welke van toepassing zijn):

- ☐ Spreekactiviteiten aangenaam maken
- ☐ Coginitieve training gericht op emoties
- ☐ Cognitieve training gericht op cognities
- ☐ Emotionele opleiding
- ☐ Vaardigheidstraining (conversatievaardigheden, sociale conversatievaardigheden, articulatievaardigheden, spraak- en stemvariatie)

☐ De logopedist betrekt de leerkracht van het kind bij de behandeling

☐ De logopedist betrekt een of meer andere belangrijke personen in de omgeving van het kind (andere dan de naaste familie) bij de behandeling.

Compliance Checklist *(to check whether parents are implementing the treatment)*

Maintenance Phase (Three treatment arms)

Date treatment session: ..../......../............

Duration of the treatment session: 30 / ..... minutes

☐ At least one parent attends the treatment session with the child

☐ This treatment session is scheduled with an interval of 2/2/4/4/8/8/16 weeks since previous session *(indicate which one applies)*

☐ The parent is vigilant and reported any (signs of) relapse in case there was a relapse

☐ The parent is able to manage relapse independently

Compliance Checklist (*pour vérifier si les parents appliquent le traitement)*

Phase d'entretien (trois bras de traitement)

Date de la séance de traitement : ..../......../............

Durée de la séance de traitement : 30/... minutes

☐ Au moins un parent assiste à la séance de traitement avec l'enfant.

☐ Cette séance de traitement est prévue à un intervalle de 2/2/4/4/8/8/16 semaines depuis la séance précédente (indiquez ce qui s'applique).

☐ Le parent est vigilant et a signalé tout (signe de) rechute au cas où il y aurait une rechute.

☐ Le parent est capable de gérer les rechutes de manière autonome.

Compliance Checklist *(om te controleren of ouders de behandeling toepassen)*

Maintenance Phase (drie therapie armen)

Datum behandelsessie: ..../......../............

Duur behandelsessie: 30/... minuten

☐ Ten minste één ouder woont de behandelingssessie met het kind bij

☐ Deze behandelingssessie is gepland met een interval van 2/2/4/8/16 weken sinds de vorige sessie (aangeven welke van toepassing is)

☐ De ouder is waakzaam en rapporteerde eventuele (tekenen van) terugval indien er een terugval was

☐ De ouder is in staat om zelfstandig met terugval om te gaan

Treatment Fidelity Checklist *(to check whether SLTs are implementing the treatment per protocol)*

Maintenance Phase (Three treatment arms)

☐ The SLT observes meticously if the treatment effects are still maintained (e.g., the child's speech, parent's implementation of techniques, child's emotions...)

☐ The SLT makes sure the parent reports in detail if there was any (sign of) relapse

☐ The SLT makes sure the parent reports in detail if and how s/he manages relapse if there was any (sign of) relapse

Treatment Fidelity Checklist *(vérifier si les logopèdes mettent en œuvre le traitement conformément au protocole)*

Phase d'entretien (trois bras de traitement)

☐ Le logopède observe méticuleusement si les effets du traitement sont toujours maintenus (par exemple, le discours de l'enfant, la mise en œuvre des techniques par le parent, les émotions de l'enfant...).

☐ Le logopède s'assure que le parent rapporte en détail s'il y a eu une rechute (ou un signe de rechute).

☐ Le logopède s'assure que le parent rapporte en détail si et comment il/elle gère la rechute s'il y a eu un (signe de) rechute.

Treatment Fidelity Checklist *(om te controleren of de logopedisten de behandeling volgens protocol uitvoeren)*

Maintenance fase (alle 3 de therapie armen)

☐ De logopedist observeert nauwgezet of de behandeleffecten nog steeds behouden blijven (bijv. de spraak van het kind, de uitvoering van de technieken door de ouder, de emoties van het kind...)

☐ De logopedist zorgt ervoor dat de ouder gedetailleerd rapporteert of er sprake was van (tekenen van) terugval

☐ De logopedist zorgt ervoor dat de ouder gedetailleerd rapporteert of en hoe hij/zij omgaat met terugval als er sprake was van (tekenen van) terugval

APPENDIX 10. AUTHORISATION OF PARTICIPATING SITES

Appendix 10.1. Required documentation

Before initiating a participating site, the following documents are requested: (1) CV participating SLT and (2) the final feasibility report.

Appendix 10.2. Procedure for initiating/opening a new site

Before a site is activated, at a site initiation visit or at a SLT's meeting, a representative of Thomas More Mechelen-Antwerpen will first review the protocol with the SLTs. The data capture requirements (i.e., eCRFs) is explained during the initiation visit.

During the study, Thomas More Mechelen-Antwerpen and CTC UZ-Antwerpen will employ several methods to ensure protocol and Good Clinical Practice (GCP) compliance and the quality/integrity of the sites' data. The Field Monitor of the CTC UZ-Antwerpen will visit the site to check the completeness of PCWS's records, the accuracy of data capture/data entry, the adherence to the protocol and to GCP, and the progress of enrolment. SLTs must be available to assist the CTC UZ-Antwerpen's Field Monitor during these visits. Continuous remote monitoring of each site's data may be performed by a logging into the online platform and by accessing each centres' eCRF data.

The SLT must maintain source documents for each PCWS in the study, consisting of case and treatment session notes (PCWC' speech-language therapy files) containing demographic and information about the stuttering, and the results of any other relevant tests or assessments. Baseline data on eCRFs must be traceable to these source documents in the PCWS's speech-language therapy file. The SLT must also keep the original informed consent form signed by at least one parent of the PCWS (a signed copy is given to the parent(s) of the PCWS).

The SLT must give the Field Monitor access to all relevant source documents to confirm their consistency with the data capture and/or data entry. See the monitoring plan for detailed discussion of what will be included in the overall monitoring plan of the participating centres.

Appendix 10.3. Participating SLT responsibilities

The following requirements are expected from the participating SLTs:

- Attendance at the training sessions of ICH-GCP, LP, Mini-KIDS and SCBT;
- Attendance at the initiation meetings organised by the Field Monitor;

- Attendance at the SLT-RCT Committee meetings
- Availability for teleconferences, in order to solve practical problems, to answer questions regarding inclusion and exclusion criteria, and to discuss specific items with regard to the completion of the trial;
- Ensuring that the SLT site file is accurately maintained.

APPENDIX 11. SAFETY REPORTING FLOW CHART

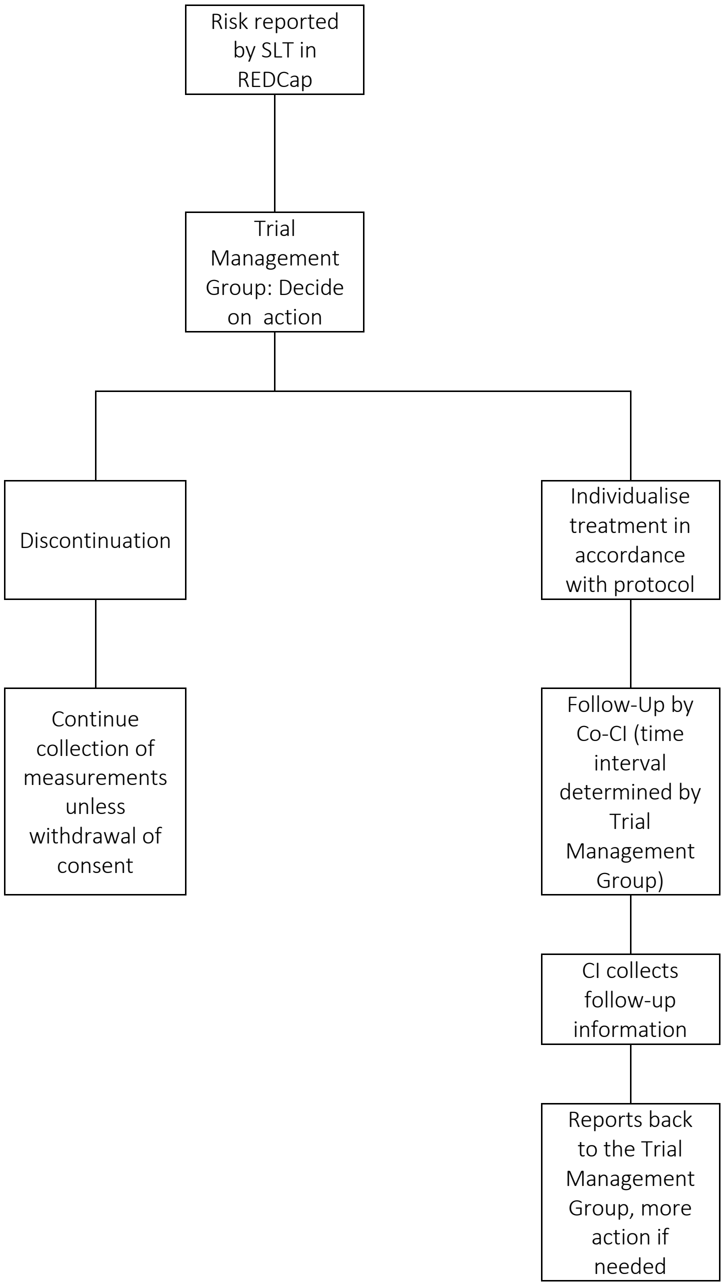

Supplement: S1 Protocol — This version is cleaned. (PDF) [file pone.0304212.s001.pdf]
