# Supplementary material for: Treatment for preschool age children who stutter: Protocol of a randomised, non-inferiority parallel group pragmatic trial with Mini-KIDS, social cognitive behaviour treatment and the Lidcombe Program—TreatPaCS
Source: PLoS One. 2024 Jul 11;19(7):e0304212. doi: 10.1371/journal.pone.0304212 (PMC11239023; doi:10.1371/journal.pone.0304212)
Supplement: S1 File — a. Ethics’ Study Approval in English/Dutch. b. Ethics’ Approval of Main Study Documents. (ZIP) [file pone.0304212.s002.zip › S2b Ethics.pdf]

ETHICAL COMMITTEE

Prof. Dr. Peter Michielsen

Secretariat

03 821 38 97

Mrs Sabine Van Eerdenbrugh

Thomas More University

College Sint-Andriesstraat 2

2000 Antwerp

Project title: TreatPaCS

Treatment for preschool-age children who stutter: a randomised, non-inferiority parallel group pragmatic trial with Mini-KIDS, social cognitive behaviour treatment and the Lidcombe Programme

Project ID 3264 - Edge n/a - BUN B3002022000031 Date:

15/03/2022

FINAL FAVOURABLE OPINION

Dear colleague

The Ethics Committee of the University Hospital Antwerp and the University of Antwerp confirms that the above-mentioned study meets the criteria set in the law of 7 May 2004 and gives a favourable opinion dated 15/03/2022.

The responses were discussed internally on 15/03/2022.

The ethics committee considers that the answers were sufficiently answered.

The following appendices were approved by the Ethics Committee according to ICH-GCP guidelines:

Document Type	File Name	Date	Version
Insurance	Insurance Certificate - 2022	25/11/2021	2022
Other	FINAL_Clinical Trial Agreement_TreatPaCS	10/12/2021	3.0
GCP	GCP Certificate_Chief Investigator_dr Sabine Van Eerdenburgh	20/12/2021	E6R2
Other	FINAL_EN - TreatPaCS - Advice active monitoring	16/02/2022	1.0
Other	FINAL_FR - TreatPaCS - Advice active monitoring	16/02/2022	1.0
Other	FINAL_EN - TreatPaCS - Advice active monitoring	16/02/2022	1.0
Other	FINAL_EN_Instructions videos	21/02/2022	1.0
Other	FINAL_FR_Instructions videos	21/02/2022	1.1
Other	FINAL_NL_instructions videos	21/02/2022	1.1
CV	CV - Chief Investigator_dr Sabine Van Eerdenbrugh_February_2022	22/02/2022	N.A.
Other	FINAL TreatPaCS_Voice overs information video parents	22/02/2022	1.0
Accompanying letter	TreatPaCS_Cover Letter_V1.0 dd 24022022	24/02/2022	1.0
Questionnaire	TreatPaCS_Letter_Location Questionnaires_V1.0 dd 24022022	24/02/2022	1.0
Other	TreatPaCS_Onepager Parent Informed Consent Procedure_V1.0 dd 24022022_Dutch	24/02/2022	1.0
Other	TreatPaCS_Onepager Parent Informed Consent Procedure_V1.0 dd	24/02/2022	1.0

	24022022_English		
Other	TreatPaCS_Onepager Parent Informed Consent Procedure_V1.0 dd 24022022_French	24/02/2022	1.0
Remarks	Protocol _TreatPaCS_INV20-1257_Version 2.2_Tracked Changes	08/03/2022	V2.2
Remarks	Protocol _TreatPaCS_INV20-1257_Version 2.2_Clean	08/03/2022	V2.2
Remarks	TreatPaCS_ICF Child_V1.1_French_English_Dutch_Clean	08/03/2022	V1.1
Remarks	TreatPaCS_ICF Child_V1.1_French_English_Dutch_Tracked Changes	08/03/2022	V1.1
Remarks	TreatPaCS_ICF_V1.3_dd09MAR2022_Dutch_Clean	09/03/2022	V1.3
Remarks	TreatPaCS_ICF_V1.3_dd09MAR2022_Dutch_Tracked Changes	09/03/2022	V1.3
Remarks	TreatPaCS_ICF_V1.3_dd09MAR2022_English_Clean	09/03/2022	V1.3
Remarks	TreatPaCS_ICF_V1.3_dd09MAR2022_English_Tracked Changes	09/03/2022	V1.3
Remarks	TreatPaCS_ICF_V1.3_dd09MAR2022_French_Clean	09/03/2022	V1.3
Remarks	TreatPaCS_ICF_V1.3_dd09MAR2022_French_Tracked Changes	09/03/2022	V1.3
Remarks	TreatPaCS_Reply Letter to EC Initial Submission_V1.1 dd 11032022	11/03/2022	V1.1
Remarks	FR - TreatPACS - Information	11/03/2022	V1.1
Remarks	EN - TreatPACS - Information	11/03/2022	V1.1
Remarks	UK - TreatPACS - Information	11/03/2022	V1.1

This approval is valid for one year from the date mentioned above. Please tell us when the first participant was included, when and why the study was stopped (early) or never started.

If the study is still ongoing after one year, we expect a follow-up report reporting any occurrences.

Finally, we point out that, for studies ongoing at the UZA, serious adverse events should be reported through the incident reporting system.

Kind regards

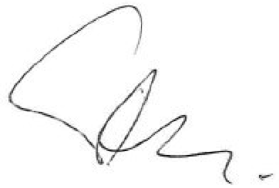

Prof Peter Michielsen

Chairman Ethics Committee UZA/UAntwerp

cc.

FAGG - Research & development department, Place Victor Horta 40 b40 - 1060 Brussels

Meeting Attendee Full Name	Meeting Attendee Qualifications
Ms Bettina Blaumeiser	Physicians
Emeritus Professor Hilde Bortier	MD, PhD
Prof. Dr. Patrick Cras	Vice-Chair, Physicians
Ms Ingrid De Meester	Pharmacologist
Ms Elyne Debaetselier	Nurse
Professor Francois Eyskens	Physician
Ms Lina Fierens	Nurse
Mr Kris Ides	Physiotherapist
Ms Johanna Kwakkel-van Erp	Physicians
Ms Barbara Michiels	General Practitioner
Mr Peter Michielsen	Chair, Physicians
Mr Pieter Moons	Coordinator Bio- and Human Tissue bank
Ms Veerle Schoeters	Nurse
Mr Kris Smulders	Nurse
Mr Guy Van Honste	Patient Representative
Dr Michiel Voeten	Physician
