# Supplementary material for: Treatment for preschool age children who stutter: Protocol of a randomised, non-inferiority parallel group pragmatic trial with Mini-KIDS, social cognitive behaviour treatment and the Lidcombe Program—TreatPaCS
Source: PLoS One. 2024 Jul 11;19(7):e0304212. doi: 10.1371/journal.pone.0304212 (PMC11239023; doi:10.1371/journal.pone.0304212)
Supplement: S1 File — a. Ethics’ Study Approval in English/Dutch. b. Ethics’ Approval of Main Study Documents. (ZIP) [file pone.0304212.s002.zip › S2a Ethics.pdf]

ETHISCH COMITE

Prof. Dr. Peter Michielsen

Secretariaat

03 821 38 97

Mrs Sabine Van Eerdenbrugh

Thomas More Hogeschool

Sint-Andriesstraat 2

2000 Antwerpen

**Project titel: TreatPaCS**

**Treatment for preschool age children who stutter: a randomised, non-inferiority parallel group pragmatic trial with Mini-KIDS, social cognitive behaviour treatment and the Lidcombe Program**

Project ID 3264 - Edge n/a - BUN B3002022000031

Datum: 15/03/2022

**DEFINITIEF GUNSTIG ADVIES**

Geachte collega

Het Ethisch Comité van het Universitair Ziekenhuis Antwerpen en de universiteit Antwerpen bevestigt dat bovenvermelde studie voldoet aan de criteria gesteld in de wet van 7 mei 2004 en geeft een gunstig advies dd. 15/03/2022.

De antwoorden werden intern besproken op 15/03/2022.

Het ethisch comité is van oordeel dat de antwoorden voldoende beantwoord werden.

De volgende bijlagen werden volgens de ICH-GCP richtlijnen door het Ethisch Comité goedgekeurd:

| Document Type | File Name | Date | Version |
|---|---|---|---|
| Insurance | Insurance Certificate - 2022 | 25/11/2021 | 2022 |
| Other | FINAL_Clinical Trial Agreement_TreatPaCS | 10/12/2021 | 3.0 |
| GCP | GCP Certificate_Chief Investigator_dr Sabine Van Eerdenburgh | 20/12/2021 | E6R2 |
| Other | FINAL_EN - TreatPaCS - Advice active monitoring | 16/02/2022 | 1.0 |
| Other | FINAL_FR - TreatPaCS - Advice active monitoring | 16/02/2022 | 1.0 |
| Other | FINAL_NL - TreatPaCS - Advice active monitoring | 16/02/2022 | 1.0 |
| Other | FINAL_EN_Instructions videos | 21/02/2022 | 1.0 |
| Other | FINAL_FR_Instructions videos | 21/02/2022 | 1.1 |
| Other | FINAL_NL_instructies videos | 21/02/2022 | 1.1 |
| CV | CV - Chief Investigator_dr Sabine Van Eerdenbrugh_February_2022 | 22/02/2022 | N.A. |
| Other | FINAL__TreatPaCS_Voice overs information video parents | 22/02/2022 | 1.0 |
| Accompanying letter | TreatPaCS_Cover Letter_V1.0 dd 24022022 | 24/02/2022 | 1.0 |
| Questionnaire | TreatPaCS_Letter_Location Questionnaires_V1.0 dd 24022022 | 24/02/2022 | 1.0 |
| Other | TreatPaCS_Onepager Parent Informed Consent Procedure_V1.0 dd 24022022_Dutch | 24/02/2022 | 1.0 |
| Other | TreatPaCS_Onepager Parent Informed Consent Procedure_V1.0 dd | 24/02/2022 | 1.0 |

| | | | |
|---|---|---|---|
| | 24022022_English | | |
| Other | TreatPaCS_Onepager Parent Informed Consent Procedure_V1.0 dd 24022022_French | 24/02/2022 | 1.0 |
| Remarks | Protocol _TreatPaCS_INV20-1257_Version 2.2_Tracked Changes | 08/03/2022 | V2.2 |
| Remarks | Protocol _TreatPaCS_INV20-1257_Version 2.2_Clean | 08/03/2022 | V2.2 |
| Remarks | TreatPaCS_ICF Child_V1.1_French_English_Dutch_Clean | 08/03/2022 | V1.1 |
| Remarks | TreatPaCS_ICF Child_V1.1_French_English_Dutch_Tracked Changes | 08/03/2022 | V1.1 |
| Remarks | TreatPaCS_ICF_V1.3_dd09MAR2022_Dutch_Clean | 09/03/2022 | V1.3 |
| Remarks | TreatPaCS_ICF_V1.3_dd09MAR2022_Dutch_Tracked Changes | 09/03/2022 | V1.3 |
| Remarks | TreatPaCS_ICF_V1.3_dd09MAR2022_English_Clean | 09/03/2022 | V1.3 |
| Remarks | TreatPaCS_ICF_V1.3_dd09MAR2022_English_Tracked Changes | 09/03/2022 | V1.3 |
| Remarks | TreatPaCS_ICF_V1.3_dd09MAR2022_French_Clean | 09/03/2022 | V1.3 |
| Remarks | TreatPaCS_ICF_V1.3_dd09MAR2022_French_Tracked Changes | 09/03/2022 | V1.3 |
| Remarks | TreatPaCS_Reply Letter to EC Initial Submission_V1.1 dd 11032022 | 11/03/2022 | V1.1 |
| Remarks | FR - TreatPACS - Information | 11/03/2022 | V1.1 |
| Remarks | NL - TreatPACS - Informatie | 11/03/2022 | V1.1 |
| Remarks | UK - TreatPACS - Information | 11/03/2022 | V1.1 |

Deze goedkeuring is geldig tot een jaar na bovenvermelde datum. Wij verzoeken u ons te melden wanneer de eerste deelnemer werd geïncludeerd, wanneer en waarom de studie (vroegtijdig) werd stopgezet of nooit werd opgestart.

Indien de studie nog loopt na een jaar verwachten we een follow-up rapport waarin eventuele voorvallen worden gemeld.

Tot slot wijzen we er op dat, voor in het UZA lopende studies, de ernstige ongewenste voorvallen dienen gerapporteerd te worden via het incidentenmeldingssysteem.

Met vriendelijke groeten

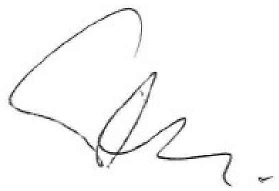

Prof. dr. Peter Michielsen

Voorzitter Ethisch Comité UZA/UAntwerpen

cc.

FAGG - Research & development departement, Victor Hortaplein 40 b40 - 1060 Brussel

| Meeting Attendee Full Name | Meeting Attendee Qualifications |
|---|---|
| Ms Bettina Blaumeiser | Physicians |
| Emeritus Professor Hilde Bortier | MD, PhD |
| Prof. Dr. Patrick Cras | Vice-Chair, Physicians |
| Ms Ingrid De Meester | Pharmacologist |
| Ms Elyne Debaetselier | Nurse |
| Professor Francois Eyskens | Physician |
| Ms Lina Fierens | Nurse |
| Mr Kris Ides | Physiotherapist |
| Ms Johanna Kwakkel-van Erp | Physicians |
| Ms Barbara Michiels | General Practitioner |
| Mr Peter Michielsen | Chair, Physicians |
| Mr Pieter Moons | Coordinator Bio- and Human Tissue bank |
| Ms Veerle Schoeters | Nurse |
| Mr. Kris Smulders | Nurse |
| Mr Guy Van Honste | Patient Representative |
| Dr Michiel Voeten | Physician |
