# Supplementary material for: Treatment for preschool age children who stutter: Protocol of a randomised, non-inferiority parallel group pragmatic trial with Mini-KIDS, social cognitive behaviour treatment and the Lidcombe Program—TreatPaCS
Source: PLoS One. 2024 Jul 11;19(7):e0304212. doi: 10.1371/journal.pone.0304212 (PMC11239023; doi:10.1371/journal.pone.0304212)
Supplement: S2 File — a. Ethics’ Approval Study Extension (recruitment period) in Dutch. b. Ethics’ Approval Study Extension (recruitment period) in English. (ZIP) [file pone.0304212.s003.zip › S3b Ethics.pdf]

ETHICAL COMMITTEE

Chairman

Prof. Dr Peter Michielsen

Secretariat

Tel. 03 821 38

97

Ms Sabine Van Eerdenbrugh CTC

Date: 17/01/2024

Our reference: Project Id 3264 - Edge 002129 - BUN B3002022000031

Project title: TreatPaCS

Dear

The Ethics Committee noted the following report(s)/document(s) in relation to the above study:

Document Type	File Name	Date	Version
Other	TreatPaCS Recruitment extension 15JAN2024	15/01/2024	1

Kind regards

Prof Peter Michielsen

Chairman Ethics Committee UZA/UAntwerp
