# Supplementary material for: Treatment for preschool age children who stutter: Protocol of a randomised, non-inferiority parallel group pragmatic trial with Mini-KIDS, social cognitive behaviour treatment and the Lidcombe Program—TreatPaCS
Source: PLoS One. 2024 Jul 11;19(7):e0304212. doi: 10.1371/journal.pone.0304212 (PMC11239023; doi:10.1371/journal.pone.0304212)
Supplement: S2 File — a. Ethics’ Approval Study Extension (recruitment period) in Dutch. b. Ethics’ Approval Study Extension (recruitment period) in English. (ZIP) [file pone.0304212.s003.zip › S3a Ethics.pdf]

ETHISCH COMITE

Voorzitter

Prof. dr. Peter Michielsen

Secretariaat

Tel. 03 821 38 97

Mevr. Sabine Van Eerdenbrugh

CTC

Datum: 17/01/2024

Onze referentie: Project Id 3264 - Edge 002129 - BUN B3002022000031

Project titel: TreatPaCS

Geachte

Het Ethisch Comité heeft akte genomen van het/de volgende melding(en)/document(en) in verband met bovenvermelde studie:

Document Type	File Name	Date	Version
Other	TreatPaCS Recruitment extension 15JAN2024	15/01/2024	1

Met vriendelijke groeten

Prof. dr. Peter Michielsen

Voorzitter Ethisch Comité UZA/UAntwerpen
