# Supplementary material for: Treatment for preschool age children who stutter: Protocol of a randomised, non-inferiority parallel group pragmatic trial with Mini-KIDS, social cognitive behaviour treatment and the Lidcombe Program—TreatPaCS
Source: PLoS One. 2024 Jul 11;19(7):e0304212. doi: 10.1371/journal.pone.0304212 (PMC11239023; doi:10.1371/journal.pone.0304212)
Supplement: S3 File — a Annual Progress Report Form. b. Ethics’ Notification of Approval Annual Progress Report Form in Dutch. c. Ethics’ Notification of Approval Annual Progress Report Form in English. (ZIP) [file pone.0304212.s004.zip › S4b Ethics.pdf]

ETHISCH COMITE

Voorzitter

Prof. dr. Peter Michielsen

SECRETARIAAT

Tel. 03 821 38 97

Mevr. Sabine Van Eerdenbrugh

CTC

Datum: 13/04/2023

Onze referentie: Project Id 3264 - Edge 002129 - BUN B3002022000031

Project titel: TreatPaCS

Treatment for preschool age children who stutter: a randomised, non-inferiority parallel group pragmatic trial with Mini-KIDS, social cognitive behaviour treatment and the Lidcombe Program

Geachte

Het Ethisch Comité heeft akte genomen van het/de volgende melding(en)/document(en) in verband met bovenvermelde studie:

Document Type	File Name	Date	Version
Other	Annual Progress Report Form_V.1	04/04/2023	1
Other	TreatPaCS_APR_V1.0 dd04042023	04/04/2023	1

Met vriendelijke groeten

Prof. dr. Peter Michielsen

Voorzitter Ethisch Comité UZA/UAntwerpen
