# Supplementary material for: Treatment for preschool age children who stutter: Protocol of a randomised, non-inferiority parallel group pragmatic trial with Mini-KIDS, social cognitive behaviour treatment and the Lidcombe Program—TreatPaCS
Source: PLoS One. 2024 Jul 11;19(7):e0304212. doi: 10.1371/journal.pone.0304212 (PMC11239023; doi:10.1371/journal.pone.0304212)
Supplement: S3 File — a Annual Progress Report Form. b. Ethics’ Notification of Approval Annual Progress Report Form in Dutch. c. Ethics’ Notification of Approval Annual Progress Report Form in English. (ZIP) [file pone.0304212.s004.zip › S4c Ethics.pdf]

Logo UZA

Ethical Committee

President

Prof. dr. Peter Michielsen

Secretary

Tel number 03 821 38 97

Mrs Sabine Van Eerdenbrugh

CTC

Date: 13/04/2023

Our reference: Project id 3264-Edge 002129 – BUN B3002022000031

Project title: TreatPaCS

Treatment for preschool age children who stutter: a randomised, non-inferiority parallel group pragmatic trial with Mini-KIDS, social cognitive behaviour treatment and the Lidcombe Program

Dear

The Ethics Committee has noted the following report(s)/document(s) in connection with the above-mentioned study:

Document type	File name	Date	Version
Other	Annual Progress Report Form_V.1	04/04/2023	1
Other (°)	TreatPaCS_APR_V1.0 dd04042023	04/04/2023	1

(°) This document was the quarterly newsletter that we wanted to send to our contacts

Kind regards,

Signature

Prof. dr. Peter Michielsen
President Ethical Committee UZA/UAntwerp
