# Supplementary material for: Treatment for preschool age children who stutter: Protocol of a randomised, non-inferiority parallel group pragmatic trial with Mini-KIDS, social cognitive behaviour treatment and the Lidcombe Program—TreatPaCS
Source: PLoS One. 2024 Jul 11;19(7):e0304212. doi: 10.1371/journal.pone.0304212 (PMC11239023; doi:10.1371/journal.pone.0304212)
Supplement: S3 File — a Annual Progress Report Form. b. Ethics’ Notification of Approval Annual Progress Report Form in Dutch. c. Ethics’ Notification of Approval Annual Progress Report Form in English. (ZIP) [file pone.0304212.s004.zip › S4a Ethics.pdf]

Annual Progress Report to Leading Ethics Committee

1. Details of Coordinating Investigator

Name:	Sabine Van Eerdenbrugh
Address:	**Thomas More**<br>Logopedie & Audiologie<br>Sint-Andries | Sint-Andriesstraat 2 | BE-2000 Antwerpen
Telephone:	+32 (0)3 432 40 40
E-mail:	sabine.vaneerdenbrugh@thomasmore.be

2. Details of study

Full title of study:	Treatment for preschool age children who stutter: A randomised, non-inferiority parallel group pragmatic trial with Mini-KIDS, social cognitive behaviour treatment and the Lidcombe Program
Name of leading Ethics Committee:	Ethisch Comité UZA/UA
LEC reference number:	3264
Date of favourable ethical opinion:	15/03/2022
Sponsor:	Thomas More
EudraCT/BUN number:	Edge n/a- BUN B3002022000031

3. Commencement and termination dates

Has the study started in Belgium?	Yes
If yes, what was the actual start date in Belgium?<br>Start date is defined as the date that the initial approval was obtained by the Ethical Committee.	25/02/2022
If no, what are the reasons for the study not commencing in Belgium?	/
What is the expected start date?	/

Has the study started in other country's?<br><br>If yes, what was the actual start date in each country?	No.<br><br><br>/

Has the study finished?<br>If yes, complete EudraCT database	No.
If no, what is the expected completion date?<br>If you expect the study to overrun the planned completion date this should be notified to the LEC for information	1/05/2026
If you do not expect the study to be completed, give reason(s)	/

4. Site information

Number Belgian research sites proposed in original application:<br><br>Number of Belgian research sites recruited to date:	30<br><br><br>30
Do you plan to increase the total number of Belgian sites proposed for the study?<br>The addition of any new sites not listed in the original application to the LEC should be notified by submitting a substantial amendment.	No

5. Recruitment of participants

Number of participants recruited:	70
Number of participants completing trial:	62

Number of withdrawals from trial to date due to:
- a. withdrawal of consent: 8
- b. loss to follow-up:
- c. death (where not primary outcome): 0

Total study withdrawals: 8

Number of treatment failures to date (prior primary outcome) due to:
- a. Adverse events: 0
- b. Lack of efficacy: 0

Total treatment failures: 0

Have there been any serious difficulties in recruiting participants?	Yes
If yes, give details:	Slower recruitment rate: fewer requests for treatment for stuttering in young children than expected, more refusals to participate in the study than expected
Do you plan to increase the planned recruitment of participants into the study?<br><br>Any increase in planned recruitment should be notified to the LEC as a substantial amendment for ethical review.	Yes, we closed a non-active site and opened four new sites recently. We expect that this has an impact on the recruitment rate.

6. Safety reports

Have there been any Suspected Unexpected Serious Adverse Reactions (SUSARs) in this trial in Belgium?	No
Have these SUSARs been notified to the Ethics Committee within 7/15 days?<br><br>If no, please arrange urgently and give reasons for late notification.	/
What is the reporting date for periodic safety reports to the LEC during this trial?<br><br>This is the date of first authorization of the trial in any EU member state or, if the sponsor is the Marketing Authorisation Holder, the International Birth Date for the product.	NA
Has the Annual Safety Report (ASR) been submitted?	NA
When is the next ASR due?	NA

7. Amendments

Have any substantial amendments been made to the trial during the year?	Yes
If yes, please give the date and amendment number for each substantial amendment made.	-17.03.2022 : first amendment, protocol 2.3<br>-31.03.22 : second amendment, protocol 2.4<br>-10.06.22 : third amendment, protocol 2.5

8. Serious breaches of the protocol or Good Clinical Practice

Have any serious breaches of the protocol or GCP occurred in relation to this trial during the year?<br><br>Under the Clinical Trials Regulations, all serious breaches must be notified to the LEC/n-LEC(s) and Competent Authorities within 7 days of the matter coming to the sponsors attention.	No
If yes, please give the date (in 'dd/mmm/yyyy' format) of each notification to the Competent Authorities.<br><br>Please provide the LEC/n-LEC(s) with a copy of each notification for information (unless previously notified)	

9. Other Issues

Are there any other developments in the trial that you wish to report to the Committee?<br><br>Are there any ethical issues on which further advice is required?<br><br>If yes to either, please attach separate statement with details.	No<br><br>No

10. Declaration

Signature of Coordinating Investigator:	Sabine Van Eerdenbrugh Digitaal ondertekend door Sabine Van Eerdenbrugh Datum: 2023.04.04 13:44:09 +02'00'
Print name:	Sabine Van Eerdenbrugh
Date of submission:	04.04.2023
