# Supplementary material for: Treatment for preschool age children who stutter: Protocol of a randomised, non-inferiority parallel group pragmatic trial with Mini-KIDS, social cognitive behaviour treatment and the Lidcombe Program—TreatPaCS
Source: PLoS One. 2024 Jul 11;19(7):e0304212. doi: 10.1371/journal.pone.0304212 (PMC11239023; doi:10.1371/journal.pone.0304212)
Supplement: S4 File — a. KCE Review process. Review process applied to the protocol for funding. b. Agreement between KCE and Thomas More University of Applied Sciences. (ZIP) [file pone.0304212.s005.zip › S5b Agreement between KCE and Thomas More University.pdf]

DocuSign Envelope ID: DA3FAC93-0EAC-4BE8-8867-AF054C945F27

AMENDMENT AND RESTATEMENT AGREEMENT

relating to a
RESEARCH AGREEMENT DATED 15 February 2022

between

BELGIAN HEALTH CARE KNOWLEDGE CENTRE

- and -

THOMAS MORE MECHELEN-ANTWERPEN VZW

THIS AMENDMENT AND RESTATEMENT AGREEMENT is entered into on the date of the last signature with retroactive effect to 15 February 2022 (the "**Amendment Effective Date**") and is made by and between:

BELGIAN HEALTH CARE KNOWLEDGE CENTRE, located at Administrative Centre Botanique, Doorbuilding (10th Floor), Boulevard du Jardin Botanique 55, B-1000 Brussels, Belgium, with registration number 0872.876.076 (hereinafter referred to as "**KCE**")

And

THOMAS MORE MECHELEN-ANTWERPEN VZW, located at Zandpoortvest 60, 2600 Mechelen, Belgium, with registration number BE0455.411.733 (hereinafter referred to as "**Contractor**")

KCE and Contractor may, from time to time, be hereinafter referred to individually as a "**Party**" or collectively as the "**Parties**".

***

WHEREAS, KCE and Contractor entered into a Research Agreement regarding KCE Trial 20-1257, "Treatment for preschool age children who stutter: a randomised, multicentre, non-inferiority parallel group pragmatic trial with Mini-KIDS, Social-Cognitive Behaviour Treatment (SCBT) and the Lidcombe Program (LP) with 249 children (TreatPaCS)" (Clinicaltrials.gov reference: NCT05185726), on 15 February 2022 ("**Original Research Agreement**");

WHEREAS, Contractor who is and remains the Sponsor of this trial, collaborates with UZA, Artevelde Hogeschool and Université de Liège ("**the Contractor's Partners"**"). These 4 parties will enter into a Consortium Agreement ("**Consortium Agreement**") to organise their collaboration and the ownership of the Results and Foreground intellectual property (IP) resulting from the Study, in accordance with this Agreement;

WHEREAS, the Original Research Agreement limits the ownership of the Results to the Contractor while it is the intention of the Consortium to work together and share the ownership of the Results and Foreground IP;

WHEREAS, with effect from the Amendment Effective Date, the Parties wish to amend and restate the Original Research Agreement to include mutually agreed changes to the terms of the Original Research Agreement, on and subject to the terms and conditions set forth in this Amendment and Restatement Agreement;

WHEREAS, this Amendment and Restatement Agreement only aim to acknowledge the collaboration between the Partners of the Consortium, shared ownership of the Study Results and shared Foreground IP without prejudice of the other provisions of the Original Research

Agreement, in particular regarding the Contractor responsibility as Sponsor of the Study or the financial arrangements;

AGREED TERMS

1. AMENDMENT AND RESTATEMENT

1.1. In consideration of the mutual promises contained in the Original Research Agreement, with effect from the Amendment Effective Date, KCE and Contractor agree to amend and restate the Original Research Agreement in the form included in Annex 1 hereto ("**Restated Research Agreement**"), such that the rights and obligations of the Parties to the Original Research Agreement shall, on and from the Amendment Effective Date, be governed by and construed solely in accordance with the provisions of the Restated Research Agreement. The Parties hereby agree to comply with the terms of the Restated Research Agreement on and from the Amendment Effective Date.

2. GENERAL

2.1. This Amendment and Restatement Agreement and any matter, dispute or claim arising out of or in connection with it or its subject matter or formation shall be governed by and construed in accordance with the laws of Belgium.

IN WITNESS WHEREOF, KCE and Contractor have caused this Agreement to be executed in two (2) originals by their respective duly authorized officers.

BELGIAN HEALTH CARE KNOWLEDGE CENTRE (KCE)

	For acknowledgement:
Maria Eyssen (Signature) Digitally signed by Maria Eyssen (Signature) Date: 2022.07.22 21:02:32 +02'00'	France Vrijens (Signature) Digitally signed by France Vrijens (Signature) Date: 2022.07.12 16:14:14 +02'00'
By ______________________	By ______________________
Print Name: Marijke Eyssen, MD	Print Name: France Vrijens
Title: General Director a.i.	Title: Head of KCE Trials Programme
Date:	Date:

THOMAS MORE MECHELEN - ANTWERPEN vzw

Digitaal ondertekend door Stijn Coenen (Signature)
Datum: 2022.08.17 14:31:02 +02'00'

By ______________________________

Print Name: Stijn Coenen

Title: President TMMA

Date:

Marijke Lemal (Signature)
Digitally signed by Marijke Lemal (Signature)
Date: 2022.08.25 22:35:03 +02'00'

By ______________________________

Print Name: Marijke Lemal

Title: Manager Unit Research & Consultancy

Date:

For acknowledgement:

Sabine Van Eerdenbrugh
Digitaal ondertekend door Sabine Van Eerdenbrugh
Datum: 2022.08.26 09:30:01 +02'00'

By: ______________________________

Print Name: Sabine Van Eerdenbrugh

Title: Chief Investigator

Date:

For purposes of this Agreement, a document (or signature page thereto) signed and transmitted by electronic means is to be treated as an original document. The signature of any Party on any such document, for purposes hereof and thereof, is to be considered as an original signature, and the document transmitted is to be considered to have the same binding effect as an original signature on an original document.

ANNEX 1

RESTATED RESEARCH AGREEMENT

DocuSign Envelope ID: DA3FAC93-0EAC-4BE8-8867-AF054C945F27

RESEARCH AGREEMENT

BETWEEN

BELGIAN HEALTH CARE KNOWLEDGE CENTRE
("KCE")

AND

Thomas More Mechelen - Antwerpen vzw
("CONTRACTOR")

IN PRESENCE OF :

Artevelde Hogeschool, having its office in Hoogpoort 15, 9000 Gent, VAT number BE0474.120.360, duly represented herein by Tomas Legrand, President
(hereinafter referred to as "Artevelde Hogeschool" or "Partner")

Université Liège, having its office in Patrimone de l'Université de Liège, Place de 20-Août, 4000 Liège, VAT number BE0325.777.171, duly represented herein by M. Pierre Wolper, Recteur-Président
(hereinafter referred to as "ULiège" or "Partner")

Universitair Ziekenhuis Antwerpen (UZA), having its office in Drie Eikenstraat 655, 2650 Edegem, VAT number BE0874.619.603, duly represented herein by Prof. dr. Marc Peeters, who entrusts the performance of UZA's activities under this Agreement to UZA's Clinical Trial Centre
(hereinafter referred to as "CTC UZA" or "Partner")

hereinafter referred to collectively as "**the Contractor's Partners**"

Version number: 2.0

11/07/2022

DocuSign Envelope ID: DA3FAC93-0EAC-4BE8-8867-AF054C945F27

Table of Contents

I. RESEARCH AGREEMENT 8

1. DEFINITIONS AND INTERPRETATION 9
2. PERFORMANCE OF THE STUDY 14
2.1 General 14
2.2 Administration and Direction of the Study 15
2.3 Study Team and Collaborators 18
3. FINANCIAL TERMS 19
3.1 Fee and Payment 19
4. ACCESS TO STUDY DATA, REPORTING, MONITORING 20
4.1 Access to Study Data 20
4.2 Obligation to inform and report – acceptance of the Final Report 21
4.3 Follow-up and governance 22
5. DATA PROTECTION 23
5.1 General obligations 23
6. CONFIDENTIALITY 24
6.1 Confidentiality and non-use 24
7. RIGHTS AND OBLIGATIONS RELATED TO CONTRACTOR BACKGROUND IP 25
7.1 Ownership of Contractor Background IP 25
7.2 Exploitation of Contractor Background IP 25
8. RIGHTS AND OBLIGATIONS RELATED TO RESULTS 25
8.1 Ownership of Results 26
8.2 Protection of Results 26
8.3 Exploitation of Results 26
8.4 Dissemination of Results – Open Access 27
9. ACCESS RIGHTS 29
9.1 Background 29
9.2 Results 29
10. PUBLICATION BY KCE 30
10.1 General30
10.2 Publishing activities 30
11. WARRANTIES 31
11.1 Both Parties warranties 31
11.2 Contractor warranties 32
12. LIABILITY AND INDEMNIFICATION 33
12.1 KCE 33
12.2 Contractor 33
12.3 Limitations and information 33

DocuSign Envelope ID: DA3FAC93-0EAC-4BE8-8867-AF054C945F27

13. INSURANCE 34
14. TERM AND TERMINATION 35
14.1 Term 35
14.2 Termination 35
14.3 Termination Consequences 36
15. FORCE MAJEURE AND HARDSHIP 37
16. GENERAL PROVISIONS 38
16.1 Severability 38
16.2 Assignment 38
16.3 Relationship 38
16.4 Publicity 38
16.5 Entire Agreement 39
16.6 Headings 39
16.7 Further Assurance 39
16.8 Waiver 39
16.9 KCE approval or consent 39
16.10 Costs 39
16.11 Language and Notices 39
16.12 Anti-corruption 40
16.13 Freedom of Information ("Openbaarheid van Bestuur" / "Publicité de l'Administration") 40
16.14 Transparency 41
17. APPLICABLE LAW, ESCALATION PROCEDURE AND DISPUTE RESOLUTION 41
17.1 Applicable law 41
17.2 Dispute resolution 42
SCHEDULE 1: BACKGROUND IP 47
SCHEDULE 2: Description of the Study and Timetable 48
SCHEDULE 3: Budget and Payment Schedule 51
A. BUDGET 51
B. PAYMENT SCHEDULE 52
SCHEDULE 4: Reporting Schedule 60
SCHEDULE 5: Study Team, Collaborators (including Partners of the Consortium), External vendors and subcontractors 61

I. RESEARCH AGREEMENT

This Research AGREEMENT ("**Agreement**") is made as of the date of last signature below (the "**Effective Date**"), by and between:

BELGIAN HEALTH CARE KNOWLEDGE CENTRE, located at Administrative Centre Botanique, Doorbuilding (10th Floor), Boulevard du Jardin Botanique 55, B-1000 Brussels, Belgium, with registration number 0872.876.076 (hereinafter referred to as "**KCE**")

and

THOMAS MORE MECHELEN-ANTWERPEN VZW, located at Zandpoortvest 80, 2600 Mechelen, Belgium, with registration number BE0455.411.733 (hereinafter referred to as "**Contractor**")

KCE and Contractor may, from time to time, be hereinafter referred to individually as a "**Party**" or collectively as the "**Parties**".

IN PRESENCE OF :

Artevelde Hogeschool, having its office in Hoogpoort 15, 9000 Gent, VAT number BE0474.120.360, duly represented herein by Tomas Legrand, President
(hereinafter referred to as "Artevelde Hogeschool" or "Partner")

Université Liège, having its office in Patrimone de l'Université de Liège, Place de 20-Août, 4000 Liège, VAT number BE0325.777.171, duly represented herein by M. Pierre Wolper, Recteur-Président
(hereinafter referred to as "ULiège" or "Partner")

Universitair Ziekenhuis Antwerpen (UZA), having its office in Drie Eikenstraat 655, 2650 Edegem, VAT number BE0874.619.603, duly represented herein by Prof. dr. Marc Peeters, who entrusts the performance of UZA's activities under this Agreement to UZA's Clinical Trial Centre (hereinafter referred to as "CTC UZA" or "Partner")

hereinafter referred to collectively as "**the Contractor's Partners**"

BACKGROUND

WHEREAS, KCE has set up a programme of practice oriented clinical studies in order to generate information and data that are immediately useful to patients, the clinical practice (effectiveness) and policy decision makers (efficiency) (the "**Programme**"); and

WHEREAS, in the framework of the Programme, KCE has selected Contractor, who accepted, to undertake, a non-commercial clinical study entitled "Treatment for preschool age children who stutter: a randomised, multicentre, non-inferiority parallel group pragmatic trial with Mini-KIDS, Social-Cognitive Behaviour Treatment (SCBT) and the Lidcombe Program (LP) with 249 children (TreatPaCS) in accordance with the award letter of KCE dated October 14th 2021 and the terms and conditions of this Agreement under supervision of the Chief Investigator.

WHEREAS, Contractor and its Partners will enter into a Consortium Agreement (the "Consortium Agreement") that will establish the terms and conditions of a consortium program and must be in accordance with this Agreement;

WHEREAS, the Consortium agreement defines rules for the collaboration and synchronisation of activities, including on data management, common approaches towards standardisation, links with regulatory activities and commonly shared dissemination activities, and for the settlement of internal disputes;

WHEREAS, the Parties and the Contractor's Partners wish to acknowledge that the ownership of the Study Results and Foreground IP is shared between the Partners of the Consortium without prejudice of any other provisions of Research Agreement, including but not limited to provision on the Contractor responsibility as Sponsor of the Study and the provisions on the financial arrangements;

NOW, THEREFORE, in consideration of the premises and the mutual promises and covenants expressed herein, the Parties agree as follows:

1. DEFINITIONS AND INTERPRETATION

As used in this Agreement the following terms and expressions shall have the meaning shown below:

1.1 "**Access Rights**" means the right to use Results and/or, where applicable, Contractor Background IP under the terms and conditions laid down in this Agreement.

1.2 "**Agreement**" means this present research agreement, together with its schedules attached hereto, as the same may be modified or amended from time to time as permitted hereunder.

1.3 "**Approval**" means the approvals, favourable opinions, authorisations of the competent Regulatory Authorities or the applicable ethics committee and/or compliance with other procedures required under the applicable laws and regulations in order to commence and/or conduct the Study.

1.4 "**Budget and Payment Schedule**" means the schedule for the budget and the payment of the Fee as set out in Schedule 3.

1.5 "**Business Day**" means a day other than Saturday, Sunday and bank holidays in Belgium.

1.6 **"Chief Investigator"** means the investigator or approved successor, identified in Schedule 5, responsible for supervising the general conduct of the Study.

DocuSign Envelope ID: DA3FAC93-0EAC-4BE8-8867-AF054C945F27

1.7 "**Collaborator**" means a third party person who or third party organisation that works with the Contractor on the Study being formalized under this Agreement (including collaborating centres in a multicentre trial, partners in the context of a consortium agreement, but also third party service providers , that support the performance of the Study with scientific input or certain management or logistic services). For avoidance of doubt, any External vendor shall not be deemed a Collaborator.

1.8 "**Commencement Date**" means the commencement date of the Study as set out in Schedule 2 or, if later, the date upon which all necessary Approvals for the commencement of the Study have been obtained.

1.9 **"Commercialisation"**: means any use of the Results that supports the generation of revenue including but not limited to:

(a) the use of the Results for marketing a product or process or creating and providing a service, including the granting of (sub)licenses;

(b) any use in support of the development, promotion or use of a product or tool that will be made available on a fee paying basis;

1.10 "**Completion Date**" means the date on which the Study and such other activities in relation thereto (such as the completion of the Final Report and main manuscript), are completed, as confirmed by KCE in writing.

1.11 "**Confidential Information**" means information of any form, however conveyed and irrespective of the media on which it is stored, that is:

(a) information which has been designated as confidential by either Party; or

(b) information that reasonably ought to be considered as confidential including information which relates to the business, affairs, properties, assets, trading practices, goods/services, developments, trade secrets, Intellectual Property, know-how, personnel, customers and suppliers and commercial sensitive information of either Party; or

(c) Personal Data.

1.12 "**Consort Statement**" means the Consolidated Standards of Reporting Trials 2010 guideline, intended to improve the reporting of parallel-group randomised controlled trial, enabling readers to understand a trial's design, conduct, analysis and interpretation, and to assess the validity of its results; the Consort Statement can be found at http://www.consort-statement.org/consort-2010.

1.13 "**Contractor Background IP**" means any Intellectual Property owned or controlled by the Contractor or, where applicable, its Collaborator(s) that is identified as being required for the undertaking of the Study at the Commencement Date as set out in Schedule 1 or that is otherwise used in the performance of the Study.

1.14 "**Contractor's Collaboration Agreement**" means the agreement(s) between the Contractor and its Collaborator(s).

1.15 "**Contractor IP Policy**" shall have the meaning as set out in Section 8.2.2.

1.16 "**Controller**" means the natural or legal person, public authority, agency or other body which, alone or jointly with others, determines the purposes and means of the Processing of Personal Data.

1.17 "**Effective Date**" shall have the meaning as set out above in this Agreement.

1.18 "**External vendor**" means a third party person who or third party organisation that

sells or makes available goods or services used by the Contractor or Collaborator for the conduct of the Study without further supporting the performance of the Study with scientific input or certain management or logistic services. External vendors are listed in Schedule 5.

1.19 "**FAMHP**" means *'het Federaal Agentschap voor Geneesmiddelen en Gezondheidsproducten' / 'Agence Fédérale des médicaments et des produits de santé'*.

1.20 "**Fee**" means the total consideration payable by KCE to Contractor (or, where applicable, to a Collaborator), in accordance with the Payment Schedule, for the performance of the Study and the granting of the rights by Contractor to KCE under this Agreement.

For the avoidance of doubt, the collection and analysis of any biomarkers and any genetic markers of patients in this Study shall, unless specifically otherwise agreed in writing by the Parties, not be covered by the Fee and shall be funded by the Contractor's own internal research funds; no compensation shall be due by KCE to the Contractor for said collection and analysis.

1.21 "**Final Report**" means the final report as identified in the Reporting Schedule.

1.22 "**Foreground IP**" means Intellectual Property that is, or has been created, exemplified or developed (whether in whole or in part) during the course and for the purpose of the Study, including for the avoidance of doubt Intellectual Property generated by a Collaborator or a member of the Study Team.

1.23 "**Good Research Practice**" means standards, practices, methods and procedures conforming to the applicable laws and regulations and the degree of skill and care, diligence, prudence and foresight which would reasonably and ordinarily be expected from a skilled and experienced person or body engaged in a similar type of undertaking under the same or similar circumstances.

1.24 **"ICH E3 Guidelines"** means the ICH Harmonized Tripartite Guideline regarding the Structure and Content of a Clinical Study Reports, a copy of which can be found at http://www.ich.org/products/guidelines/efficacy/efficacy-single/article/structure-and-content-of-clinical-study-reports.html or such updated versions thereof.

1.25 **"ICH E6 GCP**" means the ICH Harmonized Tripartite Guideline for Good Clinical Practice together with such other good clinical practice requirements as are specified in Directives 2001/20/EC and 2005/28/EC of the European Parliament and the Council relating to medicinal products for human use and in guidance published by the European Commission pursuant to such Directives;

1.26 "**Informed Consent Form**" means a document drafted to obtain the consent of a Study Subject, prior to their participation in a Study, approved by the appropriate ethics committee.

1.27 "**Intellectual Property**" **("IP")** means all patents, rights to inventions, copyright and related rights, trademarks and trade names, rights to goodwill or to sue for passing off, rights in designs, database rights, rights in Confidential Information (including in know-how) and any other intellectual property rights, in each case whether registered or unregistered and including all applications (or rights to apply) for, and renewals or extensions of, such rights and all similar or equivalent rights or forms of protection which subsist or will subsist now or in the future in any part of the world.

1.28 "**Joint Controllers**" means two or more Controllers jointly determining both the

DocuSign Envelope ID: DA3FAC93-0EAC-4BE8-8867-AF054C945F27

purposes and the means of the Processing to be carried out.

1.29 “**Law of 2004**” means the Belgian law of 7 May 2004 concerning experiments on the human person, published in the Belgian State Gazette on 18 May 2004, as such law may be amended and/or replaced from time to time (including, but not limited to, as applicable, by the Clinical Trials Regulation (EU) 536/2014, and the corresponding Belgian laws and regulations further implementing said Regulation).

1.30 “**Long Stop Date**” means the date by which the Study should have been effectively commenced as detailed in Schedule 2.

1.31 “**Long-Term Extension Study**” means the additional clinical study to be conducted by Contractor under the Agreement, which (i) is designed to evaluate the long-term safety, efficacy and quality of the Study product(s) and (ii) requires the submission of a new protocol and, where applicable, any additional Approvals.

1.32 “**Material**” means any report, executive summary, paper, abstract or other document provided by the Contractor under Section 4.2. For the avoidance of doubt this means the copyright in such reports, summaries and papers but shall not extend to Results, Foreground IP or other Intellectual Property described therein.

1.33 “**Personal Data**” means any information relating to an identified or identifiable natural person (“**Data Subject**”); for the purposes of this definition, an “identifiable natural person” means a person who can be identified, directly or indirectly, in particular by reference to an identification number or to one or more factors specific to his physical, physiological, mental, economic, cultural or social identity.

1.34 “**Personal Data Protection Legislation**” means the EU General Data Protection Regulation (Regulation EU 2016/679) (the “**GDPR**”) and all other existing or new applicable laws relating to Personal Data protection.

1.35 **“Principal investigator”** means the investigator or approved successor responsible for the conduct of the Study at a clinical trial site;

1.36 “**Prioritisation Group**” means the group composed of independent experts in clinical trials in part delegated by the KCE Board members, who advise and offer their expertise to the KCE Trials programme. They advise on the competing proposals that are within scope and score them according to their relevance and importance for the Belgian health care system. Final funding decision making however remains with the KCE Board.

1.37 “**Processor**” means a natural or legal person, public authority, agency or any other body which Processes Personal Data on behalf of the Controller.

1.38 “**Processing**”, “**Process(es)**”, “**Processed**” means any operation or set of operations which is performed upon Personal Data or on sets of Personal Data, whether or not by automatic means, such as collection, recording, organization, structuring, storage, adaptation or alteration, retrieval, consultation, use, disclosure by transmission, dissemination or otherwise making available, alignment or combination, restriction, erasure or destruction.

1.39 “**Protocol**” means the scientific document for the conduct of the Study, to be drafted by or on behalf of Contractor and approved by KCE before commencement of the Study, including any subsequent amendment thereto.

1.40 “**Pseudonymised Personal Data**” means Personal Data that can no longer be attributed to a specific Data Subject without the use of additional information, provided that such additional information is kept separately and is subject to technical and

DocuSign Envelope ID: DA3FAC93-0EAC-4BE8-8867-AF054C945F27

organisational measures to ensure that the Personal Data are not attributed to an identified or identifiable natural person.

1.41 “**Quality Management System**” means the systems and processes established by Contractor to ensure that the Study is conducted and the Results are generated in accordance with ICH/GCP, the Protocol and applicable laws and regulations.

1.42 “**Regulatory Authority**” means the statutory or governmental bodies with authority under the laws of the territory where the Study (or part of it) is conducted in relation to clinical studies, including the relevant ethics committees with responsibility for clinical studies, the European Medicines Agency (EMA) the FAMHP and the Belgian Data Protection Authority.

1.43 “**Reporting Schedule**” means the reporting schedule as set out in Schedule 4.

1.44 “**Results**” means (i) the Study Data and (ii) any specimens, minutes of meetings, methods, know how, discoveries, inventions and all other information in tangible form generated, acquired, discovered, conceived, developed or otherwise arising out of the Study (including the trial master file, subject diaries and adverse event reporting forms); whatever its form or nature, whether it can be protected or not, as well as any rights attached to it, including Foreground IP.

1.45 “**Steering Committee**” shall have the meaning as set out in Section 4.3.1 (b).

1.46 “**Study**” means the main clinical study to be conducted by Contractor under this Agreement and further described in Schedule 2; for the avoidance of doubt, for the purpose of this Agreement, “Study” shall be understood as also including such additional services or work to be performed by Contractor, before, during or after the clinical study, as agreed by the Parties.

1.47 “**Study Data**” means (i) the Study Protocol and any amendments thereto, (ii) the cleaned and locked electronic database used for the main analysis including any and all pseudonymized clinical and non-clinical data collected or generated in the performance of the Study, (iii) the annotated case report forms (CRFs) including an explanation of all variables and coding conventions used in de database, (iv) the log of changes made to the database starting from the CRF completion to database lock, (v) any and all Study reports identified in the Reporting Schedule and (vi) any and all data generated at a later stage (e.g. based on exploratory analyses of samples collected in the performance of the Study), but not included in the main analysis and Final Report (for the sake of clarity, this data shall be provided to KCE as an addendum as soon as becoming available). For the avoidance of doubt, (i) Study Data shall not include any patient health records used to help complete the CRFs and (ii) a Long-Term Extension Study shall be considered a separate study with separate Study Data.

1.48 “**Study End Date**” means, unless agreed otherwise between the Parties and detailed in the Protocol, the date on which the last visit by the last Study Subject according to the Protocol (including follow-up visits) has been completed under the Study, as detailed under Schedule 2.

1.49 “**Study Site(s)**” means the location(s) where the Study shall be conducted in accordance with this Agreement as listed in Schedule 5, which may be updated from time to time upon the Parties’ mutual consent.

1.50 “**Study Subject**” means an individual who is participating in the Study.

1.51 “**Study Team**” means those individuals appointed by the Contractor and, where applicable, the Collaborator to conduct the Study, of which the key members are identified in Schedule 5 and which may be updated from time to time.

1.52 “**Timetable**” means the timelines listed in Schedule 2.

1.53 “**Use**” shall have the meaning as set out in Section 7.2.1.

1.54 The interpretation and construction of this Agreement shall be subject to the following provisions:

(i) a reference to any law, regulation or other similar instrument shall be construed as a reference to the law, regulation or instrument as subsequently amended or re- enacted;

(ii) references to Sections and Schedules are to sections of and schedules to this Agreement;

(iii) where the context allows, references to male gender include the female gender and the neuter, and the singular includes the plural and vice versa;

(iv) references to a Party shall include that Party's personal representatives, successors or permitted assignees; and

(v) general words are not to be given a restrictive meaning because they are followed by particular examples, and any words introduced by the terms “including”, “include”, “in particular” or any similar expression will be construed as illustrative and the words following any of those terms will not limit the sense of the words preceding those terms.

2. PERFORMANCE OF THE STUDY

2.1 General

2.1.1 *General requirements.* The Contractor shall, alone or (subject to Section 2.3) with Collaborators, perform the Study at the Study Site(s) in accordance with (i) Good Research Practice, (ii) the Protocol, (iii) ICH/GCP, (iv) the most recent version of the World Medical Association Declaration of Helsinki, (v) all applicable laws and regulations (including but not limited to the Law of 2004, the Law of 19 December 2008 related to the collection and use of human materials, the Law of 22 August 2002 related to the rights of patients, the law of 7 May 2017 on clinical trials with medicinal products for human use, the law of 22 December 2020 regarding medical devices, including their implementing Royal Decrees), (v) applicable Personal Data Protection Legislation and (vi) the terms and conditions of this Agreement. The Contractor shall install and maintain during the entire term of the Study an adequate Quality Management System.

2.1.2 *Responsibility as Study sponsor.* Contractor shall act as sponsor of the Study, as defined in the Law of 2004, and shall assume all responsibilities and liabilities in connection therewith and procure the mandatory liability insurance coverage in accordance with the Law of 2004. Contractor shall ensure that it shall be mentioned in the Protocol, the Informed Consent Forms and in other relevant communication with the Study Subjects or the Regulatory Authorities as sponsor of the Study. Contractor acknowledges and agrees for the avoidance of doubt that KCE shall under no circumstances be considered as sponsor of the Study or assume any responsibilities or liabilities in connection therewith, and Contractor shall make no representations whatsoever in this respect.

2.1.3 *Conflict of interest.* (a) Contractor must take all measures to prevent any situation where the impartial and objective performance of the Study is compromised for reasons involving any conflicting interests. Contractor shall notify KCE promptly in writing of any situation constituting or likely to lead to a conflict of interests and immediately take all steps to rectify this situation.

(b) Without prejudice to the generality of the foregoing, Contractor shall, and shall ensure that any member of the Study Team and (where applicable) any Collaborator shall, during the term of the Agreement, adhere to the Timetable and to conduct the Study in a timely manner without delay or hindrance.

2.1.4 *Long-Term Extension Study*. To extent applicable and requested by KCE, and unless expressly agreed otherwise between the Parties, Contractor shall perform any Long-Term Extension Studies in accordance with the terms and conditions set out in this Agreement.

2.2 Administration and Direction of the Study

2.2.1 *Scope and objectives.* The scope and objectives of the Study are set out in Schedule 2. The day-to-day conduct of the Study shall be under the control of Contractor, in consultation, as appropriate, with KCE.

2.2.2 *Timetable.* In conducting the Study, Contractor shall use best efforts to comply with the Timetable. The Timetable may be modified upon both Parties' written consent as a result of (i) force majeure, (ii) unforeseen requirements of KCE, or (iii) delays in obtaining or rejection or revocation of or changes in the Approvals mentioned in Section 2.2.3, for reasons for which Contractor is not responsible; or (iv) for any other good reason agreed in writing between the Parties. If at any time, Contractor has reasons to believe that it will not be able to comply with the Timetable, Contractor shall inform KCE as soon as possible.

2.2.3 *Approvals.* Contractor shall be responsible to obtain and maintain any and all Approvals before the commencement of the relevant activity which is subject to the Approval. Contractor shall keep evidence of any such Approvals before the commencement of the relevant activity which is subject to said Approval and make such evidence available to KCE upon KCE's request.

2.2.4 *Protocol.* Before commencement of the Study, Contractor shall submit the Protocol to KCE. KCE shall have the right to suggest changes to the Protocol, which Contractor shall implement unless it has reasons (e.g. Contractor or the investigator reasonably believes that the suggested change is not in the best interest of the Study Subjects), in which event Parties shall, giving full consideration to Contractor's scientific independence, discuss in good faith how to solve the matter. Any further amendment of the Protocol intended to be made by the Contractor shall also be subject to the prior review of KCE and, as the case may, the application of the aforementioned procedure. KCE shall have the right to request an amendment of the Protocol, which Contract only may refuse for reasons as referred to above.

Before any amendment of the Protocol, KCE and Contractor shall agree on the impact on the Timetable whenever relevant. It being understood that any such amendments shall not have an impact on the Budget. Both Parties shall not unreasonably withhold their consent on any amendment intended to be made by Contractor or requested by KCE. Where required under applicable law and regulations, Contractor shall obtain the Approvals or inform the competent Regulatory Authorities in relation to such amendment. For the avoidance of doubt, no such proposed amendment to the Protocol shall be effective unless all Approvals shall have been obtained. The

DocuSign Envelope ID: DA3FAC93-0EAC-4BE8-8867-AF054C945F27

Contractor shall comply with any amendment to the Protocol requested by a Regulatory Authority or ethics committee and the amended Protocol will then be provided to KCE for review.

2.2.5 *Study Subjects.* (a) Contractor shall ensure that the recruitment of the Study Subjects shall take place in accordance with the approved Protocol and the agreed Timetable.

(b) In the context of this Study, and unless otherwise agreed between parties, Contractor shall not collect any additional patient data, samples or any type of information other than those described in the Protocol.

(c) Contractor shall ensure that all Study Subjects are properly informed of the nature, implications and risks of the Study in accordance with all applicable laws and regulations, including ICH/GCP. Contractor shall ensure that each Study Subject has understood and signed (or that, in case of minors or protected adult, their parent(s) or guardian have signed) the required Informed Consent Forms before their participation in the Study. Contractor shall inform the Study Subjects, via the Informed Consent Form (or in the absence thereof, via any other means of providing information regarding the Processing of Personal Data), that (i) the main purpose of the Study is to improve clinical practice and the (Belgian) health care system, (ii) the Study Data may be used in accordance with Section 4.1 by KCE, and/or any other governmental department, institution, body, office, public service and/or agency for further non-commercial research purposes and/or for health care purposes (the latter purpose to be understood as the use with the aim to improve clinical practice and the health care system and/or to design, evaluate, and/or implement policies or programmes in connection with or related to health care, health economics, pharmacoeconomics and/or social security) and (iii) their aggregated Pseudonymised Personal Data may be published for public interests. Contractor shall also inform the Study Subjects, via the Informed Consent Form (or in the absence thereof, via any other means of providing information regarding the Processing of Personal Data), that their Pseudonymised Personal Data may be transferred by KCE to any public health insurance agencies (including, but not limited to, RIZIV-INAMI or their EU counterpart) or governmental department, institution, body or office (for example, the EU counterpart of KCE) within the European Economic Area for further data analyses and/or use for further non-commercial research purposes and/or for health care purposes (the latter purpose to be understood as the use with the aim to improve clinical practice and the health care system and/or to design, evaluate, and/or implement policies or programmes in connection with or related to health care, health economics, pharmacoeconomics and/or social security). Contractor shall submit the final draft Informed Consent Form (or in the absence thereof, any other means of providing information regarding the Processing of Personal Data) to KCE for review as well as any amendments to the Informed Consent Form. KCE shall provide the Pseudonymised Personal Data only to public health insurance agencies (including, but not limited to, RIZIV-INAMI or their foreign counterpart) or governmental departments, institutions, bodies or offices (for example the foreign counterpart of KCE) outside the European Economic Area, if the relevant country is deemed to offer adeaquate Personal Data protection in accordance with article 45 of the GDPR; in such event, KCE and Contractor shall collaborate to adequately draft or, where applicable, amend the Informed Consent Form (or in the absence thereof, any other means of information regarding the Processing of Personal Data) in this respect. For the avoidance of doubt, KCE shall not provide Pseudonymised Personal Data to any health insurance agencies or governmental department, institution, body or office unless for further data analyses and/or use for further non-commercial research purposes and/or for health care purposes (the latter purpose to be understood as the use with

DocuSign Envelope ID: DA3FAC93-0EAC-4BE8-8867-AF054C945F27

the aim to improve clinical practice and the health care system and/or to design, evaluate, and/or implement policies or programmes in connection with or related to health care, health economics, pharmacoeconomics and/or social security).

(d) Contractor shall ensure to have the Study at the Study Site supervised and to have the Study Subjects monitored in such a way in order to ensure at all times the integrity, health and welfare of the Study Subjects.

(e) Contractor shall ensure that all Informed Consent Forms will contain wording that clearly specifies that (i) the Study may be discontinued at all times and with immediate effect, and that (ii) any such discontinuation shall not entitle the Study Subject to any compensation. Before deciding on discontinuation, KCE and Contractor will always take into account medical and ethical considerations and the safety of the Study Subjects.

2.2.6 If, in accordance with the Protocol, the Contractor intends to use the Study Subjects' national registry number (*rijksregisternummer/numéro national*) to link with certain data (such as RIZIV-INAMI billing data), Contractor shall first discuss with KCE and KCE shall provide reasonable assistance to Contractor to obtain approval by the federal chamber of the Information Security Committee and Contractor shall ensure that the Study Subjects have given their unambiguous consent with such use in the Informed Consent Form and that such use shall be implemented in strict compliance with applicable laws and regulations. The use of Study Subjects' national registry number may *inter alia* require the appointment of a trusted third party. As long as the approval of the federal chamber of the Information Security Committee has not been obtained, the national registry number should be kept at the investigator site.

If, in accordance with the Protocol, KCE intends to use the Study Subjects' national registry number (*rijksregisternummer/numéro national*) to link with certain data (such as RIZIV-INAMI billing data), KCE shall obtain approval by the federal chamber of the Information Security Committee. Contractor shall ensure that the Informed Consent Form is updated with this information so that Study Subjects can give their unambiguous consent with such use and that such use can be implemented in strict compliance with applicable laws and regulations. The use of Study Subjects' national registry number may *inter alia* require the appointment of a trusted third party. As long as the approval of the federal chamber of the Information Security Committee has not been obtained, the national registry number sh ould be kept at the investigator site. KCE will inform the Contractor and/or Chief Investigator at the end of the Study if Study Subjects' national registry number (rijksregisternummer/numéro national) will need to be transferred to the trusted third party.

In this study, the Study Subjects' national registry number will not be used.

2.2.7 *Supplies.* (a) Unless otherwise agreed in writing between the parties, any supplies that are used in the conduct of the Study, including medication or devices, Study products, comparator products (where applicable), and all technical information required to safely administer such products to the Study Subjects shall be supplied or procured by Contractor at its costs, unless the costs of such clinical supplies are eventually assumed by RIZIV-INAMI (or any of its counterparts). In this study, the intervention consists of treatment sessions by Speech Language Therapists (SLTs). The sessions follow the current reimbursement by RIZIV/INAMI except for the first 6 additional sessions in the MiniKIDS and Lidcombe programme which are paid for by the Study budget.

DocuSign Envelope ID: DA3FAC93-0EAC-4BE8-8867-AF054C945F27

(b) Contractor shall ensure that any arrangements made with External vendors/ Subcontractors with regard to the purchasing/supplying of supplies or services for use in the Study comply with the terms and condition of this Agreement

KCE may ask to review any agreements in relation to the Study before they are executed to ensure that they are consistent with the Agreement.

2.2.8 *Information and reporting.* Contractor shall ensure that any member of the Study Team and (where applicable) any Collaborator and any other person involved in the Study, shall comply with the information and reporting requirements set out in Section 4.

2.2.9 *Transparency - Registration of the Study.* (a) Contractor shall comply with all transparency obligations in accordance with all applicable laws and regulations, including but not limited to applicable Personal Data Protection Legislation, Directive 2001/20/EC and Regulation (EU) No 536/2014 (including the registration of the Study, Protocol related data and information and Study Data in public registers and/or databases).

(b) Without prejudice to the generality of Section 2.2.9.(a), Contractor shall register the Study in a publicly accessible registry (such as clinicaltrials.gov) before the Commencement Date.

2.3 Study Team and Collaborators

2.3.1 *General.* (a) The Contractor shall appoint, and shall ensure (where applicable) that any of its Collaborators undertakes to appoint, the necessary qualified personnel, facilities, equipment and supplies to perform the Study under this Agreement. In fulfilling its obligations hereunder, Contractor shall appoint, and shall ensure (where applicable) that any of its Collaborators undertakes to appoint, only persons with the appropriate training, skills and qualifications to perform the Study.

(b) Contractor shall be responsible to ensure that any member of the Study Team and (where applicable) any Collaborator shall comply with the terms of this Agreement and shall promptly advise any such persons or organisations of any changes in the scope of this Agreement or the Study. Contractor shall be liable vis-à-vis KCE for any breach of the Agreement by any of the aforementioned persons or organisations.

Study Team. (a) Before the Commencement Date, Contractor shall store in the trial master file a short curriculum vitae and relevant references of the key members of the Study Team. Contractor shall ensure that no Collaborator shall remove or replace any key member of the Study Team (the initial list of key members is included in Schedule 5) without Contractor's prior written approval, unless the person has left the employ of any of the Collaborators.

(b) The Contractor shall ensure that the agreements with any member of the Study Team contain provisions in respect of Intellectual Property and confidentiality compatible with the terms of this Agreement.

2.3.2 *Collaborators.* (a) Contractor may not involve any Collaborators without the prior written approval of KCE (which approval KCE shall not unreasonably withhold or delay). Together with its request for approval, Contractor shall submit to KCE the final (draft) Contractor's Collaboration Agreement. Contractor shall ensure that the terms and conditions of any such Contractor's Collaboration Agreement complies with the terms and conditions of this Agreement, in particular in respect of arrangements in relation to dissemination of Results and ownership, exploitation and access rights to Results.

DocuSign Envelope ID: DA3FAC93-0EAC-4BE8-8867-AF054C945F27

(b) The Contractor's Collaboration Agreement shall, whenever appropriate and notably when the Contractor is acting in consortium with other study and/or research institutions, provide for the creation of governance structures to decide on the collaboration and synchronisation of activities, including on data management, common approaches towards standardisation, links with regulatory activities and commonly shared dissemination activities, and for the settlement of internal disputes.

2.3.3 *Record keeping.* Without prejudice to the generality of Section 4, the Contractor shall keep and shall cause any member of the Study Team and (where applicable) any Collaborator to keep full, detailed and accurate (electronic) records of all activities performed and Results obtained in connection with the Study and, where relevant, keep laboratory notebooks recording all research, development and other work carried out in respect of the Study.

3. FINANCIAL TERMS

3.1 Fee and Payment

3.1.1 *Fee.* In consideration of the performance of the Study in compliance with this Agreement and the granting of rights as set out in this Agreement, KCE shall pay Contractor the Fee in accordance with the Payment Schedule and the milestones set out therein. The Fee is based on the budget set forth in Schedule 3, which is the result of a detailed estimation of the workload (in hours) involved for the Study and all related activities (see the budget table in Schedule 3). Contractor guarantees to make the necessary human resources available (as foreseen in the budget table) for the performance of each task as part of the Study.

3.1.2 Unless otherwise specifically agreed between the Parties, the Fee shall constitute the full and complete compensation for the performance of the Study and the granting of the rights hereunder and no other or additional amounts shall be due by KCE under or in connection with this Agreement (including no pass-through costs). The Payment Schedule may include specific additional provisions concerning KCE's payment of part of the Fee directly to a Collaborator and containing both Parties' obligations in respect thereto.

3.1.3 *Invoices and payment term.* Contractor shall issue invoices within six (6) months of achievement of the relevant milestone event or milestone date as set out in the Payment Schedule. Prior to issuing any invoice, KCE will send out a Request for Invoice (RFI) or Contractor shall request prior approval to invoice to KCE (which approval KCE shall not unreasonably withhold or delay). Contractor shall send the definitive invoice to the following address **KCE Finances; Kruidtuinlaan 55, Doorbuilding (10th floor); 1000 Brussel; KCE.Finances@kce.fgov.be**. KCE shall pay the invoice in EURO within thirty (30) calendar days from the date of receipt of the invoice.

3.1.4 *Suspension.* KCE may suspend its payment obligations under this Agreement in the event Contractor (or any other person or organisation involved in the Study) does not comply with its material obligations under this Agreement after a remediation period of thirty (30) calendar days following the date of receipt of a written notice by KCE specifying the non-compliance and requiring its remedy.

3.1.5 *Payments to third parties.* Unless and solely to the extent expressly provided otherwise in the Payment Schedule, the Contractor is responsible for payments to third parties, including but not limited to Collaborators, participating sites, ethics committees, Study Subjects, etc. and shall ensure that such payments are made promptly and in accordance with applicable laws and regulations.

DocuSign Envelope ID: DA3FAC93-0EAC-4BE8-8867-AF054C945F27

3.1.6 *Final payment.* The final payment due by KCE on the Completion Date as set out in the Payment Schedule, shall be subject to the following conditions:

(i) the reports (including the trial report) and the main manuscript required under Section 4 and the Reporting Schedule have been submitted by the Contractor to KCE, and have been accepted by KCE according to the procedure set forth in Section 4.2.2(c); and

(ii) agreement has been reached in respect of any items remaining for disposal.

4. ACCESS TO STUDY DATA, REPORTING, MONITORING

4.1 Access to Study Data

4.1.1 Upon submission of the first draft of the Final Report and for a further period of six (6) years, and subject to Section 4.1.1, Contractor shall provide KCE upon KCE's request with the Study Data in the format to be agreed between the Parties. Any such provision of Study Data will not require additional data analysis, unless agreed otherwise. KCE may only access the Study Data for non-commercial health care research purposes. This is to be understood as the use with the aim to improve clinical practice and the health care system and/or to design, evaluate, and/or implement policies or programmes in connection with or related to health care, health economics, pharmacoeconomics and/or social security). For the avoidance of doubt, no access shall be provided to KCE with respect to patient health records.

4.1.2 Contractor shall ensure that the Study Data that are disclosed to KCE or to which KCE has otherwise access to in accordance with this Agreement upon submission of the first draft of the Final Report shall only include Pseudonymised Personal Data. Upon submission of the first draft of the Final Report and pursuant to Section 4.1.1, KCE shall be given a copy (in a format to be agreed upon with KCE) of the Study Data. KCE shall use such copy, in its capacity as independent Controller, in compliance with all applicable Personal Data Protection Legislation. Contractor shall at all times ensure that (i) the unique code concerning such Pseudonymised Personal Data will only be in the possession of the members of the (clinical) Study Team who are in direct contact with the relevant Data Subjects, or a trusted third party (where applicable), (ii) such Pseudonymised Personal Data can only be traced or linked back by said Study Team members, or trusted third party and (iii) said Study Team members, or trusted third party, shall treat these codes as strictly confidential. In relation to the Pseudonymised Personal Data to which KCE is granted access in accordance with this Section, KCE shall, in accordance with Section 5.1.1(b), comply as a separate and independent Controller with all applicable Personal Data Protection Legislation. Notwithstanding the foregoing, the Parties may, on an exceptional basis, mutually agree to disclose any Pseudonymised Personal Data to KCE prior to the submission of the first draft of the Final Report; in that case, the Parties shall discuss in good faith, any amendments required to this Agreement, the Informed Consent Form (or in the absence thereof, to any other means of providing information regarding the Processing of Personal Data) under applicable Personal Data Protection Legislation.

4.1.3 Except if the procedure for publication as set forth in Section 8.4 has been followed, Contractor shall not provide (a copy of) the Study Data to a third party without the prior written approval of KCE, which approval KCE shall not unreasonably withhold or delay and which KCE may subject to specific conditions in order to ensure that the provision of said Study Data does not have a negative impact on the further performance of the Study in accordance with this Agreement, the rights granted to

DocuSign Envelope ID: DA3FAC93-0EAC-4BE8-8867-AF054C945F27

KCE under this Agreement and/or the benefit of the Study for the patients and/or the public payers.

4.2 Obligation to inform and report – acceptance of the Final Report

4.2.1 *Information*. Subject to Section 4.1, Contractor shall during the term of the Study (and for a period of six (6) years thereafter) provide all information on any aspect of the Study as reasonably requested by KCE, allowing KCE to be informed on the progress of the Study and any important event in relation therewith. In addition, Contractor shall inform KCE promptly of (i) any event which is likely to affect significantly or delay the performance of the Study or KCE's interests, (ii) changes in its legal, financial, technical, organisational situation, circumstances affecting compliance with the requirements under this Agreement, (iii) significant developments, including developments in relation to the safety of Study Subjects or to the scientific direction of the Study taking into account the research objectives described in Schedule 2. For the avoidance of doubt, any safety data reporting obligations in accordance with the applicable laws and regulations shall vest in Contractor.

4.2.2 *Reporting*. (a) Subject to Section 4.1, Contractor shall comply with the Reporting Schedule and shall use the format as determined by KCE (as KCE may amend from time to time). Contractor shall provide KCE with high-level progress reports at least twice a month.

(b) Each progress report shall detail all relevant information relating to the Study (including the recruitment of Study Subjects) up to the relevant date.

(c) The Final Report shall, with respect to its content and format, be in compliance with the ICH E3 Guidelines or similar format acceptable to KCE and with the CONSORT Statement (unless KCE has confirmed that said report(s) may be set up pursuant to adapted or simplified standards). The Contractor shall also provide, in a form to be agreed with KCE, a draft summary Final Report. If within one (1) year of the Study End Date the Contractor has not produced the Final Report which satisfies KCE, KCE may prepare and publish, or arrange for the preparation and publication of such a report.

KCE has the possibility to object in writing against or provide written comments on the draft Final Report and/or the draft summary Final Report during a period of two (2) months from the date of receipt. Following objections or comments received from KCE within that two (2) month period, KCE and Contractor shall discuss in good faith on any adjustments to be made to the draft report(s); in any case, CONTRACTOR shall, and shall ensure that any of its Collaborators shall, make those adjustments to the Final Report which are required to ensure compliance with the Protocol. KCE is also allowed to give suggestions for adjustment of the Final Report from a scientific point of view, and Contractor, resp. Collaborator will use its best efforts to take into account such reasonable suggestions as long as such suggested adjustments do not change the scientific conclusion of the findings and do not interfere with the scientific integrity of the findings. Contractor shall, and shall ensure that its Collaborators shall, implement the agreed upon adjustments to said reports as soon as possible after KCE and Contractor having agreed on the adjustments. For the avoidance of doubt, if Contractor has not received any written objections or comments within the above-mentioned two (2) month period, the draft Final Report and the draft summary Final Report, as previously submitted to KCE, will be deemed to be accepted by KCE.

(d) KCE reserves the right to reproduce the findings of the Final Report or to provide a summary of the findings with a reference to the Final Report. In any case, KCE cannot change the scientific conclusions of the findings; notwithstanding the foregoing, KCE,

or any party appointed by it, shall be entitled to perform additional analysis if deemed appropriate by it; as the case may be, KCE may come to different conclusions or findings than the conclusions or findings set forth in the Final Report submitted by (or on behalf of) the Contractor to KCE, provided, however, that KCE shall present these different conclusions or findings as its proper conclusions or findings.

(e) For the avoidance of doubt, the Reporting Schedule may contain additional or more detailed reporting requirements (such as the requirement to submit more elaborate intermediate reports or long-term follow-up reports).

4.3 Follow-up and governance

4.3.1 *Governance.* (a) The Contractor and KCE shall review and discuss the conduct and progress of the Study twice a month by phone and email. A face to face meeting at KCE will be planned within one month if requested by either Party.

(b) Contractor shall install and organise a steering committee ("**Steering Committee**") that shall oversee the performance of the Study and discuss important topics in relation thereto. The Steering Committee shall meet on average 3 times per year or as necessary when adapted to the stage of the trial (set-up, conduct, analysis) once every 6 months ("ordinary meetings") or at such other time as reasonably requested in advance by KCE or deemed necessary by Contractor ("extraordinary meetings"). Its composition is detailed in the Protocol.

KCE shall have the right (but not the obligation) to be present at each Steering Committee meeting as an observer without voting power. Contractor shall provide KCE with a proposed agenda for such meetings at the latest ten (10) Business Days in advance of the meeting; KCE shall have the right to add additional items to the agenda. Contractor shall provide draft meeting minutes at the latest ten (10) Business Days after the meeting to KCE for approval (irrespective whether KCE participated or not to said meeting and which approval KCE shall not unreasonably withhold or delay). The meeting minutes shall be deemed to be accepted by KCE if no comments are raised by KCE in writing within fifteen (15) Business Days after receipt of the minutes.

4.3.2 *Audits.* (a) The Contractor shall provide, and shall ensure that any member of the Study Team and (where applicable) any Collaborator undertake to provide, all reasonable cooperation and assistance at all times during the term of this Agreement and for a period of six (6) years after termination or expiry of this Agreement for the purposes of allowing KCE to obtain the information as is necessary to fulfil KCE's obligations to supply information for parliamentary, governmental, judicial or other regulatory or administrative purposes.

(b) Contractor shall provide, and shall ensure that any member of the Study Team or (where applicable) any Collaborator shall provide, all reasonable cooperation and assistance at all times during the term of this Agreement and for a period of two (2) years after termination or expiry of this Agreement to allow KCE (or its agents) to carry out an audit of the Contractor's or (where applicable) any of its Collaborators' compliance with this Agreement (including all activities, performance, security and integrity in connection therewith), and Contractor's Quality Management System. In this respect, Contractor shall ensure, during business hours and upon giving reasonable prior notice, free access of KCE's independent auditors to Contractor's and (where applicable) any of its Collaborators' facilities and Study Sites, and all relevant information, data and records relevant to the Study, including the trial master file, taking into account Collaborator's and Collaborator's facilities and Study Sites' procedures for access.

KCE and/or KCE's agents performing such audit shall have only access to, and only be allowed to report to KCE, such information as strictly required to verify the compliance with this Agreement. Any such information will be accessed by KCE and/or KCE' agent only after the persons conducting the audit have been informed of and bound by confidentiality provisions and restrictions at least as stringent as those stated in Section 6.1 below.

KCE will inform Contractor of the main conclusions of the audit. Contractor shall use best efforts to undertake corrective and preventive actions, as appropriate, in the best interest of the Study and the Study Subjects.

(c) If, during the term of this Agreement, Contractor becomes aware of a scheduled inspection of the Study at any study Site by a Regulatory Authority, Contractor will immediately inform KCE in writing. At its discretion, KCE may choose to be present during such inspection, unless such inspecting Regulatory Authority opposes to KCE being present during the inspection. Any inspection report made by a Regulatory Authority, relevant to the performance of the Study, will promptly be shared by Contractor with KCE.

4.3.3 *Measures.* The Contractor shall take all measures reasonably requested by KCE in order to ensure compliance with this Agreement and performance of the Study within the research objectives as set out in Schedule 2.

5. DATA PROTECTION

5.1 General obligations

5.1.1 *Controller / Processor.* (a) In relation with any Processing of (Pseudonymised) Personal Data in connection with the performance of the Study by Contractor and, as the case may be any Collaborator; it is the understanding of the Parties that Contractor shall act as Controller and, as the case may be, the relevant Collaborator shall act as Processor.

(b) In relation with any Processing of Pseudonymised Personal Data for research purposes and/or use not related to the performance of the Study, and as included in the Informed Consent Form (or in the absence thereof, in any other means of providing information regarding the Processing of Personal Data) pursuant to Section 2.2.5(b), by the Parties and, as the case may be any other recipient of such Pseudonymised Personal Data; it is the understanding of the Parties that the Parties (and any other recipients of the Pseudonymised Personal Data) shall act as independent Controllers of that Pseudonymised Personal Data.

(c) In the event the Parties would jointly decide on the purposes and the means of the Processing of any Personal Data, and therefore qualify as Joint Controllers, the Parties shall discuss in good faith any amendments required to this Agreement under applicable Personal Data Protection Legislation.

5.1.2 *Obligations Parties.* (a) The Parties undertake that any Processing of Personal Data, as referred to in Section 5.1.1, shall be performed in accordance with all applicable Personal Data Protection Legislation.

(b) in the event a Controller engages a Processor for the Processing of Personal Data on its behalf, Controller and such Processor will, in advance of the Processing, enter into a written agreement to reflect such Processing in accordance with Personal Data Protection Legislation.

DocuSign Envelope ID: DA3FAC93-0EAC-4BE8-8867-AF054C945F27

(c) The Controller shall ensure that any Personal Data shall be treated as confidential at all times including during collection, handling and use, and that the Personal Data (including in any electronic format) shall be stored securely at all times and with all technical and organisational security measures that would be necessary for compliance with Personal Data Protection Legislation. The Controller shall take appropriate measures to ensure the security of all Personal Data and guard against unauthorised access thereto or disclosure thereof or loss or destruction while in its/their custody.

(d) In addition, but without prejudice to the provisions of Section 5.1.2(c), the Controller shall treat, and shall ensure that any member of the Study Team and (where applicable) any Collaborator shall treat, any Personal Data obtained in connection with the Study confidential, in order to ensure that the Data Subjects to whom such Personal Data relates are afforded the data protection and privacy rights to which they are entitled.

(e) No information which would lead to the identification of an individual shall be included in any publications without the prior agreement in writing of the Data Subject concerned. No mention shall be made of individual officers of KCE, nor shall information be included which might lead to their identification, without the prior agreement in writing of KCE.

6. CONFIDENTIALITY

6.1 Confidentiality and non-use

6.1.1 *General.* In respect of any Confidential Information it may receive from the other Party and subject always to the remainder of this Section 6, the receiving Party undertakes to keep secret and strictly confidential and shall not disclose any such Confidential Information to any third party other than those involved in the Study who are bound by similar confidentiality obligations, without the disclosing Party's prior written consent provided that:

(i) the receiving Party shall not be prevented from using any general knowledge, experience or skills which were in its possession prior to the commencement of this Contract; and

(ii) nothing herein shall be so construed as to prevent either party from using data processing techniques, ideas, know-how and the like gained during the performance of this Agreement in the furtherance of its normal business, to the extent that this does not result in a disclosure of any Confidential Information or infringement of any valid Intellectual Property rights of either Party or the unauthorised Processing of any Personal Data; and

(iii) nothing herein shall be so construed as to prevent KCE from exercising its rights granted under this Agreement provided that it complies with all applicable laws and regulations, including applicable Personal Data Protection Legislation.

For the avoidance of doubt, the Contractor shall be entitled to disclose this Agreement to any Collaborator.

6.1.2 *Exception.* The obligation of confidentiality and non-use shall not apply to any Confidential Information received by one Party from the other:

(i) which is or becomes public knowledge (otherwise than by breach of

DocuSign Envelope ID: DA3FAC93-0EAC-4BE8-8867-AF054C945F27

Section 6.1.1);

(iv) which was in the possession of the receiving Party, without restriction as to its disclosure, before receiving it from the disclosing Party;

(v) which is received from a third party who lawfully acquired it and who is under no obligation restricting its disclosure;

(vi) is independently developed without access to the Confidential Information; or

(vii) which must be disclosed pursuant to a statutory, legal or parliamentary obligation placed upon the Party making the disclosure.

6.1.3 *Term.* The obligations of each of the Parties contained in this Section in respect of Confidential Information shall continue until the date on which one or more of the conditions of Section 6.1.2 (other than condition (vi)) applies with respect to such Confidential Information. A failure to comply with this Section, shall constitute a material breach of this Agreement.

7. RIGHTS AND OBLIGATIONS RELATED TO CONTRACTOR BACKGROUND IP

7.1 Ownership of Contractor Background IP

7.1.1 *Principle.* Nothing in this Agreement shall affect Contractor’s or, where applicable, any of its Collaborators’ rights in Contractor Background IP nor imply grant of any license to such Contractor Background IP, unless expressly set out herein.

7.1.2 *Identification.* In the event that Contractor Background IP will be used for the performance of the Study, such Contractor Background IP shall be identified in Schedule 1, including the legal restrictions of which it or its Collaborators are aware that may affect the use of the Contractor Background IP for the purpose of the Study or the rights granted to KCE under this Agreement. Contractor shall on a best effort’s base procure that neither itself nor any of its Collaborators will use any Contractor Background IP that was not identified in Schedule 1 for the performance of the Study.

7.2 Exploitation of Contractor Background IP

7.2.1 *General.* In respect to the Contractor Background IP, Contractor shall, and procures (where applicable) that any Collaborator shall, remain free to license, assign, or otherwise dispose or transfer ownership (“Use”) of such Contractor Background IP provided that Contractor (and, where applicable, any Collaborator) shall pass on its obligations specified under this Agreement (or the Contractor’s Collaboration Agreement), regarding such Contractor Background IP, to the (where applicable) licensee, assignee, transferee or acquirer, including the obligation to pass those obligations on to any subsequent (where applicable) licensee, assignee, transferee or acquirer.

7.2.2 *Notification and objection.* Contractor shall, during the term of the Agreement, notify KCE of such Use in advance and KCE shall be entitled to object to such Use if, in KCE’s reasonable opinion, the Use prevents or limits Contractor’s performance under this Agreement. The foregoing obligation to notify KCE of such Use, shall be without prejudice to the obligation of Contractor set forth in Section 7.2.1, which obligation shall continue to apply after the term of the Agreement.

8. RIGHTS AND OBLIGATIONS RELATED TO RESULTS

8.1 Ownership of Results

8.1.1 *Principle.* The Results are owned by Contractor and its Partners under the Consortium Agreement.

8.2 Protection of Results

8.2.1 *Information.* The Contractor shall inform, and shall ensure (where applicable) that any Collaborator informs, KCE of any Results, including any Foreground Intellectual Property, whether patentable or not, which are capable of exploitation either by direct adoption into the health care service or via commercialisation in a timely manner.

8.2.2 *IP policy.* The Contractor and its Partners will identify, protect and maintain Intellectual Property in accordance with their standard institutional policy ("Contractor IP Policy"), if available. The Contractor will make available a copy of the Contractor IP Policy on the request of KCE, if available. Contractor shall take due consideration of KCE's attitude to the clearly inappropriate use of patents which it considers detrimental to scientific endeavour or to advances in healthcare.

Before the Contractor (or where applicable any Collaborator) decides whether or not to protect the invention by filing a patent application or whether or not to abandon prosecution of the patent application, the Contractor shall communicate such decision to KCE and both Parties shall discuss in good faith how to proceed in the best interest of patients and the public payers.

8.2.3 *Records.* Contractor shall keep proper records showing the description of the Contractor Background IP and Foreground IP generated.

8.3 Exploitation of Results

8.3.1 *General.* Contractor and its Partners acknowledge that the main purpose of the research performed under this Agreement is to generate results that will serve the general public interests, and specifically the interests of the patients and health care payers, and, therefore, undertakes not to exploit the Results in any way that is or could be detrimental to such interests. In this respect, Contractor and its Partners acknowledge the importance of the dissemination of the Results and the Access Rights in accordance with the principles set forth herein. Contractor and its Partners are encouraged to use the Results in further research activities and to generate additional results that could further support the aforementioned interests.

8.3.2 *Commercialisation.* (a) In accordance with the acknowledgements and the principles set forth or referred to in Section 8.3.1, the Commercialisation of the Results is not and should never be the main aim of Contractor or its Partners under this Agreement. Without prejudice to Section 8.4, in the event that a Commercialisation opportunity nevertheless arises, the Contractor and its Partners shall or shall procure (where applicable) that any Collaborator shall inform KCE thereof in advance in writing and shall seek the prior written consent of KCE before it or (where applicable) any Collaborator, as the case may be, makes any commercial use of, or grants to any third party any exploitation rights over the Results and/or transfer, dispose or assigns the Results to another party. Contractor shall or shall procure (where applicable) that any Collaborator shall provide all appropriate details of any proposed Commercialisation, licensing, transfer or assignment arrangements, including but not limited to any deal sheet or commercial terms in circulation, which information KCE shall keep confidential.

(b) KCE shall evaluate such intended Commercialisation against the purpose and interests set forth in Section 8.3.1. KCE shall, acting reasonably, have the right to

DocuSign Envelope ID: DA3FAC93-0EAC-4BE8-8867-AF054C945F27

refuse such approval in the event KCE reasonable believes that the intended Commercialisation, licensing or assignment is likely to have a negative impact on the further performance of the Study in accordance with this Agreement, the rights granted to KCE under this Agreement and/or on the main purpose and/or interests set forth in Section 8.3.1. For example and without limiting the foregoing, such right of refusal shall apply in the event the Contractor intends to grant exclusive rights to third parties hindering or limiting the Access Rights granted to KCE hereunder; or in the event the Commercialisation may lead to the scenario where the government has to pay twice (e.g. for the Research under this Agreement and for the reimbursement of commercial products incorporating or making use of one or more Results). In this respect, KCE shall have the right to subject its approval to specific terms which are reasonable and appropriate in the specific case and which should ensure that the intended Commercialisation has no negative impact on the further performance of the Study in accordance with this Agreement, the rights granted to KCE under this Agreement and/or on the main purpose and/or interests set forth in Section 8.3.1. For example and without limiting the foregoing, such specific terms could include the payment to KCE of a fair compensation and/or conditions concerning the pricing of the commercial product or service incorporating or making use of one or more Results.

(c) KCE shall within thirty (30) Business Days after having received all appropriate details of the proposed Commercialisation or transfer, inform the Contractor and/or Collaborator if KCE agrees to start negociations for a commercialisation agreement with Contractor.

Any such commercialisation agreement shall as a minimum address the allocation of revenue, equity or other benefits arising from the proposed Commercialisation arrangements and rights to use the Foreground IP and/or Results.

8.4 Dissemination of Results – Open Access

8.4.1 *General obligation.* (a) Unless otherwise agreed between the Parties, Contractor must as soon as possible disseminate the Results owned by it and/or (where applicable) any Collaborator and Partners, by disclosing them to the public by appropriate means, including in scientific publications (in any medium). Contractor shall inform and discuss its dissemination strategy with KCE in advance.

(b) The foregoing general obligation does not change the right to protect Results through patent applications in accordance with Section 8.2, the confidentiality obligations in Section 6 or the obligations to protect Personal Data further to Section 5, all of which still apply.

(c) Notwithstanding the foregoing and subject to Section 4.1, the Final Report should be made available for review and comment by KCE in accordance with Section 4.2.2, before the Results are disseminated in accordance with this Section 8.4.

(d) The Contractor must notify KCE prior to any dissemination (including publication) (whether in oral, written or other form) of the Foreground IP or Results or Study Data or of matters arising from the Study. The Contractor shall send one draft copy of the proposed dissemination to KCE at least ten (10) calendar days for an abstract and thirty (30) calendar days for a manuscript before the date intended for dissemination. For the avoidance of doubt, this obligation continues after the end of the Study, for a further period of six (6) years. KCE may object to such dissemination, by giving written notice to the Contractor (a "Confidentiality Notice"), to prevent the dissemination of its Confidential Information, or to delay the proposed dissemination

DocuSign Envelope ID: DA3FAC93-0EAC-4BE8-8867-AF054C945F27

for a maximum of four months after the date of receipt of the Confidentiality Notice if, in its reasonable opinion, such delay is necessary in order to seek patent or similar protection for any Results which are the subject of the intended dissemination. In the event Contractor or (where applicable) any Collaborator intends not to protect the Results it needs to formally notify KCE thereof before the dissemination takes place and Parties shall act as per Section 8.2.2.

A Confidentiality Notice must contain a precise and motivated request for necessary adaptations to the intended dissemination/publication. If such objection has been raised, KCE and the Contractor will discuss how to overcome the justified grounds for the objection on a timely basis (for example by adapting the planned publication and/or by protecting Results before publication). The opposition to the intended dissemination will not be unreasonably continued if both Parties agree that appropriate actions have been taken following the discussion. Confidentiality Notices must be sent within ten (10) calendar days for abstracts and thirty (30) calendar days for manuscripts, after receipt of the draft dissemination/publication. The Contractor shall have the right to proceed with the proposed dissemination/publication if it has not received a Confidentiality Notice within that relevant applicable calendar day period.

Furthermore, Contractor will use its best efforts to take into account any reasonable scientific suggestions from KCE as long as such suggested adjustments do not change the scientific conclusion or the findings and do not interfere with the scientific integrity of the findings; provided, however, that if KCE, on reasonable and objective grounds (e.g. on the basis of additional analysis of the data), disagrees with the scientific conclusions or findings or has reasonable doubts on the scientific integrity of the findings, the Contractor shall not proceed with the dissemination until agreement with respect thereto is found between the Parties. In such cases, KCE and Contractor will consult with the independent methodological experts of the KCE Trials Board to find a solution.

(e) Contractor shall ensure that any dissemination is scientifically correct, objective and unbiased (taking into consideration the primary endpoint(s)).

(f) Contractor shall ensure that the manuscript for publication includes references to the data access plan, as well as the contact details of the person responsible within contractor for the management of third party access to the Study Data, as further set forth in Section 9.2.3.

(g) In the event of a multicentre Study, Contractor shall not, and shall use its best efforts to ensure that its Collaborators shall not, independently publish or otherwise disclose any findings resulting from the Study before publication of the main multicentre publication. In the event the main multicentre publication is not published within eighteen (18) months from the date the Final Report is accepted by KCE in accordance with Section 4.2.2(c), the Contractor and/or the Collaborators shall be entitled to publish the site-specific publication, subject to the procedure and conditions set forth in Section 8.4.1 (d) and (f) above.

8.4.2 The Contractor shall ensure that any dissemination shall acknowledge KCE's financial support and carry a disclaimer as KCE may require or in the absence of direction from KCE a notice as follows:

"*This report is independent research funded by Belgian Health Care Knowledge Centre under the Clinical Trials Programme. The views expressed in this publication are those of the author(s) and not necessarily those of Belgian Health Care Knowledge Centre*."

DocuSign Envelope ID: DA3FAC93-0EAC-4BE8-8867-AF054C945F27

8.4.3 *Open access to scientific publications.* Contractor must ensure open access (free of charge, online access for any user) to all peer-reviewed scientific publications relating to the Results owned by it and/or the Collaborators. In particular it must:

(i) As soon as possible and at the latest on publication, deposit a machine readable electronic copy of the published version or final peer-reviewed manuscript accepted for publication in a repository for scientific publications; moreover Contractor must aim to deposit at the same time the research data needed to validate the Results presented in the deposited scientific publications; and

(ii) Ensure open access to the deposited publication, via the repository at the latest on publication (if an electronic version is available for free via the publisher) or, within six (6) months of publication in any other case.

9. ACCESS RIGHTS

9.1 Background

Access Right. Regarding the Contractor Background IP listed in Schedule 1 as per Section 7.1.2, or any Contractor Background IP not listed in Schedule 1 which was used in the performance of the Study in contravention of Section 7.1.2, Contractor hereby grants, and procures (where applicable) that any Collaborator grants, to KCE a non-exclusive, worldwide, irrevocable, unlimited, royalty-free and transferable Access Rights to Contractor Background IP, with the right to sub-license, to the extent such Contractor Background IP is needed to use the Results in accordance with the license grant in accordance with Section 9.2. KCE shall ensure that any of its sub-licensees complies with the terms and conditions of the Access Rights set forth herein. For the avoidance of doubt, patient health records are not needed to make use of the Results.

9.2 Results

9.2.1 *Access Right.* (a) In furtherance of the main purpose and interests set forth in Section 8.3.1 above, Contractor hereby grants, and procures (where applicable) that any Collaborator grants, to KCE and any other Belgian federal or regional institution, body, office, public service and/or agency at the end of the Study, a non-exclusive, worldwide, irrevocable, unlimited, royalty-free and transferable Access Right to the Results, with the right to sub-license, for any non-commercial research purposes, public health care services purposes, and/or for designing, evaluating, and/or implementing policies or programmes in connection with or related to health care, health economics, pharmacoeconomics and/or social security.

(b) Upon request of KCE after the end of the Study, Contractor shall grant, and procures (where applicable) that any Collaborator shall grant, non-exclusive and royalty-free Access Rights to the Results and to Contractor Background IP to use such Results, to EU or EU member state’s institutions, bodies, offices, public services and/or agencies, for any non-commercial research purposes, public health care services purposes and/or for designing, evaluating and/or implementing policies or programmes in connection with or related to health care, health economics, pharmacoeconomics and/or social security.

(c) The foregoing Access Rights shall include the right to publish, upon consultation with Contractor, any Results for any non-commercial purpose, including any entry in a register of research findings or an individual issue of or a review article in a

DocuSign Envelope ID: DA3FAC93-0EAC-4BE8-8867-AF054C945F27

monograph series prepared on KCE's behalf. The content and timing for such publication will be subject to consultation with the Contractor and will take into account the publication timetables in other peer-reviewed journals and the need to make research findings publicly available as soon as practicable. KCE shall ensure that such dissemination shall acknowledge Contractor as the sponsor (in the sense of the Law of 2004) of the Study.

9.2.2 *Publication.* This does not change the right of KCE to publish any Material, (aggregated) Study Data or other information in relation to the Study received from the Contractor for communication and publicising activities as set out in Section 10.

9.2.3 *Access right to third parties.* After the Completion Date, subject to the provisions of this Agreement (Section 5 and 6), Contractor shall use its best efforts to grant, and procures (where applicable) that any Collaborator shall grant, Access Rights to the Results to third parties on a non-exclusive basis and at fair and reasonable terms. In this respect, Contractor shall establish a data access plan managing the access by third parties to the Results subject to the third party entering into a data use agreement with the Contractor (or the person appointed by it) providing the terms and conditions for such access. Contractor shall appoint a contact person that shall manage the third party access on its behalf. In the event Contractor and the relevant third party are unable to agree on the terms and conditions for the access to the Results, Contractor may request KCE to facilitate the discussions.

9.2.4 *Access to Study Data.* The Parties expliticly agree that any access to Study Data shall be construed in accordance with Section 4.1 of this Agreement.

10. PUBLICATION BY KCE

10.1 General

10.1.1 The research contracted by KCE under this Agreement is open and, subject to the provisions of this Agreement, KCE is entitled to publish details of the selection process, the research objectives, plan and costs and this Agreement.

10.2 Publishing activities

10.2.1 *Communication and publishing activities.* (a) without prejudice to KCE's rights under Section 9 and subject to Sections 5 and 6, KCE may use, for its communication and publicising activities, the Materials and all other deliverables, documents and information in connection with the Study provided by Contractor to KCE.

(b) However, if KCE's use of these Materials, documents and information, would risk compromising Contractor's legitimate interests, the Contractor may request KCE not to use it for said purposes and the Parties shall discuss in good faith an acceptable way to proceed, considering at all times the purpose and the objective of the Study.

(c) KCE's right to use the Materials, documents and information includes: (i) for its own purposes, in particular making them available to persons working for KCE or any other federal, regional, EU or other EU member state institution, body, public service, office or body, and copying or reproducing them in whole or in part, in unlimited numbers; (ii) distribution to the public, in particular publication as hard copies and in electronic or digital format, publication on the internet, as a downloadable or non-downloadable file, broadcasting by any channel, public display or presentation, communicating through press information services or inclusion in widely accessible databases or indexes; (iii) editing or redrafting for communication and publicising activities (including shortening, summarising, inserting other

DocuSign Envelope ID: DA3FAC93-0EAC-4BE8-8867-AF054C945F27

elements (such as meta-data, legends, other graphic, visual, audio or text elements), extracting parts (e.g. audio or video files), dividing into parts, use in a compilation; (iv) translation; (v) giving access in response to individual requests in furtherance of the applicable legislation on freedom of information act (*'openbaarheid van bestuur'*); (vi) storage in paper, electronic or other form; (vii) archiving; and (vii) the right to authorise third parties to act on its behalf or sub-license the modes of use set out above to third parties if needed for the communication and publicising activities of KCE. In any case, KCE's right to use the Materials, documents and information set forth in this Section 10.2.1(c) does in no case allow KCE to change the scientific conclusion of the findings notwithstanding the foregoing, KCE, or any party appointed by it, shall be entitled to perform additional analysis if deemed appropriate by it; as the case may be, KCE may come to different conclusions or findings than the conclusions or findings set forth in the aforementioned Materials, documents and information submitted by (or on behalf of) the Contractor to KCE, provided, however, that KCE shall present these different conclusions or findings as its proper conclusions or findings.

(d) Contractor shall ensure it obtains all necessary approvals from third parties concerned in order to comply with its obligations under this Section. In this respect, Contractor shall, and shall ensure that its Collaborator shall, before the commencement of the Study, have entered into appropriate (employment) agreements with its employees, representatives, agents and personnel, in which such employees, representatives, agents or personnel have assigned or granted to Contractor, resp. Collaborator, such rights in order for Contractor to comply with its obligations under this Section.

(e) Where requested by Contractor, KCE will insert the following information:

> "© - *[year] - [name of the copyright owner]. All rights reserved. Licensed to Belgian Health Care Knowledge Centre under conditions*."

11. WARRANTIES

11.1 Both Parties warranties

11.1.1 Each Party represents and warrants that, to its reasonable knowledge at the Effective Date:

(i) the Party's execution, delivery and performance of this Agreement (a) have been authorised by all necessary corporate action, (b) do not violate the terms of any law, regulation, research standards, or court order to which such Party is subject or the terms of any agreement to which the Party may be subject and (c) are not subject to the consent or approval of any third party;

(ii) this Agreement is the valid and binding obligation of the representing Party, enforceable against such Party in accordance with its terms; and

(iii) such Party is not subject to any pending or threatened litigation or governmental action which could interfere with such Party's performance of its obligations hereunder.

11.1.2 Except as expressly provided in this Agreement, none of the Parties gives any warranties or makes any representations with respect to any of the Foreground IP and/or Contractor Background IP or any products derived from them, or their fitness for any purpose, or that any material produced or supplied by any Party and any processes or techniques used, proposed or recommended by any Party will not

DocuSign Envelope ID: DA3FAC93-0EAC-4BE8-8867-AF054C945F27

infringe the Intellectual Property rights of any person in any country.

11.2 Contractor warranties

11.2.1 The Contractor warrants that:

- it shall use best efforts to devote all resources and efforts as may be necessary for the satisfactory and timely completion of the Study in compliance with the Timetable;
- it has full capacity, power and authority and all necessary licences, permits and consents to assume and fully perform all of its obligations under this Agreement;
- there are no actions, suits or proceedings pending or, to the Contractor's knowledge, threatened against or affecting the Contractor before any court or administrative body or tribunal that might affect the ability of the Contractor to meet and carry out its obligations under this Agreement;
- it shall comply with its obligations under this Agreement, including with the standards for performing the Study set out in Section 2.1.1.
- at the Effective Date is not a party to an agreement which would prevent Contractor from fulfilling its obligations under this Agreement;
- it shall during the term of the Study not enter into any agreement or arrangement which would substantially restrict Contractor's ability to perform the Study;
- it shall during the term of the Study not do any other act which may have a substantial adverse effect on the availability of Study Subjects, including providing services to third parties in relation to a study which would or could recruit the same Study Subjects;
- it shall not enter into any Contractor's Collaboration Agreements in which the Intellectual Property arrangements would adversely affect the Contractor's ability to comply with the terms of this Agreement without the prior consent of KCE, such consent not to be unreasonably withheld or delayed.
- the Study Team will have the expertise in the disease and patient population relevant to the Study and will have the training, information, licenses, approvals or certifications necessary for safely, adequately and lawfully conducting the Study;
- none of the Study Team (including Collaborators) shall be subject to any conflicting obligation or shall have any conflict of interest that may interfere or be perceived as interfering with the performance of the Study or that might impair the validity of the Study Data;
- it shall perform the Study in compliance with all ethical principles, including avoiding fabrication, falsification, plagiarism or other research misconduct;
- unless otherwise agreed in writing by KCE, neither the Contractor nor any of its Collaborators has obtained or will obtain during the term of the Study any other (EU, federal, regional, local or foreign) public (other than from KCE) or private funding for the performance of the Study;
- to the best of its knowledge and belief:

a. subject to the declaration set out in Schedule 1 it is (or, where applicable, any Collaborator is) the legal and beneficial owner of all right, title and interest in and to the Contractor Background IP listed in Schedule 1;

b. it and/or (where applicable) a Collaborator will be the legal and beneficial owner(s) of all right, title and interest in and to the Results and Foreground IP and where reasonable and practicable the Collaborator will own and manage such Foreground IP in accordance with, and subject to the terms of this Agreement; and

c. it has not granted any third party any right in respect of the Foreground IP (other than in accordance with the provisions of this Contract), and has not charged or encumbered and will not charge or encumber any of the same.

12. LIABILITY AND INDEMNIFICATION

12.1 KCE

12.1.1 *KCE indemnification.* Subject to the provisions of Section 12.3, KCE shall indemnify and hold Contractor and its respective officers, directors, employees and agents (each, a "Contractor Indemnified Party") harmless from and against any and all claims, liabilities, lawsuits, threats of lawsuits or other governmental action, or losses suffered, incurred or sustained by any Contractor Indemnified Party, by reason of any claim or proceeding to the extent arising out of or resulting from (a) KCE's negligence or wilful misconduct in the performance of its obligations under this Agreement; (b) KCE's breach of applicable law. Notwithstanding the foregoing, KCE shall not be liable for losses to the extent such losses are caused by the negligence, recklessness, or misconduct of Contractor or breach of any of the terms of this Agreement by Contractor. KCE's obligation to indemnify, defend and hold harmless shall only exist provided that Contractor is not in breach of its duty to comply with KCE's instructions regarding such suit or claim and to keep KCE informed thereof.

12.2 Contractor

12.2.1 *Contractor Indemnification.* Subject to the provisions of Section 12.3, Contractor shall indemnify and hold KCE or any other governmental institution, body, public service or agency and its or their respective officers, directors, employees and agents (each, a "KCE Indemnified Party") harmless from and against any and all claims, liabilities, lawsuits, threats of lawsuits or other governmental action, or losses suffered, incurred or sustained by any KCE Indemnified Party, by reason of any claim or proceeding to the extent arising out of or resulting from (a) any non-compliance of Contractor with any of its obligations or warranties under this Agreement; (b) any breach by Contractor of any agreement between Contractor and Collaborator and/or Study Team; (c) any claims arising out of or in connection with or as a result of the performance of the Study.

12.3 Limitations and information

12.3.1 *Limitation of liability.* Subject to Section 12.3.2 and Section 12.3.3, and except for each Party's indemnification obligations set out in Section 12.1 and 12.2 and Contractor's obligations set out in Section 8.3, each Party's aggregate liability under or in relation to this Agreement will not exceed two times the total amount of the Fee paid or payable by KCE under this Agreement.

12.3.2 *Exclusion indirect damages.* Except and to the extent caused by third party claims or

for breaches of Contractor’s obligations set out in Section 8.3, neither Party shall be liable towards the other for any consequential, special, indirect or punitive damages whatsoever, including but not limited to financial loss, lost profits, loss of opportunity or damage to reputation.

12.3.3 *No exclusion or limitation.* Nothing in this Agreement shall exclude or limit a Party’s liability for personal injury or death or for fraud, fraudulent misrepresentation, wilful misconduct and/or gross negligence or breaches of Section 5 and 6 of this Agreement.

12.3.4 *Notification and management.* The Party wishing to seek indemnification hereunder shall (i) notify the other Party immediately of any third party claim for which it seeks to be indemnified under this Agreement; such notice shall set out sufficient details, to the extent reasonably available, of the legal and factual basis of the claim, together with a first estimate, to the extent reasonably possible, of the amount of the liability, (ii) grant, to the extent permitted by law and to the extent not prohibited by the insurance policy of Contractor and/or Collaborator, to the other Party the sole control of any defence or settlement of such third party claim, provided that the other Party shall not enter into any settlement of any claim for which indemnification is sought which requires an admission of fault by the Party wishing to seek indemnification without that Party’s prior written consent, which consent shall not be unreasonably withheld, (iii) not acknowledge any such third party claim or enter into any settlement negotiations relating thereto without the express prior written approval of the other Party, which shall not be unreasonably withheld, (iv) cooperate fully with the other Party and its agents in defence of the third party claim, and (v) provide, to the extent permitted by law and to the extent not prohibited by the relevant insurance policy, the indemnifying Party with copies of all documents and correspondence from the third party and all other correspondence and documents relating to the third party claim as the indemnifying Party may request and the Party claiming indemnification shall give reasonable cooperation, information and assistance in connection therewith. The Party wishing to seek indemnification hereunder shall have the right to participate in the defence of the third party claim by utilizing attorneys of its choice; however, the Party shall bear all costs associated with its participation.

12.3.5 *Obligation to mitigate.* Notwithstanding any other provision of this Agreement, each Party shall use its reasonable endeavours to mitigate losses it may incur that are covered by indemnities provided by the other Party.

12.3.6 *No assignment of claims against KCE.* Contractor may not assign any of its claims for payment against KCE to any third party, except if approved by KCE.

13. INSURANCE

13.1 Without prejudice to Section 12, the Contractor shall throughout the duration of this Agreement effect and maintain with a reputable insurance company a policy or policies of insurance providing an adequate level of cover in respect of all risks which may be incurred by the Contractor arising out of the Contractor's performance of this Agreement, including the insurance that is required to be taken out as sponsor the Study as set out in the Law of 2004.

13.2 The Contractor shall produce on demand by KCE documentary evidence that any insurance policies required by Section 13.1 are in force.

13.3 The terms or the amount of cover of any insurance shall not relieve the Contractor of any liabilities under the Agreement.

14. TERM AND TERMINATION

14.1 Term

14.1.1 *Term.* Unless otherwise terminated in accordance with the provisions hereof, this Agreement shall be effective as from the Effective Date and shall, subject to Section 14.1.2, automatically expire on the Completion Date.

14.1.2 *Surviving provisions.* Notwithstanding Section 14.1.1, the following provisions shall survive the expiration or termination of the Agreement: Section 4.1 (*Access to Study Data*), Section 4.3.2 (*Audits*), Section 5 (*Data Protection*), Section 6 (*Confidentiality*), Section 7 (*Contractor Background IP*), Section 8 (*Results*), Section 9 (*Access Rights*), Section 10 (*Publication*), Section 12 (*Liability and Indemnification*), Section 14.3 (*Termination Consequences*) and Section 17 (*Applicable Law*), including any cross-references set out in these Sections. The foregoing shall be without prejudice to any other provision of the Agreement that by its nature survives expiration or termination of the Agreement.

14.2 Termination

14.2.1 *Both Parties.* Either Party shall have the right to terminate this Agreement immediately upon written notice, in the event:

(i) Of a material breach committed by the other Party which, if capable of being remedied, is not remedied for a period of thirty (30) calendar days following the date of receipt of a written notice specifying the nature of the breach and requiring its remedy; and/or

(ii) The other Party committing numerous breaches of its duties or obligations under this Agreement which collectively constitute a material breach of this Agreement; and/or

(iii) The other Party is dissolved or liquidated, files or has filed against it a petition under any bankruptcy or insolvency law, makes an assignment for the benefit of its creditors or has a receiver appointed for all or substantially all of its property, or experiences an event analogous to any of the foregoing in any jurisdiction in which any of its assets are situated; and/or

(iv) Such Party is the non-defaulting Party, in case a force majeure event continues in effect for a period of more than three (3) months and/or

(v) Immediately upon giving notice if a Regulatory Authority directs that the Study be terminated or refuses, revokes or cancels any Approval or in the event any Approvals for the commencement of the Study is not given at the latest six (6) months after the Effective Date for reasons outside the reasonable control of the Party wishing to terminate the Agreement; and/or

(vi) Immediately upon giving notice if the safety of the Study Subjects cannot be guaranteed anymore for reasons outside the reasonable control of the Party wishing to terminate the Agreement.

14.2.2 *KCE.* In addition to the termination rights provided herein, this Agreement may be terminated by KCE at any time:

(i) Following thirty (30) calendar days written notice, on reasonable grounds communicated by KCE; reasonable grounds could be a.o.

- significant delays in recruitment (eg – failure to recruit at least 33% of

DocuSign Envelope ID: DA3FAC93-0EAC-4BE8-8867-AF054C945F27

the patients as referred to in the milestone table in Schedule 2- taking into account ethics committee approval and the opening of the sites),

- significant and relevant new evidence has been published that makes the Study irrelevant and unnecessary to be further conducted as discussed and agreed in the KCE Trials PG Group, …

(ii) Immediately upon giving notice if KCE reasonably demonstrates that Contractor or any of its Collaborators has (no longer) the capacity or ability to perform the Study within the research objectives set out in Schedule 2; and/or

(iii) Immediately upon giving notice if Contractor receives any other public (other than from KCE) or private funding for the performance of the Study, unless KCE has given its prior written consent for such additional funding; and/or

(iv) Immediately upon giving notice in the event of substantial non-justified delays in the performance of the Study, including in the event the Study has not effectively commenced by the Long Stop Date; and/or

(v) Upon giving three (3) months written notice, in case of a change of control over the Contractor (as such is defined in the Belgian Code of Companies), provided KCE sends its intention to terminate the Agreement within thirty (30) calendar days after notification of the change of control by Contractor to KCE;

14.2.3 *Suspension*. KCE, upon its sole discretion, may require the Contractor to suspend until further notice the performance of the Study for any of the reasons set out in Sections 14.2.1 and 14.2.2 and in accordance with the notice periods (if any) set out therein. If such suspension exceeds a period of four (4) months, the Parties will consult on the impact of such suspension on the agreed budget for the Study. If no agreement can be reached between the Parties within one (1) month from the initiation of the consultation, either Party will have the right to terminate this Agreement immediately upon giving written notice pursuant to Section 14.2.

14.3 Termination Consequences

14.3.1 *General.* Subject to Section 14.3.2, termination of the Agreement by KCE in accordance with Section 14.2 shall not entitle Contractor to receive any compensation or indemnity by KCE in relation to such termination. Termination or expiration of this Agreement in accordance with the provisions hereof shall be without prejudice to the surviving obligations of the Parties as set out in Section 14.1.2.

14.3.2 *Indemnification by KCE in case of termination.* Except in case of termination by KCE pursuant to Section *14.2.1* (i) and (ii) and *14.2.2* (iii) and (iv), KCE shall indemnify the Contractor from and against all work done in compliance with this Agreement prior to termination and all and any actual and duly documented direct non-cancellable costs and direct costs necessarily incurred by reason or in consequence of the termination provided that the Contractor takes all immediate and reasonable steps to minimise the loss [e.g. pay pro rata for the patients included linked to the next milestone payment]; provided, however, that any such indemnification, when taken together with any sums paid or due or becoming due to the Contractor under this Agreement shall not exceed the total Fee which would otherwise have been payable in the event the Study would have been completed in accordance with this Agreement.

14.3.3 *Accrued rights.* The termination or expiration of this Agreement shall be without prejudice to or affect any rights, action or remedy which shall have accrued before

DocuSign Envelope ID: DA3FAC93-0EAC-4BE8-8867-AF054C945F27

termination or expiration or shall accrue thereafter to any Party.

14.3.4 *Closing-down obligations.* Upon receipt of the termination notice, Contractor shall use all reasonable efforts to incur no further expense and to perform no further work except as is reasonably necessary to close down the Study within the given period. In addition, Contractor shall:

(i) take all necessary steps to cease the conduct of the Study in an orderly and professional manner, without compromising quality, and to minimise the further costs and expenses payable by KCE hereunder; and

(ii) make any declaration to or notify any Regulatory Authority in respect of the completion or early termination of the Study if such declaration is required under any applicable laws and regulations; and

(iii) within six (6) months of the effective date of expiry or termination, prepare and submit to KCE a report on the Study in the form and containing the particulars specified by KCE.

14.3.5 In the event of termination of this Agreement by KCE for other reasons than the gross negligence of Contractor (or any of its Collaborators, including Partners), such termination shall only have effect *ex nunc* and not affect the payments already effected before termination. In the event of termination of this Agreement by KCE for the gross negligence of Contractor (or any of its Collaborators), KCE shall have the right to reclaim part or the entire amount of the payments effected by it before termination.

15. FORCE MAJEURE AND HARDSHIP

15.1 In the event that any Party is delayed in the performance of its obligations under this Agreement by an event of Force Majeure (as defined hereafter), the obligations of the Parties under this Agreement shall remain in suspense until the cause thereof has ceased. "**Force Majeure**" shall include, without being limited to, any of the following: riots, sabotage, acts of war, terrorism or piracy, destruction of essential equipment by fire, explosion, storm, flood or earthquake, and delay caused by failure of power supplied or transport facilities or any other cause beyond the control of the Parties which renders performance of this Agreement impossible.

15.2 Neither of the Parties shall be liable to the other for any loss including but not limited to any damages or abatement of charges whether directly or indirectly caused or incurred by any failure or delay in the performance of its obligations due to Force Majeure.

15.3 If either of the parties shall become aware of Force Majeure which give or are likely to give rise to any failure or delay on its part it shall forthwith notify the other by the most expeditious method then available and shall say how long it is estimated that such failure or delay shall continue.

15.4 Any failure by the Contractor to perform or any delay by either of the Parties in performing its obligations under the Agreement which results from any failure or delay in the performance of its obligations by any person, firm or company with which the Contractor shall have entered into any contract, supply arrangement or sub-contract or otherwise, shall be regarded as a failure or delay due to Force Majeure only in the event that person, firm or company shall itself be prevented from or delayed in complying with its obligations under such contract, supply

DocuSign Envelope ID: DA3FAC93-0EAC-4BE8-8867-AF054C945F27

arrangements or sub-contract or otherwise as a result of Force Majeure.

15.5 If as a result of unforeseen events or developments the performance of this Agreement shall cause inequitable hardship for one or both Parties which runs counter to the aim of this Agreement and which the one Party cannot reasonably and in good faith expect the other Party to tolerate, the Parties will meet and seek, in good faith, to find equitable ways to amend the Agreement in order to re-establish the basic economic balance of this Agreement.

16. GENERAL PROVISIONS

16.1 Severability

16.1.1 If any of the provisions of this Agreement are held to be or rendered void or unenforceable, the Parties agree that the same shall not result in the nullity or unenforceability of the remaining provisions of this Agreement, but that they shall use their best efforts to replace such provision with a valid and enforceable provision which shall achieve, to the extent possible, the economic, business or other purpose of said void or unenforceable provision.

16.2 Assignment

16.2.1 Except as set out in Section 2.3.2, the Contractor shall not sub-contract, transfer or assign the whole or any part of this Agreement or collaborate with any third party in the performance of its obligations under this Agreement without the prior written consent of KCE, which consent KCE shall not unreasonably withhold or delay and which may be subject to such terms and conditions as KCE may specify.

16.2.2 The Contractor shallbe responsible for the acts and omissions of its sub- contractors or Collaborators as though they were its own.

16.2.3 The Contractor shall ensure that, to the extent that they are relevant, and where reasonable to do so, the terms and conditions of this Agreement are incorporated into any sub-contract and that all reasonable steps are taken by it to ensure that its sub-contractors and Collaborators are aware of and adhere to the terms and conditions of this Agreement.

16.3 Relationship

16.3.1 This Agreement does not make any Party the employee, agent, partner or legal representative of the other Party for any purpose whatsoever. No Party is granted any right or authority to assume or create any obligation or responsibility, expressed or implied, on behalf of or in the name of the other Party. In fulfilling obligations pursuant to this Agreement the Contractor shall be acting as an independent contractor.

16.4 Publicity

16.4.1 Before and after the Commencement Date, and prior to the publication of the Results or of matters arising from the Study in accordance with Section 10, the Contractor shall not without the prior written consent of KCE, which shall not be unreasonably refused or delayed, release, or otherwise make available to third parties, any information relating to this Agreement or the Study by means of any public statement, in particular any media announcement or display or by putting on any website or oral presentation to meetings where the Results are likely to be reported by the media. This condition shall not apply where the Contractor has a contractual, legal or similar obligation to publish specific details about the Agreement or the

DocuSign Envelope ID: DA3FAC93-0EAC-4BE8-8867-AF054C945F27

Study.

16.4.2 In the event that the Contractor fails to comply with Section 16.4.1, KCE reserves the right to terminate this Agreement with immediate effect by notice in writing.

16.5 Entire Agreement

16.5.1 This Agreement (including its Schedules) constitutes the full and complete statement of the agreement of the Parties with respect to the subject matter hereof and supersedes all prior agreements, representations, warranties, understandings, relationships, whether written or oral, between the Parties, with respect to the subject matter hereof.

16.5.2 No amendment or variation of this Agreement shall be valid unless made in writing and with both Parties' consent.

16.5.3 In the event of any inconsistencies between the terms of this Agreement and the terms of the Protocol or the schedules or other documents referred to in this Agreement, the terms of this Agreement shall prevail except to the extent that any conflict relates to a clinical or medical matter, in which case the Protocol shall prevail.

16.6 Headings

16.6.1 The Section and sub-section headings in this Agreement are for convenience only and shall not in any way affect the meaning or interpretation of this Agreement.

16.7 Further Assurance

16.7.1 Each Party shall at the reasonable request of the other do or procure the doing of all such further acts, and execute or procure the valid execution of all such documents, as may from time to time be necessary in the requesting Party's reasonable opinion to give full effect to this Agreement and to vest in the requesting Party the full benefit of the assets, rights and benefits to be transferred to the requesting Party under this Agreement.

16.8 Waiver

16.8.1 No delay or omission by either Party hereto to exercise any right occurring upon any non-compliance or default by the other Party with respect to any of the terms of this Agreement shall impair any such right or power or be construed to be a waiver of such right. A waiver by either of the Parties hereto of any of the covenants, conditions or agreements to be performed by the other shall not be construed to be a waiver of any succeeding breach of this Agreement or of any covenant, condition or agreement contained in this Agreement.

16.9 KCE approval or consent

16.9.1 Where KCE's approval or consent is requested as per this Agreement, KCE shall use reasonable efforts to communicate its position to Contractor within thirty (30) Business Days as per Contractor's request, except where this Agreement explicitly provides for a different timeframe for such communication to be given by KCE.

16.10 Costs

16.10.1 Each of the Parties shall pay its own costs incurred in connection with the negotiation, preparation and implementation of this Agreement.

16.11 Language and Notices

16.11.1 *Language.* Contractor explicitly agrees that the Agreement is made in the English language and hereby waives any claim in relation to the use of the English language

in any communication or correspondence from KCE to Contractor in relation to this Agreement.

16.11.2 *Notices.* (a) Any notice required under this Agreement shall be made in English, either by registered mail or an internationally recognised overnight courier to KCE and to Contractor at their respective addresses first above written or as subsequently changed by notice duly given in writing at such addresses. Notwithstanding the above all correspondence with regard to a termination of this Agreement shall be by registered mail or by an internationally recognised overnight courier.

(b) Notices by registered mail are deemed to be given upon receipt. Notices by internationally recognised overnight courier are deemed to be given one Business Day following delivery with such courier.

16.12 Anti-corruption

16.12.1 *Prevention of fraud.* The Contractor shall take all reasonable steps, in accordance with Good Research Practice, to prevent fraud in connection with the receipt of monies from KCE.

(i) The Contractor shall notify KCE immediately if it has reason to suspect that any fraud has occurred or is occurring or is likely to occur.

(ii) If the Contractor or Contractor's staff (or any staff of a sub-contractor or Collaborator) commits fraud in relation to this or any other contract with a governmental institution, body or agency (including KCE), KCE may: (a) terminate the Agreement immediately by giving notice in writing and recover from the Contractor the amount of any proven loss suffered by KCE (or such other governmental institution, body or agency) resulting from the termination, including the cost reasonably incurred by KCE of making other arrangements for the performance of the Study and any additional expenditure incurred by KCE throughout the remainder of the term of the Study; or (b) recover in full from the Contractor any other p r o v e n loss sustained by KCE (or any such governmental institution, body or agency) in consequence of any breach of this Section 16.12.

16.13 Freedom of Information ("Openbaarheid van Bestuur" / "Publicité de l'Administration")

16.13.1 The Contractor acknowledges that KCE is subject to the requirements of the relevant Belgian legislation on the freedom of information ("*Publicité de l'Administration/Openbaarheid van Bestuur*", hereafter "FoI") and shall assist and reasonably cooperate with KCE to enable KCE to comply with these requirements.

16.13.2 The Contractor shall and shall procure that its sub-contractors and Collaborators shall:

(i) transfer to KCE all requests for information that it receives under FoI that in its opinion are for KCE;

(ii) consult KCE where it has any doubt whether the request is for KCE as soon as practicable and in any event within two Business Days of receiving a request for information;

(iii) provide KCE with a copy of all information in its possession or power in the form that KCE requires to be provided within a reasonable period time (and in any case within the timeframe that is required for KCE to comply with its obligations under the FoI) in relation to KCE's request; and

DocuSign Envelope ID: DA3FAC93-0EAC-4BE8-8867-AF054C945F27

(iv) provide all necessary assistance as reasonably requested by KCE to enable KCE to respond to the request for information within the time for compliance set out in the FoI.

16.13.3 KCE shall be responsible for determining at its absolute discretion, and notwithstanding any other provision in this Agreement or any other agreement, whether commercially sensitive information and/or any other information is exempt from disclosure in accordance with the relevant provisions of FoI.

16.13.4 In no event shall the Contractor respond directly to a request for information unless expressly authorised to do so by KCE, unless obliged by law or regulation.

16.13.5 The Contractor acknowledges that KCE may, acting in accordance with the FoI request, have to disclose information concerning the Contractor or the Study:

(i) in certain circumstances without consulting the Contractor; or

(ii) following consultation with the Contractor and having taken their views into account;

provided always that KCE takes reasonable steps, where appropriate, to give the Contractor advance notice, or failing that, to draw the disclosure to the Contractor’s attention after any such disclosure.

16.14 Transparency

16.14.1 KCE shall be responsible for determining at its absolute discretion whether any of the content of this Agreement is exempt from disclosure in accordance with the provisions of FoI.

16.14.2 KCE may consult with the Contractor to inform its decision regarding any redactions but KCE shall have the final decision at its absolute discretion.

16.14.3 KCE may, at its sole discretion, redact information from the Agreement prior to publishing for one or more of the following reasons:

(i) national security;

(ii) information protected by intellectual property law;

(iii) third party or Collaborator confidential information;

(iv) IT security; or

(v) prevention of fraud.

16.14.4 The Contractor shall assist and cooperate with KCE to enable KCE to publish this Agreement.

16.14.5 Notwithstanding any other term of the Agreement, the Contractor hereby gives consent for KCE to (i) publish the Agreement in its entirety, including from time to time any agreed changes to the Agreement, to the general public, (ii) in the event the Contractor has not published the Protocol itself within six (6) months from the Commencement Date, publish the Protocol, as approved by the appropriate ethics committee, on the KCE website and (iii) publish the Final Report on the KCE website, provided that such Final Report shall not contain any (Pseudonymized) Personal Data.

17. APPLICABLE LAW, ESCALATION PROCEDURE AND DISPUTE RESOLUTION

17.1 Applicable law

DocuSign Envelope ID: DA3FAC93-0EAC-4BE8-8867-AF054C945F27

17.1.1 This Agreement shall be governed by and construed in accordance with the substantive laws of Belgium, without taking into account its conflict-of-law rules.

17.2 Dispute resolution

17.2.1 The Parties shall use their best efforts to resolve any dispute or claim arising out of or relating to this Agreement through negotiations between their respective designated representatives in accordance with the following escalation procedure, prior to resorting to any court action:

(i) First Level:

KCE:

France Vrijens

Head of KCE Trials Programme

Contractor:

Marijke Lemal Marijke Lemal (Signature) Digitally signed by Marijke Lemal (Signature) Date: 2022.08.25 22:33:18 +02'00'

Manager Unit Research & Consultancy

If the Parties have not come to an agreement as to the dispute or the claim within twenty (20) Business Days of the matter being raised by either Party to such first level representatives, the dispute shall be notified in writing by either Party to the second level representatives set out below. The Parties shall procure that such representatives use their best efforts to resolve the dispute within ten (10) Business Days of such notice.

Second Level:

KCE:

Marijke Eyssen

General Director (*Directeur Général a.i.- Algemeen Directeur a.i.*)

Contractor:

Stijn Coenen

Algemeen Directeur Thomas More-Hogeschool

In the event that such attempts should fail or in the event that any delay would cause irreparable harm to a Party, then the Parties hereto agree to submit such dispute to the exclusive jurisdiction of the Courts of Brussels, Belgium, and agree same shall be subject to Belgian law with the exclusion of its conflict of law rules to the extent that these would refer to foreign law.

17.2.2 Notwithstanding the foregoing, any Party may seek immediate injunctive or other interim relief from any court of competent jurisdiction with respect to any matter for

DocuSign Envelope ID: DA3FAC93-0EAC-4BE8-8867-AF054C945F27

which monetary damages would not adequately protect such Party's interests.

[REMAINDER OF THE PAGE INTENTIONALLY LEFT BLANK]

IN WITNESS WHEREOF, KCE and Contractor have caused this Agreement to be executed electronically by their respective duly authorized officers.

BELGIAN HEALTH CARE KNOWLEDGE CENTRE (KCE)

For acknowledgement

Maria Eyssen (Signature) Digitally signed by Maria Eyssen (Signature) Date: 2022.07.22 21:04:12 +02'00'

By ___________________________

Print Name: Marijke Eyssen, MD
Title: General Director a.i.
Date:

France Vrijens (Signature) Digitally signed by France Vrijens (Signature) Date: 2022.07.12 16:15:41 +02'00'

By ___________________________

Print Name: France Vrijens
Title: Head of KCE Trials Programme
Date:

THOMAS MORE MECHELEN - ANTWERPEN vzw

Digitaal ondertekend door Stijn Coenen (Signature) Datum: 2022.08.17 12:13:21 +02'00'

By ___________________________

Print Name: Stijn Coenen
Title: General Director
Date:

Marijke Lemal (Signature) Digitally signed by Marijke Lemal (Signature) Date: 2022.08.25 14:59:29 +02'00'

By ___________________________

Print Name: Marijke Lemal
Title: Manager Unit Research & Consultancy
Date:

For acknowledgement:

Sabine Van Eerdenbrugh Digitaal ondertekend door Sabine Van Eerdenbrugh Datum: 2022.08.26 09:33:41 +02'00'

By:___________________________

Print Name: Sabine Van Eerdenbrugh
Title: Chief Investigator
Date:

For purposes of this Agreement, a document (or signature page thereto) signed and transmitted by electronic means is to be treated as an original document. The signature of any Party on any such document, for purposes hereof and thereof, is to be considered as an original signature, and the document transmitted is to be considered to have the same binding effect as an original signature on an original document.

Université de Liège

Anne Nyssen (Signature) Signature numérique de Anne Nyssen (Signature) Date : 2022.10.17 10:10:44 +02'00'

By ______________	By ______________
Print Name:	Print Name:
Title:	Title:
Date:	Date:

For acknowledgement:

Anne-Lise Leclercq (Signature) Signature numérique de Anne-Lise Leclercq (Signature) Date : 2022.10.17 14:04:31 +02'00'

By:______________

Print Name: Anne-Lise Leclercq

Artevelde Hogeschool

Tomas Legrand (Signature) Digitaal ondertekend door Tomas Legrand (Signature) Datum: 2022.10.21 09:00:21 +02'00'

By ______________	By ______________
Print Name: Tomas Legrand	Print Name:
Title:	Title:
Date:	Date:

For acknowledgement:

Veerle Waelkens (Signature) Digitaal ondertekend door Veerle Waelkens (Signature) DN: cn=Veerle Waelkens (Signature), c=BE Datum: 2022.11.15 19:26:07 +01'00'

By:______________

Print Name: Veerle Waelkens

DocuSign Envelope ID: DA3FAC93-0EAC-4BE8-8867-AF054C945F27

Universitair Ziekenhuis Antwerpen (UZA)

DocuSigned by:
Marc Peeters
52D3E6F1892A4A9...

By ______________________	By ______________________
Print Name:prof. dr. M. Peeters	Print Name:
Title: CEO	Title:
Date:28-9-2022	Date:

LIST OF SCHEDULES:

Schedule 1: Background IP

Schedule 2: Description of the Study and Timetable

Schedule 3: Payment Schedule

Schedule 4: Reporting Schedule

Schedule 5: Study Team and Collaborators

SCHEDULE 1: BACKGROUND IP

**Owner**	**Background IP**	**Type of Background IP**	**Legal restrictions to the use of the background IP as described in this Agreement**
SLT (Speech Language Therapist)	Speech Language Therapy files	Speech Language Therapy files	KCE shall not have access to/ ownership of the SLT file:<br>(i) SLT files are not Study Data or Results; and<br>(ii) SLT files are not needed to use the Results; hence the access rights to Contractor Background IP included in Section 9.1 shall not apply with respect to SLT files

DocuSign Envelope ID: DA3FAC93-0EAC-4BE8-8867-AF054C945F27

SCHEDULE 2: Description of the Study and Timetable

1. Research Objectives

In Belgium, Speech Language Therapists (SLTs) deliver three treatment approaches for stuttering in preschool age children who stutter (PCWS): Mini-KIDS, the Lidcombe Program (LP) and Social-Cognitive Behaviour Treatment (SCBT). We believe it is necessary to assess the treatment outcome of those three treatments as it is vital to try to achieve the best possible outcome for PCWS in the daily clinical practice before the age of 6 years, so within the preschool age years (also called 'the window of opportunity').

We estimate, based on findings from the literature, that the three treatment programs will achieve similar outcomes at 18 months post-treatment initiation. That is, (near) zero levels of stuttering and linked to this result, a high score on Quality-Of-Life scores (QOL) at 18 months, 2 and 5 years post-treatment initiation. These three stuttering treatment approaches for preschool age children are delivered daily to Belgian preschool age children who stutter. Their effect compared to each other, however, is not known. We believe, as it is our duty to aim for as much recovery of early childhood stuttering as possible before stuttering becomes persistent, that the current trial is necessary to support current practice in Belgium. Also, it is essential to provide evidence for the treatments to help SLTs choose the most appropriate treatment approach for a family.

As a secondary objective, we expect a significant difference in treatment time necessary to reach the treatment goals. We believe that the direct treatment programs (the LP and Mini-KIDS) need less treatment time (measured in hours) than SCBT with a difference of about 1/3 to nearly 1/2 of the treatment time. This is an estimation based on available publications (Arnott et al., 2014; Bridgman et al., 2016; De Sonneville-Koedoot et al., 2015; Donaghy et al., 2020). We believe that this is an important objective with implications on both the families and the Belgian reimbursement system. To shorten the treatment time implies a reduction of parents' burden of providing time and travelling to the stuttering practice and the costs for parents and the Belgian federal government.

In conclusion, the primary objective for this trial is to compare the % Syllables Stuttered (%SS, a measurement for the frequency of stuttering) between the speech samples of PCWS treated with Mini-KIDS, SCBT and the LP at 18 months post-treatment initiation.

The secondary objectives for this trial are

- To compare the %SS, measured on video recordings by blinded video analysts, between PWCS treated with Mini-KIDS, SCBT and the LP at 3, 6, 9 and 12 months post-treatment initiation
- To compare the Severity Ratings (SR, a subjective measure for frequency and severity, measured on video recordings by blinded video analysts) between PCWS treated with Mini-KIDS, SCBT and the LP at 3, 6, 9, 12 and 18 months post-treatment initiation
- To compare the Quality of Life (QOL, measured with the EQ-5D-Y proxy 1) and the impact of stuttering on PCWS and parents (measured with the ISPP-questionnaire) between PCWS treated with Mini-KIDS, SCBT and the LP at 3, 9 and 18 months post-treatment initiation

- To compare parent report about stuttering severity and satisfaction with everyday communication between PCWS treated with Mini-KIDS, SCBT and the LP at 3, 6, 9, 12 and 18 months, 2 and 5 years post-treatment initiation
- To compare the communication attitude (measured with the KiddyCAT) between PCWS treated with Mini-KIDS, SCBT and the LP at 18 months post-treatment initiation
- To compare the %SS and SR (measured on video recordings by blinded video analysts) and QOL (EQ-5D-Y proxy 1) between PCWS treated with Mini-KIDS, SCBT and the LP at 2 and 5 years post-treatment initiation
- To compare the treatment time (in hours), number of weeks and number of treatment sessions until the start the maintenance phase of each treatment
- To look at the proportion of children that are successful (defined as <1 %SS and SR ≤ 1) at 18 months and at 5 years post-treatment initiation.
- To evaluate %SS and SR at 18 months post-treatment initiation for the videos at home.
- To evaluate %SS and SR at 18 months post-treatment initiation for the videos recorded during the treatment sessions.

Flowchart

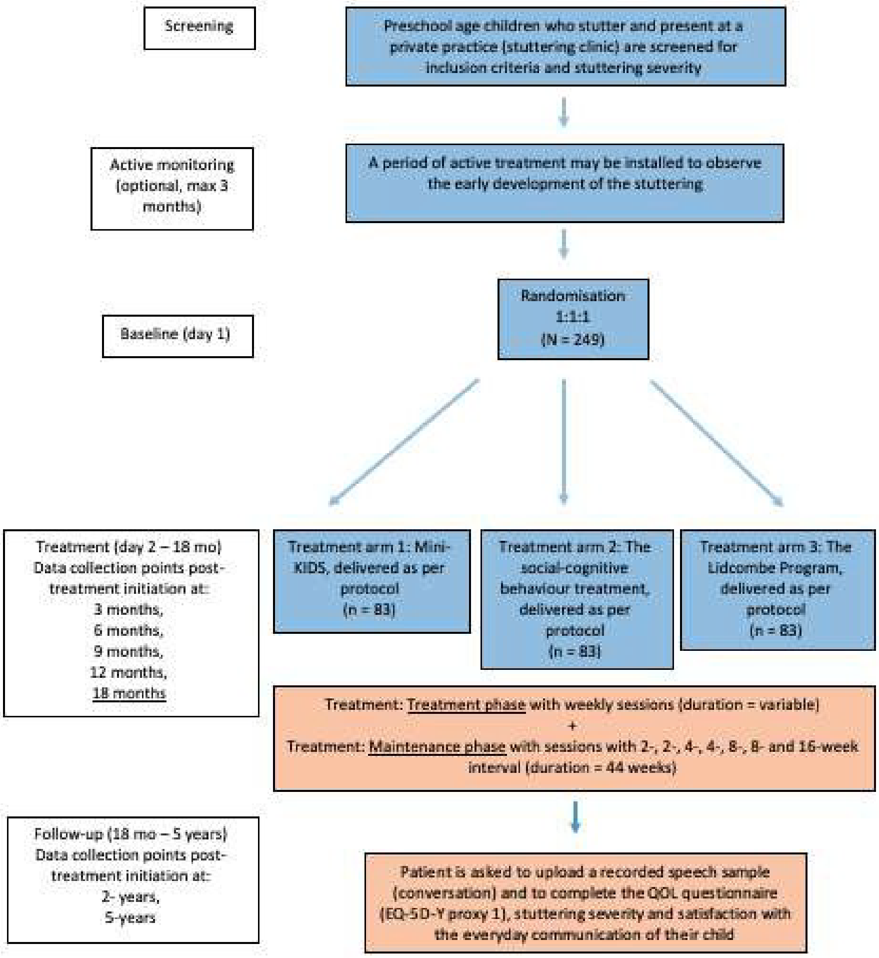

This study ("TreatPaCS") is a three arm, 1:1:1 randomised, open-label pragmatic comparative trial comparing Mini-KIDS, SCBT and the LP.
To show non-inferiority of Mini-KIDS compared to LP and SCBT compare to LP a parallel group design will be used. Stratified randomisation will be used according to site (+/- 30 sites) and gender.
TreatPaCS is a multicentre trial. Eligibility criteria for the sites and participating SLTs are:

- The site is a private practice specialised in stuttering (does not need to be exclusively in stuttering)
- The participating SLT is experienced in treating PCWS with at least 2 years of experience
- The participating SLT is prepared to deliver the three treatment approaches
- The participating SLT attends (again) the three workshops (Mini-KIDS, SCBT and LP) and the International Conference on Harmonisation of technical requirements for registration of pharmaceuticals for human use- Good Clinical Practice (ICH-GCP) workshop
- The participating SLT starts stuttering treatment with on average 1.5 PCWS per 3 months per site or more

2. Timetable

**Task**	**Target date for completion**
Sign KCE-BCC agreement	01/02/2022
Approval CA/EC	15/03/2022
First Patient In (FPI)	01/04/2022
82 patients recruited	01/10/2022
164 patients recruited	01/04/2023
Last Patient In (LPI)	01/10/2023
Last Subject Last Visit (LSLV)	01/11/2025
Data Base Lock (DBL)	01/02/2026
Clinical Study Report (CSR)	01/05/2026
Publication submitted	01/06/2026
Long stop date	01/02/2023

SCHEDULE 3: Budget and Payment Schedule

A. BUDGET

The maximum Fee to be paid by KCE for this Study and all related activities under this Agreement (including any overhead) is 1 772 645,71 €
VAT is not applicable to the amounts granted by the KCE to the Sponsor for the conduct of the clinical study (E.T.131.445/2). This maximum budget is based on an estimate of the workload involved for each task as part of the Study and all related activities (as detailed in the budget table below).

	Budget incl overhead
Project Design and Set up	88 518,67 €
Regulatory and Ethics Review	11 108,62 €
Monitoring	277 326,43 €
Quality Assurance	11 700,00 €
TMF Handling & Administration	98 178,17 €
Safety	
Data Management	100 018,15 €
Statistics, report and publication	60 840,00 €
Patient and Public Involvement	6 896,76 €
Project Management	475 979,34 €
Site Costs*	453 162,62 €
External vendors/contractors/central review*	188 916,94 €
Overall Cost total	1 772 645,71 €

*includes the non deductible VAT for the sponsor where applicable

The actual Fee due by KCE under this Agreement depends on the actual number of sites and patients participating to the Study and the actual number of FTEs involved by (or on behalf of) the Contractor. The actual Fee due by KCE may therefore be lower than the aforementioned budgeted amount, but cannot be higher. Contractor guarantees to make the necessary human resources available (as foreseen in the budget table) for the performance of each task as part of the Study. Any major deviation in such number of available resources shall require the prior written approval of KCE.

DocuSign Envelope ID: DA3FAC93-0EAC-4BE8-8867-AF054C945F27

B. PAYMENT SCHEDULE

There are 3 payment schedules:
B1. Payment Sponsor Costs: milestone-based payments of the sponsor costs
B2. Payment Site Costs: to cover the site fees as referenced in the site agreement with the participating sites.
B3. Payment Other Costs: to cover other fees as referenced in other agreements (Collaborators, External Vendors or any subcontractor).

	including any overhead
**TOTAL COST STUDY**	1 772 645,71 €
**TOTAL SPONSOR COST***	583 528,71 €
**TOTAL SITE COST***	453 162,62 €
**TOTAL UZA COST***	535 337,44 €
**TOTAL OTHER COSTS***	200 616,94 €

*includes the non deductible VAT for the Sponsor where applicable

By rounding figures, it is possible that there are very small differences when calculating the totals of the different subschedule as taken from the preparatory excel budget tool. The total maximum amounts are taken from the table above.

Contractor will be in charge of the payment of any invoices (including VAT) from any of its Collaborators, External Vendors or any subcontractor.

B1. Payment Sponsor Costs

The total amount to cover the costs for the sponsor is 583 528,71 € and will be paid according to the milestone schedule below.

**MILESTONE DATE OR EVENT**	**%**	**AMOUNT**	**ADDITIONAL CONDITINOS OR REQUIREMENTS**
Advance Payment		18 750,00 €	Advance payment letter signed
Contract signed	15%	68 779,31 €	Contract signed by all parties
All plans provided	5%	29 176,44 €	Risk Assessment Plan, Monitoring Plan and Data Management Plan received by KCE
First Patient In	5%	29 176,44 €	First Patient Randomised and entered in EDGE
33% patients randomised (82)	5%	29 176,44 €	33% of the patients randomised, EDGE updated and data entered into eCRF
66% patients randomised (164)	5%	29 176,44 €	66% of the patients randomised, EDGE updated and data entered into eCRF
all patients randomised (249)	15%	87 529,31 €	All patients randomised, EDGE updated and data entered into

			eCRF
50% patients completed (125) 12 months	5%	29 176,44 €	50% of the patients completed 12 months visit in eCRF
100% patients completed (249) 12 months	10%	58 352,87 €	All patients completed 12 months visit in eCRF
50% patients completed (125) 18 months	5%	29 176,44 €	50% of the patients completed 18 months visit in eCRF
100% patients completed (249) 18 months	10%	58 352,87 €	All patients completed 18 months visit in eCRF
100% patients completed (249) 2 years	5%	29 176,44 €	All patients completed 24 months visit in eCRF
database lock	5%	29 176,44 €	Database has been declared as clean and locked by data manager
clinical study report	5%	29 176,44 €	Clinical Study Report delivered to KCE
manuscript submitted	5%	29 176,44 €	Manuscript submitted to journal and KCE confirms in writing that the Study has been completed in accordance with the Agreement

B2. Payments Site Costs

The Contractor is responsible for the payment of any fees directly to the participating sites under the Study. The total costs for payments to participating sites should not exceed 453 162,62 € € (OH incl, VAT incl). Overhead is not applicable on the site fees. Deductible VAT is an ineligible cost for KCE funding. VAT that is recoverable is not a genuine and definitive cost and will not be payed for by KCE.
The overall maximum amount is based on the assumption that 249 patients will enter and complete the trial in 30 sites. The breakdown of the costs is detailed below.

<u>B2.1. Costs related to the visits</u> (administration, data entry, extra sessions)
The table below is based on the estimated time required by the sites to complete the non-standard of care tasks related to the study. In line with good practice recommendations for the Mini-KIDS and the Lidcombe Program, an additional 30 minutes of care for the first 6 sessions of these two programs have been included in the budget as the current reimbursement rules of INAMI-RIZIV do not allow for reimbursement of 60-minute sessions in children under 10 years of age.
The total maximum amount for payment to the participating SLTs (start-up, administration, extra sessions) is 277 625,96 € (Overhead not applicable, VAT excl) or 335 927,41 € (OH and applicable VAT incl) based on 249 children participating in 30 practices.

DocuSign Envelope ID: DA3FAC93-0EAC-4BE8-8867-AF054C945F27

Fee or visit period	AMOUNT (overhead not applicable)	AMOUNT (incl. VAT charged if applicable)*	#	Total maximum amount for the study VAT incl	ADDITIONAL CONDITIONS OR REQUIREMENTS
Screening and Randomisation Visit	186,98 €	226,24 €	249	56 334,60 €	Visit data entered in the eCRF
Visit 1	124,65 €	150,83 €	249	37 556,40 €	Visit data entered in the eCRF
Visit 2	124,65 €	150,83 €	249	37 556,40 €	Visit data entered in the eCRF
Visit 3	124,65 €	150,83 €	249	37 556,40 €	Visit data entered in the eCRF
Visit 4	124,65 €	150,83 €	249	37 556,40 €	Visit data entered in the eCRF
Visit 5	124,65 €	150,83 €	249	37 556,40 €	Visit data entered in the eCRF
Visit 6	124,65 €	150,83 €	249	37 556,40 €	Visit data entered in the eCRF
Start-Up Fee	200,00 €	242,00 €	30	7 260,00 €	Contract signed, site initiated and at least 1 patient included
Archiving	260,00 €	314,60 €	30	9 438,00 €	Site closure has been performed and site included at least 1 patient
Fee per 30 minutes session for LP or Mini-Kids	31,16 €	37,71 €	996	37 556,40 €	Treatment session entered and verified in eCRF

** Sites may charge the amount inclusive VAT to the sponsor however deductible VAT should not be invoiced to KCE*

Total maximum fee for a patient completing all visits is 934,89 € (OH incl, VAT excl) or 1 131,22 € (OH incl, VAT incl)
Total maximum fee for a patient completing the 1st 6 sessions of the Lidcombe or the Mini-KIDS programme is 186,98 € (VAT excl) or 226,24 € (VAT incl). The total maximum number of children needing these sessions is 166. The total maximum amount to cover the additional costs for these sessions is 37 556,40 €.

B2.2. Costs related to the training

To establish a common and consistent way of working for all 3 stuttering approaches, training sessions will be organized by the study team. It is expected that each participating SLT will take part in the training programme which consists of:

GCP-training: 3h
SCBT training: 3 x 6h

Mini-KIDS: 3 x 6h
Lidcomb Program: 3 x 6h

The total duration of the training takes 57h and will be compensated at a rate of 56,66€ per hour with a maximum of 3 229,62 € (VAT not included) or 3 907,84 € (VAT included where applicable) for an SLT completing the full training programme. The compensation will be paid if at least 1 child is recruited by the SLT within 1 year after initiation of the study at the LST's site.
The total maximum budget for the compensation for training is 96 888,60 € (VAT not included) or 117 235,21 € (VAT included where applicable) taking into account that maximum 30 sites will participate.

B2.3. Invoicing for site related costs
Contractor will be in charge of the payment of any invoices (including VAT) from any of its Collaborators. The VAT amount will only be charged by Contractor to KCE if charged by any of said Collaborators and not being deductible by the Contractor.
Subject to the terms of this Agreement, the Parties agree that payments to cover the costs of participating sites will be invoiced by contractor to KCE bi-annually (in April and October).

To facilitate the oversight, the Contractor will provide an aggregated overview detailing the amounts per site and for each site a summary containing details on the amounts (visits completed, start-up, archiving. KCE will confirm the amount that can be invoiced by Contractor to KCE by sending a Request for Invoice.

KCE will receive a copy of the invoices paid by Contractor to the sites as part of the reporting responsibilities; any discrepancy in the fees paid by sponsor to the sites and the amount paid by KCE in consideration of these site activities will be deducted or added by sponsor to the next invoice to KCE for site activities. In no case can the overall amount invoiced by Contractor to KCE for site activities be higher than the overall amount budgeted for these activities nor can any unused part of this budget be used for other activities performed by the sponsor.

KCE expects Contractor to use the EDGE web-based software tool in order to allow KCE to monitor the recruitment in an anonymized manner "in real time". KCE is paying for the software license to facilitate the use of EDGE.
The payment of the invoice regarding the site costs will depend on the number of patients entered in EDGE and confirmed by the study team.

B3. Payments for activity based costs (non-sites)

The budget has been calculated to include activity based costs that are depending upon delivery of the activity and that can be provided by the Contractor himself or by Collaborators, External Vendors or subcontractors. The Contractor is responsible for the payment of any fees directly to the Collaborators, External Vendors, subcontractors involved in providing services or goods for this Study. The total amount for payments of the activity based costs should not exceed 200 616,94 € (OH incl, VAT incl).

DocuSign Envelope ID: DA3FAC93-0EAC-4BE8-8867-AF054C945F27

The general overview of the breakdown of the costs can be found below:

Service	Total maximum amount for the study (incl. overhead and incl. VAT charged by provider if applicable)*
Quality Assurance	11 700,00 €
Study specific equipment	11 497,18 €
Delivery of Training	42 337,87 €
Review of Video samples	98 727,89 €
Vouchers for patients	36 354,00 €
Total maximum amount	200 616,94 €

** Collaborators, External Vendors, subcontractors will charge the amount inclusive VAT to the sponsor however deductible VAT should not be invoiced to KCE*

The deliverables can be split in the following categories:

B3.1. Quality Assurance

The total maximum costs for Quality Assurance activities is 11 700 € (including overhead and applicable VAT) and will be provided after receipt of an audit certificate. The following amounts can be allocated:

- Sponsor audit including TMF: up to 5 850,00 €
- Site audit: up to 5 850,00 €
- TMF audit: up to 2 925,00 €
- Audit part of a systems audit (eCRF, safety, …): up to 2 925 €

B3.2. Study specific equipment

The total cots for study-specific equipment should not exceed 9 501,80 € (VAT not included) or 11.497,18 € (VAT included where applicable) to cover the following costs:

- Fluency raters
- Hardware for video recorded speech samples
- Hardware back-up video recording

	AMOUNT (incl. overhead)*	AMOUNT (incl. overhead and incl. VAT charged by site)*	#	Total maximum amount for the study VAT incl	ADDITIONAL CONDITIONS OR REQUIREMENTS
Fluency raters	6,00 €	7,26 €	5	39,93 €	Can be invoiced together with the 1st milestone
Hardware for collecting videorecordings	8 800,00 €	10 648,00 €	1	10 648,00 €	On receipt of invoice
Hardware back-up video recording	167,20 €	202,31	4	809,24 €	On receipt of invoice

B3.3. Delivery of Training

- GCP training (delivered by UZA)
- SCBT NL training (delivered by external partner)

DocuSign Envelope ID: DA3FAC93-0EAC-4BE8-8867-AF054C945F27

- MINI-KIDS and LP training (delivered by study team)
- SCBT FR training (delivered by study team)

	**AMOUNT (incl. overhead)***	**AMOUNT (incl. overhead and incl. VAT charged by site)***	**#**	**Total maximum amount for the study VAT incl**	**ADDITIONAL CONDITIONS OR REQUIREMENTS**
GCP-training	584,07 €	706,72 €	2	1 413,44 €	On receipt of invoice (+ dates, location, program and number of attendees)
SCBT NL training	2 430,00 €	2 940,30 €	2	5 880,60 €	On receipt of invoice (+ dates, location, program and number of attendees)
Mini-KIDS training	3 504,38 €	NA	4	14 017,54 €	On confirmation that training has been provided with dates, location, program and number of attendees
Lidcombe Program training	3 504,38 €	NA	4	14 017,54 €	On confirmation that training has been provided with dates, location, program and number of attendees
SCBT FR training	3 504,38 €	NA	2	7 008,76 €	On confirmation that training has been provided with dates, location, program and number of attendees

<u>B3.4. Review of Video samples</u>

- Rating of speech samples
- Assessment of treatment fidelity

	**AMOUNT (incl. overhead) ***	**AMOUNT (incl. overhead and incl. VAT charged by site)***	**#**	**Total maximum amount for the study VAT incl**	**ADDITIONAL CONDITIONS OR REQUIREMENT S**
Rating videosamples	22,10 €	NA	3834	84 731,40 €	On receipt of invoice detailing the number of videos rated
Assessment of treatment fidelity *(in 20%*	97,34 €	NA	131	13 993,20 €	On receipt of invoice detailing the number of

of participants 30min each video + first 60 minutes in 2 arms, 4x during treatment + 5% ad hoc if inconsistenties appear at certain sites)					hours spent for rating and the number of videos rated

B3.5. Vouchers for patients

	**AMOUNT (incl. overhead)***	**AMOUNT (incl. overhead and incl. VAT)***	**#**	**Total maximum amount for the study VAT incl**	**ADDITIONAL CONDITIONS OR REQUIREMENTS**
Patient vouchers	€ 20.00	NA	249	36 354,00 €	On receipt of invoice

B3.6. Sponsor activities conducted by UZA

Part of the sponsor tasks are conducted by the CTC of UZA. As a Partner of the Consortium, they provide support for monitoring, data management and statistics as well as general support for the follow-up of the study. The milestones will follow the same schedule as in B1. Payment Sponsor Costs.
The total maximum amount for delegated sponsor costs for UZA will be 535 337,44 € (OH included, VAT included).
If other activities are provided by UZA which are activity based (e.g. GCP training), they will be invoiced separately from the milestones.

**MILESTONE DATE OR EVENT**	**%**	**AMOUNT***	**ADDITIONAL CONDITINOS OR REQUIREMENTS**
Advance Payment		6 250,00 €	Advance payment letter signed
Contract signed	15%	74 050,62 € (Advance payment deducted)	Contract signed by all parties
All plans provided	5%	26 766,87 €	Risk Assessment Plan, Monitoring Plan and Data Management Plan received by KCE
First Patient In	5%	26 766,87 €	First Patient Randomised and entered in EDGE
33% patients randomised (82)	5%	26 766,87 €	33% of the patients randomised, EDGE updated and data entered into eCRF
66% patients randomised (164)	5%	26 766,87 €	66% of the patients randomised, EDGE updated and data entered into eCRF

all patients randomised (249)	15%	80 300,62 €	All patients randomised, EDGE updated and data entered into eCRF
50% patients completed (125) 12 months	5%	26 766,87 €	50% of the patients completed 12 months visit in eCRF
100% patients completed (249) 12 months	10%	53 533,74 €	All patients completed 12 months visit in eCRF
50% patients completed (125) 18 months	5%	26 766,87 €	50% of the patients completed 18 months visit in eCRF
100% patients completed (249) 18 months	10%	53 533,74 €	All patients completed 18 months visit in eCRF
100% patients completed (249) 2 years	5%	26 766,87 €	All patients completed 24 months visit in eCRF
database lock	5%	26 766,87 €	Database has been declared as clean and locked by data manager
clinical study report	5%	26 766,87 €	Clinical Study Report delivered to KCE
manuscript submitted	5%	26 766,87 €	Manuscript submitted to journal and KCE confirms in writing that the Study has been completed in accordance with the Agreement

<u>B3.7. Invoicing for activity based costs</u>

To facilitate the oversight, the Contractor will provide an overview detailing the milestones or services that will be charged by the Collaborators, External Vendors, subcontractors. KCE will confirm the amount that can be invoiced by Contractor to KCE by sending a Request for Invoice.

KCE will receive a copy of the invoices paid by sponsor to the Collaborators, External Vendors, subcontractors as part of the reporting responsibilities; any discrepancy in the fees paid by sponsor to the subcontractor and the amount paid by KCE in consideration of these activities will be deducted or added by sponsor to the next invoice to KCE for subcontractor activities. In no case can the overall amount invoiced by Contractor to KCE for Collaborators, External Vendors, subcontractors activities be higher than the overall amount budgeted for these activities nor can any unused part of this budget be used for other activities performed by the sponsor.

SCHEDULE 4: Reporting Schedule

Contractor shall report to KCE on the study progress through monthly web meetings. Minutes of these update meetings will be written by the Contractor and provided to KCE. Minutes should at least address the topics below and in addition to these minutes, the following documents (and any updates) should be provided by the Contractor to KCE for information purposes:

Before EC/RA approval:
- Protocol and any amendments: submitted to KCE for review as stated in section 2.2.4
- Informed Consent Form and any amendments: submitted to KCE for review as stated in section 2.2.5 (b)

Before FPI:
- Study Plans (as far as applicable to the trial setting): Risk Assessment Plan, Data Management Plan, Monitoring Plan, Safety Management Plan, IMP Management Plan, …
- Draft annotated eCRF
- Approval of the Competent Authority and/or Ethics Committee of the initial submission as well as approvals of any amendments in the course of the study

During the Study:
- Study progress reports, detailing the number of sites who have signed the contract as well as the dates of initiation of the sites, financial information, status of monitoring, status of data entry, data query generation and query resolution
- Any publication on the Study whether in a journal, lay press, website, scientific meeting as in section 16.4
- Any updates to the documents mentioned above

Before Data Base Lock:
- Statistical Analysis Plan
- Study Results Dissemination Plan

After Study Closure:
- Clinical Study Report
- Any publication on the Study Results whether in a journal, lay press, scientific meeting as in section 8.4.1
- Lay language summary in all relevant languages (at least NL, FR, ENG)
- Confirmation of uploading of the study results on the public website (eg ClinicalTrials.gov, EudraCT, …) that the study was published on

DocuSign Envelope ID: DA3FAC93-0EAC-4BE8-8867-AF054C945F27

SCHEDULE 5: Study Team, Collaborators (including Partners of the Consortium), External vendors and subcontractors

1. Key members of the Study Team

Chief Investigator
Sabine Van Eerdenbrugh
Thomas More Mechelen-Antwerpen vzw, Opleiding Logopedie en Audiologie
Sint-Andriesstraat 2, B-2000 Antwerpen, Belgium
+32 485 152 033
Sabine.vaneerdenbrugh@thomasmore.be

Co-Chief Investigators (including training and video-analysis)
Anne-Lise Leclercq
Université de Liège, Département de Logopédie
30 rue de l'Aunaie, B.38, B-4000 Liège
+32 479 355 044
Al.leclercq@uliege.be

Veerle Waelkens
Artevelde Hogeschool, Opleiding Logopedie en audiologie,
Campus Kantienberg, Voetweg 66, B-9000 Gent
+32 475 856 704
Veerle.waelkens@arteveldehs.be

Field Monitor
Mathias Allegaert
Universitair Ziekenhuis Antwerpen (UZ-ANTWERPEN)
Drie Eikenstraat 655, B-2650 Edegem, Belgium
+32 3 821 59 47
Mathias.allaegaert@uza.be

Randomisation
Sofie Grenier and Debbie Smet
Universitair Ziekenhuis Antwerpen (UZ-ANTWERPEN)
Drie Eikenstraat 655, B-2650 Edegem, Belgium
Ctc@uza.be
+32 3 821 23 67

Data Management
Kim Claes
Universitair Ziekenhuis Antwerpen (UZA)
Drie Eikenstraat 655, B-2650 Edegem, Belgium
+32 3 821 59 42

Kim.claes@uza.be

Statistician and Safety Data Reporting:

Ella Roelant
Universitair Ziekenhuis Antwerpen (UZA)
Drie Eikenstraat 655, B-2650 Edegem, Belgium
+32 3 821 42 25
Ella.roelant@uza.be

Coordination statistical analyses, data management & site monitoring

Iris Verhaegen
Universitair Ziekenhuis Antwerpen (UZA)
Drie Eikenstraat 655, B-2650 Edegem, Belgium
+32 3 821 35 44
Iris.verhaegen@uza.be

Elke Smits
Universitair Ziekenhuis Antwerpen (UZA)
Drie Eikenstraat 655, B-2650 Edegem, Belgium
+32 3 821 40 49
Elke.smits@uza.be

2. Collaborators

CTC UZA
Universitair Ziekenhuis Antwerpen (UZA)
Drie Eikenstraat 655, B-2650 Edegem, Belgium

Université de Liège
Département de Logopédie
30 rue de l’Aunaie, B.38, B-4000 Liège

Artevelde Hogeschool
Opleiding Logopedie en audiologie,
Campus Kantienberg, Voetweg 66, B-9000 Gent

List of SLTs that initially agreed to participate in the study

Name	First Name	Address	
Aerts	Maxime	Hemeldonk 3	2275 Gierle
Bauwens	Jaïke	Ter Platen 84	9000 Gent
Berndt	Candice	Av Montjoie 13	1180 Uccle

DocuSign Envelope ID: DA3FAC93-0EAC-4BE8-8867-AF054C945F27

Bilteryst	Céline	Rue de Liberchies 31	6238 Luttre
Bodeux	Edith	Haut Nivezé, 27	4845 Sart-lez-Spa
Bongaerts	Anke	Groenendallaan 107	2170 Merksem
Coekaerts	Rachel	Hullekenstraat 88 bus 3	9200 Dendermonde
Copejans	Isabelle	Anjelierenstraat 27	9120 Beveren
Cornez	Charlotte	Rue Pierreuse, 188	4000 Liège
De Briey	Blanche	Av Marie de Hongrie 64	1083 Ganshoren
De Clercq	Lisa	Klaroenstraat 9	8540 Deerlijk
Desmaris	Anaïs	Rue Jean Paquot 34	1050 Ixelles
Dontaine	Florence	rue Baron Lambert 38	1140 Etterbeek
Fleerackers	Julie	Beekstraat 59	2500 Koningshooikt
Gorissen	Veerle	Dr. Houbenlaan 68/70	3630 Maasmechelen
Hansoulle	Laurie	Rue des glacis 15	4000 Liège
Lorrain	Elise	Rue du Ham 126	1180 Uccle
Masselis	Kirsten	Kongostraat 18	8520 Kuurne
Moyse	Astrid	Rue du rivage 7	4920 Aywaille
Onclin	Fanny	Close de Hesbaye 28	4300 Waremme
Otte	Clara	Avenue de Broqueville 158	1200 Woluwe-Saint-Lambert
Peeters	Lieve	Hemeldonk 3	2275 Gierle
Schaers	Brechje	Kroonstraat 71	9940 Ertvelde
Van den Eynde	Evelien	Boekevijverstraat 11a	9506 Geraardsbergen
Van Vooren	Jenna	Vroentestraat 65	3320 Hoegaarden
Vanhoutte	Sarah	Stationsstraat 28	9840 De Pinte
Vicq	Alice	Chaussée de Vleurgat 266	1050 Bruxelles

3. Subcontractors and External vendors

UZA Dienst ICT – Wetenschappelijke toepassingen - Elyne Scheurwegs
Initiële opzet own-cloud server + VM provisional (small) + technische dienstverlening
Storage, back up en off-site beschikbaarheid 6TB gedurende 7 jaar

Sodexo
Vouchers for parents

Thomas More Dienstverlening - Mark Meersman
Training of Social Cognitive Behavioural Treatment
