# Supplementary material for: Treatment for preschool age children who stutter: Protocol of a randomised, non-inferiority parallel group pragmatic trial with Mini-KIDS, social cognitive behaviour treatment and the Lidcombe Program—TreatPaCS
Source: PLoS One. 2024 Jul 11;19(7):e0304212. doi: 10.1371/journal.pone.0304212 (PMC11239023; doi:10.1371/journal.pone.0304212)
Supplement: S4 File — a. KCE Review process. Review process applied to the protocol for funding. b. Agreement between KCE and Thomas More University of Applied Sciences. (ZIP) [file pone.0304212.s005.zip › S5a KCE Review process.pdf]

KCE TRIALS PROGRAMME
STANDARD OPERATING PROCEDURE

**TITLE: INVESTIGATOR-LED WORKSTREAM: CALL LAUNCH TO TRIALS BOARD RECOMMENDATION TO FUND**		
ID: KCET-SOP-CA-001	Version Number: 2.1	Effective Date: 24/03/2022

**Revision History**		
**Version No.**	**Effective Date**	**Description**
1.0	01/06/2018	Initial release
2.0	14/10/2019	First revision: addition of “Abbreviations”, “Responsibilities” and diagrams. Modified for the use of SmartSimple. Integration of KCET_WI_CA_001.
2.1	24/03/2022	Modify slightly which call documents need to be signed<br>Include references to recent laws clinical trials<br>Small changes to responsibilities<br>Small change to panel composition

Author: **Date:**

Elisabeth Marynen (Authentication) Digitally signed by Elisabeth Marynen (Authentication) Date: 2022.03.28 12:22:10 +02'00'

Lisa Marynen, Researcher**,** Signature above

Approved By: **Date:**

France Vrijens (Signature) Digitally signed by France Vrijens (Signature) Date: 2022.03.25 10:18:52 +01'00'

France Vrijens, Head of KCE trials, Signature above

Contents

1 PURPOSE 3
2 SCOPE 4
3 DEFINITIONS/ABBREVIATIONS 4
4 RESPONSIBILITIES 4
5 PROCEDURES 5
5.1 CALL FOR PROPOSALS AND THEIR PRIORITISATION 5
5.1.1 Call for research outlines 5
5.1.2 Receipt of research outlines and verification that they are valid and within scope 5
5.1.3 Prioritisation 6
5.1.3.1 Scoring for potential value for money by KCE health economist 6
5.1.3.2 Scoring for clinical relevance and need for evidence by Trials panel 7
5.1.3.3 Prioritisation of research outlines by the Prioritisation Group 7
5.1.3.4 Feedback to applicants 7
5.1.4 Schematic diagram 8
5.2 REVIEW AND SHORTLISTING OF RESEARCH OUTLINES BY THE TRIALS BOARD 8
5.2.1 Composition of the review 8
5.2.2 Trials Board meeting 8
5.2.3 Communication of TB decisions 9
5.3 REVIEW AND RECOMMENDATION FOR FUNDING OF FULL RESEARCH PROPOSALS BY THE TRIALS BOARD 10
5.3.1 Trials Board meeting 10
5.3.2 Proposals invited to resubmit an updated FRP 11
5.3.3 Full research proposals which are recommended for funding with changes 11
5.4 PRIORITISATION OF FULL RESEARCH PROPOSALS BY THE PRIORITISATION GROUP 11
5.5 SCHEMATIC DIAGRAM (FROM RO SHORTLISTING TO FRP PRIORITISATION) 11
5.6 INTERNATIONAL TRIALS WITH SPONSOR ABROAD. 12
5.7 TIMELINES/END OF CALL 12
5.8 POSSIBILITY TO POSTPONE, SUSPEND AND/OR WITHDRAW ANY PENDING RESEARCH CALL 12
5.9 PILOT STUDIES 12
6 REFERENCES 13
7 APPENDICES 13

1 PURPOSE

This SOP describes the KCE Trials investigator-led workstream including call for proposals, clinical question prioritisation by panels, review and shortlisting of outlines and prioritisation of full research proposals for funding decision. This SOP describes the processes from the publication of the call until recommendation for funding by the Trials Board (TB) to the Prioritisation Group (PG).

Another workstream to fund clinical trials is the commissioned workstream (top-down) for which the first part of the process is different and described in a separate SOP. From the Research Outline (RO) evaluation of the Trials Board, both procedures are identical and described in this SOP (see Fig 1).

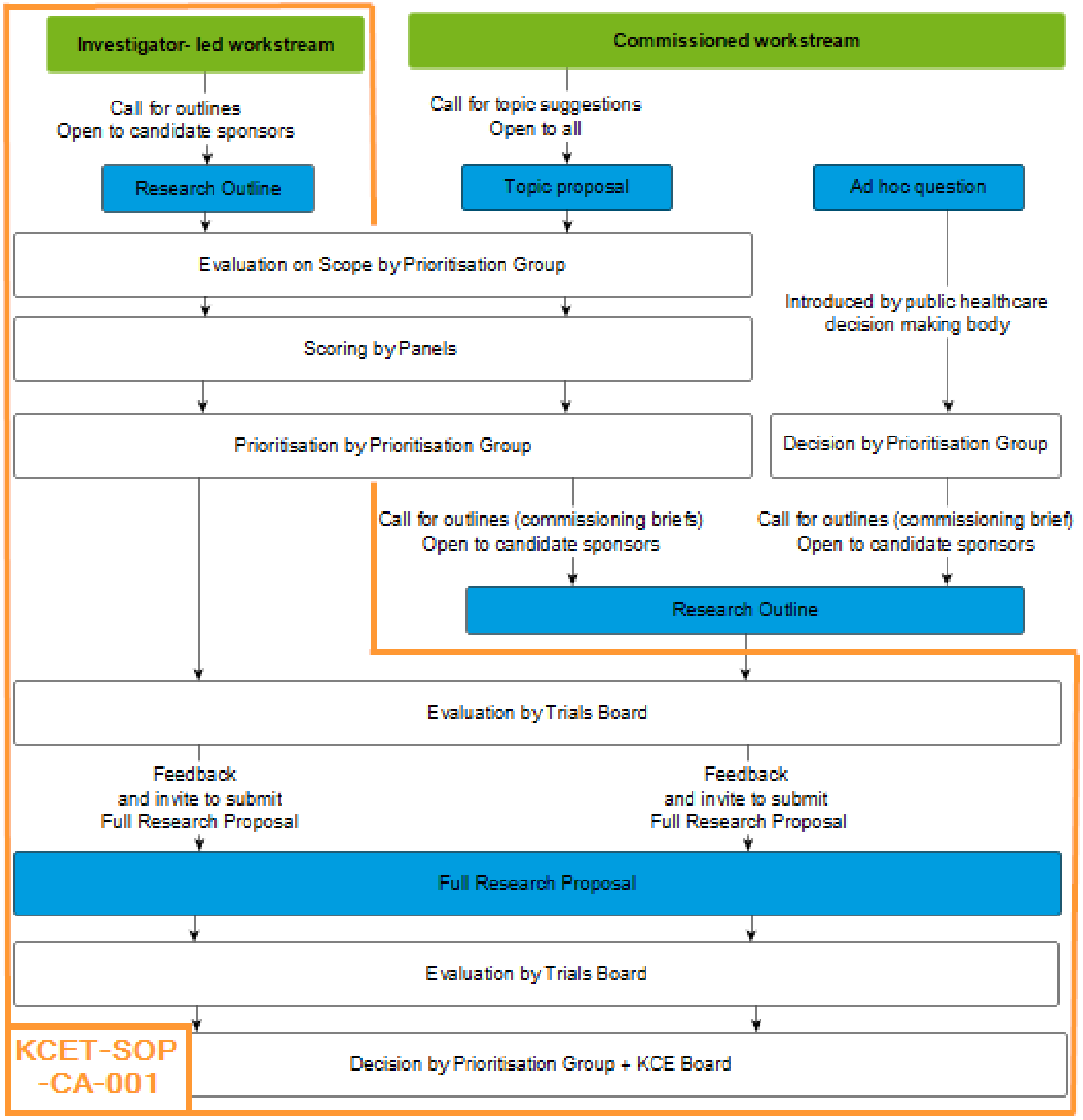

Fig 1 Steps covered by KCET-SOP-CA-001

2 SCOPE

This SOP applies to members of the KCE Trials team and any collaborators who they sub-contract tasks to for all activities related to the selection and prioritisation of the bottom-up 'investigator-led workstream' for clinical trial funding. Investigator-led call set up in collaboration with other funding agencies are out of scope.

3 DEFINITIONS/ABBREVIATIONS

CI Chief Investigator

FRP: Full Research Proposal

HE: Health Economic

NIHR: National Institute for Health Research

NIHDI: National Institute for health and Disability Insurance (RIZIV/INAMI)

PG: Prioritisation Group

RO: Research Outline

ROI: Return on Investment

TB: Trials Board

4 RESPONSIBILITIES

**Roles *(add each function impacted)***	**Tasks**
Head of KCE Trials	Sign-off call text and research agreement template when posted on the website<br>Participate in call coordination and planning<br>Sign-off of decision letters with chair TB<br>Sign-off advance letters and contracts for acknowledgement
Call coordinator	Ensure call coordination and planning<br>Overall management of SmartSimple (online grant management system)<br>Participate in reviewing Research Outlines (ROs) and Full Research Proposals (FRPs)
Programme assistant	Support in planning meetings and inviting members<br>Set-up review meetings in SmartSimple and help with any support needed for the system
KCE Trials team members	Participate in call coordination and planning<br>Review ROs and FRPs<br>Draft decision letters

KCE (Trials) health economist	Assess the Full Research Proposals (potential value for money and ROI, specific questionnaires for HTA).

5 PROCEDURES

5.1 Call for proposals and their prioritisation

5.1.1 Call for research outlines

A call for studies within the investigator-led workstream starts with a call for research outlines (RO). This is done via an open call which is at minimum advertised on the KCE website and an email alert sent to the list of subscribers to the KCE Trials newsletter. Additional ways to spread information about the call as broadly as possible may include presentations about the call at hospitals or other meetings and dissemination of information via Clinical Trial Units (CTUs).

The information related to the call is published on the KCE website. It will include:

- Call text with scope, eligibility and selection criteria and procedures for the call
- All necessary templates (e.g. budget tool template), guidelines and instructions on the process and how to submit
- Closing date for applications (applicants will be given a minimum of 8 weeks to submit a proposal)

The call text and the research agreement template are signed off by the head of KCE Trials and filed on the KCE Trials share drive or stored on paper.

All submissions must be done using the KCE Trials online submission platform (SmartSimple). Only the Chief Investigator (CI) can start and submit an application but other team members can help completing the online application form.

When submitting a RO, investigators should have the written support of a non-commercial legal entity that can function as sponsor for non-commercial multicentre trials and has the ability to comply with all sponsor related obligations under the applicable laws[a]. .

Candidates that submit a proposal for funding of Belgian participation to an international trial, should submit a commitment letter signed by the international sponsor, stating that they accept the terms and conditions of the KCE Trials programme, including conditions regarding data sharing and IP rights.

5.1.2 Receipt of research outlines and verification that they are valid and within scope

All ROs received are collected and are initially reviewed by the KCE Trials team and categorised according to whether they are valid and appear to be within the scope of the call or not.

To be deemed valid and to enter the selection process, a submission should:

- Be received before the deadline mentioned in the call text
- Be submitted following the appropriate procedures mentioned in the call text and be readable
- Include details of chief investigator and candidate sponsor

[a] the law of May 7, 2004 or 2017(for drugs) or December 2020 (for the medical devices).

- Include a signed letter of support including acceptance of the terms and conditions of the KCE Research Agreement by a candidate sponsor who qualifies as non-commercial sponsor under the applicable laws
- Include a clearly defined research question in the PICO format (Participants, Intervention, Comparator, Outcome)
- Be a study with a non-commercial primary aim.
- In addition:
    - The candidate sponsor should qualify as non-commercial sponsor under the applicable laws, including the law of May 7, 2004 or equivalent e.g. if the sponsor is not located in Belgium.
    - The holder of the intellectual property rights on the studied intervention or comparator to which the experiment relates is neither directly nor indirectly the sponsor of the experiment.
    - The sponsor exercises the intellectual property rights to the concept of an experiment, its implementation and the scientific data resulting from it.
    - The funding granted under the research agreement complies with the European state aid rules and regulations.

If attachments are missing or there are issues with documents, applicants are notified as soon as possible in order to make a valid submission within the deadline specified by KCE Trials.

Detailed eligibility criteria and definition of scope will be published with each call.

All submitted projects are checked for eligibility and scope by the KCE Trials team. The categorisation performed by the Trials team is discussed by the Prioritisation Group (PG) who decides whether the ROs are eligible and in scope or not. ROs that don't meet the eligibility criteria or are not within scope, don't enter the selection and prioritisation process. For ROs deemed to be not eligible or out of scope by the PG, a letter is sent to the applicant via SmartSimple. In addition, the PG will decide on which ROs proceed to the Panel in case the number of eligible ROs is too high (see 5.1.3.1.)

5.1.3 Prioritisation

Valid and in scope ROs are then reviewed by KCE (Trials) health economists (HE) and by the KCE Trials panels.

5.1.3.1 Scoring for potential value for money by KCE health economist

KCE (Trials) HE review the ROs and score them according to the following criteria (this scoring is performed by at least two persons):

- Highest score: substantial cost savings are expected. Either substantial savings per patient for small populations or savings for large populations that are substantial because of the size of the population, fall within this category. Interventions with an equivalent effectiveness that result in relevant cost savings compared with existing alternatives also fall within this category.
- High score: Potential increased patient benefit comes at acceptable extra expense for society.
- Low score: It is very questionable whether the increased patient benefit comes at an acceptable extra expense for society. ROs that contain insufficient information to judge the expected return on investment will receive a low score.

Within each category, no further ranking is applied. Depending on the total number of in-scope ROs, those with a low score for the potential value for money criterion may not be discussed by the panel but already be excluded at this stage. A maximum of 15 proposals will be discussed by the panel.

5.1.3.2 Scoring for clinical relevance and need for evidence by Trials panel

A Trials panel is a group of health care professionals, including professionals from different medical areas with also some patient representativesthat assists the KCE Trials team in selecting the research questions that are most relevant for routine clinical practice and the Belgian healthcare system. A balanced representation of the Dutch speaking and French speaking part of the country is aimed for. All panel members submit a signed conflict of interest and confidentiality undertaking form. Panel members who have a conflict of interest for a given submission, will leave the room when that submission is discussed.

Trials Panels review and score the ROs for their clinical relevance and the need for (randomised) evidence according to the general and specific criteria of the call. Generally, this is done by having 2-3 rapporteurs for each RO who send a report and initial scores in advance and present their comments during the meeting. In addition, all ROs are evaluated by a patient representative from a patient perspective. The patient representative also gives his/her comments after the other rapporteurs. This is followed by a general discussion. Finally, all panel members in the room score the proposal using an anonymous voting system. ROs are ranked according to the mean score. The Trials panel may also suggest improvements to the research questions suggested in the outlines. Comments from the panel will be provided to the Trials Board (TB) and integrated together with the comments from the TB in the decision letters (see below).

5.1.3.3 Prioritisation of research outlines by the Prioritisation Group

Before the PG meeting, and if capacity is available, the KCE Trials team will perform a search for existing evidence and ongoing trials to inform the PG during the meeting.

The final list of prioritised ROs is selected by the PG. By default, the PG considers first the outlines with highest score for good clinical relevance score and highest value for money, then outlines with a high score for value for money and lastly the outlines with a low score for value for money, following the ranking by the panels (mean score) within each category. However, the PG has the liberty to change the prioritisation order of the outlines as suggested by the HE evaluation and the panel, by consensus during the PG meeting. If no consensus can be reached, the prioritisation order suggested by the HE evaluation and panel prevails. The maximum number of ROs that can be forwarded to the TB will be decided by the KCE Trials team and the PG, depending on the available budget.

5.1.3.4 Feedback to applicants

After the final prioritisation by the PG, all candidate research teams are informed about the result of the selection process. All feedback to the candidates will be anonymised, with a summary of the discussion at the Trials Panel and PG. The selected ROs are forwarded to the TB without revision by the research teams. However, if the information available in an RO is too limited, the PG can request the candidate research team to resubmit a more elaborated RO before it is forwarded to the TB.

5.1.4 Schematic diagram

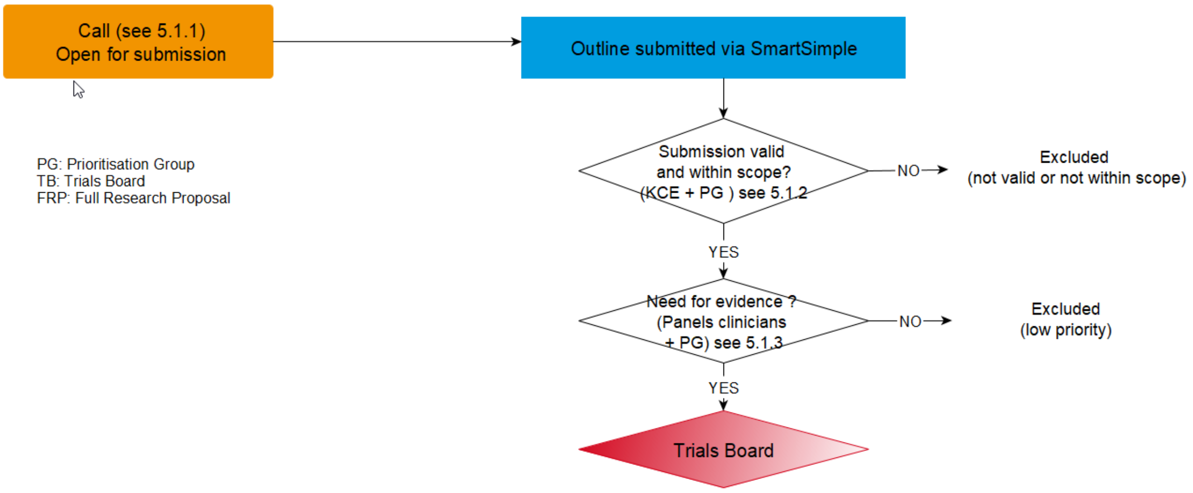

Fig 2 Call for proposals and their prioritisation

5.2 Review and shortlisting of research outlines by the Trials Board

5.2.1 Composition of the review

All members of the TB will review all prioritised ROs and can comment on all aspects of the submission. Generally, 3 members of the TB will be selected to act as rapporteurs for each RO. The rapporteurs will be selected so that ideally there is at least a clinical expert and a statistician, taking into account the conflict of interest policy. In addition, all ROs are evaluated by a patient representative from a patient perspective. The rapporteurs will be asked to provide their comments and preliminary scores through SmartSimple before the TB meeting and to present their comments during the meeting.

TB members are usually given 2-3 weeks to review the ROs, which are made available in SmartSimple. TB members can send their written comments in advance via SmartSimple, even when they cannot attend the meeting.

Before the TB meeting, a check is made that TB members don't have a conflict of interest (COI) with any of the CI-Sponsor teams.

5.2.2 Trials Board meeting

- Chair,– non-voting
- Head KCE Trials programme – non-voting
- Other KCE Trials team members – non-voting
- Rapporteurs (usually 3 per RO**) – voting*
- Other Members – voting*
- KCE Trials administrative support – non-voting
- Observers – non-voting

*if present in the meeting room for the specific vote

** 3 per brief for the commissioned workstream

KCE Trials members can give background e.g., summary of panel discussions or an update of information related to a specific research proposal and participate to the discussions. Members of the KCE Trials Team however, are non-voting.

Quorum will be judged for the whole meeting and not for individual agenda points. For the meeting to be quorum, 50% of the voting TB members must be in attendance. If the quorum is not reached, the meeting will be postponed.

Longstanding interests and potential COIs related to any of the outlines to be discussed will be registered prior to the meeting or at the latest during the meeting according to the applicable Policy. A probity report will be provided to the TB at the start of the meeting and will be reviewed by the TB.

Usually, the TB will decide to decline, decline but invite to resubmit or to shortlist a RO based on consensus. If this is not possible; a vote will be held.

If a vote is held, each voting member present in the room for the discussion, has to vote. The voting scale is:

6: Excellent
Proposed research can be shortlisted as it stands,

5: Good
Proposed research can be shortlisted with minor changes,

4: Good potential
There is much merit in this proposal, but it could only be shortlisted after resubmission, perhaps with additional external support,

3: Some merit
There are significant weaknesses in this application, but these could in principle be addressed

2: Poor
Weak application

1: Extremely poor
Unsupportable application

A median score of 5 or more means that the proposal will be shortlisted and invited to submit a full research proposal (FRP). A median score between 3 and 5 (including 3) means that the candidate will be invited to resubmit a RO. A median score below 3 means that the RO is declined. At the end of each discussion, points of feedback to the applicant are agreed upon by the Trials Board.

The minutes and other meeting documents (if applicable) are provided to the TB members via SmartSimple within two weeks after the meeting.

5.2.3 Communication of TB decisions

TB decision letters will be sent via SmartSimple to all applicants detailing the decision and summarising feedback, if applicable. For declined ROs, reasons for declining will be explained in the decision letter.

For the ROs invited to resubmit and shortlisted ROs, the applicants will be informed and provided with the timelines to submit their FRP.

The invitation to submit a FRP will also indicate the possibility to provide an advance payment of € 12.500 to support the development of the FRP. This request will be evaluated by KCE Trials upon its own discretion. The ad hoc funding will be deducted from the final total budget if recommended for funding. Projects that finally don't get funded do not have to return the money received.

For ROs which are declined but invited to resubmit, if the applicant decides to take into account the TB comments and resubmit an adapted RO, this should be done in time for review at the next scheduled TB meeting. The applicant will be informed of the timelines. In the resubmission, the applicant must address the comments of the TB and highlight any changes made.

5.3 Review and recommendation for funding of full research proposals by the Trials Board

Applicants whose ROs were shortlisted will be given a minimum of 6 weeks to submit a FRP.

The review of FRPs will proceed as described above for the ROs for members of the TB. In addition, for each FRP, there are usually between 1-3 (national or international) external reviewers who are not members of the TB (see KCET_POL_FU01_Confidentiality and potential conflict of interest). External reviewers who are not members of the TB are required to send their comments via SmartSimple before the TB meeting. External reviewers’ comments will be available to the rapporteurs and TB members in SmartSimple. The rapporteurs will integrate them into their comments or discuss them during the meeting.

5.3.1 Trials Board meeting

After the discussion of each FRP, the TB votes on whether to recommend a FRP for funding to the PG. All voting members present in the room must vote. The voting scale is:

6: Excellent
Proposed research can be funded as it stands,

5: Good
Proposed research can be funded with minor changes,

4: Good potential
There is much merit in this proposal, but it could only be funded after resubmission, perhaps with additional external support,

3: Some merit
There are significant weaknesses in this application, but these could in principle be addressed

2: Poor
Weak application

1: Extremely poor
Unsupportable application

Proposals with a median score of 6 are recommended to fund without changes and those with a median score of 5 are recommended to fund with changes. Proposals with a median above or equal to 3 and below 5 are requested to submit a revised FRP. Proposals with a median below 3 are declined.

At the end of each discussion, points of feedback to the applicant are agreed upon by the TB.

For those FRPs recommended to fund with changes, the changes requested by the TB are clearly detailed in the TB decision letter.

If the protocol has been accepted by the TB, an advance payment of €12.500 to perform a feasibility study can be requested. This possibility will be evaluated by KCE Trials upon its own discretion. The ad hoc funding will be deducted from the final total budget. Projects that finally don’t get funded do not have to return the money received.

5.3.2 Proposals invited to resubmit an updated FRP

For FRPs which are declined but invited to resubmit, if the applicant decides to take into account the TB comments and resubmit an adapted FRP, this should be done in time for review at the next scheduled TB meeting. The applicant will be informed of the timelines. In the resubmission, the applicant must address the comments of the TB and highlight any change made. For resubmitted FRPs, no second round of external review will be organised.

5.3.3 Full research proposals which are recommended for funding with changes

For FRPs which are recommended for funding with changes, there will usually be a meeting at KCE with the applicant after the TB meeting. The applicant must decide whether they are willing to make the specified changes. If so, they should inform the KCE Trials team within 4 weeks of the TB notification. If the project is proposed for funding by the PG, see below, the changes can be implemented in parallel with contract negotiations, additional feasibility checks and budget review. The KCE Trials team will have to receive any updated documents e.g. protocol versions and confirm that the requested changes have been made prior to contract signature (see KCET-SOP-FU-001 steps from trials board recommendation to fund to first patient in).

5.4 Prioritisation of full research proposals by the Prioritisation Group

The PG will review the proposals recommended for funding (with or without changes) by the TB. The PG will consider whether recommendations can be supported based on the final prioritisation and consideration of the available KCE budget for the Trials programme. Shortly after the TB, each project recommended for funding will be discussed by the PG. If the project probably will be funded taking into account the overall portfolio and finances, the project team can go ahead with the finalisation of the protocol and budget and the feasibility study. The PG will consider the project for funding when the protocol including all changes requested by the TB and budget are available. If approved, the PG will submit its final recommendations for funding to the KCE Board for approval.

5.5 Schematic diagram (from RO shortlisting to FRP prioritisation)

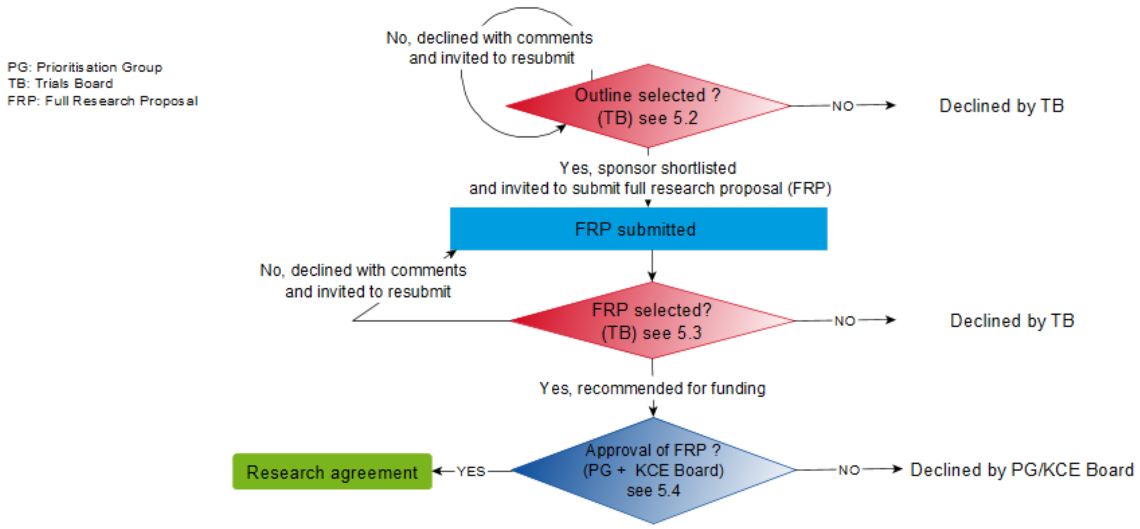

Fig 3 From TB review of the ROs to approval

5.6 International trials with sponsor abroad

International trials with a Belgian sponsor with the majority of participants recruited in Belgium follow the procedure as described above.

International trials with only a minority of participants recruited in Belgium that have not started recruitment in Belgium at the time of the submission deadline are eligible for the investigator-led call.

Review of ROs for international trials will be included in the review by the KCE Trials panel and prioritisation by the PG as other proposals. If selected, ROs for trials with sponsors outside of Belgium will not be forwarded to the TB as protocols for international trials are usually written in consensus with the international partners. The final draft protocol and budget for Belgium participation has to be submitted to the TB within the timelines of the call (5.7). Protocols for international trials will be reviewed by the TB once, in a ‘take it or leave it’ fashion. Exceptionally, a second review by the TB can be done, if requested changes still can be implemented by the international sponsor. Decisions regarding to recommend an international trial for funding will be taken by consensus and majority vote if no consensus can be reached.

For international trials with sponsor abroad, no ad hoc funding for FRP development will be provided. If the protocol has been accepted by the TB, an advance payment to perform a feasibility study can be requested.

5.7 Timelines/End of call

A maximum of 5 rounds of TB review will be organised for each call. If a project is not recommended for funding during the last TB meeting of a call, the project is declined but could be resubmitted for another call. Proposals resubmitted to a future call, have to follow the same selection process as the other proposals submitted to the call i.e. start at the beginning again.

5.8 Possibility to postpone, suspend and/or withdraw any pending research call

KCE shall remain entitled at all times to postpone, suspend and/or withdraw any pending research call (even during the negotiation of the research agreement) at its own discretion; KCE shall under no circumstances be obliged to select any pending FRP or enter into a research agreement after FRP selection. Candidate sponsors can withdraw their submission at any time before signature of the research agreement.

5.9 Pilot studies

Proposals may include an internal pilot study. Furthermore, the Panel or the TB may advise to a candidate research team to first perform a pilot study before embarking on a full RCT. In each case, the protocol of the pilot study must go through the full review and prioritisation cycle by the TB and PG, as for other protocols. Research teams can ask for advance payment to develop the FRP of the pilot study.

The budget and funding of a pilot study will be approved by the PG independently from the approval of the full RCT. When results of the pilot study are available, they should be presented to the PG. The PG then decides if the RCT would be eligible for funding. If accepted by the PG, the protocol of the full RCT should be submitted to the TB for review, as for other protocols. An advance payment for the development of the full protocol will only be considered if no advance payment for the pilot was requested. Feasibility visits together with the CRO will not be performed in case of a pilot study.

6 REFERENCES

SmartSimple Grants Management Platform (including forms for applicants, for reviewers, and templates for decisions letters)

7 APPENDICES

KCET_POL_FU_001: COI policy

KCET_SOP_CA_002: commissioned workstream

KCET-SOP-FU-001 steps from trials board recommendation to fund to first patient in

Templates decision letters for PG and TB (declined, resubmission, recommended for funding, selection)

KCET_TEMP_SE_024_KCE TRIALS, EXTERNAL REVIEWERS CONFLICT OF INTEREST AND CONFIDENTIALITY UNDERTAKING FORM

KCET_temp_SE_010_Meeting Draft probity report (for TB, panel or PG)

END OF DOCUMENT
