# Supplementary figures and images for: Treatment for preschool age children who stutter: Protocol of a randomised, non-inferiority parallel group pragmatic trial with Mini-KIDS, social cognitive behaviour treatment and the Lidcombe Program—TreatPaCS
Source: PLoS One. 2024 Jul 11;19(7):e0304212. doi: 10.1371/journal.pone.0304212 (PMC11239023; doi:10.1371/journal.pone.0304212)

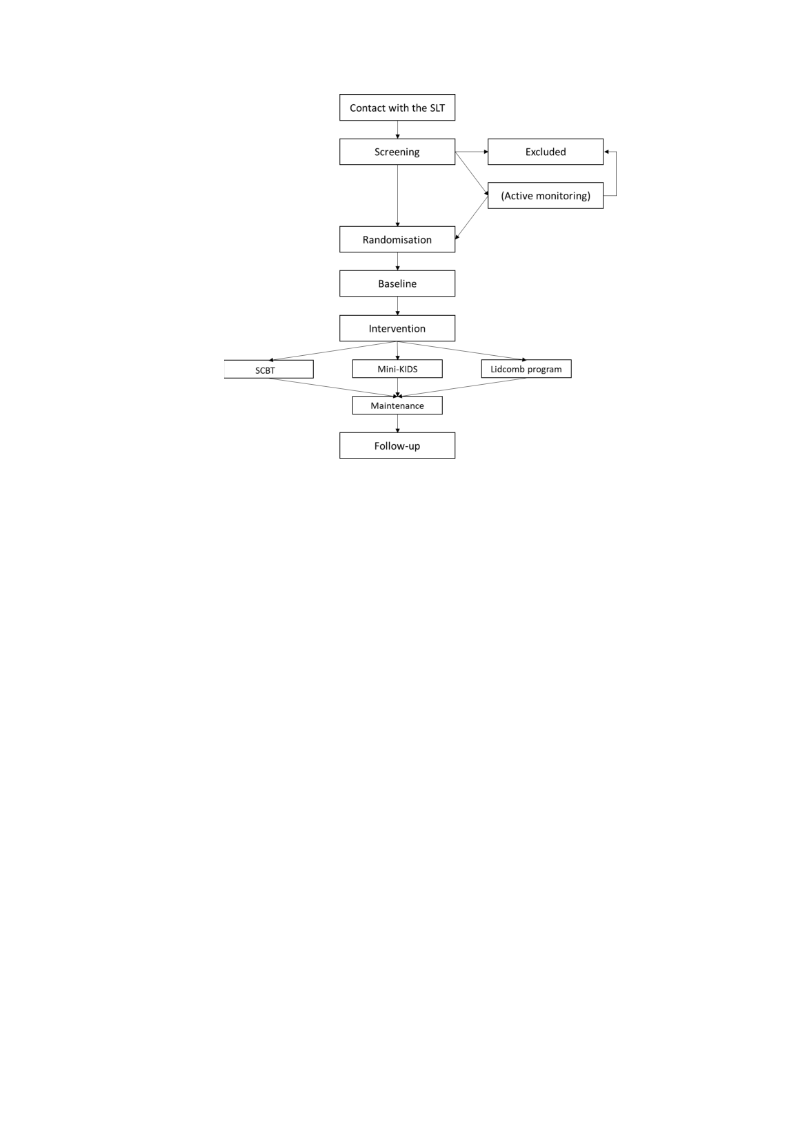

Supplement: S1 Fig — (TIF) [file pone.0304212.s007.tif]

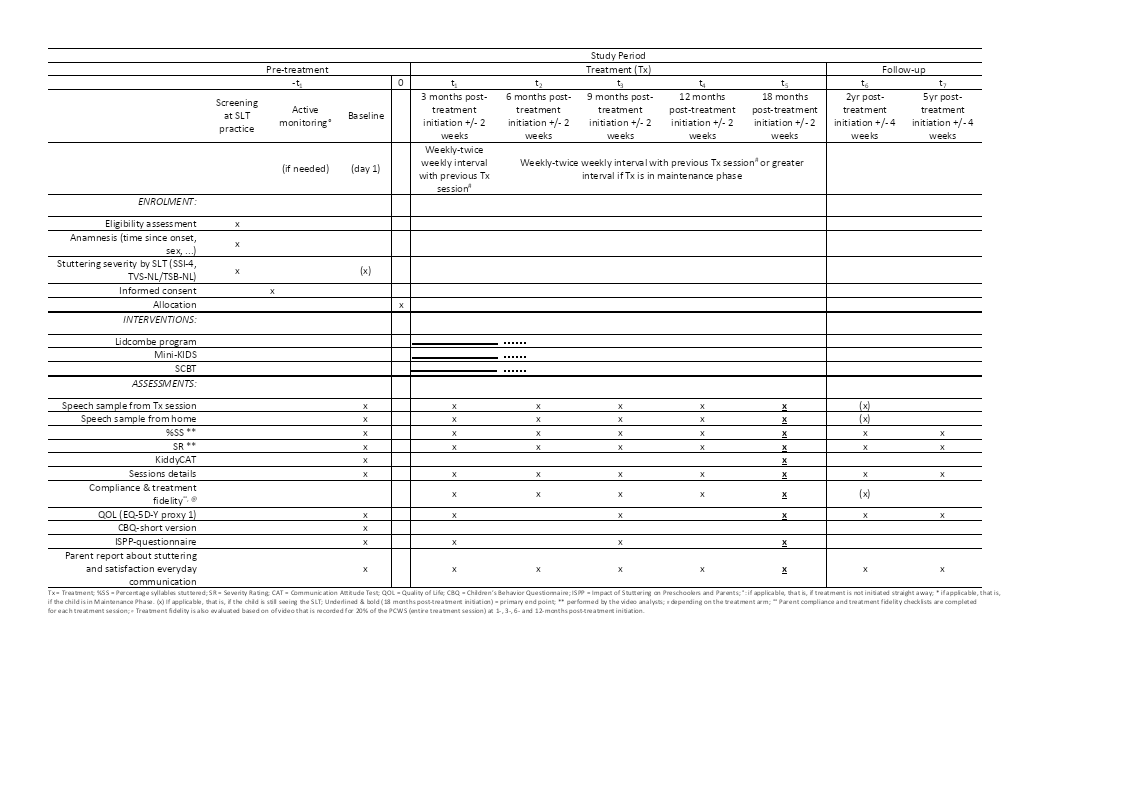

Supplement: S2 Fig — (TIF) [file pone.0304212.s008.tif]
